# Comparative Analysis of Microbial Community and Metabonomics in Traditional Sour Meat from Different Regions of China

**DOI:** 10.3390/foods15193424

**Published:** 2026-09-24

**Authors:** Qing Guo, Shijie Liu, Qian Ding, Miaoyun Li, Dong Liang, Lijun Zhao, Yanxia Liu

**Affiliations:** 1 College of Food Science and Technology, Henan Agricultural University, Zhengzhou 450002, China; guoqing0312@163.com (Q.G.); 15938978937@163.com (S.L.); 17337134960@163.com (Q.D.); liangdong0221@126.com (D.L.); zhaolj2014@126.com (L.Z.); liuyanxia@henau.edu.cn (Y.L.); 2 International Joint Laboratory of Meat Processing and Safety in Henan Province, Henan Agricultural University, Zhengzhou 450002, China

**Keywords:** fermented sour meat, regional differences, microbial community, untargeted metabolomics, differential metabolites, KEGG

## Abstract

To characterize the microbial communities and metabolomic profiles of traditional sour meat from different regions of China, sour meat samples collected from Guangxi, Hunan, Guizhou, and Yunnan, together with laboratory-prepared sour meat (LSM), were analyzed using 16S rRNA gene amplicon sequencing and untargeted metabolomics. Significant regional differences were observed in both microbial community composition and metabolite profiles. Firmicutes were the predominant bacterial phylum in most samples, whereas Proteobacteria showed relatively higher abundances in Guizhou sour meat (GZSM) and Yunnan sour meat (YNSM). At the genus level, *Lactobacillus* predominated in Guangxi sour meat (GXSM) and Hunan sour meat (HNSM), while GZSM and YNSM exhibited distinct bacterial community characteristics with enrichment of specific genera. Metabolomic analysis identified substantial differences in metabolite composition among regions, and the differential metabolites were mainly associated with amino acid, carbohydrate, and lipid metabolic pathways. Overall, traditional sour meat from different geographical origins exhibited pronounced regional differentiation in microbial and metabolomic characteristics. These findings provide new insights into the regional characteristics of traditional Chinese sour meat and support future studies on microbial resources, quality evaluation, and product standardization.

## 1. Introduction

Traditional fermented meat products constitute a vital part of China’s specialty meat products, characterized by a long processing history and distinctive regional cultural features [1,2]. Sour meat, a representative traditional fermented meat product in southern China, is typically produced from pork through salting, supplementation with cereals or starchy adjuncts, and spontaneous fermentation. During fermentation, macromolecular substances in the raw materials, including proteins, lipids, and carbohydrates, undergo continuous degradation and biotransformation under the action of endogenous enzymes and microbial metabolism, resulting in the formation of lactic acid, amino acids, fatty acids, peptides, nucleotides, and other volatile and non-volatile flavor compounds, which collectively impart the unique sour, meaty, and umami characteristics of sour meat [3,4]. Traditional sour meat production is generally characterized by diverse raw material sources, complex processing environments, non-standardized fermentation conditions, and reliance on spontaneous microbial fermentation. Consequently, sour meat from different geographical origins exhibits pronounced variations in sensory quality, physicochemical properties, and flavor composition [5]. With increasing consumer interest in traditional specialty foods, regional foods, and fermented foods, systematic elucidation of the quality formation basis and regional differences in traditional sour meat from different regions is of great significance for the exploration of traditional fermented meat resources, the establishment of quality standards, and the modernization of production processes.

The microbial community serves as a critical driving force during the fermentation of traditional sour meat, with its composition and dynamic succession directly influencing substrate degradation, acidification processes, and flavor compound formation [6,7]. Sour meat is produced through an open, spontaneous fermentation system, in which the microbial community is shaped by multiple factors, including raw material characteristics, salt concentration, fermentation temperature, environmental microbiota, processing facilities, and traditional practices. This typically results in a complex microecosystem comprising lactic acid bacteria, staphylococci, micrococci, and other bacteria and fungi [5,8]. Therefore, differences in microbial community composition and dominant populations among sour meat from different regions may constitute an important reason for the variations in their metabolomic profiles and quality attributes [3]. Previous studies on traditional Chinese sour meat have primarily focused on samples from individual regions, including analyses of microbial communities, physicochemical properties, flavor characteristics, and dominant functional microorganisms. Although several studies have reported regional differences among fermented meat products, most investigations have examined either microbial communities or metabolite profiles separately. Comprehensive cross-regional studies integrating microbiome and metabolome analyses remain limited. Consequently, the relationships between microbial community variation and metabolic differentiation among sour meat from different traditional fermentation systems are still poorly understood.

China has a vast territory, and traditional sour meat produced in different regions exhibits substantial variation in raw materials, seasoning formulations, fermentation duration, processing practices, and environmental conditions. These factors collectively shape region-specific fermentation ecosystems and microbial communities. Therefore, the term “regional differences” in the present study refers not only to geographical location itself but also to the combined effects of local production practices and environmental conditions associated with different traditional fermentation systems [9]. Previous studies have indicated that the regional characteristics of traditional fermented foods are not merely reflected by simple differences in dominant microorganisms, but rather represent an integrated outcome of interactions among microbial communities, substrate utilization, metabolic pathways, and metabolites [10,11,12]. In the case of sour meat, comparisons of microbial abundance alone are insufficient to explain the regional differences in sourness, umami, meaty aroma, and overall flavor profiles [7]. Therefore, beyond comparing the microbial community structures of sour meat from different regions, it is necessary to further dissect their functional differences at the metabolic level, thereby revealing the formation basis of the regional characteristics of traditional sour meat. Metabolomics enables the holistic characterization of the composition and variation in small-molecule metabolites in biological systems and has emerged as a powerful tool for elucidating the quality formation mechanisms of complex fermented foods [13,14]. Metabolomics can not only reveal differences in overall metabolic profiles among products from different regions, but also further clarify the potential roles of amino acid metabolism, lipid metabolism, carbohydrate metabolism, and organic acid metabolism in region-specific quality formation [15]. Consequently, the integrated analysis of microbiome and metabolome is expected to overcome the limited explanatory power of individual omics approaches in addressing the regional differences in sour meat.

Therefore, this study first identified and compared the microbial community structure of traditional sour meat in Guangxi, Hunan, Guizhou, and Yunnan, and took the sour meat produced in the laboratory (Zhengzhou, Henan) as a reference. Secondly, non-targeted metabolomics is used to identify the metabonomic characteristics and differential metabolic pathways related to the region. Finally, the relationship between microbial community composition and metabolic differentiation in different traditional fermentation systems was explored. By integrating microbiome and metabolome analyses, this study provides new insights into the mechanisms underlying regional quality characteristics of traditional Chinese sour meat and offers a scientific basis for microbial resource exploitation, quality control, and industrial standardization.

## 2. Materials and Methods

### 2.1. Sample Collection

A total of 20 sour meat samples were collected from five groups, including Guangxi (GXSM), Hunan (HNSM), Guizhou (GZSM), Yunnan (YNSM), and laboratory-produced sour meat (LSM). LSM was independently prepared in our laboratory in Henan Province using pork under controlled fermentation conditions. Briefly, 30% rice meal and 8% salt were added to the meat, followed by thorough mixing. The mixture was then subjected to fermentation at 25 °C for 60 days. A total of four independent fermentation batches were prepared, and each batch was treated as an independent biological replicate. The complete formulation and fermentation procedure were standardized across all laboratory batches. For each group, four independent sour meat samples (n = 4) were obtained from different producers and independent fermentation batches. Traditional sour meat samples were collected from representative sour meat-producing regions in Liuzhou (Guangxi Province), Xiangxi (Hunan Province), Kaili (Guizhou Province), and Pu’er (Yunnan Province) between May and July 2024. The laboratory sour meat samples were prepared independently in our laboratory in Henan Province according to a standardized fermentation procedure. Approximately 200 g of sour meat was collected from each batch using sterile sampling procedures and individually packaged in sterile polyethylene bags. Samples were transported to the laboratory in insulated containers containing ice packs within 24 h and stored at −80 °C until microbiome and metabolomics analyses. All samples were analyzed individually and were not pooled. For microbiome and metabolomic analyses, each sour meat sample was considered an independent biological replicate. Any repeated LC–MS injections were treated as technical replicates and were used solely for instrument stability assessment.

### 2.2. DNA Extraction, PCR, and High-Throughput Sequencing

Microbial genomic DNA was extracted using the TIANamp Bacteria DNA Kit (TIANGEN, Beijing, China) according to the manufacturer’s instructions [16]. The V3–V4 hypervariable region of the bacterial 16S rRNA gene was amplified using the universal bacterial primers 338F (5′-ACTCCTACGGGAGGCAGCAG-3′) and 806R (5′-GGACTACHVGGGTWTCTAAT-3′). The PCR mixture contained 12.5 μL of 2 × PCR Master Mix, 8.5 μL of ddH_2_O, 2 μL of each primer, and 2 μL of template DNA. The PCR thermal cycling conditions were as follows: initial denaturation at 95 °C for 5 min; 30 cycles of denaturation at 94 °C for 1 min, annealing at 58 °C for 1 min, and extension at 72 °C for 1 min; followed by a final extension at 65 °C for 15 min. The PCR products were verified by 1% agarose gel electrophoresis, purified, and subjected to library preparation and paired-end sequencing on the Illumina MiSeq platform by Shenzhen Huada Gene Technology Co., Ltd. (BGI, Shenzhen, China). The paired-end reads were quality-filtered and merged based on their overlapping regions before downstream microbial community analysis.

### 2.3. Sequence Analysis

Raw sequencing reads were quality-filtered to remove low-quality sequences. Samples that failed to meet quality-control requirements were re-sequenced when necessary. High-quality reads were assigned to individual samples according to their unique barcode and index sequences, after which barcode sequences were trimmed. Sequence denoising and quality control were initially performed using the DADA2 plugin in QIIME2. Chimeric sequences were identified and removed using the consensus method implemented in DADA2. The resulting high-quality sequences were subsequently clustered into operational taxonomic units (OTUs) at a 97% sequence similarity threshold using VSEARCH 2.30.0. To minimize biases caused by uneven sequencing depth, the OTU table was rarefied to the minimum sequencing depth across samples prior to diversity analyses. Taxonomic assignment was performed against the SILVA 138 reference database using a Naïve Bayes classifier with a confidence threshold of 0.7. The taxonomic composition of each sample was characterized at different taxonomic levels (phylum, class, order, family, genus, and species). Alpha-diversity metrics, including Observed Species, Chao1, Shannon, and Simpson indices, were calculated based on the rarefied OTU table using QIIME2 and visualized using R software 3.2.3 [17].

### 2.4. Non-Targeted Metabolomics Sample Preparation

Accurately weighed minced samples (50.00 mg) were placed in pre-cooled 2 mL centrifuge tubes, and 400 μL of a methanol/water extraction solution (4:1, *v*/*v*) containing 0.02 mg/mL L-2-chlorophenylalanine as an internal standard was added for metabolite extraction. The sample solution was homogenized using a frozen tissue grinder at 50 Hz for 6 min at −10 °C, followed by ultrasonication-assisted extraction at 40 kHz for 30 min in a 5 °C water bath. After extraction, the samples were incubated at −20 °C for 30 min, and then centrifuged at 13,000× *g* and 4 °C for 15 min. The supernatants were transferred to autosampler vials for instrumental analysis [18].

### 2.5. Non-Targeted Metabolomics Analysis

Chromatographic separation was performed using an ultrahigh-performance liquid chromatography system coupled to a Q Exactive HF-X hybrid quadrupole-Orbitrap mass spectrometer (Thermo Fisher Scientific, Bremen, Germany). Metabolites were separated on a column model, ACQUITY UPLC HSS T3, 2.1 × 100 mm, 1.8 μm maintained at 40°C. Mobile phase A consisted of water containing 0.1% formic acid, and mobile phase B consisted of acetonitrile containing 0.1% formic acid. The flow rate was [0.30] mL/min, and the injection volume was 5 μL. The following gradient was applied: 0–1 min, 2% B; 1–3 min, 2–10% B; 3–8 min, 10–30% B; 8–12 min, 30–50% B; 12–15 min, 50–95% B; 15–17 min, 95% B; 17–17.1 min, 95–2% B; and 17.1–20 min, 2% B for column re-equilibration. The total chromatographic run time was 20 min per injection. The mass spectrometer was operated in both positive and negative electrospray ionization (ESI) modes. Full-scan MS spectra were acquired over an *m*/*z* range of 100–1500 at a resolving power of 120,000 (FWHM at *m*/*z* 200). The automatic gain control (AGC) target was set to 3 × 10^6^, with a maximum injection time of 100 ms. For data-dependent MS/MS acquisition, the top −5/10 most intense precursor ions were selected for fragmentation, with an isolation window of 1.0 m/z and a resolving power of [60,000]. Fragmentation was performed using stepped normalized collision energy (NCE) of 20, 40, and 60. The sheath gas and auxiliary gas were set to 30 and 20, respectively, using the instrument’s specified gas-flow units rather than L/min. The spray voltage was set to +3.5 kV in positive mode and −2.5 kV in negative mode, and the capillary temperature was maintained at 350 °C.

A pooled quality-control (QC) sample was prepared by mixing equal aliquots of all individual sample extracts and was used to assess system stability and analytical reproducibility. Before analysis of the study samples, the pooled QC sample was injected 3 times to condition the chromatographic system. During the analytical sequence, QC samples were injected after every 5 study samples. Study samples were randomized prior to injection to minimize systematic effects associated with injection order. Solvent blanks were analyzed at the beginning of the sequence and periodically throughout the run to monitor background contamination. Additional blank injections were performed after QC or representative high-response samples to evaluate potential carryover. Carryover was considered acceptable when the peak areas of major features in the subsequent blank were less than 20% of those observed in the preceding sample/QC injection. Analytical stability was evaluated based on the total ion chromatogram (TIC), base-peak chromatogram (BPC), mass accuracy, and relative standard deviations (RSDs) of detected features in pooled QC samples. Features showing an *RSD* > 30% across QC injections were excluded from downstream statistical analyses. Raw LC–MS data were processed using Compound Discoverer version 3.1. The workflow included peak detection, chromatographic peak alignment, grouping of ion features, blank subtraction, adduct and isotope grouping, and metabolite annotation. Features detected in solvent blanks were removed when their signal intensities exceeded 30% of those observed in biological samples. Missing values were handled by half of the minimum detected value. Features with excessive missingness (>30% of the study samples) were removed before statistical analysis. Peak intensities were normalized using the internal standard L-2-chlorophenylalanine and, where applicable, corrected using pooled QC-based signal drift correction. QC-based normalization was performed against acquisition time using linear regression to reduce signal drift during the analytical sequence. Thermo Fisher’s Compound Discoverer workflow supports pooled-QC-based correction using regression functions and QC *RSD* criteria. After normalization, the feature matrix was log2-transformed and Pareto-scaled before multivariate statistical analysis [19].

### 2.6. Data Processing and Statistical Analysis

Raw metabolomic data were processed using Compound Discoverer (Thermo Fisher Scientific, Waltham, MA, USA). Peak detection, retention-time alignment, deconvolution, and normalization were performed using the default workflow. Metabolic features with a relative standard deviation (*RSD*) >30% in quality-control (QC) samples were removed to improve data reliability. Signal drift and batch effects were monitored using pooled QC samples that were analyzed throughout the analytical sequence. Metabolite annotation was conducted by matching accurate mass, isotope distribution, and MS/MS fragmentation patterns against public databases, including HMDB, KEGG, and mzCloud. Only metabolites meeting the established identification criteria were retained for downstream analyses. For microbial community analyses, alpha-diversity indices (Observed Species, Chao1, Shannon, and Simpson) were calculated using QIIME2 Compound Discoverer 3.0. Differences among groups were assessed using one-way analysis of variance (ANOVA) followed by Tukey’s multiple-comparison test. Beta-diversity was evaluated using Bray–Curtis distance matrices and visualized by non-metric multidimensional scaling (NMDS). Statistical significance was assessed using PERMANOVA with 999 permutations. Linear discriminant analysis effect size (LEfSe) was performed to identify significantly enriched microbial taxa among groups using a logarithmic LDA score threshold of 3.0 and a significance level of *p* < 0.05. Multivariate analyses of metabolomic data, including principal component analysis (PCA) and partial least-squares discriminant analysis (PLS-DA), were conducted using SIMCA software (version 2025). Model robustness was evaluated using 200 permutation tests. Differential metabolites were identified based on a combination of variable importance in projection (*VIP*) scores derived from the PLS-DA model, fold change (FC), and univariate statistical analysis. Metabolites satisfying VIP > 1.0, FC ≥ 2.0 or FC ≤ 0.5, and *p* < 0.05 were considered significantly different. False discovery rate (FDR) correction was applied where appropriate.

## 3. Result and Analysis

### 3.1. Alpha and Beta Diversity Analyses of Microbial Communities

The alpha diversity indices of the microbial communities are presented in Figure 1A–C. The Chao1 index was used to characterize species richness, while the Shannon and Simpson indices reflected species diversity and evenness [20]. The GZSM samples exhibited the highest Chao1, Shannon, and Simpson indices among all groups, significantly exceeding those of the other groups (*p* < 0.05), indicating that the GZSM samples possessed the greatest microbial species richness and community diversity. The LSM samples also maintained relatively high alpha diversity levels. In contrast, the HNSM samples showed the lowest Chao1 and Shannon indices which were 538.412 and 3.92104, respectively, and the Simpson index was 0.76757 was also significantly lower than those of the other groups, suggesting that the HNSM samples harbored the fewest microbial species and the lowest community diversity. The YNSM and GXSM samples exhibited moderate alpha diversity levels, with the GXSM diversity being slightly lower than that of YNSM. These alpha diversity results demonstrated that the microbial community richness and diversity of sour meat vary significantly among different geographical origins.

Non-metric multidimensional scaling (NMDS) analysis based on Bray–Curtis distances were performed to elucidate the differences in microbial community structure among sour meat samples from the five regions, as shown in Figure 1D. The stress value of the NMDS model was 0.0202, which was below 0.05, indicating that the ordination results were reliable. Samples from the same geographical origin clustered closely together, whereas those from different origins were distinctly separated. These results indicate that microbial community composition differed among sour meat samples from different regions and exhibited apparent regional clustering patterns. However, NMDS is primarily a visualization method and does not by itself establish geographical origin as a causal driver of community differentiation.

### 3.2. Analysis of Microbial Community Composition

The phylum-level relative abundances of microbial communities in sour meat samples from the five regions are presented as a bar plot in Figure 2A. *Firmicutes* were the overwhelmingly dominant phylum across all samples, accounting for approximately 98% of the total community in GXSM, HNSM, and LSM samples. In contrast, the relative abundance of Firmicutes decreased in GZSM (~73%) and YNSM (~66%) samples. Correspondingly, the relative abundance of Proteobacteria was higher in GZSM and YNSM samples, reaching approximately 34% in YNSM and 23% in GZSM, while a lower abundance was observed in HNSM. In contrast, Proteobacteria occurred at very low levels in GXSM and LSM. All other phylum were present at low relative abundances and were collectively categorized as “others”. Collectively, these results revealed distinct differences in the phylum-level microbial community composition among sour meat samples from different geographical origins. Firmicutes remained the predominant phylum across all groups, while Proteobacteria exhibited relatively higher abundances in GZSM and YNSM than in the other geographical groups. GXSM, HNSM, and LSM were characterized by a particularly high predominance of Firmicutes.

The order-level community structure was further resolved using stacked bar plots, as shown in Figure 2B. Lactobacillales was the predominant order, accounting for extremely high proportions in HNSM and GXSM samples. In LSM samples, in addition to Lactobacillales, a considerable proportion of Bacillales was also observed. GZSM samples exhibited the highest community diversity at the order level, with multiple orders including Enterobacteriales and Clostridiales present at appreciable abundances alongside Lactobacillales. In YNSM samples, the abundance of Enterobacteriales increased markedly, corroborating the high abundance of Proteobacteria observed at the phylum level. LSM samples contained a relatively high proportion of Gemellales, whereas GXSM samples exhibited the simplest community composition, consisting almost entirely of Lactobacillales. These results demonstrated that geographical origin drives the differentiation of microbial community structure at the order level, with GXSM and HNSM exhibiting simpler community structures and GZSM and YNSM displaying greater community complexity.

The family-level community composition is shown in Figure 2C, revealing distinct regional differences among sour meat samples. *Lactobacillaceae* predominated in HNSM and GXSM, accounting for approximately 85% and nearly 100% of the relative abundance, respectively. LSM exhibited a more heterogeneous composition, with *Lactobacillaceae* accounting for approximately 58% and additional contributions from *Staphylococcaceae* and *Moraxellaceae*. GZSM displayed a relatively diverse family-level profile, with *Lactobacillaceae* as the predominant family alongside several other bacterial families. In contrast, *Streptococcaceae* was predominant in YNSM, accompanied by substantial contributions from *Enterobacteriaceae* and *Lactobacillaceae*. Overall, the family-level profiles differed markedly among geographical groups, highlighting distinct patterns of microbial community composition in sour meat from different regions.

The genus-level community composition of sour meat samples from different geographical origins is presented in Figure 2D. Distinct differences in the relative abundances of dominant genera were observed among the geographical groups. *Lactobacillus* predominated in LSM and HNSM and remained the major genus in GXSM. In contrast, GXSM contained a greater variety of low-abundance genera. GZSM exhibited a relatively heterogeneous genus-level composition, with several genera contributing substantially to the community, whereas YNSM was characterized by relatively high abundances of *Lactococcus* and *Lactobacillus*, together with other less abundant genera. Overall, the genus-level community profiles varied considerably among geographical groups, indicating distinct microbial assemblages associated with sour meat from different regions.

### 3.3. Species Differentiation Analysis

A Venn diagram based on operational taxonomic unit (OTU) levels was constructed to analyze the shared and unique OTUs among sour meat samples from five different regions (Figure 3A). The five groups shared 16 OTUs, accounting for 0.09% of the total OTU count, whereas a large number of OTUs were unique to individual groups. This pattern suggested considerable differences in OTU membership among sour meat samples from different geographical origins. However, the occurrence of shared OTUs across all groups should not be interpreted as evidence of a biologically stable core microbiome because neither prevalence within groups nor relative abundance was considered in this analysis. Meanwhile, each group harbored a substantial number of unique OTUs, with LSM possessing the highest number at 6000 (32.92%), followed by GZSM with 5492 (30.13%), YNSM with 3240 (17.78%), GXSM with 1597 (8.76%), and HNSM with 852 (4.67%). Additionally, varying numbers of shared OTUs were observed among pairs, triples, and quadruples of groups. These results suggested substantial variation in OTU membership among geographical groups. Nevertheless, the large number of group-specific OTUs may partly reflect the contribution of low-abundance taxa detected in individual samples.

Linear Discriminant Analysis Effect Size (LEfSe) analysis was performed to identify taxonomically differentiated species with statistical significance among groups [21]. The LDA bar plot illustrated the differentially abundant taxa enriched in each group (Figure 3B). At the phylum level, Firmicutes and Proteobacteria were the dominant phyla across all samples, whereas other phyla were present at relatively low abundances. LEfSe analysis identified Firmicutes and Proteobacteria, with LDA scores (log10) greater than 4.0, as important discriminatory taxa among the sample groups. At the order level, a total of 20 bacterial orders were detected. Burkholderiales was identified as a significantly enriched taxon in specific groups, whereas Lactobacillales remained the predominant order according to the relative abundance analysis presented in Figure 2B. At the family level, GXSM was characterized by Lactobacillaceae, YNSM by Streptococcaceae, GZSM by Enterococcaceae and Moraxellaceae, HNSM by Enterobacteriaceae and Leuconostocaceae, and LSM by Staphylococcaceae. These results indicate that sour meat samples from different geographical origins possessed distinct microbial biomarkers, further supporting the regional differentiation of microbial community composition.

### 3.4. Comparative Metabolomic Analysis

To elucidate the overall differences and regional characteristics of the metabolomic profiles of traditional sour meat from different regions, multivariate statistical analyses were performed on the metabolomic data of GXSM, HNSM, GZSM, YNSM and LSM samples (Figure 4). The PCA results, shown in Figure 4A, revealed a clear spatial separation among sample groups in the two-dimensional score plot, with PC1 and PC2 accounting for 39.80% and 22.90% of the total variance, respectively, and a cumulative explained variance of 62.70%, indicating that the first two principal components adequately captured the major variation in metabolomic composition among sour meat samples from different regions. To further maximize discrimination among regional sour meat samples, a supervised PLS-DA model was established (Figure 4B). Samples from the five regions formed distinct clusters with minimal overlap, demonstrating substantial differences in metabolite composition among geographical origins. Model robustness was further evaluated using a permutation test with 200 random permutations (Figure 4C). The intercepts of the regression lines were R^2^ = 0.2612 and Q^2^ =−0.8909. In addition, all permuted Q^2^ values were lower than that of the original model, and the negative Q^2^ intercept suggested that the model was not overfitted. The horizontal axis in the permutation plot represents the correlation coefficient between the original and permuted class assignments, with lower values indicating a greater degree of randomization. Together, these results support the stability and reliability of the PLS-DA model. An UpSet plot was further employed to analyze the shared and unique metabolite compositions among sour meat samples from the five regions (Figure 4D). The numbers of detected metabolites in GXSM, HNSM, GZSM, YNSM, and LSM samples were 2434, 2340, 2405, 1941, and 2531, respectively, with 1411 metabolites commonly detected across all five regions, indicating a substantial shared metabolomic foundation among sour meat samples from different origins, reflecting the conserved core metabolic network derived from pork raw material composition and the traditional sour meat fermentation process [22]. Meanwhile, each region still harbored a considerable number of region-specific or combinatory-specific metabolites, suggesting that while geographical factors maintain the common metabolomic features of sour meat, they also impart distinctive metabolomic fingerprints [23]. Overall, traditional sour meat from different regions of China exhibited significant regional differentiation in metabolomic profiles, characterized by shared core metabolite compositions alongside region-specific metabolomic signatures, providing a crucial basis for screening differential metabolites and elucidating key metabolic pathways.

### 3.5. Overview of Metabolites

To further characterize the chemical diversity of traditional sour meat, annotated metabolites were classified using KEGG and HMDB databases (Figure 5). Among the metabolites successfully assigned to KEGG chemical categories, amino acids represented the largest category (16 compounds), followed by organic acid carboxylic acids (11 compounds), phospholipids (8 compounds), and fatty acids (8 compounds) (Figure 5A). These categories are commonly associated with protein degradation, lipid metabolism, and microbial biotransformation processes occurring during fermentation. Based on HMDB secondary classification (Figure 5B), carboxylic acids and derivatives represented the largest proportion of classified metabolites (23.06%), followed by other metabolites (15.47%), fatty acyls (11.14%), organooxygen compounds (8.95%), and prenol lipids (8.38%). At the tertiary level (Figure 5C), amino acids, peptides, and analogs accounted for 21.60% of classified metabolites, followed by carbohydrates and carbohydrate conjugates (6.52%) and fatty acids and conjugates (3.54%) [5,24]. Overall, the metabolite profile of traditional sour meat was dominated by amino acid-, organic acid-, and lipid-related compounds. Although these metabolite classes have been reported to participate in flavor formation pathways during meat fermentation, the present classification analysis alone does not provide direct evidence for their specific contributions to sourness, umami taste, or aroma characteristics. Further quantitative metabolite–flavor association analyses would be required to establish such relationships.

### 3.6. Differential Metabolite Analysis

To further elucidate the differences in metabolite composition between traditional sour meat from different regions and laboratory-scale fermented sour meat, differential metabolite analyses were performed using LSM as the reference, comparing GXSM, HNSM, GZSM, and YNSM individually in Figure 6. Detailed annotation information was provided in Table S1. Metabolite annotation was performed by matching accurate mass, isotope pattern, and MS/MS spectra against public and commercial databases. Metabolite identification confidence was assigned according to the Metabolomics Standards Initiative (MSI) guidelines [25]. As shown in Figure 6A, a substantial number of metabolite differences were observed between GXSM and LSM, with a total of 1073 differential metabolites identified, among which 603 were upregulated and 470 were downregulated, indicating a pronounced differentiation in overall metabolic profiles between GXSM and LSM. Figure 6B and Table S1 present the statistical analysis of representative differential metabolites between GXSM and LSM. The results revealed marked abundance changes in multiple metabolites between the two groups, with some metabolites exhibiting relatively high fold changes. The predominantly upregulated compounds were Calendoflavoside, Methyl (9Z,14Z)-12,13,16-Trihydroxyoctadeca-9,14-Dienoate, and 3-(2-Furanylmethylene)-2,4-Pentanedione, while the predominantly downregulated compounds were 1-(2,6-Dihydroxy-4-Methoxyphenyl)-3-Phenyl-1-Propanone, 1-Deoxy-1-Morpholino-D-Fructose, 14-Hdohe, and 3-Phenylpropyl Acetate. Figure 6C,D and Table S2 showed the volcano plot and differential metabolite statistical chart for HNSM versus LSM, respectively. A total of 957 differential metabolites were identified, comprising 438 upregulated and 519 downregulated metabolites. The representative differential metabolites between HNSM and LSM included putatively annotated lipid-, carbohydrate-, and plant-derived compounds, such as Dgdg(O-16:3/2:0), 1-Deoxy-1-Morpholino-D-Fructose, Apigenin, and Pinolidoxin. These metabolites may reflect differences in raw materials, microbial activities, and fermentation-associated metabolic transformations between the two groups. However, because metabolite annotations were primarily based on database matching, the identities of individual compounds should be considered putative unless confirmed by authentic standards. As shown in Figure 6E, substantial metabolic differences were observed between GZSM and LSM, with a total of 947 differential metabolites identified, including 352 upregulated and 595 downregulated metabolites. Figure 6F and Table S3 further revealed that the predominantly upregulated metabolites were Inosinic Acid, Calendoflavoside, and Zapoterin, while the differential metabolites comprised multiple chemical classes, including nucleotide derivatives, phenolic compounds, lipid-related metabolites, and other putatively annotated features. Several metabolites, such as Inosinic Acid and Calendoflavoside, showed marked abundance differences between GZSM and LSM. Certain annotations with limited biological plausibility in fermented meat products were retained as tentative database matches and were not further interpreted. As shown in Figure 6G,H and Table S4, a total of 1160 differential metabolites were identified in YNSM relative to LSM, with 216 upregulated and 944 downregulated metabolites. The differential metabolites between YNSM and LSM encompassed multiple metabolite categories, including amino acids and their derivatives, organic acids, carbohydrates and their derivatives, and lipid-related compounds. Several putatively annotated metabolites, including Inosinic Acid, Pinolidoxin, 1-Deoxy-1-Morpholino-D-Fructose, and N6-Methyl-2′-Deoxyadenosine, exhibited substantial abundance differences between YNSM and LSM. These metabolites may contribute to the distinct metabolomic profiles observed among regions. However, annotations not confirmed by authentic standards should be interpreted with caution.

These results suggested that sour meat from different regions exhibited distinct metabolic remodeling patterns. Traditional sour meat was typically produced through spontaneous fermentation, during which the microbial community was influenced by multiple factors including raw material composition, salt concentration, temperature, fermentation duration, and regional environmental conditions. The dominant microorganisms and their metabolic capacities vary across regions, consequently leading to differential substrate conversion pathways for proteins, carbohydrates, and lipids, as well as differential accumulation of end products [26,27]. Therefore, the formation of differential metabolites likely resulted from the combined effects of region-specific microbial communities and fermentation environments [9]. Compared with laboratory-fermented sour meat, traditional sour meat samples from different regions exhibited distinct metabolomic patterns, among which selected differential metabolites with high Variable Importance in Projection (*VIP*) values may represent candidate metabolites associated with differences between regional traditional sour meat samples and laboratory-fermented sour meat.These results demonstrated that each traditional sour meat group exhibited substantial metabolomic differences relative to laboratory-fermented sour meat (LSM). Differential metabolites were screened using VIP > 1.0, |log2FC| > 1, and FDR-adjusted *p* < 0.05. The observed differences likely reflect variations in fermentation environments, microbial communities, raw materials, and processing practices among regions. However, because the present analyses compared each traditional sour meat group only with LSM, the identified metabolites should be interpreted as candidate discriminatory metabolites relative to the laboratory-fermented reference rather than definitive regional biomarkers distinguishing among the four traditional sour meat products. Additional pairwise comparisons among GXSM, HNSM, GZSM, and YNSM and validation using independent biological samples will be required to establish robust regional marker metabolites.

To further elucidate the metabolic differences between traditional sour meat from different regions and LSM, representative differential metabolites with high *VIP* values were selected for hierarchical clustering analysis, with results presented in Figure 7. As shown in Figure 7A, the differential metabolites between GXSM and LSM exhibited relatively consistent expression patterns, displaying pronounced reverse abundance trends between the two groups. Most metabolites showed lower abundances in GXSM and higher abundances in LSM, indicating a clear differentiation in the metabolomic profiles between GXSM and LSM [28]. Notably, several metabolites, including 8-Deoxy-11-Hydroxy-13-Chlorogrosheimin, (7′R,8′R)-4,7′-Epoxy-3′,5-Dimethoxy-4′,9,9′-Lignanetriol 9′-Glucoside, Pi(13:0/16:4), and Isoeruboside B, exhibited marked inter-group differences with VIP scores exceeding 2.0, suggesting that these compounds contributed substantially to the discrimination between GXSM and LSM. Figure 7B revealed distinct clustering patterns of differential metabolites between HNSM and LSM. Based on the row-wise Z-score normalized heatmap, most representative metabolites exhibited relatively higher abundances in LSM and lower abundances in HNSM. These results suggested substantial differences in metabolite composition between the two groups. The differential metabolites primarily included Pi(13:0/16:4), 8-Deoxy-11-Hydroxy-13-Chlorogrosheimin, 17-Hydroxy-15-Kauren-19-Oic Acid, Cyclohexylsulfamate, and Lyciumoside IV, among which certain metabolites exhibited VIP scores of 2.2–2.4 with high statistical significance, suggesting that amino acid derivatives and lipid-related metabolites might serve as important metabolic features distinguishing HNSM from LSM. As shown in Figure 7C, the top-ranked discriminatory features included signals tentatively annotated as 8-Deoxy-11-Hydroxy-13-Chlorogrosheimin, Meticillin, Sucrose, Tenatoprazole, and Inosinic Acid. Because non-targeted metabolite annotation was primarily based on accurate mass and MS/MS spectral matching, these identifications should be regarded as putative (MSI level 2) unless confirmed using authentic reference standards. Therefore, these features are discussed only as candidate discriminatory metabolites rather than definitive biomarkers. As shown in Figure 7D, the differential metabolites between YNSM and LSM primarily included 8-Deoxy-11-Hydroxy-13-Chlorogrosheimin, Inosinic Acid, Lyciumoside IV, Poncirin, and Sucrose, with VIP scores exceeding 2.3, demonstrating strong explanatory power for the metabolomic differences between YNSM and LSM [29].

Collectively, the high-VIP differential metabolites identified in sour meat from different regions mainly involved amino acids and their derivatives, lipids and fatty acid derivatives, nucleosides, organic acids, and plant-derived phenolic compounds. These findings suggested that processes including proteolysis and amino acid conversion, lipolysis and lipid oxidation, and carbohydrate metabolism might collectively contribute to the formation of the characteristic metabolomic profiles of sour meat from different geographical origins [30]. Several limitations should be acknowledged. First, metabolite identification was primarily based on database matching and therefore most annotations correspond to MSI level 2 confidence rather than confirmation with authentic standards. Second, the comparisons were performed against laboratory-fermented sour meat (LSM), which limits the ability to identify definitive geographical marker metabolites among traditional sour meat products. Third, no direct quality measurements, such as sensory evaluation, targeted taste compounds, pH, salt content, or water activity, were included. Therefore, potential links between differential metabolites and flavor or quality attributes should be regarded as hypotheses requiring further validation.

### 3.7. KEGG Pathway Enrichment and Topology Analysis of Differential Metabolites

To further elucidate the potential biological processes associated with the differential metabolites between traditional sour meat from different regions and LSM, KEGG pathway enrichment and topology analyses were performed on the differential metabolites of GXSM, HNSM, GZSM and YNSM compared with LSM, with results presented in Figure 8. As shown in Figure 8A, the differential metabolites between GXSM and LSM were mainly enriched in multiple pathways, including Phenylalanine metabolism, Alcoholism, Cholesterol metabolism, cGMP-PKG signaling pathway, and Steroid hormone biosynthesis. KEGG topology analysis (Figure 8B) further revealed marked differences in the topological impact of the pathways involved in GXSM versus LSM. Among these, retinol metabolism exhibited the highest Impact value (0.26) with considerable statistical significance. Additionally, starch and sucrose metabolism, Toluene degradation, Betalain biosynthesis, and alpha-linolenic acid metabolism also showed relatively high Impact values. Combining *p*-values and Impact values, retinol metabolism emerged as a key pathway with substantial potential contribution to the metabolic differences between GXSM and LSM, while starch and sucrose metabolism and alpha-linolenic acid metabolism may be associated with carbon source utilization and lipid metabolism alterations. These results suggested that the metabolic differences between GXSM and LSM were not confined to a single metabolic process, but rather involve integrated changes across amino acids, lipids, nucleotides, carbohydrates, and other compound classes [31]. Similarly, as shown in Figure 8C,D, the differential metabolites between HNSM and LSM were mapped to multiple KEGG pathways. Although several disease-related pathways, such as Parkinson disease, and signaling pathways, such as the cAMP signaling pathway, were significantly enriched, these annotations likely resulted from the participation of shared metabolites in multiple KEGG categories and should therefore be interpreted with caution. In the context of fermented meat, pathways directly related to microbial metabolism and substrate conversion are more biologically meaningful. Accordingly, aminobenzoate degradation, retinol metabolism, starch and sucrose metabolism, and secondary metabolite biosynthesis were considered the major metabolic pathways associated with the observed compositional differences between HNSM and LSM. KEGG topology analysis further highlighted aminobenzoate degradation, retinol metabolism, and starch and sucrose metabolism as the most influential pathways. As shown in Figure 8E,F, the differential metabolites between GZSM and LSM were primarily enriched in pathways such as Axon regeneration, Citrate cycle (TCA cycle), Tryptophan metabolism, Asthma, and Bile secretion. KEGG topology analysis between GZSM and LSM identified Teichoic acid biosynthesis, Linoleic acid metabolism, and Tryptophan metabolism as key metabolic pathways. Teichoic acid biosynthesis exhibited a relatively high pathway impact value, indicating that the matched metabolites occupied topologically important positions within the pathway network. However, the enrichment *p*-value did not reach the significance threshold, suggesting that this pathway should be interpreted with caution. As shown in Figure 8G,H, the differential metabolites between YNSM and LSM were mainly enriched in pathways including biosynthesis of phenylpropanoids, ethylbenzene degradation, caprolactam degradation, synaptic vesicle cycle, and bile secretion. KEGG topology analysis between YNSM and LSM identified teichoic acid biosynthesis, flavone and flavonol biosynthesis, and alanine, aspartate, and glutamate metabolism as key metabolic pathways. Collectively, substantial differences were observed in both microbial community composition and metabolomic profiles among sour meat samples from different geographical regions. These parallel patterns suggest that regional fermentation environments, processing practices, and microbial communities may all contribute to the observed metabolomic variation. However, because direct microbe–metabolite association analyses and metabolic flux measurements were not performed in the present study, the relationships between specific microbial taxa and metabolite accumulation remain speculative and require further investigation [10].

### 3.8. Discussion

The present study demonstrated pronounced regional variations in both microbial communities and metabolomic profiles among traditional sour meat samples collected from different areas of China. Similarly to previous studies on fermented meat products, members of the phylum Firmicutes, particularly lactic acid bacteria, represented the dominant microbial group in most samples, reflecting their important role in carbohydrate fermentation, acid production, and flavor development during sour meat fermentation. Previous investigations of traditional fermented meat products from Southwest China have likewise reported the predominance of Lactobacillus, Weissella, and related lactic acid bacteria, which contribute to product preservation and sensory quality through organic acid production and competitive exclusion of undesirable microorganisms.

At the genus level, substantial regional differences were observed. GXSM and HNSM were characterized by high abundances of Lactobacillus, whereas YNSM showed greater enrichment of Weissella and Staphylococcus. These differences may be associated with variations in raw materials, fermentation environments, salt concentrations, fermentation duration, and indigenous microbial resources. Previous studies have shown that spontaneous fermentation processes are strongly influenced by local environmental microbiota and traditional manufacturing practices, resulting in distinct microbial succession patterns and regional microbial signatures.

Metabolomic analysis further revealed significant regional differentiation in metabolite composition. Differential metabolites were mainly involved in amino acid metabolism, lipid metabolism, carbohydrate metabolism, nucleoside metabolism, and plant-derived secondary metabolite pathways. These findings are consistent with previous reports demonstrating that proteolysis, lipolysis, and carbohydrate conversion represent major biochemical processes shaping the flavor and quality characteristics of fermented meat products. The enrichment of pathways related to amino acid metabolism may contribute to the generation of flavor precursors, whereas lipid metabolic pathways may be associated with the formation of volatile aroma compounds and oxidative products.

Fermentation practices may also represent important confounding factors contributing to the observed regional differences. Traditional sour meat production varies considerably among regions with respect to meat type, seasoning ingredients, fermentation containers, temperature, humidity, fermentation duration, and starter usage. Such variations can influence both microbial succession and metabolic activity, thereby affecting the final metabolomic characteristics. Therefore, the regional differences observed in this study likely reflect the combined effects of geographical origin, environmental microbiota, and processing practices rather than geographical location alone.

Although the integrated microbiome–metabolome approach provided valuable insights into regional characteristics of sour meat, several limitations should be acknowledged. First, the cross-sectional sampling design cannot establish causal relationships between microbial taxa and metabolite accumulation. Second, detailed production parameters were not completely standardized among traditional products collected from different regions, which may have contributed to variation in both microbial and metabolite profiles. Third, direct correlation analyses between microbial taxa and metabolites were not performed in the present study. Therefore, the proposed links between microbial community composition and metabolite accumulation should be interpreted as potential associations rather than direct evidence of microbial metabolic functions. Future studies incorporating larger sample sizes, controlled fermentation experiments, and multi-omics correlation analyses will be necessary to clarify the mechanistic relationships between microbial communities and metabolite formation during sour meat fermentation.

## 4. Conclusions

In conclusion, significant regional differences in both microbial communities and metabolomic profiles were observed among traditional sour meat samples from different regions of China. Distinct dominant microbial taxa and metabolite compositions contributed to region-specific biological characteristics, while amino acid, lipid, and carbohydrate metabolism represented the major pathways underlying metabolic differentiation. These findings provide valuable insights into the regional characteristics of traditional Chinese sour meat and offer a scientific basis for microbial resource development, quality evaluation, and future standardization of fermented meat products.

## Figures and Tables

**Figure 1:**
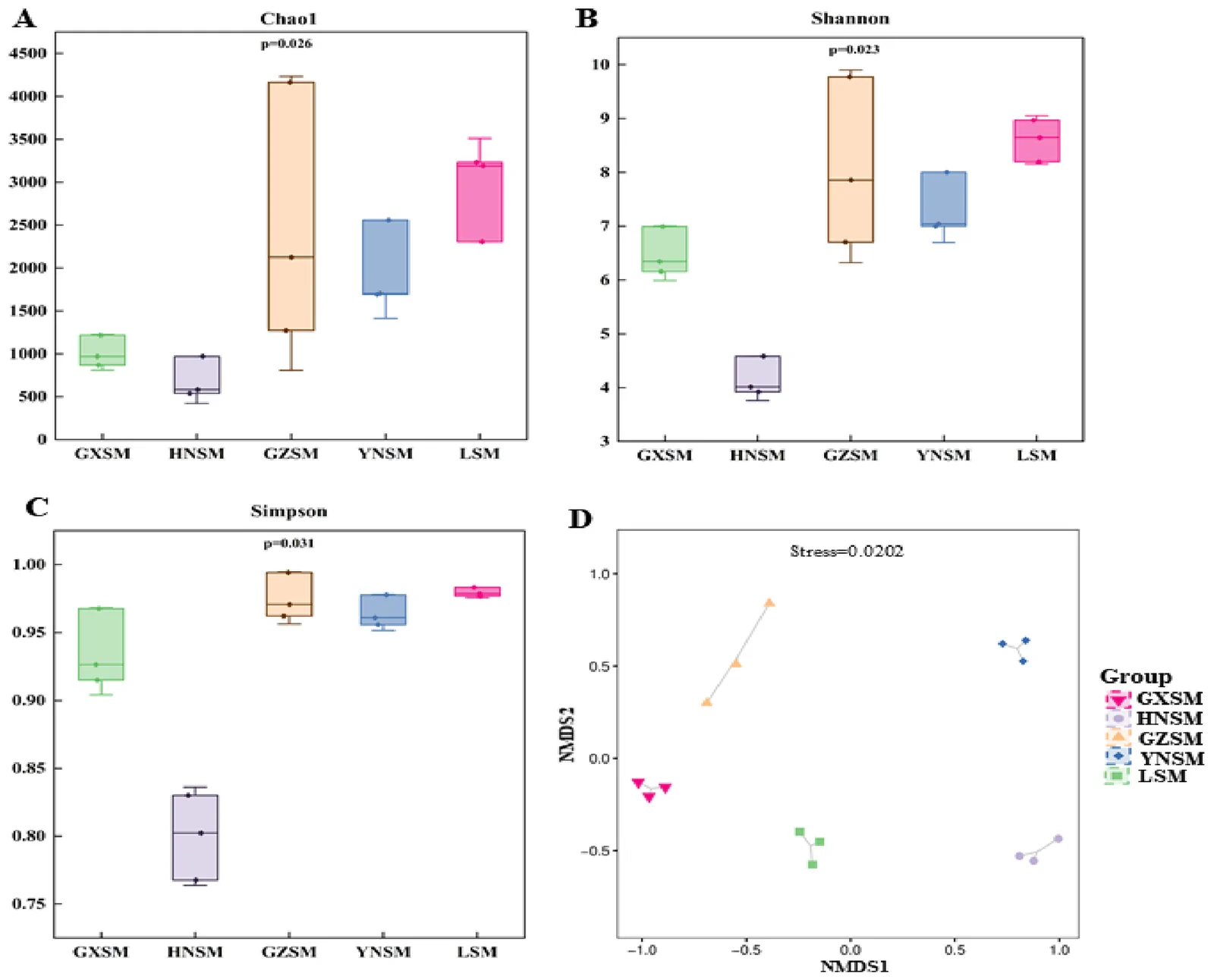
Alpha and beta diversity analyses of microbial communities in sour meat samples from different geographical origins. (**A**), Chao1 index; (**B**), Shannon index; (**C**), Simpson index; (**D**), NMDS analysis. Wherein, GXSM, HNSM, GZSM, YNSM, and LSM represent sour meat samples from Guangxi, Hunan, Guizhou, Yunnan, and laboratory sour meat, respectively.

**Figure 2:**
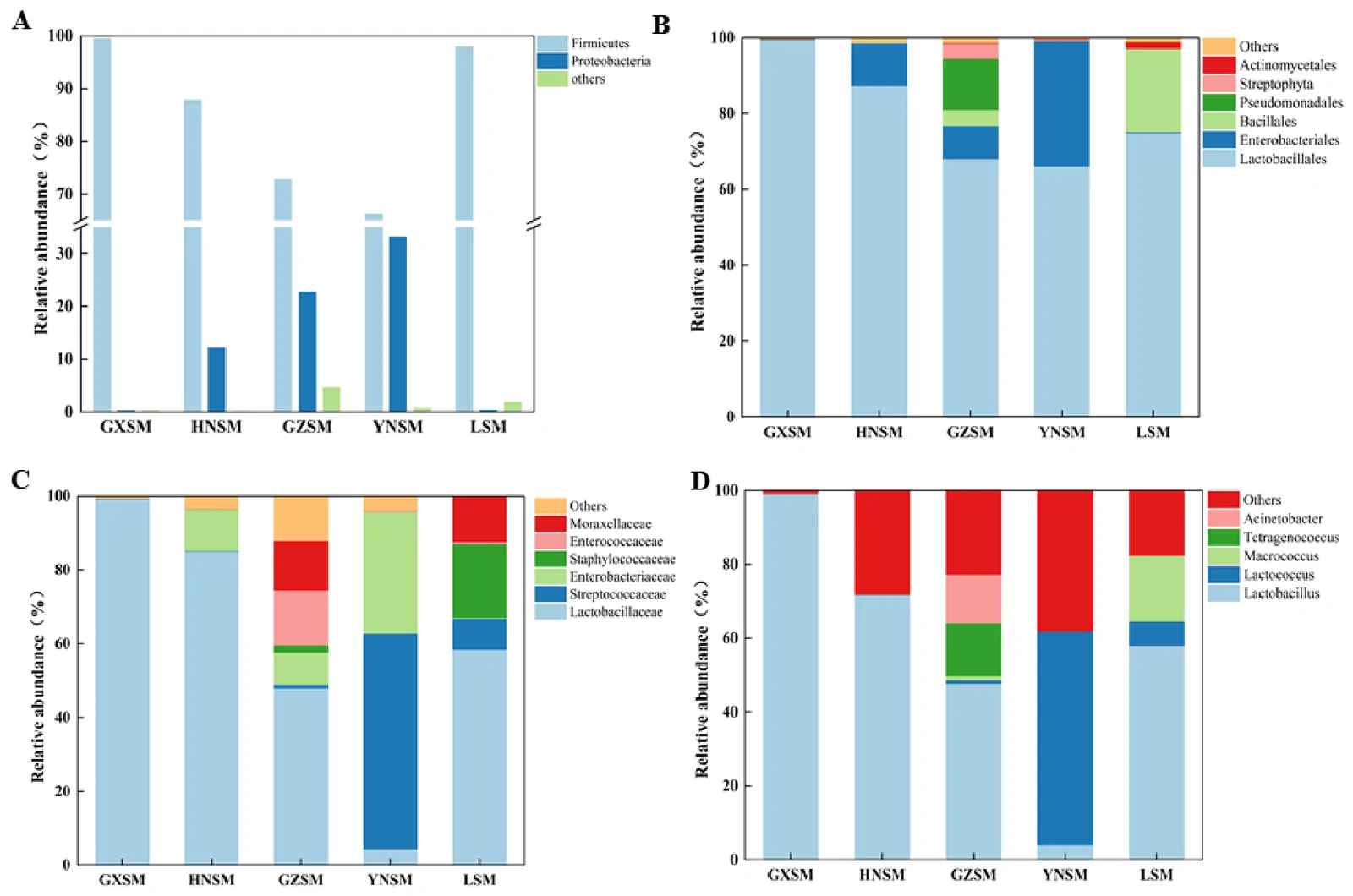
Microbial community composition of sour meat samples from different geographical origins at the phylum, order, family, and genus levels. (**A**), Phylum level; (**B**), Order level; (**C**), Family level; (**D**), Genus level. Wherein, GXSM, HNSM, GZSM, YNSM, and LSM represent sour meat samples from Guangxi, Hunan, Guizhou, Yunnan, and laboratory sour meat, respectively.

**Figure 3:**
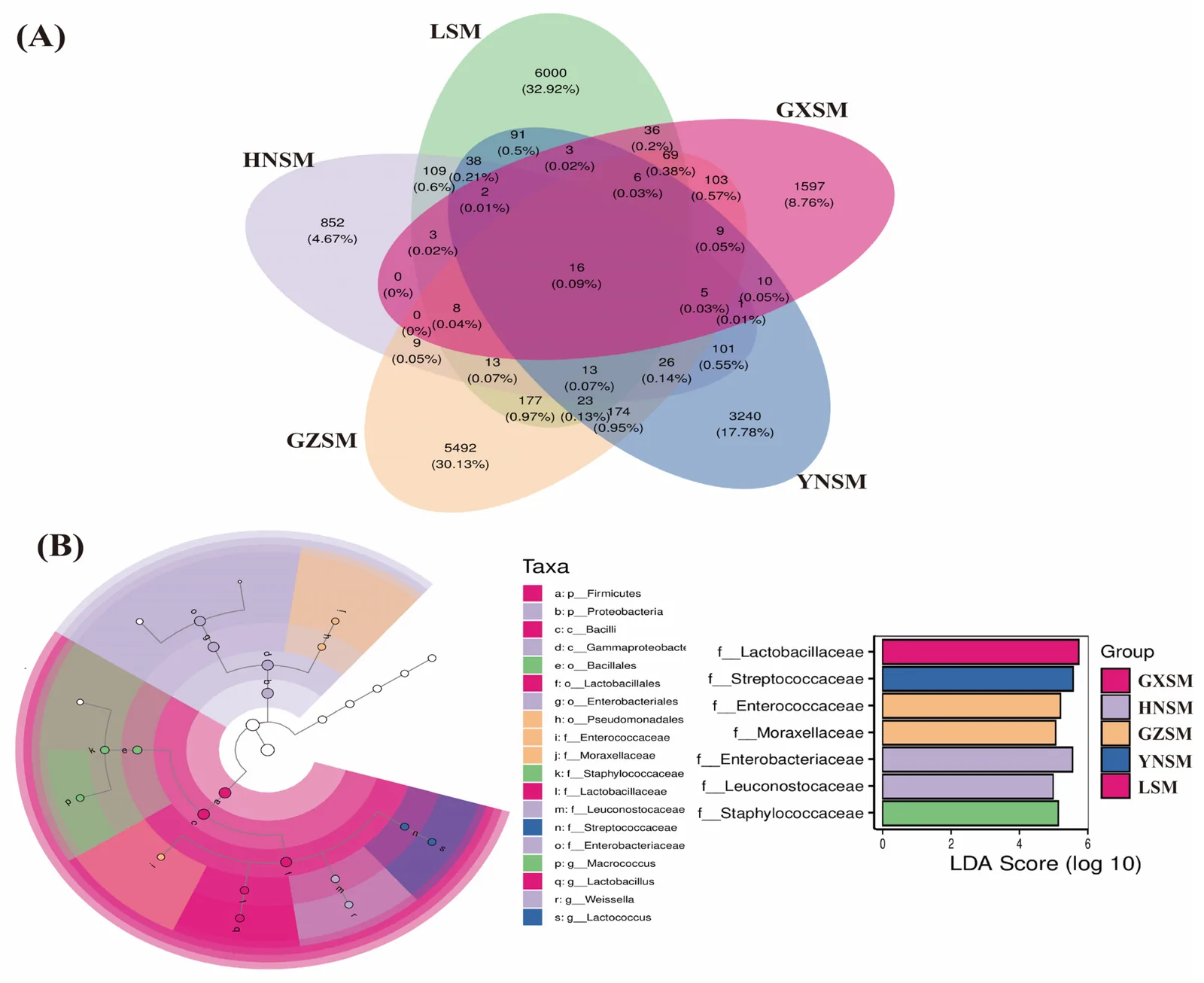
Unique and shared OTUs, species distribution, and LEfSe-based differential species analysis of microbial communities in sour meat samples from different geographical origins. (**A**) Venn diagram at the OTU level; (**B**) LEfSe differential species analysis, with the cladogram on the left and the LDA score bar plot on the right. The cladogram illustrated the hierarchical evolutionary relationships from phylum to genus, with different colors representing taxa enriched in each corresponding group. Wherein, GXSM, HNSM, GZSM, YNSM, and LSM represented sour meat samples from Guangxi, Hunan, Guizhou, Yunnan, and laboratory sour meat, respectively.

**Figure 4:**
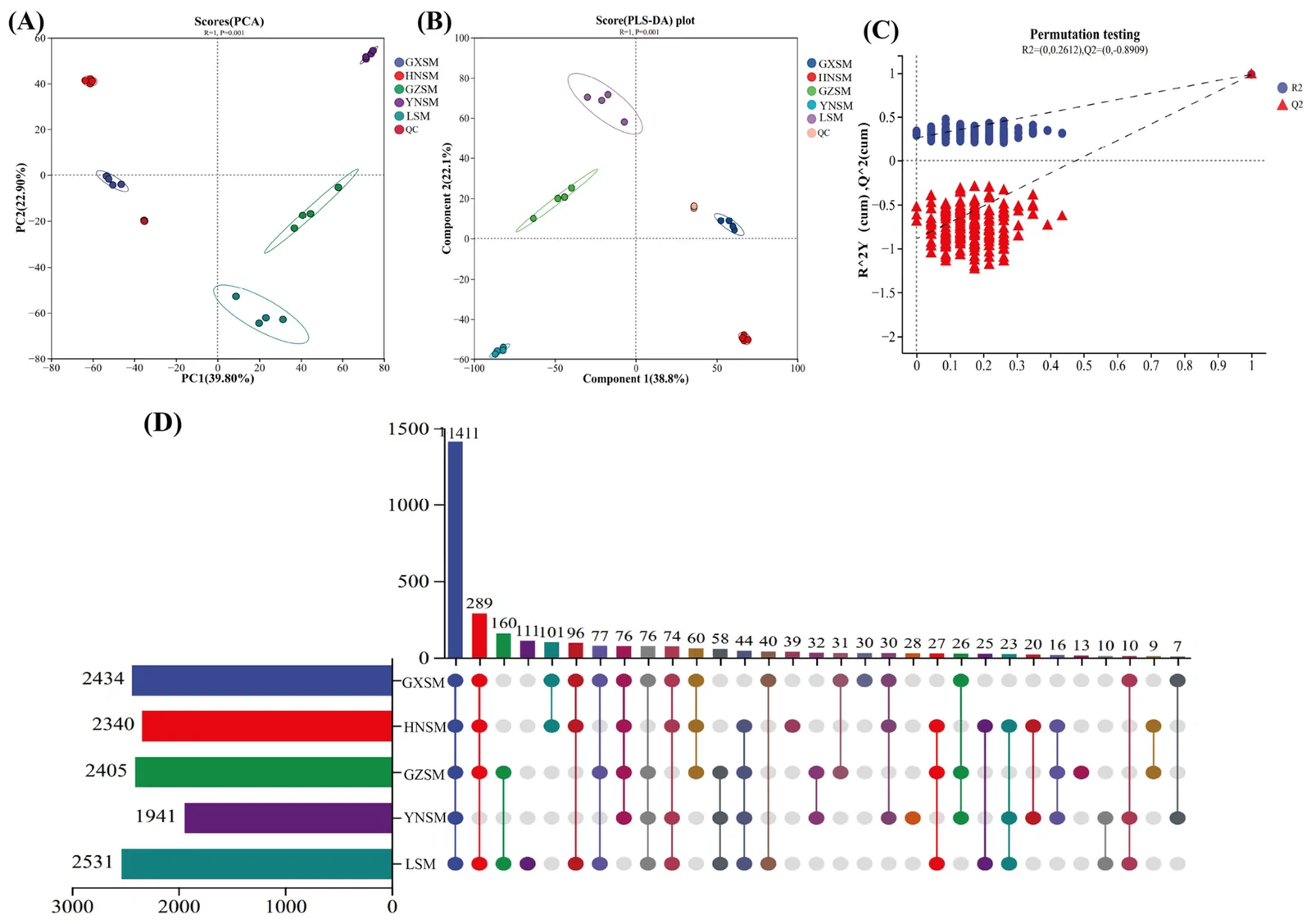
Multivariate statistical analysis of metabolites in fermented sour meat from different geographical origins. (**A**), PCA score plot of metabolites in fermented sour meat from different regions; (**B**), PLS-DA score plot of metabolites in fermented sour meat from different regions; (**C**), Permutation validation of the PLS-DA model for metabolites in fermented sour meat from different regions; (**D**), UpSet plot showing the shared and unique metabolites among fermented sour meat samples from different regions. Wherein, GXSM, HNSM, GZSM, YNSM, and LSM represented samples from Guangxi, Hunan, Guizhou, Yunnan, and laboratory sour meat, respectively.

**Figure 5:**
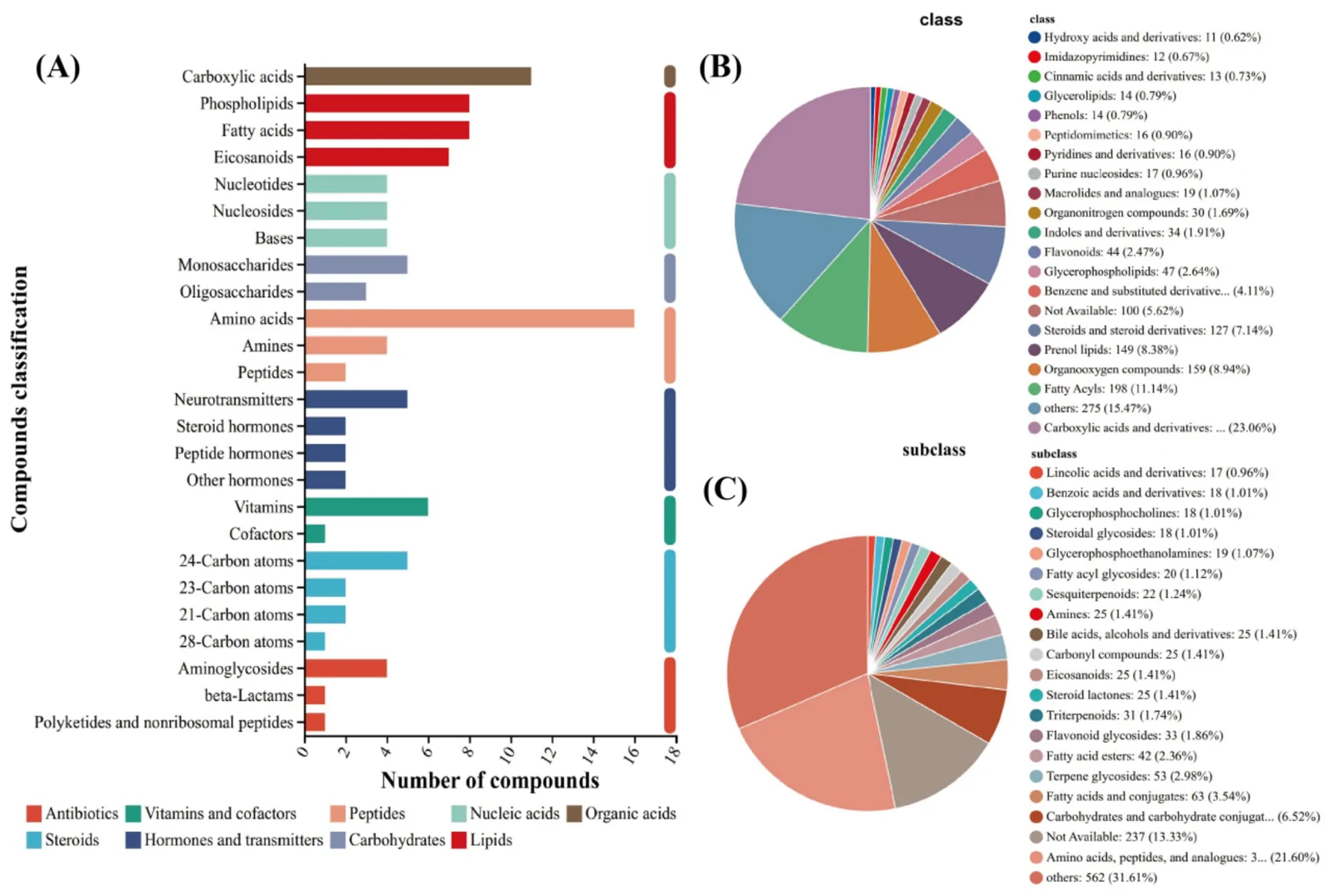
Metabolite profiling of fermented sour meat from different geographical origins. (**A**), KEGG chemical classification of metabolites; (**B**), Distribution of metabolite counts based on HMDB secondary classification; (**C**), Distribution of metabolite counts based on HMDB tertiary classification. Wherein, GXSM, HNSM, YNSM, GZSM, and LSM represent sour meat samples from Guangxi, Hunan, Guizhou, Yunnan, and laboratory sour meat, respectively.

**Figure 6:**
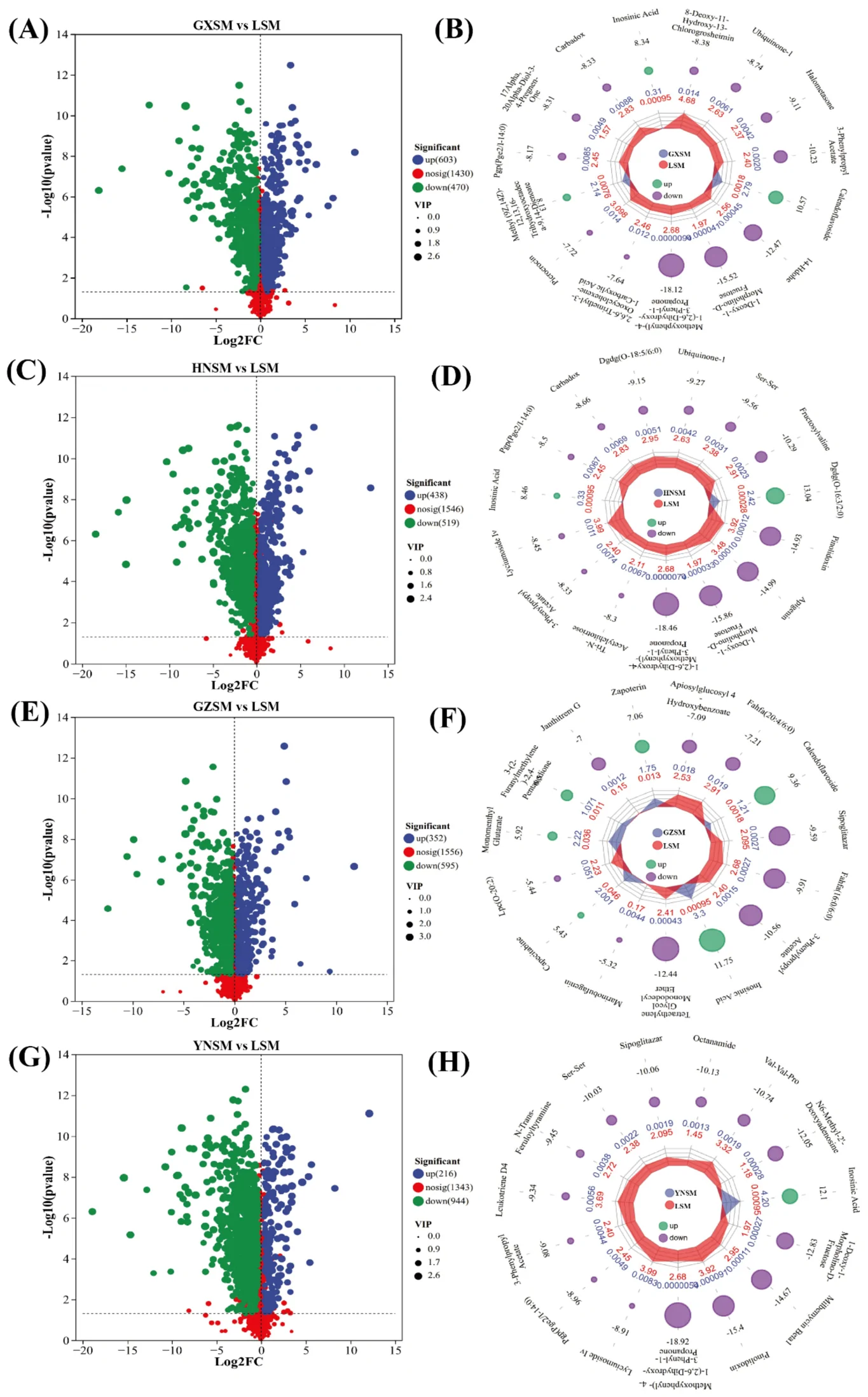
Differential metabolite analysis of fermented sour meat from different geographical origins. (**A**,**B**) Volcano plot and statistical chart of differential metabolites between GXSM and LSM, respectively; (**C**,**D**), Volcano plot and statistical chart of differential metabolites between HNSM and LSM, respectively; (**E**,**F**), Volcano plot and statistical chart of differential metabolites between GZSM and LSM, respectively; (**G**,**H**), Volcano plot and statistical chart of differential metabolites between YNSM and LSM, respectively. Wherein, GXSM, HNSM, GZSM, YNSM, and LSM represent sour meat samples from Guangxi, Hunan, Guizhou, Yunnan, and laboratory sour meat, respectively.

**Figure 7:**
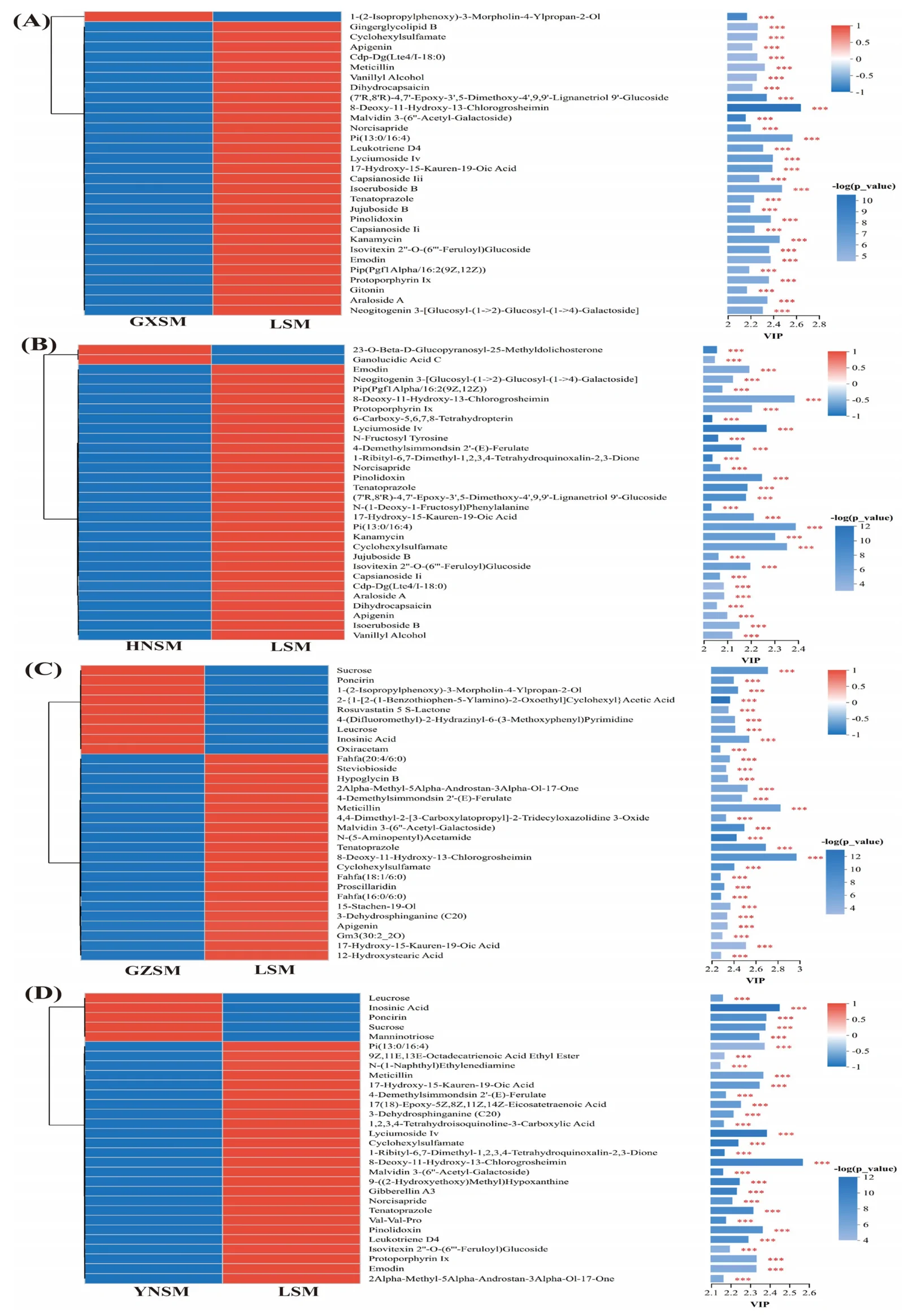
Hierarchical clustering heatmap analysis of differential metabolites between sour meat from different geographical origins and LSM. Heatmaps were generated using row-wise Z-score normalization of metabolite abundances. Red and blue colors represent relatively higher and lower abundances, respectively, within each metabolite across samples. *** stands for *p* < 0.001, with a significant difference. (**A**) GXSM vs. LSM; (**B**) HNSM vs. LSM; (C) GZSM vs. LSM; (D) YNSM vs. LSM. Wherein, GXSM, HNSM, GZSM, YNSM, and LSM represent sour meat samples from Guangxi, Hunan, Guizhou, Yunnan, and laboratory sour meat, respectively.

**Figure 8:**
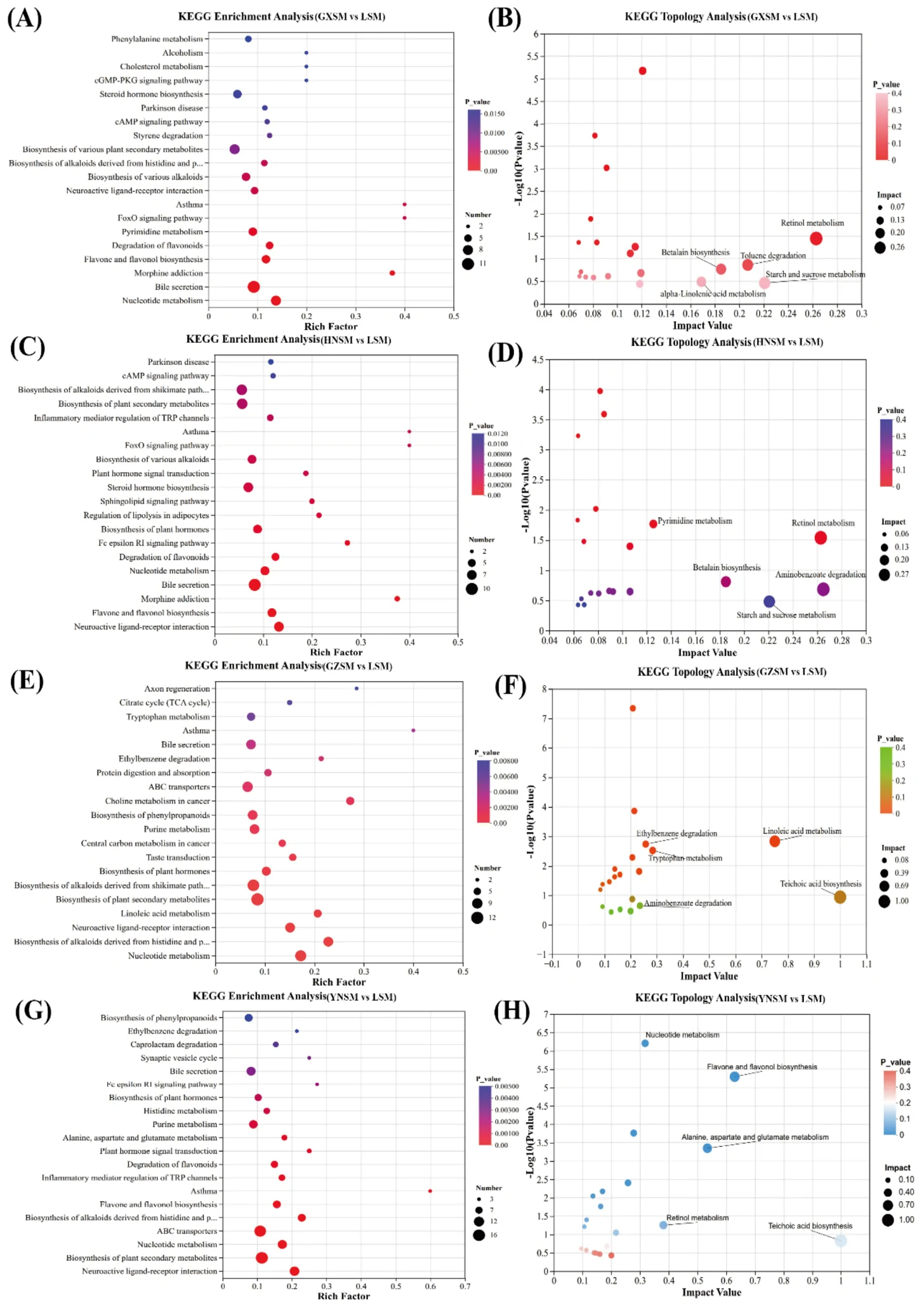
KEGG pathway enrichment and topological analysis of differential metabolites. (**A**,**B**), KEGG enrichment analysis diagram and KEGG topological analysis bubble diagram of GXSM vs. LSM, respectively; (**C**,**D**), KEGG enrichment analysis diagram and KEGG topological analysis bubble diagram of HNSM vs. LSM, respectively; (**E**,**F**), KEGG enrichment analysis diagram and KEGG topological analysis bubble diagram of GZSM vs. LSM, respectively. (**G**,**H**), KEGG enrichment analysis diagram and KEGG topological analysis bubble diagram of YNSM vs. LSM, respectively.

## Data Availability

The datasets generated and analyzed during this study include 16S rRNA gene sequencing data and untargeted metabolomics data. Raw sequencing reads, metabolomics raw data, processed feature tables, metabolite annotation tables (including retention time, precursor m/z, adduct information, mass error, database matches, and annotation confidence levels), and sample metadata are available through public repositories (accession numbers to be provided upon acceptance). Detailed bioinformatics and metabolomics analysis workflows are described in the Methods section and Supplementary Materials. Additional data supporting the findings of this study are available from the corresponding author upon reasonable request.

## Supplementary Materials

The following supporting information can be downloaded at: https://www.mdpi.com/article/10.3390/foods15193424/s1. Table S1. Statistics of Differential Metabolites between GXSM and LSM. Table S2. Statistics of Differential Metabolites between HNSM and LSM. Table S3. Statistics of Differential Metabolites between GZSM and LSM. Table S4. Statistics of Differential Metabolites between YNSM and LSM.
