# Supplementary material for: Comparative Analysis of Microbial Community and Metabonomics in Traditional Sour Meat from Different Regions of China
Source: Foods. 2026 Sep 24;15(19):3424. doi: 10.3390/foods15193424 (PMC13664529; doi:10.3390/foods15193424)
Supplement: Supplementary file 1 [file foods-15-03424-s001.zip › foods-4536729-supplementary.pdf]

Comparative analysis of microbial community and metabonomics in traditional sour meat from different regions of China

Qing Guo[ab], Shijie Liu[ab], Qian Ding[ab], Miaoyun Li[ab*]，Dong Liang[ab], Lijun Zhao[ab]，Yanxia Liu[ab]

a College of Food Science and Technology, Henan Agricultural University, Zhengzhou, 450002, P.R. China

b International Joint Laboratory of Meat Processing and Safety in Henan Province, Henan Agricultural University, Zhengzhou 450002, China

* Corresponding author. E-mail: limy7476@126.com (Miaoyun Li)

Table S1 Statistics of Differential Metabolites between GXSM and LSM

Metabolite	P_value	FDR	VIP_PLS-DA	FC(A/E)	Log2FC(A/E)	Regulate	level	Library ID	M/Z	Retention time
8-Deoxy-11-Hydroxy-13-Chlorogrosheimin	3.383E-11	1.675E-8	2.6178	0.003	-8.3774	down	LevelBii	HMDB0041037	357.114	2.8403
Pi(13:0/16:4)	1.456E-7	3.461E-6	2.5336	0.0503	-4.3121	down	LevelBi	-	805.4202	6.4339
Isoeruboside B	5.324E-7	9.091E-6	2.438	0.1249	-3.0016	down	LevelBii	HMDB0029769	1125.5302	7.044
Kanamycin	2.19E-7	4.646E-6	2.4254	0.0198	-5.6591	down	LevelBii	HMDB0015303	967.4702	5.7845
Lyciumoside Iv	1.122E-7	2.851E-6	2.3684	0.0356	-4.8136	down	LevelBii	HMDB0033499	821.415	5.462
17-Hydroxy-15-Kauren-19-Oic Acid	8.112E-8	2.219E-6	2.3609	0.0146	-6.0982	down	LevelBi	-	301.2143	5.8592
Pinolidoxin	3.148E-7	6.036E-6	2.3417	0.0344	-4.8627	down	LevelBii	HMDB0301836	359.1483	4.0277
Emodin	2.768E-6	3.124E-5	2.3419	0.0215	-5.5412	down	LevelBi	HMDB0035214;LMPK13040008	269.0449	5.2503
Isovitexin 2''-O-(6'''-Feruloyl)Glucoside	2.553E-7	5.174E-6	2.3314	0.006	-7.3735	down	LevelBii	HMDB0038042	769.1958	4.139
Protoporphyrin Ix	1.566E-6	2.038E-5	2.3271	0.0603	-4.0512	down	LevelBii	HMDB0000241	1123.5138	6.8225
Araloside A	1.033E-6	1.502E-5	2.3143	0.007	-7.1626	down	LevelBii	HMDB0034535	961.4617	6.9054
(7'R,8'R)-4,7'-Epoxy-3',5-Dimethoxy-4',9,9'-Lignanetriol 9'-Glucoside	4.513E-9	2.959E-7	2.3203	0.0091	-6.7819	down	LevelBii	HMDB0038711	521.2002	3.4425
Meticillin	1.425E-5	8.14E-5	2.3109	0.1691	-2.5638	down	LevelBii	HMDB0015541	379.0959	2.8775
Leukotriene D4	1.602E-7	3.687E-6	2.277	0.0304	-5.0412	down	LevelBii	HMDB0003080	541.2626	5.2412
Neogitogenin 3-[Glucosyl-(1->2)-Glucosyl-(1->4)-Galactoside]	1.041E-6	1.512E-5	2.2738	0.1814	-2.4628	down	LevelBii	HMDB0041134	963.4787	7.1181
Capsianoside Iii	4.455E-7	7.861E-6	2.2589	0.1519	-2.7185	down	LevelBii	HMDB0031891	1145.521	4.657
Gingerglycolipid B	9.098E-6	5.567E-5	2.2235	0.0858	-3.5425	down	LevelBii	HMDB0041094	659.3616	6.4618
Cdp-Dg(Lte4/I-18:0)	1.623E-5	9.046E-5	2.2204	0.1283	-2.9621	down	LevelBii	HMDB0293917	1145.5212	5.1303
Cyclohexylsulfamate	7.848E-6	4.926E-5	2.2557	0.3585	-1.48	down	LevelBi	HMDB0031340	178.0541	2.8592
Vanillyl Alcohol	1.274E-5	7.412E-5	2.2176	0.0995	-3.3298	down	LevelBi	HMDB0032012	137.0591	6.3883
Capsianoside Ii	3.323E-7	6.28E-6	2.2047	0.1377	-2.8602	down	LevelBii	HMDB0031892	1085.5305	5.4492
Tenatoprazole	5.854E-7	9.707E-6	2.2135	0.1448	-2.7879	down	LevelBii	HMDB0258812	737.2202	2.8683
Dihydrocapsaicin	1.29E-5	7.487E-5	2.1841	0.1933	-2.3709	down	LevelBi	HMDB0038457;LMFA08020318	308.2215	6.7388
Apigenin	2.14E-5	0.0001137	2.1837	0.037	-4.7574	down	LevelBi	HMDB0002124;LMPK12110005	271.0596	5.2537
Norcisapride	3.141E-8	1.128E-6	2.176	0.1511	-2.7262	down	LevelBii	HMDB0061018	671.2319	3.9904
Jujuboside B	5.71E-7	9.545E-6	2.1656	0.1548	-2.6911	down	LevelBii	HMDB0035745	1079.5244	7.5737
Pip(Pgf1Alpha/16:2(9Z,12Z))	2.132E-6	2.593E-5	2.1589	0.1367	-2.8705	down	LevelBii	HMDB0278720	1031.466	7.1273
1-(2-Isopropylphenoxy)-3-Morpholin-4-Ylpropan-2-Ol	2.801E-9	2.222E-7	2.1485	40.0446	5.3235	up	LevelBi	-	280.1891	3.912
Gitonin	9.928E-7	1.465E-5	2.1373	0.1809	-2.4669	down	LevelBii	HMDB0301779	1109.5349	7.5177
Malvidin 3-(6''-Acetyl-Galactoside)	7.754E-10	9.846E-8	2.1404	0.1418	-2.8185	down	LevelBii	HMDB0301662	580.1383	2.8865
Janthitrem G	1.188E-6	1.664E-5	2.132	20.8624	4.3828	up	LevelBii	HMDB0030531	594.36	3.2123
Pc(O-22:4)	2.232E-6	2.674E-5	2.1437	5.4151	2.437	up	LevelBi	-	594.3515	8.144
Picrocrocin	3.204E-8	1.139E-6	2.1153	0.0047	-7.7194	down	LevelBii	HMDB0005796	705.3302	5.7015
N-Fructosyl Phenylalanine	2.101E-11	1.398E-8	2.1182	0.2093	-2.2566	down	LevelBi	-	328.138	2.1616
4-Demethylsimmondsin 2'-(E)-Ferulate	9.557E-8	2.518E-6	2.1096	0.2065	-2.2754	down	LevelBii	HMDB0034773	558.1572	2.8683
Fisetin	1.211E-5	7.099E-5	2.0959	0.0242	-5.3696	down	LevelBi	LMPK12111566	285.0393	4.8996
Capsaicin	1.585E-5	8.876E-5	2.0888	0.3361	-1.5728	down	LevelBi	HMDB0002227;HMDB0302729;LMFA08020085	306.2059	6.3883
N-Fructosyl Tyrosine	7.941E-7	1.229E-5	2.0788	0.1024	-3.2884	down	LevelBi	-	344.1321	1.8729
Gm3(30:2_20)	4.84E-5	0.0002233	2.0621	0.1559	-2.6812	down	LevelBi	-	1139.6317	7.0625
Cinncassiol D4 2-Glucoside	5.397E-8	1.613E-6	2.0739	0.1849	-2.4354	down	LevelBii	HMDB0035065	559.2727	4.8811
Fumonisin A1	3.936E-5	0.0001878	2.0514	0.0981	-3.3497	down	LevelBii	HMDB0034713	822.4102	6.4339
Lys-Pro-Ile	7.199E-5	0.0003104	2.063	10.2757	3.3612	up	LevelBi	-	357.2464	2.3901
2-{[2-(1H-Indol-3-Yl)Ethyl]Ethanimidoyl}Phenol	7.248E-9	3.964E-7	2.0668	22.8721	4.5155	up	LevelBi	-	333.1545	2.4099
1-Ribityl-6,7-Dimethyl-1,2,3,4-Tetrahydroquinoxalin-2,3-Dione	2.1E-5	0.0001121	2.0658	0.1099	-3.1864	down	LevelBi	-	325.1378	3.133
Dgdg(O-18:5/6:0)	1.165E-5	6.874E-5	2.0457	0.0056	-7.4719	down	LevelBi	-	801.4241	7.3685
Tangeraxanthin	5.047E-6	3.847E-5	2.0439	12.0287	3.5884	up	LevelBii	HMDB0039015	505.3056	8.3635
Chenodeoxycholyltryptophan	0.0001723	0.0006264	2.0411	0.2119	-2.2388	down	LevelBii	HMDB0242395	633.3839	6.3883
Trans-3'-Hydroxycotinine O-Beta-D-Glucuronide	4.848E-8	1.513E-6	2.0335	0.0771	-3.6975	down	LevelBii	HMDB0341403	427.1347	4.0277
6-Carboxy-5,6,7,8-Tetrahydropterin	3.044E-6	3.35E-5	2.0279	0.225	-2.1519	down	LevelBii	HMDB0060410	246.0396	2.9046
Loquatifolin A	3.795E-6	3.847E-5	2.0261	0.2091	-2.2578	down	LevelBii	HMDB0030434	821.4139	6.0435
Carbadox	1.717E-7	3.89E-6	2.0211	0.0031	-8.3291	down	LevelBii	HMDB0031762	307.0707	4.7218
S-(Formylmethyl)Glutathione	3.258	6.684	2.022	0.190	-2.391	down	Leve	HMDB0060507	314.0	1.6103

Metabolite	P_value	FDR	VIP_PLS-DA	FC(A/E)	Log2FC(A/E)	Regulate	level	Library ID	M/Z	Retention time
	E-12	E-9	3	6	4		lBii		829	
Calendoflavoside	6.671E-9	3.742E-7	2.0201	1517.0917	10.5671	up	LevelBii	HMDB0037745	623.16	3.9158
Monomenthyl Glutarate	2.624E-8	9.915E-7	2.0166	77.264	6.2717	up	LevelBii	HMDB0303264	334.1971	4.2074
11Beta-Hydroxytestosterone	5.421E-8	1.615E-6	2.0146	0.0969	-3.3678	down	LevelBii	HMDB0012533	349.1997	5.8494
Alpha-Tocopheryloxyacetic Acid	9.005E-8	2.417E-6	2.0143	0.055	-4.1845	down	LevelBii	HMDB0248242	506.4175	7.7057
Carmine Red	1.488E-7	3.509E-6	2.0031	0.0366	-4.7706	down	LevelBii	HMDB0030658	513.0674	4.2781
Pe(O-16:4/6:0)	2.458E-7	5.056E-6	1.9988	4.5007	2.1701	up	LevelBi	-	1057.6236	8.2071
2Alpha-Methyl-5Alpha-Androstan-3Alpha-Ol-17-One	4.742E-5	0.0002197	1.9966	0.19	-2.3959	down	LevelBi	-	269.2252	6.4371
(3Beta,5Alpha,6Beta,9Alpha,22E,24R)-23-Methylergosta-7,22-Diene-3,5,6,9-Tetrol	1.644E-5	9.152E-5	1.9921	3.5804	1.8401	up	LevelBii	HMDB0033634	499.3203	2.2409
Milbemycin Beta1	2.105E-5	0.0001122	1.9785	0.0657	-3.9291	down	LevelBii	HMDB0254727	608.3514	6.7388
(3Ar,4S,7S,7As)-3A,4,5,6,7,7A-Hexahydro-1H-4,7-Methanoinden-6-Yl Propionate	2.288E-6	2.724E-5	1.9748	0.0649	-3.9459	down	LevelBi	-	207.1372	5.8592
Proscillaridin	1.693E-8	7.107E-7	1.9781	0.0945	-3.4042	down	LevelBii	HMDB0256851	565.2597	5.8957
Lpc(19:1)	6.755E-6	4.333E-5	1.9737	5.4378	2.443	up	LevelBi	LMGP01050130	580.3593	8.309
Hypoglycin B	1.111E-6	1.589E-5	1.9712	0.187	-2.419	down	LevelBii	HMDB0029428	309.0856	4.7167
1-(2,6-Dihydroxy-4-Methoxyphenyl)-3-Phenyl-1-Propanone	4.966E-7	8.645E-6	1.97	0	-18.1201	down	LevelBii	HMDB0034435	331.1169	4.0648
Dlcl(16:4/22:7)	5.799E-8	1.703E-6	1.9682	0.0374	-4.7422	down	LevelBi	-	983.4332	4.8351
Steviobioside	8.08E-6	5.041E-5	1.9541	0.1052	-3.2494	down	LevelBi	HMDB0036707	641.3133	6.0435
Pyridoxine	1.347E-7	3.225E-6	1.9661	0.2546	-1.9739	down	LevelBi	HMDB0000239;PW_C000161	170.0797	1.4959
Kentsin	2.399E-5	0.0001252	1.9516	7.3946	2.8865	up	LevelBii	HMDB0005776	564.3258	6.2803
Temsirolimus	6.995E-9	3.863E-7	1.9629	3.4336	1.7797	up	LevelBii	HMDB0015632	1047.6327	7.7929
Ubiquinone-1	2.342E-7	4.865E-6	1.9511	0.0023	-8.7384	down	LevelBii	HMDB0002012	314.1369	4.4039
Dehydroisoandrosterone 3-Glucuronide	2.899E-9	2.259E-7	1.9529	0.1302	-2.9407	down	LevelBii	HMDB0010327	497.2706	4.8829
N-Eicosapentaenoyl Aspartic Acid	1.777E-5	9.735E-5	1.9482	0.067	-3.8996	down	LevelBii	HMDB0242067	438.2292	5.4712
Ganolucidic Acid C	3.232E-5	0.0001597	1.9567	10.8072	3.4339	up	LevelBii	HMDB0039691	551.3617	2.6169
Xanthoxylin	1.377E-5	7.908E-5	1.9355	5.2427	2.3903	up	LevelBi	HMDB0029645	197.0801	5.664
Flavone Base+3O,C-Hex-Feruloylhex	7.217E-6	4.582E-5	1.9361	0.2816	-1.8282	down	LevelBi	-	771.2079	4.1388
Prostaglandin F1A	0.0002095	0.0007305	1.9445	5.1781	2.3724	up	LevelBii	HMDB0002685	356.2592	2.7562
5-Pentyltetrahydro-2-Oxo-3-Furancarboxylic Acid	1.538E-9	1.534E-7	1.9456	0.1858	-2.4284	down	LevelBii	HMDB0030989	221.0784	4.7981
Met-Gly	4.605E-9	2.993E-7	1.9385	5.6031	2.4862	up	LevelBi	HMDB0028973	207.0785	1.6199
14-Hdohe	3.047E-11	1.634E-8	1.9365	0.0002	-12.4667	down	LevelBii	HMDB0060044	391.2059	2.4066
Delta-Valerolactone	8.166E-8	2.223E-6	1.9307	0.2315	-2.1109	down	LevelBii	HMDB0250981	199.0967	4.7981
Xi-7-Hydroxyhexadecanedioic Acid	1.39E-6	1.867E-5	1.922	0.1419	-2.8169	down	LevelBii	HMDB0037830	301.2006	5.7845
Ceanothine B	0.0003079	0.0009516	1.9114	4.1706	2.0602	up	LevelBii	HMDB0029339	568.2915	4.2569
Diisodityrosine	1.008E-7	2.63E-6	1.8983	0.2213	-2.1758	down	LevelBii	HMDB0029798	739.2182	3.9904
Clindamycin	2.732E-6	3.104E-5	1.9028	0.2756	-1.8594	down	LevelBii	HMDB0015321	425.1893	2.915
Lnape(16:3/N-5:0)	3.981E-11	1.787E-8	1.8986	12.0241	3.5879	up	LevelBi	-	576.299	2.9979
5-Hydroxydecanedioylcarnitine	2.691E-9	2.192E-7	1.8971	16.3512	4.0313	up	LevelBii	HMDB0241133	342.1907	3.6564
Tragopogonsaponin D	4.571E-6	3.847E-5	1.8895	0.2846	-1.813	down	LevelBii	HMDB0037915	1099.5133	5.6277
2,6,6-Trimethyl-3-Oxocyclohexene-1-Carboxylic Acid	4.081E-9	2.778E-7	1.8904	0.005	-7.6421	down	LevelBi	-	183.1003	4.7951
Tryptophan N-Glucoside	0.0006236	0.001696	1.8879	0.2052	-2.2851	down	LevelBii	HMDB0341443	367.1483	2.3402
Zearalanone	1.559E-7	3.617E-6	1.881	0.0119	-6.3954	down	LevelBii	HMDB0259993	341.1366	4.0277
Mifepristone	5.608E-7	9.425E-6	1.8789	0.0832	-3.5876	down	LevelBii	HMDB0014972	434.247	6.3883
6''-O-(3-Hydroxy-3-Methylglutaroyl)Astragalin	1.996E-8	7.939E-7	1.882	9.049	3.1778	up	LevelBii	HMDB0041129	651.1546	4.0928
Apiin	6.103E-7	1.003E-5	1.8776	0.1577	-2.6649	down	LevelBi	HMDB0030843;LMPK12110337	565.1509	3.991
Pgp(Pge2/I-14:0)	1.541E-6	2.013E-5	1.8741	0.0035	-8.1668	down	LevelBii	HMDB0275018	905.3961	6.1478
Vinyl Ether	4.079E-10	7.415E-8	1.884	0.0067	-7.2244	down	LevelBii	HMDB0259824	185.0807	5.3422
1-Methylene-5Alpha-Androstan-3Alpha-Ol-17-One	4.836E-5	0.0002232	1.867	0.4069	-1.2972	down	LevelBi	-	303.2307	7.0116
Cucurbitacin D	0.0001949	0.0006907	1.8668	3.3112	1.7273	up	LevelBii	HMDB0034695	516.3104	2.0614
N-(1-Deoxy-1-Fructosyl)Phenylalanine	1.2E-5	7.051E-5	1.865	0.2674	-1.9031	down	LevelBii	HMDB0037846	326.1233	2.1593
3-Phenylpropyl Acetate	7.194E-8	2.01E-6	1.8697	0.0008	-10.2292	down	LevelBii	HMDB0033386	223.096	5.6553
Apo-12'-Zeaxanthinal	3.161E-9	2.354E-7	1.8618	0.1696	-2.56	down	LevelBii	HMDB0302023	347.2417	4.8442
(S)-3-Hydroxy-N-Methylcoclaurine	3.356E-7	6.325E-6	1.8553	0.0502	-4.3156	down	LevelBii	HMDB0006921	316.1536	4.0598
Rimcazole	1.17E-5	6.897E-5	1.8511	3.0025	1.5862	up	LevelBii	HMDB0257233	342.192	3.3483

Metabolite	P_value	FDR	VIP_PLS-DA	FC(A/E)	Log2FC(A/E)	Regulate	level	Library ID	M/Z	Retention time
Halometasone	1.811E-9	1.723E-7	1.861	0.0018	-9.1092	down	Level1Bii	HMDB0253041	479.1176	4.8627
Cilengitide	3.647E-6	3.847E-5	1.8502	0.2493	-2.0043	down	Level1Bii	HMDB0250242	606.3357	6.398
19-Noretiocholanolone 3-O-Glucuronide	7.732E-5	0.0003288	1.8452	0.0836	-3.5803	down	Level1Bii	HMDB0341226	453.2456	6.0806
7(14)-Bisabolene-2,3,10,11-Tetrol	8.811E-9	4.618E-7	1.8506	0.1066	-3.23	down	Level1Bii	HMDB0035918	317.1952	4.8996
1,2-Diphenylhydrazine	0.0009796	0.002457	1.8657	0.164	-2.608	down	Level1Bi	-	217.1321	2.9844
Val-Pro-Ala	0.0001657	0.0006091	1.8488	2.4298	1.2809	up	Level1Bi	-	286.1769	6.2803
Smgdg(O-13:1/7:0)	6.538E-5	0.0002866	1.8384	0.0831	-3.5898	down	Level1Bi	-	671.3259	6.9054
N-Palmitoyl Gaba	1.727E-5	9.501E-5	1.8428	0.2491	-2.0054	down	Level1Bii	HMDB0062338	340.2829	8.5103
Convalloside	8.547E-6	5.277E-5	1.8361	0.1801	-2.473	down	Level1Bii	HMDB0034195	711.3171	6.2492
Pyrovalerone	0.0007367	0.001945	1.8513	2.6713	1.4176	up	Level1Bi	HMDB0256994	246.1846	6.2803
Tenuazonic Acid	2.858E-6	3.189E-5	1.8534	0.1061	-3.2369	down	Level1Bi	HMDB0036074	198.1115	4.9511
17-Debromo-Aplysiatoxin,	3.088E-5	0.0001537	1.838	4.2509	2.0878	up	Level1Bii	HMDB0248517	627.2984	2.4348
Ser-Ser	4.211E-8	1.371E-6	1.842	0.0263	-5.2489	down	Level1Bii	HMDB0029048	383.143	2.2829
Epothilone B	9.884E-10	1.145E-7	1.8409	17.8359	4.1567	up	Level1Bii	HMDB0251874	506.2583	3.1865
Npc	8.668E-7	1.312E-5	1.8303	0.0053	-7.5518	down	Level1Bii	HMDB0060499	1081.4294	5.2134
N-Monodemethyl Roxithromycin	3.2E-9	2.369E-7	1.8379	3.057	1.6121	up	Level1Bii	HMDB0060845	791.4929	7.6677
Fructosylvaline	0.001705	0.003863	1.8033	0.1574	-2.6675	down	Level1Bii	HMDB0252498	278.1238	1.156
Myristoleic Acid	4.197E-5	0.0001979	1.8225	0.3616	-1.4674	down	Level1Bi	HMDB0002000;LMFA01030051	227.1994	8.1247
Pgp(Pgf2Alpha/22:6(4Z,7Z,10Z,13Z,16Z,19Z))	1.2E-6	1.68E-5	1.8206	0.1031	-3.2782	down	Level1Bii	HMDB0274193	977.4479	5.322
Concanamycin	1.228E-5	7.187E-5	1.8175	4.2983	2.1038	up	Level1Bii	HMDB0250429	751.4694	3.2148
1,2-Cyclohexanedicarboxylic Acid, 4-Methyl-	2.952E-10	6.625E-8	1.8236	0.1433	-2.8033	down	Level1Bii	HMDB0253138	204.1219	3.5573
Slaframine	5.054E-7	8.733E-6	1.8102	4.8304	2.2722	up	Level1Bii	HMDB0243523	441.269	3.2242
Managlinat Dialanetil	7.703E-8	2.119E-6	1.8169	0.2228	-2.1664	down	Level1Bii	HMDB0254319	467.1862	2.5469
3-(2-Furanylmethylene)-2,4-Pentanedione	2.839E-6	3.174E-5	1.8108	192.1651	7.5862	up	Level1Bi	-	179.0693	5.664
Cdp-Dg(Pgf2Alpha/22:5(4Z,7Z,10Z,13Z,16Z))	1.891E-6	2.359E-5	1.8097	0.3418	-1.5488	down	Level1Bii	HMDB0292458	1146.516	5.4527
Dihydroouabain	6.297E-7	1.023E-5	1.8186	3.5562	1.8303	up	Level1Bii	HMDB0251322	641.3158	2.6369
Dlcl(14:0/16:4)	9.49E-5	0.0003873	1.809	0.2058	-2.2806	down	Level1Bi	-	885.4197	5.9512
3-Hydroxynevirapine Glucuronide	4.056E-9	2.774E-7	1.8177	0.1954	-2.3557	down	Level1Bii	HMDB0060743	479.1177	4.3429
Nona-5,7-Dienoic Acid	1.754E-9	1.691E-7	1.8142	0.1747	-2.5168	down	Level1Bii	HMDB0341026	137.0953	4.7951
Phenylacetylglutamine	1.271E-7	3.082E-6	1.8118	0.301	-1.7321	down	Level1Bii	HMDB0006344	323.1235	3.1301
17(18)-Epoxy-5Z,8Z,11Z,14Z-Eicosatetraenoic Acid	2.482E-6	2.886E-5	1.8105	0.3965	-1.3348	down	Level1Bi	HMDB0010212	319.2255	5.8592
Flurithromycin	7.806E-10	9.846E-8	1.8122	3.9775	1.9919	up	Level1Bii	HMDB0252409	732.4362	8.8614
Chlorogenic Acid	4.802E-6	3.847E-5	1.8098	5.6441	2.4967	up	Level1Bii	HMDB0003164	353.0863	2.7461
4-(2,2,6,6-Tetramethyl-1-Oxylpiperidin-4-Ylamino)-4'-Demethylepipodophyllotoxin	1.244E-6	1.727E-5	1.8056	0.4293	-1.2198	down	Level1Bii	HMDB0252920	537.2621	4.7364
(2R)-2-[[(2R)-2,6-Diaminohexanoyl]Amino]-3-Phenylpropanoic Acid	3.333E-13	1.197E-9	1.81	10.4782	3.3893	up	Level1Bi	-	316.1608	1.6103
Gly-Leu-Lys	3.187E-8	1.136E-6	1.8033	4.5787	2.1949	up	Level1Bi	-	317.2171	1.6589
N,N-Diallyl-N'-Phenylthiourea	0.003179	0.006527	1.8224	4.389	2.1339	up	Level1Bi	-	274.1368	3.2322
Cyclosquamosin D	4.432E-7	7.829E-6	1.8026	0.1337	-2.9029	down	Level1Bii	HMDB0303369	835.3926	5.5541
Torvonin A	7.857E-6	4.928E-5	1.7986	0.289	-1.7908	down	Level1Bii	HMDB0030732	759.414	6.4339
Androsterone Glucuronide	0.0001731	0.0006283	1.8003	0.3177	-1.6542	down	Level1Bii	HMDB0002829	525.2677	6.6005
Cyclopassifloside Iii	7.623E-6	4.808E-5	1.7974	6.1082	2.6107	up	Level1Bii	HMDB0038390	879.4563	2.9885
Kievitone Hydrate	0.0002438	0.0007876	1.8086	0.125	-2.9998	down	Level1Bii	HMDB0038111	373.127	4.2596
Phenylbutyric Acid	0.0001021	0.0004112	1.7912	0.1581	-2.6609	down	Level1Bi	-	165.09	4.7951
6-Hydroxy-4,4,7A-Trimethyl-5,6,7,7A-Tetrahydrobenzofuran-2(4H)-One	8.598E-10	1.029E-7	1.7953	0.2981	-1.7462	down	Level1Bi	-	197.1165	3.9318
Cdp-Dg(Pgj2/I-16:0)	6.543E-9	3.714E-7	1.7867	3.5319	1.8205	up	Level1Bii	HMDB0293707	1012.4714	2.576
Amp-Deoxynojirimycin	1.093E-5	6.5E-5	1.775	4.437	2.1496	up	Level1Bii	HMDB0248353	420.2691	2.5269
Pi(6 Keto-Pgf1Alpha/18:3(6Z,9Z,12Z))	0.002227	0.004827	1.7863	0.183	-2.4501	down	Level1Bii	HMDB0276784	981.466	6.6929
Vitamin D3 Glucosiduronate	1.365E-9	1.4E-7	1.775	5.5227	2.4654	up	Level1Bii	HMDB0259846	605.3648	3.6936
Tramadol N-Oxide	8.592E-5	0.0003573	1.7629	2.7133	1.44	up	Level1Bi	-	280.1892	3.7548
Methyl Cinnamate	2.348E-6	2.772E-5	1.7631	0.1447	-2.7886	down	Level1Bi	HMDB0303905;HMDB0033833	163.0744	4.0206
Methyl (9Z,14Z)-12,13,16-Trihydroxyoctadeca-9,14-Dienoate	1.175E-6	1.653E-5	1.7608	279.7968	8.1282	up	Level1Bi	-	384.2719	5.5369
Dibekacin	8.794E-5	0.0003636	1.7507	0.3277	-1.6096	down	Level1Bii	HMDB0251157	515.2809	4.7364

Metabolite	P_value	FDR	VIP_PLS-DA	FC(A/E)	Log2FC(A/E)	Regulate	level	Library ID	M/Z	Retention time
Lys-Ile-Asn	0.000218	0.0007512	1.7618	4.5636	2.1902	up	LevelBi	-	374.2368	1.9026
1-Cyclohexylcyclohexanecarboxylic Acid	0.000509	0.001436	1.7639	0.2849	-1.8113	down	LevelBi	-	211.1687	7.7542
Bn-Ncc-2	0.0003066	0.0009486	1.7651	0.1242	-3.009	down	LevelBii	HMDB0038230	837.3169	4.0091
7-Hydroxybutylidenephthalide 7-(6-Malonylglucoside)	6.278E-10	8.872E-8	1.7681	0.0925	-3.4349	down	LevelBii	HMDB0034753	497.13	4.7428
15-Stachen-19-Ol	0.001139	0.002773	1.7624	0.3539	-1.4986	down	LevelBi	-	289.2511	7.5701
Cyclohexanesulfamic Acid	5.678E-11	2.204E-8	1.7654	0.3886	-1.3638	down	LevelBi	HMDB0031340	180.068	2.8357
Zotarolimus	0.0007657	0.002006	1.762	2.3318	1.2214	up	LevelBii	HMDB0260048	1029.5873	7.8024
7-[(3-Methoxy-4-Propan-2-Yloxyphenyl)Methyl]-7-Azaspiro[3.5]Nonan-3-Ol	0.0001623	0.0005998	1.7541	0.2434	-2.0383	down	LevelBi	-	320.2202	6.7681
Meta-O-Dealkylated Flecainide Lactam	1.313E-9	1.356E-7	1.7608	0.1756	-2.5099	down	LevelBii	HMDB0060832	391.11	3.1301
Timosaponin B Ii	0.0002234	0.0007637	1.7542	0.419	-1.2549	down	LevelBi	-	925.4683	7.6294
Ala-Phe-Ser	2.51E-10	6.009E-8	1.7596	10.8512	3.4398	up	LevelBi	-	341.1844	4.4333
Fludrocortisone Acetate	3.783E-9	2.625E-7	1.7569	20.2366	4.3389	up	LevelBii	HMDB0252329	440.2447	4.1978
15(S)-Hpete	2.971E-7	5.789E-6	1.757	0.0591	-4.081	down	LevelBii	HMDB0004244	371.2025	4.9828
28-Glucosyloleanolic Acid 3-[Rhamnosyl-(1->2)-Galactosyl-(1->3)-Glucuronide]	3.235E-8	1.144E-6	1.7526	0.2544	-1.9748	down	LevelBii	HMDB0040859	1151.5092	5.4436
Esculetin	1.865E-6	2.338E-5	1.756	0.3025	-1.7252	down	LevelBi	HMDB0030819	177.0185	2.9885
Eriojaposide B	2.421E-5	0.0001261	1.7452	0.2826	-1.8233	down	LevelBii	HMDB0038029	1077.5079	7.3405
N-(1-Deoxy-1-Fructosyl)Proline	1.906E-5	0.0001032	1.7458	0.2566	-1.9622	down	LevelBii	HMDB0038493	278.1215	0.737
Oxoglutaric Acid	9.84E-10	1.145E-7	1.7585	0.212	-2.2376	down	LevelBii	HMDB0000208	205.0345	2.1593
Stearidonic Acid Ethyl Ester	0.0003016	0.0009363	1.7376	0.3252	-1.6206	down	LevelBi	-	305.2458	7.6196
Alpha-Cyperone	0.0002182	0.0007513	1.7509	0.214	-2.2243	down	LevelBi	-	219.174	6.447
Genistein 4'-O-Glucoside	0.000115	0.0004538	1.7464	0.2375	-2.0743	down	LevelBi	HMDB0252680;LMPK12050168	431.0955	4.139
Miglitol	5.581E-6	3.847E-5	1.7465	0.2509	-1.9948	down	LevelBii	HMDB0014634	188.0919	2.7083
N-[4-(Aminosulfonyl)Phenyl]Propanamide	1.25E-9	1.35E-7	1.7502	0.2949	-1.7617	down	LevelBi	-	229.063	2.9249
2-Amino-1-Phenylethanol	6.139E-5	0.0002726	1.7383	2.8288	1.5002	up	LevelBi	HMDB0001065	120.0797	1.6103
Formyl-5-Hydroxykynurenamine	0.0001341	0.0005152	1.74	5.1763	2.3719	up	LevelBii	HMDB0012948	267.0971	1.5948
Lpc(O-16:2)	4.158E-6	3.847E-5	1.7465	0.2579	-1.9553	down	LevelBi	-	522.3166	7.9126
Asp-Trp	9.323E-5	0.0003815	1.7453	2.6333	1.3969	up	LevelBi	HMDB0028764	320.1235	2.7762
Agnuside	5.621E-10	8.321E-8	1.7445	0.2016	-2.3103	down	LevelBii	HMDB0036561	431.1355	4.5992
10-Nitrolinoleic Acid	1.082E-5	6.449E-5	1.7317	4.1501	2.0531	up	LevelBii	HMDB0005049	370.2216	4.7704
Vellein	1.502E-8	6.543E-7	1.7378	0.044	-4.5077	down	LevelBii	HMDB0303360	413.1191	3.6936
Methoxpropamine	3.252E-9	2.382E-7	1.7375	2.2763	1.1867	up	LevelBi	-	262.1791	3.4295
Lpe(30:8)	8.934E-6	5.476E-5	1.7237	11.7367	3.5529	up	LevelBi	-	678.3789	2.9885
Sipoglitazar	5.728E-7	9.554E-6	1.7332	0.0086	-6.8572	down	LevelBii	HMDB0258310	444.1378	4.4817
Abscisic Acid	1.201E-8	5.674E-7	1.7328	0.1882	-2.4094	down	LevelBi	HMDB0035140;HMDB0036093	263.1273	4.6849
Thr-Arg-Lys-Glu	0.0001196	0.0004697	1.7226	0.0398	-4.6496	down	LevelBi	-	531.2957	5.9789
Lyciumoside Vii	1.582E-6	2.053E-5	1.725	0.4373	-1.1932	down	LevelBii	HMDB0033501	983.4666	4.8996
2-Phenyl-1,3-Propanediol Monocarbamate	7.732E-8	2.123E-6	1.7305	2.9496	1.5605	up	LevelBii	HMDB0060351	178.0872	2.2211
Hydroxygaleon	0.0003182	0.0009773	1.7105	0.361	-1.47	down	LevelBii	HMDB0031535	343.152	4.0206
3-O-Feruloylquinic Acid	1.09E-8	5.39E-7	1.7267	4.2761	2.0963	up	LevelBi	HMDB0030669	367.1016	3.196
Cis-3-Methyl Thiofentanyl	6.84E-6	4.379E-5	1.7201	0.1553	-2.6866	down	LevelBi	-	357.2009	5.7522
Gentamicin C	0.001875	0.004179	1.7123	2.7769	1.4735	up	LevelBii	HMDB0252688	470.2597	2.5665
Salidroside	1.258E-8	5.849E-7	1.7221	0.0076	-7.044	down	LevelBii	HMDB0257463	359.1342	3.7218
gamma-Glu-Cys-Gly	1.534E-7	3.583E-6	1.7205	2.2191	1.15	up	LevelBi	-	308.0903	1.2553
Asperulosidic Acid	5.711E-6	3.847E-5	1.716	10.5971	3.4056	up	LevelBii	HMDB0248668	491.1389	2.4817
Phe-Glu-Lys	2.303E-6	2.735E-5	1.7104	2.7861	1.4782	up	LevelBi	-	423.228	3.1825
Odalasvir	7.531E-5	0.0003221	1.7073	2.611	1.3846	up	LevelBii	HMDB0255919	999.5436	7.8296
(R)-Dihydroactinidiolide	2.108E-7	4.512E-6	1.7157	0.3659	-1.4503	down	LevelBi	-	181.1216	5.3904
Fluocortolone Caproate	3.182E-10	6.72E-8	1.7153	4.6714	2.2239	up	LevelBii	HMDB0252352	473.2754	3.8692
Retinol	6.151E-7	1.005E-5	1.7133	0.1656	-2.5942	down	LevelBi	LMPR01090001	269.225	4.6482
Lpc(O-14:0)	2.084E-5	0.0001115	1.7175	0.3966	-1.3341	down	LevelBi	LMGP01060007	498.3165	7.7115
Glycocholate Glucuronide	0.0001746	0.0006328	1.702	4.6668	2.2224	up	LevelBii	HMDB0341324	661.3858	3.9713
Magnolol	0.0003634	0.001089	1.6995	0.1757	-2.5089	down	LevelBi	HMDB0254305	265.1217	7.0719

Metabolite	P_value	FDR	VIP_PLS-DA	FC(A/E)	Log2FC (A/E)	Regulate	level	Library ID	M/Z	Retention time
Tyr-Ile-Leu	0.002903	0.00604	1.6934	3.768	1.9138	up	LevelBi	HMDB0013024	408.2464	3.6165
Rebaudioside A	0.0003043	0.0009431	1.7008	0.1118	-3.1614	down	LevelBii	HMDB0034950	967.4383	5.9668
Rosamicin	0.002528	0.005358	1.7112	2.5782	1.3664	up	LevelBii	HMDB0257306	580.3443	2.576
Pi(Pgf1Alpha/20:0)	8.468E-5	0.0003535	1.6933	1.7094	0.7735	up	LevelBii	HMDB0277060	1021.619	7.6962
Quercetin 3-Arabinoside	2.175E-7	4.635E-6	1.6969	6.7915	2.7637	up	LevelBi	HMDB0033795	433.0756	3.9904
5-(3-Pyridyl)-2-Hydroxytetrahydrofuran	3.396E-8	1.187E-6	1.7046	0.219	-2.1909	down	LevelBii	HMDB0062407	210.0761	3.0167
1-Deoxy-1-Morpholino-D-Fructose	4.267E-8	1.383E-6	1.7036	0	-15.5238	down	LevelBii	HMDB0243858	270.0939	3.4894
Misoprostol Acid	0.000254	0.0008129	1.677	0.2186	-2.1934	down	LevelBii	HMDB0254756	389.2282	7.6204
Lys-Ala-Arg	3.755E-8	1.269E-6	1.6902	4.4965	2.1688	up	LevelBii	HMDB0341172	408.2102	3.3296
5A,6A-Epoxy-7E-Megastigmene-3A,9E-Diol 3-Glucoside	0.01005	0.01736	1.7367	2.6238	1.3916	up	LevelBii	HMDB0031676	447.2227	2.6515
Adenosyl Cobyrinic Acid A,C Diamide	3.822E-6	3.847E-5	1.6821	0.2408	-2.0544	down	LevelBii	HMDB0001083	1167.4386	7.1364
Azepan-1-Yl-(6-Thiophen-2-Ylpyridin-3-Yl)Methanone	3.136E-8	1.128E-6	1.6879	0.2444	-2.0329	down	LevelBi	-	287.1236	4.6876
Mg(5-Iso Pgf2Vi/0:0/0:0)	5.216E-8	1.587E-6	1.6838	0.188	-2.4113	down	LevelBii	HMDB0260485	421.2186	5.4251
Gibberellin A28	6.912E-9	3.833E-7	1.6819	0.0562	-4.1529	down	LevelBii	HMDB0303573	375.1415	3.48
Bezitramide	7.648E-5	0.0003261	1.6756	1.8885	0.9172	up	LevelBii	HMDB0249171	534.2895	3.2418
Acetyldiphenylalanyl-Leucyl-Aspartyl-Isoleucyl-Isoleucyl-Tryptophan	2.706E-5	0.0001381	1.6759	0.2927	-1.7726	down	LevelBii	HMDB0256173	968.4728	5.9973
Lpc(22:5-Sn1)	0.0005585	0.001549	1.6767	2.1485	1.1033	up	LevelBi	-	592.3326	7.8902
Dodecyl-Beta-D-Maltoside	0.0004864	0.001383	1.6915	0.2806	-1.8332	down	LevelBii	HMDB0247203	491.2835	5.2964
Pi(3:0/18:5)	7.195E-5	0.0003103	1.6672	7.6973	2.9443	up	LevelBi	-	691.2705	3.4141
Pinacidil	4.14E-8	1.361E-6	1.6679	4.9301	2.3016	up	LevelBii	HMDB0256556	266.1383	4.0648
Arg-Glu-Leu	1.36E-5	7.839E-5	1.6657	3.5649	1.8338	up	LevelBi	-	417.2473	4.1586
Delta-Decalactone	1.275E-5	7.412E-5	1.662	0.3636	-1.4594	down	LevelBi	HMDB0037116;LMFA07040028	171.1369	7.7446
Histamine	8.484E-10	1.024E-7	1.6673	0.4873	-1.037	down	LevelBi	HMDB0000870;PW_C000693	112.0865	0.6298
1-Methylene-5Alpha-Androstan-3Alpha-Ol-17-One Glucuronide	6.855E-7	1.09E-5	1.6575	0.3375	-1.5672	down	LevelBii	HMDB0341213	479.2605	5.2342
2,6,10,10-Tetramethyl-1-Oxaspiro[4.5]Decan-6-Ol	0.0002498	0.0008038	1.6539	0.4685	-1.0937	down	LevelBii	HMDB0037907	213.1835	8.115
N-Docosahexaenoyl Valine	5.108E-5	0.0002337	1.6563	0.4179	-1.2588	down	LevelBii	HMDB0242025	460.3389	5.2049
Phe-Hyp	5.78E-5	0.0002598	1.6538	0.3706	-1.4321	down	LevelBii	HMDB0011176	261.1224	3.2023
Ala-Gly-Tyr	0.0009195	0.002337	1.6615	2.3642	1.2413	up	LevelBi	-	310.138	1.8336
Apc	6.404E-6	4.15E-5	1.6507	0.2543	-1.9755	down	LevelBii	HMDB0060661	677.2796	4.2873
Pravastatin	2.78E-6	3.131E-5	1.6508	0.2281	-2.1321	down	LevelBii	HMDB0005022	483.2561	5.9789
Ps(Pgf2Alpha/14:1(9Z))	1.217E-7	2.996E-6	1.6593	3.7637	1.9121	up	LevelBii	HMDB0280933	784.4384	2.7933
2',4',6'-Trihydroxydihydrochalcone 2'-Glucoside	1.208E-6	1.688E-5	1.6497	0.1607	-2.6378	down	LevelBii	HMDB0037483	441.1133	4.2596
23-Hydroxyphysalolactone	9.608E-5	0.0003905	1.644	4.7032	2.2336	up	LevelBii	HMDB0031388	613.2376	3.2242
Kaempferol 3-[Apiosyl-(1->2)-Galactoside]	3.403E-6	3.674E-5	1.6453	0.0085	-6.8726	down	LevelBii	HMDB0037430	579.1333	3.6657
Glycocholic Acid	1.86E-5	0.0001012	1.6413	2.2977	1.2002	up	LevelBi	HMDB0000138;LMST05030001;PW_C000089	470.2928	2.567
Pip(Pgf2Alpha/16:0)	1.899E-5	0.0001029	1.6425	0.207	-2.2725	down	LevelBii	HMDB0278446	1023.4594	5.5172
N-Phenethylacetamide	3.927E-7	7.12E-6	1.6505	0.2844	-1.8139	down	LevelBi	HMDB0255222	164.1061	4.1783
Naratriptan	1.495E-9	1.512E-7	1.6462	3.6597	1.8717	up	LevelBii	HMDB0015087	336.1732	3.7746
Pyrrolidino Paf C-16	2.953E-6	3.275E-5	1.6437	1.8893	0.9179	up	LevelBi	-	550.3834	8.6503
Serofendic Acid	3.597E-7	6.674E-6	1.6473	0.2089	-2.2591	down	LevelBii	HMDB0258253	417.1866	5.8587
Lpg(20:5)	2.792E-9	2.222E-7	1.6493	0.2308	-2.1154	down	LevelBi	LMGP04050030	575.2677	3.48
Succinylcholine	6.739E-6	4.328E-5	1.6375	2.1891	1.1303	up	LevelBi	HMDB0014347	273.2161	4.0993
Streptozocin	1.131E-7	2.87E-6	1.6522	0.1967	-2.3459	down	LevelBii	HMDB0014572	264.0855	3.675
Prolinamide,D-Tyrosyl-L-Arginylglycyl-4-Nitro-L-Phenylalanyl-	2.775E-7	5.511E-6	1.6367	0.4411	-1.1809	down	LevelBii	HMDB0247954	727.3134	5.3238
Spinorphin	0.0007569	0.001989	1.6375	0.1889	-2.4046	down	LevelBii	HMDB0059790	875.4637	6.7023
Lys-Ala	8.196E-5	0.0003442	1.6321	3.4214	1.7746	up	LevelBi	HMDB0028944	200.139	1.1185
4,4-Dimethyl-2-[3-Carboxylatopropyl]-2-Tridecyloxazolidine 3-Oxide	0.0007072	0.001877	1.6412	0.4166	-1.2632	down	LevelBii	HMDB0246830	366.2991	8.5851
Etiocholanolone Glucuronide	0.004791	0.009248	1.6418	3.0974	1.631	up	LevelBii	HMDB0004484	508.2924	3.6363
Leucic Acid	4.702E-8	1.478E-6	1.634	0.07	-3.8369	down	LevelBii	HMDB0000624	113.0599	5.3422
Desmethyldihydrocapsaicin	0.000159	0.0005889	1.6259	0.1594	-2.6495	down	LevelBi	-	292.19	6.3508
Lys-Asp-Tyr	6.88E-5	0.0002991	1.6213	0.1121	-3.1577	down	LevelBii	HMDB0304806	459.1635	6.1478
Penitrem E	0.0008885	0.00227	1.6274	6.6842	2.7407	up	LevelBii	HMDB0256241	620.3041	2.6894
6-Tert-Butyl-1,2,3,4-Tetrahydroquinoline	0.001	0.002	1.640	2.547	1.3489	up	Leve	-	244.1	3.3505

Metabolite	P_value	FDR	VIP_PLS-DA	FC(A/E)	Log2FC(A/E)	Regulate	level	Library ID	M/Z	Retention time
	159	812	7	3			lBi		687	
7Beta-Hydroxy-7-Desacetoxykhivorinic Acid, Methyl Ester	0.0001503	0.0005643	1.6253	0.148	-2.7566	down	LevelBi	-	571.2377	4.8627
Carpinontriol B	4.293E-7	7.633E-6	1.6187	0.4011	-1.3179	down	LevelBi	-	345.1304	3.6363
Yessotoxin	1.045E-8	5.267E-7	1.6207	0.4122	-1.2784	down	LevelBii	HMDB0033320	1187.4818	5.4527
[8]-Shogaol	0.001651	0.003761	1.6221	0.3309	-1.5955	down	LevelBi	HMDB0031463	322.2359	7.0989
Matairesinol	0.0007821	0.00204	1.6163	0.3169	-1.6579	down	LevelBii	HMDB0035698	357.1324	4.2873
Cyclovirobuxine D	0.001383	0.003254	1.6248	0.2336	-2.0976	down	LevelBi	HMDB0250688	407.3388	7.0892
15-Epi Prostaglandin A1	9.292E-5	0.0003803	1.6058	0.4046	-1.3055	down	LevelBi	-	337.2354	5.8592
Metoprolol	7.991E-6	4.996E-5	1.6082	3.9921	1.9971	up	LevelBii	HMDB0001932	312.1804	3.045
Tomatine	4.387E-5	0.0002052	1.6059	0.4323	-1.21	down	LevelBii	HMDB0034103	1092.5546	5.0105
Tyr-Asp	0.0001152	0.0004545	1.6079	2.5164	1.3314	up	LevelBi	HMDB0029101	297.1059	1.8336
Quadranoside Iii	0.00194	0.0043	1.6046	2.3802	1.2511	up	LevelBi	-	705.3549	3.5968
Adomeglivant	1.172E-6	1.652E-5	1.6026	0.5195	-0.9448	down	LevelBii	HMDB0248020	573.2949	2.0512
10-Formyldihydrofolate	1.462E-7	3.471E-6	1.6075	0.3383	-1.5637	down	LevelBii	HMDB0006485	470.1423	2.5853
Arg-Ile	1.205E-7	2.978E-6	1.6128	3.2822	1.7147	up	LevelBii	HMDB0028712	286.1876	1.8648
Ethylphosphate	7.913E-5	0.0003345	1.6068	2.9219	1.5469	up	LevelBii	HMDB0012228	124.9997	0.7458
Trp-Val-Ala	0.0005183	0.001457	1.5988	4.2857	2.0995	up	LevelBi	-	375.2035	2.7362
Tyr-Hyp	6.613E-9	3.724E-7	1.6054	0.1227	-3.0268	down	LevelBii	HMDB0029106	339.1181	2.2258
1,2,3,4-Tetrahydro-6-Propanoylpyridine	2.761E-8	1.033E-6	1.61	0.2127	-2.2334	down	LevelBii	HMDB0034884	198.1124	4.38
Ethyl Acrylate	4.795E-9	3.083E-7	1.6042	0.3611	-1.4696	down	LevelBi	HMDB0033978	101.0593	2.2706
Azadiradione	9.746E-5	0.0003956	1.5987	3.9206	1.9711	up	LevelBi	-	449.2372	2.7461
Lys-Tyr	0.00281	0.005876	1.6077	2.4491	1.2923	up	LevelBi	HMDB0028963	310.1751	1.3911
Phe-Glu	0.001375	0.00324	1.6069	2.0369	1.0264	up	LevelBi	HMDB0028994	295.1273	2.1512
Gymnemic Acid I	0.001614	0.003692	1.5954	2.958	1.5646	up	LevelBii	HMDB0341056	787.4335	4.139
(2S,3R,4R,5S,6S)-2-(Hydroxymethyl)-6-Phenylmethoxyoxane-3,4,5-Triol	8.432E-6	5.217E-5	1.5904	0.2729	-1.8734	down	LevelBii	HMDB0243491	269.1015	3.1396
3-Ureidopropionic Acid	3.964E-8	1.32E-6	1.5916	0.2975	-1.7489	down	LevelBi	HMDB0000026	115.0495	1.1285
Pi(Pgf2Alpha/22:2(13Z,16Z))	2.588E-5	0.0001329	1.5912	1.7827	0.8341	up	LevelBii	HMDB0277722	1043.6008	7.6962
His-Ile-Gly	0.004479	0.008726	1.6053	2.5119	1.3288	up	LevelBi	-	326.1802	1.8827
Avicularin	0.0006705	0.001798	1.6268	3.8207	1.9338	up	LevelBii	HMDB0030860	433.0755	4.1298
1-Linoleoyl Glycerol	0.02563	0.03897	1.6568	2.2671	1.1808	up	LevelBi	-	355.2829	8.3582
Lys-Pro	1.242E-5	7.259E-5	1.5855	2.1181	1.0828	up	LevelBi	HMDB0028959	244.1644	2.1311
Diphenylsulfone	0.001498	0.003476	1.6514	4.0822	2.0293	up	LevelBi	HMDB0251453	219.0463	5.3024
Peridinin	0.000353	0.001065	1.5869	2.2687	1.1819	up	LevelBii	HMDB0256341	635.3364	2.517
Milbemycin Alpha5	2.89E-7	5.662E-6	1.5882	2.5441	1.3471	up	LevelBii	HMDB0254723	736.4017	2.6467
2Beta-Hydroxytestosterone	2.812E-5	0.0001422	1.5882	0.1799	-2.4749	down	LevelBii	HMDB0012654	349.1992	5.5908
Asp-Phe	7.951E-5	0.000336	1.6041	2.4161	1.2727	up	LevelBi	HMDB0000706	281.1124	2.4294
Abu-Abu-Oh	0.000877	0.002248	1.5906	2.5877	1.3717	up	LevelBi	-	295.0924	1.8366
Qynad	9.264E-8	2.458E-6	1.5801	4.7012	2.233	up	LevelBii	HMDB0006730	668.2505	3.2337
Tramadol	0.0009995	0.002493	1.5961	1.863	0.8977	up	LevelBi	HMDB0014339;HMDB0259401;PW_C008740	264.195	6.2803
Phe-Asp	0.004185	0.008234	1.5957	2.1452	1.1011	up	LevelBi	HMDB0028991	281.1135	2.1311
Arg-Met-His	2.207E-9	1.932E-7	1.5812	0.3687	-1.4394	down	LevelBi	-	460.2478	1.1086
Phe-His	7.814E-5	0.0003317	1.5774	2.1657	1.1148	up	LevelBi	HMDB0028892	303.1433	1.6688
Val-Phe-Val-Tyr	3.446E-8	1.195E-6	1.5853	0.4668	-1.0992	down	LevelBii	HMDB0304812	571.2819	2.0535
Cdp-Dg(Lte4/I-14:0)	2.148E-5	0.0001141	1.5727	0.3971	-1.3324	down	LevelBii	HMDB0293501	1167.4993	5.6368
Vindoline	2.482E-5	0.0001287	1.5852	8.4668	3.0818	up	LevelBii	HMDB0259814	498.2594	2.7562
Mlcl(14:0/14:1/18:3)	0.00106	0.002613	1.5735	0.4086	-1.2912	down	LevelBi	-	1123.6365	7.5551
Oxiracetam	3.302E-7	6.256E-6	1.5729	2.978	1.5743	up	LevelBii	HMDB0255986	123.0538	1.3528
N-(1-Deoxy-1-Fructosyl)Isoleucine	2.58E-7	5.211E-6	1.5787	0.5035	-0.9899	down	LevelBii	HMDB0039780	292.1391	1.9403
Soybean Saponin Bg	0.0002231	0.0007631	1.5616	0.3722	-1.4257	down	LevelBii	HMDB0302658	945.4665	7.3871
Roxindole	8.592E-6	5.3E-5	1.583	0.2385	-2.0677	down	LevelBii	HMDB0257319	405.2205	2.7743
Eugenosedin-A	2.132E-8	8.365E-7	1.5652	20.4588	4.3546	up	LevelBii	HMDB0252125	475.2026	1.8648
Kudzusaponin Sa2	4.963E-5	0.000228	1.5588	0.423	-1.2414	down	LevelBii	HMDB0041241	979.472	5.2227
Borrelidin	5.941E-5	0.0002655	1.551	1.7796	0.8315	up	LevelBii	HMDB0341293	472.3101	3.735

Metabolite	P_value	FDR	VIP_PLS-DA	FC(A/E)	Log2FC (A/E)	Regulate	level	Library ID	M/Z	Retention time
Lys-Phe	0.00453	0.008811	1.5748	1.9135	0.9362	up	LevelBi	HMDB0028958	294.1805	1.5909
Cryptophycin 52	0.0006706	0.001798	1.562	2.6667	1.415	up	LevelBii	HMDB0250550	701.3171	2.0216
2-Pyrrolidinone	5.87E-6	3.849E-5	1.5518	0.5037	-0.9894	down	LevelBi	HMDB0002039	86.0598	0.6887
Pesticide4_Ivermectin_C48H74014_22,23-Dihydroavermectin B1	0.0007226	0.001911	1.5602	2.5929	1.3745	up	LevelBii	HMDB0247302	839.4932	4.1288
(S)-2-((2-Hydroxy-2-Phenylethyl)Amino)-2-Methylpropan-1-Ol	1.986E-6	2.459E-5	1.5636	0.1572	-2.6691	down	LevelBi	-	242.1747	3.9416
Indinavir	5.259E-5	0.0002399	1.5426	3.4616	1.7914	up	LevelBi	HMDB0014369	612.3493	2.7461
5Beta-Androstan-3Alpha-Ol-17-One Sulfate	1.889E-9	1.765E-7	1.5581	3.5278	1.8188	up	LevelBi	-	369.1765	2.463
Arg-Thr-Lys-Arg	7.48E-6	4.726E-5	1.5491	2.0633	1.0449	up	LevelBii	HMDB0304796	524.3406	3.4882
D[-Arg-2]Kyotorphan	0.0008529	0.002198	1.5598	2.9053	1.5387	up	LevelBi	-	336.1666	1.2213
2-{2-[2-(Dimethylamino)Ethoxy]Ethoxy}-N,N-Dimethylethanamine	2.237E-6	2.677E-5	1.5491	1.8375	0.8778	up	LevelBi	-	259.2008	3.735
Tyr-Asn	0.0009949	0.002485	1.5605	2.2427	1.1653	up	LevelBi	HMDB0029100	296.1229	1.4008
(4-Formyl-9-Methoxy-5-Methylnaphtho[2,3-B]Furan-3-Yl)Methyl Acetate	7.9E-6	4.95E-5	1.5475	0.3922	-1.3504	down	LevelBi	-	345.1303	4.2569
(6E,9E)-Hexadeca-6,9-Dienedioylcarnitine	1.573E-5	8.83E-5	1.5406	1.8118	0.8574	up	LevelBii	HMDB0241489	430.2556	3.2715
2-Phenylethyl Acetate	3.374E-9	2.459E-7	1.5472	0.1732	-2.5296	down	LevelBii	HMDB0033945	209.0808	3.6564
5-Methoxy-6-(2-Propenyl)-1,3-Benzodioxole	1.648E-8	6.982E-7	1.5501	0.2162	-2.2098	down	LevelBii	HMDB0303894	227.0482	2.9322
Trp-Ser	4.949E-7	8.626E-6	1.5511	0.216	-2.2107	down	LevelBii	HMDB0029092	336.1183	2.6326
Lpc(O-18:4)	0.0001046	0.0004192	1.537	0.2415	-2.0498	down	LevelBi	-	546.3154	7.611
2-{1-[2-(1-Benzothiophen-5-Ylamino)-2-Oxoethyl]Cyclohexyl}Acetic Acid	1.604E-6	2.076E-5	1.5525	17.7493	4.1497	up	LevelBi	-	332.1332	1.8237
Pro-Pro	0.0001327	0.0005108	1.5595	0.4719	-1.0834	down	LevelBi	HMDB0011180	213.1222	0.737
Ganosporeric Acid A	2.103E-5	0.0001122	1.5302	3.2695	1.7091	up	LevelBii	HMDB0033022	527.2611	3.2715
4-(Difluoromethyl)-2-Hydrazinyl-6-(3-Methoxyphenyl)Pyrimidine	4.731E-5	0.0002193	1.5605	3.0798	1.6228	up	LevelBi	-	231.0829	1.6688
Mocimycin	0.007105	0.01292	1.5251	3.6252	1.8581	up	LevelBii	HMDB0030465	797.4239	3.3112
Gant 61	4.919E-5	0.0002263	1.5277	1.985	0.9892	up	LevelBi	HMDB0252638	430.298	2.2706
Trans-O-Coumaric Acid 2-Glucoside	2.872E-7	5.634E-6	1.5417	0.2613	-1.9359	down	LevelBi	HMDB0033581	325.0917	2.7555
Val-Cys	0.006406	0.01185	1.5641	2.3575	1.2372	up	LevelBi	HMDB0029124	221.094	1.6493
11-Alpha-O-Beta-D-Glucopyranosyl-16Alpha-O-Methylneoquassin	6.225E-5	0.0002758	1.5212	2.5423	1.3461	up	LevelBii	HMDB0039773	569.2901	4.109
Tyr-Gly-Gly-Trp-Leu	0.0001258	0.0004889	1.5552	0.1794	-2.4785	down	LevelBii	HMDB0259342	615.2601	3.48
N-Decanoyl-Dl-Homoserine Lactone	9.752E-10	1.145E-7	1.5309	2.4215	1.2759	up	LevelBi	-	256.1889	4.2271
3,4-Dihydroxyhydrocinnamic Acid	1.072E-5	6.4E-5	1.5252	2.701	1.4335	up	LevelBii	HMDB0000423	163.0389	1.846
Multinoside A	2.929E-6	3.253E-5	1.5233	0.3099	-1.69	down	LevelBii	HMDB0037935	609.1427	3.9998
Pro-Lys-Ile	0.0002364	0.00078	1.5272	2.9077	1.5399	up	LevelBii	HMDB0341190	355.2337	2.0914
Arg-Pro-Pro	1.707E-6	2.187E-5	1.5209	0.3732	-1.4218	down	LevelBi	-	373.1943	5.7323
Americine	0.007071	0.01287	1.5331	2.1057	1.0743	up	LevelBii	HMDB0034441	528.299	2.4582
Avenacoside A	0.0001985	0.0007002	1.5176	0.4499	-1.1522	down	LevelBii	HMDB0033815	1107.5183	7.2847
Muzanzagenin	1.506E-5	8.515E-5	1.5293	2.5888	1.3723	up	LevelBii	HMDB0032601	484.31	3.0238
N-Acetylneuraminic Acid	5.293E-8	1.604E-6	1.5326	0.5444	-0.8772	down	LevelBii	HMDB0000230	290.0868	1.6041
Coumarin	0.001965	0.004345	1.522	2.192	1.1323	up	LevelBi	HMDB0001218	147.0427	1.8436
Ile-Glu-Ile	0.0001246	0.0004854	1.5197	2.9381	1.5549	up	LevelBi	-	396.2085	4.3253
6-Piperazin-1-Yl-Isoquinoline	1.218E-7	2.996E-6	1.522	2.3833	1.2529	up	LevelBi	-	313.1192	2.1912
Homogentisic Acid	9.365E-8	2.477E-6	1.5145	4.8807	2.2871	up	LevelBii	HMDB0000130	167.0336	4.0091
Hemicalyculin A	0.003307	0.006753	1.5293	1.6928	0.7594	up	LevelBii	HMDB0253083	1006.6312	8.0959
Pro-Ile	3.067E-6	3.37E-5	1.5218	0.4837	-1.0477	down	LevelBi	HMDB0304810	211.143	2.1912
Polyporusterone B	0.004737	0.00916	1.5321	2.0482	1.0343	up	LevelBii	HMDB0038496	997.6247	7.9407
N-[(5-Hydroxy-2-Pyridinyl)Methyl]Adenosine	8.294E-6	5.145E-5	1.5072	0.4192	-1.2543	down	LevelBii	HMDB0040912	397.1227	4.2569
Wikstromol	2.36E-6	2.78E-5	1.5064	0.2367	-2.0791	down	LevelBii	HMDB0030598	355.116	4.2596
Benzoic Acid	0.0001007	0.0004061	1.503	1.846	0.8844	up	LevelBi	HMDB0001870	123.0432	1.8436
Pe-Cer(12:2_20/18:5)	1.372E-7	3.273E-6	1.5042	3.5356	1.822	up	LevelBi	-	637.3579	8.9824
Palmitoyl Glucuronide	0.02082	0.03256	1.5647	2.0794	1.0561	up	LevelBii	HMDB0010331	399.2736	8.3544
Lisinopril	4.003E-9	2.75E-7	1.5101	5.871	2.5536	up	LevelBii	HMDB0001938	440.1994	4.3244
Aconitic Acid	2.268E-6	2.71E-5	1.5222	0.2943	-1.7645	down	LevelBi	HMDB0247961	173.0079	1.9403
Ethylketocyclazocine	1.71E-5	9.431E-5	1.4961	2.1627	1.1128	up	LevelBii	HMDB0242274	322.1756	2.8059
Glucitol-Lysine	0.0005548	0.00154	1.5053	2.2352	1.1604	up	LevelBii	HMDB0252764	275.1598	2.3602
10,11-Dihydro-12R-Hydroxy-Leukotriene E4	0.000	0.000	1.504	2.603	1.3806	up	Leve	HMDB0012501	458.2	1.9224

Metabolite	P_value	FDR	VIP_PLS-DA	FC(A/E)	Log2FC(A/E)	Regulate	level	Library ID	M/Z	Retention time
	2592	8263		7			lBii		566	
(R)-6’-O-(4-Geranyloxy-2-Hydroxycinnamoyl)-Marmin	0.0002491	0.0008019	1.5004	2.595	1.3758	up	LevelBii	HMDB0039061	635.2958	2.3204
1-Myristoyl-2-Hydroxy-Sn-Glycero-3-Phosphoethanolamine	3.957E-7	7.161E-6	1.5009	2.8031	1.487	up	LevelBi	HMDB0011500	426.2593	7.5209
N-(3,5-Dimethylphenyl)-2-(5-Pyrazin-2-Yl-1,3,4-Oxadiazol-2-Yl)Acetamide	2.012E-7	4.338E-6	1.5093	0.5801	-0.7857	down	LevelBi	-	310.1281	2.1616
3-Butyn-1-Al	0.0001613	0.0005965	1.4988	0.4621	-1.1137	down	LevelBii	HMDB0006853	69.0334	0.6887
Tyr-gamma-Glu	1.989E-5	0.0001072	1.495	1.9482	0.9621	up	LevelBii	HMDB0029119	308.1239	1.8366
(2S)-2-Amino-7-(1-Aminoethylideneamino)-5-Sulfanylideneheptanoic Acid	0.0004987	0.001413	1.5006	2.7775	1.4738	up	LevelBii	HMDB0252980	276.1014	1.3241
Garcimangosone C	0.0001015	0.0004092	1.495	0.2126	-2.2341	down	LevelBii	HMDB0036984	471.1686	2.5194
Pinoquercetin	0.0004558	0.001309	1.492	0.356	-1.4901	down	LevelBii	HMDB0256564	337.0309	5.2503
Hyp-Val	0.001613	0.003691	1.5028	1.9666	0.9757	up	LevelBii	HMDB0028876	251.1021	2.082
Delimotecan	0.002018	0.004447	1.5021	3.2984	1.7218	up	LevelBii	HMDB0250970	655.2244	2.3969
Shexcer(10:0_20/20:1_0)	0.001317	0.003122	1.4907	2.0513	1.0365	up	LevelBi	-	762.44	2.796
Ala-Leu-Val-Arg	0.004122	0.008124	1.5115	1.7547	0.8112	up	LevelBi	-	458.3057	2.7262
Cinnamyl Anthranilate	1.899E-5	0.0001029	1.486	0.2728	-1.8742	down	LevelBii	HMDB0041319	312.1218	4.4169
Benzyl[(3-Methylphenyl)Methyl]Amine	0.0002321	0.00078	1.5006	1.9047	0.9296	up	LevelBi	-	244.1684	3.4295
Micronomicin	6.25E-5	0.0002767	1.4834	1.8008	0.8487	up	LevelBii	HMDB0254707	527.3143	3.4588
[2-Amino-2-(Hydroxymethyl)-5-(4-Octylphenyl)Pentyl] Dihydrogen Phosphate	2.955E-8	1.091E-6	1.4891	4.073	2.0261	up	LevelBii	HMDB0252505	400.2291	1.846
Queuine	2.62E-7	5.264E-6	1.4855	0.4432	-1.1739	down	LevelBii	HMDB0001495	278.1223	1.02
Mgdg(O-16:4/2:0)	0.0001291	0.0004994	1.4779	2.9002	1.5362	up	LevelBi	-	557.2937	4.4169
Quercetin 3-O-Glucoside	8.109E-6	5.053E-5	1.4768	1.8343	0.8753	up	LevelBi	HMDB0037362	463.0876	3.8321
Ile-Leu-Arg	0.003607	0.007265	1.4831	2.0669	1.0475	up	LevelBi	-	401.2848	2.5469
Rifamycin	6.103E-9	3.572E-7	1.4861	2.8209	1.4962	up	LevelBi	LMPK05000005	696.2951	3.1583
Dihydro-2,5-Furandione	1.761E-5	9.669E-5	1.4808	0.3959	-1.3367	down	LevelBi	HMDB0032523	101.0226	1.7849
Epothilone A	0.0004997	0.001415	1.4799	2.0889	1.0628	up	LevelBii	HMDB0251873	458.2408	3.4588
N-Arachidonoyl Asparagine	5.454E-6	3.847E-5	1.4729	1.9869	0.9905	up	LevelBii	HMDB0241991	423.2621	8.833
(24E)-3Alpha,15Alpha-Diacetoxy-23-Oxo-7,9(11),24-Lanostatrien-26-Oic Acid	0.006284	0.01166	1.4889	2.3623	1.2402	up	LevelBii	HMDB0035388	627.3484	4.0742
Gambogic Acid	0.0006726	0.001802	1.4664	0.6082	-0.7174	down	LevelBii	HMDB0034095	687.3194	6.0435
Calenduloside H Methyl Ester	0.001185	0.002863	1.4784	0.2857	-1.8076	down	LevelBii	HMDB0041021	1005.4864	7.2568
Dialdehyde	3.095E-10	6.72E-8	1.4775	5.6446	2.4969	up	LevelBii	HMDB0251125	403.1463	4.4538
Lpc(22:4)	0.0001283	0.0004972	1.4674	1.5253	0.6091	up	LevelBi	HMDB0010401	616.3593	8.0341
Deoxycholylmethionine	0.006402	0.01185	1.5022	2.878	1.525	up	LevelBii	HMDB0242435	568.3339	3.8134
N-Cyclooctyl-1-(4-Fluorobenzoyl)Piperidine-4-Carboxamide	9.411E-6	5.727E-5	1.4691	0.494	-1.0175	down	LevelBi	-	361.2309	5.5077
Cdp-Dg(Pgf2Alpha/18:1(11Z))	1.797E-10	4.778E-8	1.4718	15.0951	3.916	up	LevelBii	HMDB0291106	1122.5379	3.527
8-Hydroxy-Deoxyguanosine	3.124E-5	0.0001552	1.4711	1.9598	0.9707	up	LevelBii	HMDB0003333	316.1276	2.7064
Dg(Pgf1Alpha/15:0/0:0)	1.694E-6	2.173E-5	1.4659	0.5854	-0.7726	down	LevelBii	HMDB0295384	637.4986	8.1247
Gingerglycolipid A	1.503E-7	3.534E-6	1.4642	0.484	-1.047	down	LevelBii	HMDB0041093	675.3567	5.4988
3-(Benzoyloxy)-2-Hydroxypropyl ?-D-Glucopyranosiduronic Acid	2.359E-8	9.084E-7	1.4645	2.5578	1.3549	up	LevelBi	-	371.0969	3.3483
Glu-Ile-Lys	1.409E-6	1.886E-5	1.4612	1.7424	0.8011	up	LevelBi	-	371.2278	2.1616
Pgp(Txb2/I-18:0)	0.000203	0.0007123	1.4645	0.3925	-1.3492	down	LevelBii	HMDB0275447	979.4694	5.6646
Annonacin	0.0008742	0.002244	1.4682	0.3063	-1.7069	down	LevelBii	HMDB0033327	617.4354	9.3543
Capecitabine	4.886E-8	1.519E-6	1.4707	32.9168	5.0408	up	LevelBii	HMDB0015233	418.1634	4.1947
4-Phenyl-1,2,3-Thiadiazole	3.087E-5	0.0001537	1.4591	1.994	0.9957	up	LevelBi	-	204.0604	0.7955
Phorbol Myristate	1.814E-5	9.914E-5	1.4536	1.8749	0.9068	up	LevelBii	HMDB0256455	574.3823	8.4157
12-Hydroxystearic Acid	0.002063	0.004528	1.4593	0.6354	-0.6543	down	LevelBi	HMDB0061706	301.2724	8.115
Byssochlamic Acid	2.105E-5	0.0001122	1.4569	0.2657	-1.9119	down	LevelBii	HMDB0034262	353.1007	4.3708
Mgdg(O-22:5/2:0)	0.0001562	0.000581	1.4568	0.5425	-0.8823	down	LevelBi	-	595.3806	7.0504
Neoacrimarine A	6.946E-6	4.444E-5	1.458	4.2571	2.0899	up	LevelBii	HMDB0040384	716.2617	7.443
Nifedipine	6.14E-6	3.992E-5	1.4573	0.5032	-0.9909	down	LevelBi	HMDB0015247;PW_C009534	347.1219	2.905
Arg-His	5.874E-5	0.0002633	1.4524	0.5428	-0.8816	down	LevelBii	HMDB0028711	356.1686	3.5552
Trp-Ile-Leu	0.006423	0.01188	1.4827	2.138	1.0963	up	LevelBi	-	431.2634	4.3843
Isocolumbin	2.735E-6	3.105E-5	1.4465	0.3776	-1.4049	down	LevelBii	HMDB0036837	339.1213	4.1855
N-(1-Deoxy-1-Fructosyl)Histidine	0.001028	0.002549	1.4441	2.6361	1.3984	up	LevelBii	HMDB0037839	359.1556	4.4527
Pyropheophorbide A	0.0001145	0.0004523	1.4445	0.4728	-1.0808	down	LevelBii	HMDB0256993	1113.5288	5.8032

Metabolite	P_value	FDR	VIP_PLS-DA	FC(A/E)	Log2FC(A/E)	Regulate	level	Library ID	M/Z	Retention time
Etimicin	0.002172	0.004724	1.457	3.1798	1.6689	up	LevelBii	HMDB0252101	458.2964	2.7933
Bafilomycin A1	1.032E-7	2.675E-6	1.452	2.6918	1.4286	up	LevelBii	HMDB0247559	667.4006	2.8121
Leu-Ser-Phe	0.0004878	0.001386	1.4406	2.1101	1.0773	up	LevelBi	-	366.2006	3.41
1,2,3,4-Tetrahydroisoquinoline-3-Carboxylic Acid	8.695E-5	0.0003604	1.4508	0.5869	-0.7688	down	LevelBii	HMDB0244117	236.0917	3.7311
Mabioside D	3.728E-6	3.847E-5	1.4392	4.0457	2.0164	up	LevelBii	HMDB0040655	819.4577	3.2148
Pi(6 Keto-Pgf1Alpha/20:1(11Z))	0.01395	0.02302	1.4698	1.6757	0.7448	up	LevelBii	HMDB0277096	977.5618	7.7297
Decanoyl-L-Carnitine	0.006842	0.01251	1.4418	3.3462	1.7425	up	LevelBi	HMDB0000651	316.2475	5.361
Nifekalant	2.206E-7	4.665E-6	1.446	4.4104	2.1409	up	LevelBii	HMDB0255595	464.2128	3.0074
25-Hydroxyvitamin D3-26,23-Lactone	0.001482	0.003446	1.4476	3.4764	1.7976	up	LevelBii	HMDB0060126	451.2811	2.7262
Nordihydrocapsaicin	0.0009247	0.002347	1.4447	0.5109	-0.9688	down	LevelBi	HMDB0036328;LMFA08020319	294.2054	6.349
Alpha-Amino-3-Hydroxy-5-Methyl-4-Isoxazolepropionic Acid	5.976E-5	0.0002667	1.4401	2.518	1.3323	up	LevelBii	HMDB0248356	185.0557	0.7365
Phaseollinisoflavan	2.919E-5	0.000147	1.4437	0.5466	-0.8715	down	LevelBii	HMDB0034411	325.142	4.0206
Smgdg(O-16:3/6:0)	3.92E-6	3.847E-5	1.4316	2.6515	1.4068	up	LevelBi	-	695.337	4.0742
Sulfolithocholylglycine	0.001672	0.003802	1.4403	2.0931	1.0657	up	LevelBii	HMDB0002639	512.2671	2.2926
20,26-Dihydroxyecdysone	0.007751	0.01391	1.4594	2.1312	1.0917	up	LevelBii	HMDB0245570	555.3161	3.2619
Glu-Phe	0.001774	0.003996	1.4368	2.102	1.0718	up	LevelBi	HMDB0029156	295.1274	2.4
Gly-Trp	0.0001635	0.000603	1.4353	2.9685	1.5697	up	LevelBii	HMDB0028852	282.0852	2.3208
1-Nitro-5-Glutathionyl-6-Hydroxy-5,6-Dihydronaphthalene	1.822E-5	9.948E-5	1.4284	0.0958	-3.3841	down	LevelBii	HMDB0060326	531.0914	1.6973
Imidazolepropionic Acid	1.582E-7	3.646E-6	1.4323	2.5773	1.3659	up	LevelBii	HMDB0002271	185.0557	1.436
Cyclo(Trp-Asp-Pro-Ile-Leu)	0.002465	0.005248	1.4405	2.1951	1.1343	up	LevelBii	HMDB0250629	642.3557	4.3943
Amphibine H	0.001821	0.004083	1.4296	2.1114	1.0782	up	LevelBii	HMDB0029333	650.3139	2.0535
2-[4-(Dimethylamino)Phenyl]-1,2,3,4-Tetrahydroquinazolin-4-One	6.157E-6	4.001E-5	1.4345	0.4375	-1.1926	down	LevelBi	-	268.144	5.1367
Beta-D-Glucosaminyl-(1->4)-Beta-D-Glucosamine	1.96E-6	2.435E-5	1.429	0.4913	-1.0253	down	LevelBii	HMDB0249120	305.1332	1.1771
Aconitine	0.00437	0.008552	1.4929	2.7751	1.4725	up	LevelBi	-	646.3166	2.6069
Enkephalinamide-Leu, Ala(2)-	0.0001495	0.0005623	1.4172	1.803	0.8504	up	LevelBii	HMDB0244970	551.3006	3.5276
3,4-Dihydro-2H-1-Benzopyran-2-One	2.739E-6	3.107E-5	1.4271	0.2364	-2.0808	down	LevelBii	HMDB0036626	193.0494	3.9066
5-O-Desmethyldonepezil	0.001049	0.002591	1.4107	2.9839	1.5772	up	LevelBii	HMDB0013958	346.1786	3.2053
Lpc(O-18:3)	0.001792	0.00403	1.4044	0.5862	-0.7705	down	LevelBi	-	548.3331	7.978
Corchoroside A	1.827E-8	7.497E-7	1.427	38.64	5.272	up	LevelBii	HMDB0033846	593.2934	2.8497
3-O-Methylkaempferol	0.001576	0.00362	1.4278	0.2711	-1.883	down	LevelBii	HMDB0302564	299.0545	5.1673
Glycochenodeoxycholic Acid 3-Glucuronide	3.292E-7	6.245E-6	1.4187	2.2604	1.1766	up	LevelBii	HMDB0002579	624.3341	2.6988
Scillipheosidin 3-[Glucosyl-(1->2)-Rhamnoside]	6.402E-6	4.15E-5	1.4237	0.4018	-1.3155	down	LevelBii	HMDB0036946	743.3083	4.3522
Alpha-Hydroxymetoprolol	1.443E-5	8.225E-5	1.4321	0.5121	-0.9654	down	LevelBii	HMDB0060994	284.1846	3.123
Pi(5-Iso Pgf2Vi/22:6(4Z,7Z,10Z,13Z,16Z,19Z))	0.001308	0.003104	1.4281	2.6279	1.3939	up	LevelBii	HMDB0278358	987.4594	3.4518
Cys-Trp-Ala	1.645E-7	3.768E-6	1.4324	0.5243	-0.9315	down	LevelBii	HMDB0341149	437.1536	2.9509
Cer(8:0_30/14:1_(20h))	0.002845	0.005938	1.426	0.4515	-1.1472	down	LevelBi	-	384.3093	7.1967
Methyl Dl-Pyroglutamate	2.184E-7	4.645E-6	1.4208	1.97	0.9782	up	LevelBi	-	176.0905	1.6493
6-Hydroxypseudooxynicotine	2.009E-7	4.338E-6	1.4308	0.3653	-1.4529	down	LevelBii	HMDB0240264	253.1187	2.2353
Tyr-Glu	0.0012	0.00289	1.4254	1.6489	0.7215	up	LevelBi	HMDB0029104	311.1224	1.9124
Blumenol C O-[Rhamnosyl-(1->6)-Glucoside]	0.002704	0.005683	1.4299	2.3287	1.2195	up	LevelBii	HMDB0031935	483.2616	4.0498
Fa(18:3)	0.0003871	0.001147	1.4095	0.5912	-0.7583	down	LevelBi	-	555.4375	8.4549
Desmethyldeschlorobenzoyl Indomethacin	4.474E-6	3.847E-5	1.4158	0.175	-2.5145	down	LevelBi	-	190.0499	3.2431
Val-Ile-Leu	1.779E-5	9.735E-5	1.4076	1.4912	0.5765	up	LevelBi	-	344.253	3.4
Ethylestrenol	0.0003919	0.001157	1.4145	0.5537	-0.8528	down	LevelBii	HMDB0252076	271.2408	6.4371
Diisobutyl Cyclohexane-1,2-Dicarboxylate	4.129E-5	0.0001953	1.4029	1.5838	0.6633	up	LevelBi	-	348.2121	2.7064
Bioallethrin	2.525E-5	0.0001304	1.4097	1.9205	0.9415	up	LevelBii	HMDB0243551	366.2063	2.2806
Ile-Thr-Trp	0.0004928	0.001398	1.4018	1.9575	0.969	up	LevelBi	-	451.2517	2.3402
Nummularine A	0.00317	0.006513	1.4196	2.1318	1.0921	up	LevelBii	HMDB0029336	682.332	7.894
Dihydrogenistein	0.0008375	0.002164	1.4026	0.3873	-1.3685	down	LevelBii	HMDB0005897	271.0597	5.0474
(4E,15Z)-Bilirubin	1.852E-5	0.0001008	1.4043	6.9856	2.8044	up	LevelBii	HMDB0000488	605.2421	8.5195
Scabioside C	0.003804	0.00759	1.4067	1.8156	0.8604	up	LevelBi	-	789.443	4.1388
(7E,10E,13E,16E)-3-Hydroxydocosa-7,10,13,16-Tetraenoylcarnitine	0.0003828	0.001137	1.403	0.5096	-0.9725	down	LevelBii	HMDB0241607	546.3722	6.729
Candesartan Cilexetil	2.551	2.96E	1.411	3.164	1.662	up	Leve	HMDB0249581	609.2	3.273

Metabolite	P_value	FDR	VIP_PLS-DA	FC(A/E)	Log2FC (A/E)	Regulate	level	Library ID	M/Z	Retention time
	E-6	-5		5			1Bii		494	
Lpc(20:4-Sn1)	0.0006317	0.001714	1.4005	1.3442	0.4268	up	Level1Bi	-	544.3365	7.6294
Ibritumomab Tiuxetan	7.046E-5	0.0003048	1.4101	0.3479	-1.5233	down	Level1Bii	HMDB0253331	570.2407	4.6482
Pe(Pgf1Alpha/P-18:0)	0.0001375	0.0005266	1.4032	1.7054	0.7701	up	Level1Bii	HMDB0285371	858.5805	8.1635
16Alpha-Fluoroestradiol	1.29E-6	1.769E-5	1.394	0.4566	-1.1311	down	Level1Bii	HMDB0244757	325.1388	2.1105
Cyasterone	3.43E-6	3.692E-5	1.3903	1.5043	0.5891	up	Level1Bi	HMDB0250618;LMST01040153	521.3108	2.7064
Hetisine	0.00057	0.001573	1.3972	0.6318	-0.6626	down	Level1Bi	-	330.2035	6.7388
Chenodeoxycholylaspartic Acid	0.0001912	0.000681	1.388	1.6769	0.7458	up	Level1Bii	HMDB0242402	552.3135	2.3969
Glycerophosphocholine	8.645E-7	1.31E-5	1.3915	1.5776	0.6578	up	Level1Bi	HMDB0000086;PW_C000059	258.1092	0.6595
Oxytocin 1-8	9.306E-7	1.389E-5	1.3921	0.5615	-0.8327	down	Level1Bii	HMDB0013033	935.3714	4.9551
Alpha-Zearalenol	0.0002335	0.00078	1.3857	0.494	-1.0175	down	Level1Bii	HMDB0041824	362.1941	3.7845
Oleic Acid Methyl Ester	0.004843	0.009331	1.3897	0.5772	-0.7929	down	Level1Bi	HMDB0034451	297.2768	8.5625
Tetraethylene Glycol Monododecyl Ether	0.01062	0.01818	1.3967	0.333	-1.5866	down	Level1Bii	HMDB0246913	383.276	7.5643
Ile-Ala-Leu	5.109E-6	3.847E-5	1.3853	2.0649	1.046	up	Level1Bi	-	316.2226	3.686
2',6'-Dihydroxy-4'-Methoxyacetophenone	6.052E-6	3.945E-5	1.3828	4.5372	2.1818	up	Level1Bii	HMDB0029646	181.0497	4.8811
Cholic Acid Glucuronide	0.0002325	0.00078	1.3861	1.6403	0.714	up	Level1Bii	HMDB0002577	567.3211	7.7251
Lomefloxacin	5.119E-8	1.569E-6	1.3925	2.5898	1.3728	up	Level1Bi	HMDB0015113	369.1758	1.0985
Dopamine	1.886E-8	7.672E-7	1.3881	4.4908	2.167	up	Level1Bii	HMDB0000073	351.156	3.3671
N6-Methyl-2'-Deoxyadenosine	0.00025	0.000804	1.3835	2.1608	1.1115	up	Level1Bii	HMDB0255300	529.2276	3.1583
Cynaroside A	0.001144	0.002782	1.3899	2.1649	1.1143	up	Level1Bii	HMDB0040532	443.1887	1.5482
1-Linoleoyl-Sn-Glycero-3-Phosphorylcholine	0.001034	0.002561	1.3872	1.3074	0.3867	up	Level1Bi	HMDB0010386	520.3381	7.6582
Quercetin 3-(6''-Malonyl-Glucoside)	0.0001205	0.0004723	1.3776	2.221	1.1512	up	Level1Bii	HMDB0037368	531.0744	3.8321
S-(Pga1)-Glutathione	0.0008473	0.002185	1.3847	1.7444	0.8028	up	Level1Bii	HMDB0013061	664.2825	8.2165
Beta-Isorhodomycinone	0.0009578	0.002416	1.3875	2.8726	1.5224	up	Level1Bii	HMDB0249134	822.2999	3.9158
Lpc(18:3)	0.001056	0.002606	1.3829	1.512	0.5965	up	Level1Bi	HMDB0010387	562.313	7.294
8-(3-Oxo-2-(Pent-2-Enyl) Cyclopentenyl)Octanoic Acid	6.602E-5	0.0002891	1.3778	0.573	-0.8034	down	Level1Bi	-	293.2096	5.7323
Vinblastine	0.0005176	0.001456	1.3789	0.4367	-1.1952	down	Level1Bii	HMDB0014710	845.3841	5.0198
2-Hydroxyadipic Acid	0.0005123	0.001442	1.367	0.4967	-1.0096	down	Level1Bii	HMDB0000321	221.0657	2.0441
Phe-Gly	0.0001645	0.0006056	1.3774	1.5388	0.6218	up	Level1Bi	HMDB0028995	223.107	2.1616
Manninotriose	0.0007084	0.00188	1.3926	4.5422	2.1834	up	Level1Bi	HMDB0060468	487.1624	1.0985
Maltitol	0.001355	0.0032	1.3788	2.6508	1.4065	up	Level1Bi	HMDB0002928	343.128	2.207
Henatinib	1.372E-8	6.214E-7	1.3763	2.323	1.216	up	Level1Bii	HMDB0253087	513.2161	1.2774
Beta-D-Xylopyranosyl-(1->5)-Alpha-L-Arabinofuranosyl-(1->5)-L-Arabinose	2.667E-6	3.047E-5	1.3775	0.0141	-6.1481	down	Level1Bii	HMDB0038860	413.1287	2.0063
Epothilone C	7.657E-5	0.0003262	1.3723	1.6023	0.6802	up	Level1Bii	HMDB0251875	536.2702	2.6042
Penitrem B	3.958E-5	0.0001887	1.3635	11.472	3.52	up	Level1Bii	HMDB0256239	642.3448	3.4425
Tyr-Met	0.006273	0.01165	1.3915	1.7659	0.8204	up	Level1Bii	HMDB0029111	293.0952	4.0091
Virginiamycin M1	0.002321	0.004996	1.373	2.6102	1.3841	up	Level1Bii	HMDB0259836	560.2191	1.9687
4-Methyl-6,7,8,9-Tetrahydro-2H-Pyrano[3,2-G]Quinolin-2-One	7.833E-5	0.0003321	1.3697	1.666	0.7364	up	Level1Bi	-	216.101	3.3505
7-Methylinosine	1.091E-6	1.566E-5	1.375	0.5292	-0.9181	down	Level1Bii	HMDB0003950	342.1176	1.8648
Pe(O-16:4/4:0)	0.01135	0.01924	1.3842	1.5329	0.6163	up	Level1Bi	-	1001.5616	7.7115
Syringetin 3-Glucoside	0.001907	0.004241	1.3582	2.2789	1.1884	up	Level1Bi	LMPK12112488	507.1124	3.703
Arg-Pro	0.01218	0.02049	1.4047	1.9587	0.9699	up	Level1Bi	HMDB0028717	272.1705	1.1086
Prednisolone Valerate Acetate	1.188E-5	6.987E-5	1.3609	3.3406	1.7401	up	Level1Bii	HMDB0256750	451.2512	3.4686
N-Oleoyl Glutamic Acid	7.294E-5	0.0003134	1.3744	0.1924	-2.3781	down	Level1Bii	HMDB0241959	392.279	8.198
Cholylthreonine	0.003645	0.007327	1.366	1.6545	0.7264	up	Level1Bii	HMDB0242443	530.3109	8.2071
(2Z,4'Z)-2-(5-Methylthio-4-Penten-2-Ynylidene)-1,6-Dioxaspiro[4.4]Non-3-Ene	2.565E-6	2.971E-5	1.37	0.4493	-1.1543	down	Level1Bii	HMDB0032660	234.0717	3.0238
Hovenoside D	0.0004999	0.001415	1.348	3.5986	1.8474	up	Level1Bii	HMDB0035746	1191.5889	2.9979
5-(N-Hexadecanoyl)Aminofluorescin	2.783E-6	3.133E-5	1.3621	1.9283	0.9473	up	Level1Bii	HMDB0246734	550.2938	2.9348
Styrene	2.584E-6	2.983E-5	1.3553	0.719	-0.476	down	Level1Bi	HMDB0034240	105.0693	2.1616
Lpc(O-20:2)	6.792E-5	0.0002956	1.3555	0.4158	-1.2661	down	Level1Bi	-	578.3781	8.5195
Ile-Val-Asp	3.488E-5	0.0001701	1.3571	0.4942	-1.0169	down	Level1Bi	-	346.1965	5.029
Trp-Gln-Tyr	0.00647	0.01195	1.3616	2.1027	1.0722	up	Level1Bi	-	460.2018	1.2065
(2-Anilino-4-Methyl-1,3-Thiazol-5-Yl)(1-Benzothiophen-2-Yl)Methanone	5.361E-7	9.127E-6	1.3642	0.3821	-1.3878	down	Level1Bi	-	389.0175	0.5895

Metabolite	P_value	FDR	VIP_PLS-DA	FC(A/E)	Log2FC (A/E)	Regulate	level	Library ID	M/Z	Retention time
Amobarbital	3.777E-8	1.272E-6	1.3579	0.5976	-0.7426	down	LevelBii	HMDB0015440	227.1379	2.9249
Phe-Ile-Phe	0.002122	0.004631	1.3513	1.8143	0.8594	up	LevelBi	-	426.2365	4.3746
Ile-Pro-Trp	0.01293	0.02156	1.3846	1.8716	0.9042	up	LevelBi	-	415.2319	3.7056
P-Tolyl Sulfate	4.107E-5	0.0001945	1.3505	2.2109	1.1446	up	LevelBi	-	187.006	3.6936
Vignatic Acid B	0.001626	0.003717	1.3555	1.8445	0.8832	up	LevelBii	HMDB0033617	574.3078	3.133
Cyclo(Pro-Phe-Trp-Lys-Thr-Phe)	0.01361	0.02253	1.3725	1.6326	0.7071	up	LevelBii	HMDB0250003	787.3943	2.1593
Octadec-9-Enedioic Acid	0.0006572	0.001769	1.3498	0.6404	-0.6429	down	LevelBi	-	313.2361	6.641
Testosterone Glucuronide	8.789E-5	0.0003635	1.3398	1.6803	0.7487	up	LevelBii	HMDB0003193	465.2439	2.9447
N-[[3-Hydroxy-2-(2-Pentenyl)Cyclopentyl]Acetyl]Isoleucine	0.0002804	0.000883	1.3469	1.8282	0.8704	up	LevelBii	HMDB0041248	360.1927	2.979
(2S)-4-Amino-2-[[(2R)-2-Amino-4-Methylsulfanylbutanoyl]Amino]-4-Oxobutanoic Acid	0.0001211	0.0004747	1.3487	2.1991	1.1369	up	LevelBi	-	264.0997	1.0886
Hetacillin	8.679E-6	5.345E-5	1.3467	0.5267	-0.9249	down	LevelBii	HMDB0014877	388.135	3.8134
N-Fructosyl Isoleucine	6.712E-8	1.904E-6	1.3553	0.7018	-0.5108	down	LevelBi	-	294.1541	1.9424
Ala-Tyr-Leu	5.369E-6	3.847E-5	1.3402	1.6125	0.6893	up	LevelBi	-	366.2019	2.6567
Phe-Ser	0.0002505	0.000805	1.347	1.5144	0.5987	up	LevelBi	HMDB0029004	253.1186	2.0812
(2E)-3-(1H-Indol-2-Yl)-2-Propenoic Acid	0.001148	0.00279	1.366	0.5124	-0.9646	down	LevelBi	-	188.0695	3.2023
3-Chloro-N-(4-Imidazo[1,2-A]Pyrimidin-2-Ylphenyl)Benzamide	8.147E-7	1.253E-5	1.3576	0.3786	-1.4012	down	LevelBi	-	349.0883	2.7562
2-Phenylacetamide	2.244E-5	0.0001182	1.3392	1.4753	0.5611	up	LevelBi	HMDB0010715	136.075	1.8436
Angiotensin Iii	0.002708	0.005689	1.353	2.678	1.4212	up	LevelBii	HMDB0001036	989.5287	4.4538
Cis-3-Hexenyl Tiglate	0.001886	0.004201	1.3348	0.49	-1.0291	down	LevelBi	HMDB0038279;LMFA07010808	183.1367	7.7446
Ps(Pgf2Alpha/16:1(9Z))	0.0007336	0.001938	1.335	1.71	0.774	up	LevelBii	HMDB0281245	814.485	7.6392
Lpc(20:3-Sn1)	0.000579	0.001594	1.3365	1.3503	0.4333	up	LevelBi	-	546.3534	7.8512
Verdamicin	0.009156	0.01603	1.3321	1.6707	0.7404	up	LevelBii	HMDB0259789	444.2786	2.6069
Dolichodial	3.97E-5	0.0001891	1.3274	0.3796	-1.3975	down	LevelBii	HMDB0251578	225.1119	5.3238
Pi(Pgf2Alpha/20:0)	0.0008366	0.002162	1.3555	2.1421	1.099	up	LevelBii	HMDB0276994	997.6175	8.1832
Avenic Acid A	0.0001906	0.0006799	1.3643	0.3571	-1.4855	down	LevelBii	HMDB0030416	357.1049	2.4066
Agavasaponin C	0.00146	0.003403	1.3378	0.5892	-0.7633	down	LevelBii	HMDB0033353	917.4671	6.9051
Ser-Tyr	0.01782	0.02846	1.3706	2.5305	1.3394	up	LevelBi	HMDB0258243	269.1109	2.0414
Ala-Trp	0.0008804	0.002255	1.339	1.5548	0.6367	up	LevelBi	HMDB0013209	276.1332	2.6467
Vomifoliol	0.0008991	0.002292	1.3346	2.4071	1.2673	up	LevelBii	HMDB0303570	259.1085	4.0277
Ethyl 4-Isothiocyanatopiperidine-1-Carboxylate	0.0002615	0.0008328	1.346	0.3201	-1.6433	down	LevelBi	-	237.0683	0.5895
Cholylcitrulline	0.01934	0.03055	1.3805	1.8331	0.8743	up	LevelBii	HMDB0242383	610.3703	8.1796
Neoeriocitrin	2.777E-7	5.511E-6	1.3326	0.3817	-1.3895	down	LevelBii	HMDB0255521	577.1529	3.5082
Pc(Pgf1Alpha/18:1(9Z))	0.01504	0.02457	1.3687	1.7601	0.8156	up	LevelBii	HMDB0286512	840.5753	7.7297
Subaphylline	0.002962	0.006143	1.3379	1.5779	0.658	up	LevelBii	HMDB0033463	527.2849	2.6799
Tri-N-Acetylchitotriose	0.01346	0.02232	1.2977	0.3279	-1.6085	down	LevelBii	HMDB0006698	648.2239	2.416
16-Deacetylgeyerline	0.001006	0.002505	1.3356	1.9203	0.9413	up	LevelBii	HMDB0244720	667.3282	4.4446
(5Alpha,6Beta,14Alpha,20R,22R)-5,6,14,20,27-Pentahydroxy-1-Oxowith-24-Enolide	0.0001906	0.0006799	1.3186	1.6231	0.6988	up	LevelBii	HMDB0033198	471.2786	3.5177
4-Pregnen-17Alpha, 20Alpha-Diol-3-One	0.02987	0.04444	1.3124	0.0032	-8.3066	down	LevelBi	HMDB0011653	333.2439	7.9885
Tyr-Lys	0.0006578	0.00177	1.3249	1.604	0.6816	up	LevelBi	HMDB0029110	310.1754	1.0591
Ile-Asn-Ile	1.566E-6	2.038E-5	1.3169	1.3892	0.4743	up	LevelBi	-	359.2275	3.3012
Ganoderic Acid X	0.01267	0.02118	1.3644	2.2244	1.1534	up	LevelBii	HMDB0035332	554.3876	4.1978
Huperzine A	0.0004935	0.001399	1.3188	1.9458	0.9604	up	LevelBii	HMDB0242212	243.151	2.0017
Stearic Acid Ethyl Ester	1.009E-6	1.481E-5	1.3314	0.5231	-0.9349	down	LevelBi	HMDB0034156	311.2941	7.8296
Thr-Met	0.003486	0.00706	1.3368	2.0173	1.0125	up	LevelBii	HMDB0029067	249.0909	1.9214
(11E,15Z)-9,10,13-Trihydroxy-11,15-Octadecadienoic Acid	1.063E-5	6.348E-5	1.318	0.7084	-0.4974	down	LevelBi	-	327.2161	5.7291
Methylpyrazine	2.375E-9	2.03E-7	1.3225	0.6408	-0.642	down	LevelBii	HMDB0033112	95.06	0.6298
Lucidenic Acid A	0.004499	0.008759	1.3412	2.1308	1.0914	up	LevelBii	HMDB0037611	463.2489	2.35
Gly-Phe-Leu-Gly	4.7E-6	3.847E-5	1.3144	2.0361	1.0258	up	LevelBii	HMDB0252827	373.1856	3.8507
2-Deoxy-2,3-Dehydro-N-Acetyl-Neuraminic Acid	1.081E-6	1.558E-5	1.3195	0.6424	-0.6385	down	LevelBi	HMDB0245680	314.0825	0.7467
Deoxyloganic Acid	5.326E-6	3.847E-5	1.3144	0.3451	-1.5348	down	LevelBii	HMDB0037028	359.133	3.9531
Pro-Tyr-Ile	0.002112	0.004614	1.3193	1.6255	0.7009	up	LevelBi	-	392.2156	3.0537
N-(1-Deoxy-1-Fructosyl)Leucine	0.001173	0.002837	1.3118	2.2495	1.1696	up	LevelBii	HMDB0037840	292.1396	1.1283
(2E,4E)-3,5,7-Trimethylocta-2,4-Dienedioic Acid	0.000	0.000	1.308	0.486	-1.038	down	Leve	-	195.0	3.6661

Metabolite	P_value	FDR	VIP_PLS-DA	FC(A/E)	Log2FC(A/E)	Regulate	level	Library ID	M/Z	Retention time
	1242	4843		8	7		lBi		998	
Pgp(Lte4/I-16:0)	0.000533	0.00149	1.3135	0.5626	-0.8299	down	LevelBii	HMDB0275235	984.4618	5.7845
Physangulide	0.000374	0.001116	1.3132	2.3119	1.2091	up	LevelBii	HMDB0037381	543.2534	6.4896
4'-Hydroxycarvedilol	5.595E-8	1.657E-6	1.3129	0.5837	-0.7767	down	LevelBii	HMDB0013947	403.1696	2.5571
Asp-His	0.0005119	0.001442	1.3093	0.5777	-0.7915	down	LevelBi	HMDB0028755	325.111	4.3449
Tyrphostin B42	0.006112	0.01139	1.3562	0.1728	-2.5332	down	LevelBi	HMDB0244549	312.1325	4.8049
1-Acetyl-3,27-Dihydroxywitha-5,24-Dienolide 3-Glucoside	0.005945	0.01112	1.3173	2.1821	1.1257	up	LevelBii	HMDB0033729	707.3707	2.416
1-Myristoyl-Sn-Glycero-3-Phosphocholine	0.0007656	0.002006	1.3036	1.4748	0.5606	up	LevelBi	HMDB0010379	468.3063	7.4429
Lnape(13:1/N-7:0)	0.005973	0.01116	1.3257	1.9151	0.9374	up	LevelBi	-	580.3235	6.4803
Deoxynivalenol 3-Glucoside	0.000294	0.0009178	1.2969	1.9237	0.9439	up	LevelBii	HMDB0039852	476.2132	3.0437
1-(1Z-Octadecenyl)-Sn-Glycero-3-Phosphocholine	0.001157	0.002808	1.2957	0.7403	-0.4338	down	LevelBi	LMGP01070009	508.3724	8.8135
3-Amino-2-Hydroxyheptanoic Acid	0.001614	0.003692	1.3107	1.8728	0.9052	up	LevelBi	-	194.1401	1.9026
Pyrodone	1.015E-6	1.485E-5	1.3044	0.4925	-1.0219	down	LevelBii	HMDB0256988	320.1844	4.7335
5-Hexyldihydro-2(3H)-Furanone	0.001345	0.003181	1.2965	0.6262	-0.6753	down	LevelBi	HMDB0037217	171.1369	8.1247
Monocaprin	0.01842	0.02929	1.3533	2.1138	1.0798	up	LevelBi	-	229.179	6.7095
Lpc(20:5)	0.007132	0.01297	1.325	1.6329	0.7074	up	LevelBi	HMDB0010397	586.3126	7.2754
Erythro-L-Galacto-Nonulose	0.0008507	0.002194	1.3082	0.5441	-0.8779	down	LevelBii	HMDB0029955	275.0723	0.776
Phe-Val-Phe	0.002347	0.005041	1.2962	1.6728	0.7423	up	LevelBi	-	412.2207	4.0303
Cyclo(Arg-Gly-Asp-Phe-Val)	2.406E-5	0.0001255	1.2936	2.3698	1.2447	up	LevelBii	HMDB0250680	595.2606	3.2919
Pro-Gln	0.0008196	0.002122	1.2998	2.4566	1.2967	up	LevelBii	HMDB0029015	288.1186	1.846
Neriifolin	2.018E-5	0.0001085	1.2863	2.4338	1.2832	up	LevelBi	LMST01120021	569.2919	3.1583
N-Docosahexaenoyl Phenylalanine	0.006456	0.01193	1.2905	1.6432	0.7165	up	LevelBii	HMDB0062333	440.2949	2.557
Anapear	0.0002892	0.0009056	1.2872	0.4987	-1.0036	down	LevelBii	HMDB0040285	155.1059	6.0938
Tuliposide B	5.234E-6	3.847E-5	1.2826	1.75	0.8074	up	LevelBii	HMDB0303141	315.0702	2.5665
Lpe(22:5)	0.0001259	0.000489	1.2828	1.327	0.4082	up	LevelBi	HMDB0011494	526.2917	7.8848
Posatirelin	0.001434	0.003352	1.2937	1.5843	0.6639	up	LevelBii	HMDB0256710	385.2421	2.3402
N-Feruloylglycyl-L-Phenylalanine	8.477E-7	1.29E-5	1.2896	0.5825	-0.7796	down	LevelBii	HMDB0029356	397.1437	2.5853
Tryptoline	0.001329	0.003145	1.2716	0.5643	-0.8254	down	LevelBi	HMDB0012488	173.1065	2.8555
Gallopamil	0.00111	0.002712	1.2859	2.9364	1.554	up	LevelBii	HMDB0252599	529.2887	7.443
2',3',4'-Trihydroxyacetophenone	1.573E-8	6.724E-7	1.2882	0.5795	-0.7872	down	LevelBi	-	167.034	2.4348
Strobilurin	2.43E-5	0.0001265	1.2876	0.4708	-1.0868	down	LevelBii	HMDB0258515	441.1953	3.9344
Gonane	0.001198	0.002887	1.3009	2.2817	1.1901	up	LevelBii	HMDB0252914	233.2259	7.2066
Ile-Ile-Ile-Pro	0.01075	0.01836	1.2957	1.7935	0.8428	up	LevelBii	HMDB0304800	455.3204	3.843
Milbemycin Beta3	7.098E-6	4.52E-5	1.2788	1.6024	0.6803	up	LevelBii	HMDB0254729	515.2805	2.3208
1-[4-(4-Chlorophenyl)Piperazin-1-Yl]-N-(3-Methylbutyl)Isoquinoline-7-Carboxamide	0.002326	0.005005	1.2923	1.678	0.7468	up	LevelBi	-	437.2126	2.2706
20-Hydroxy-Leukotriene B4	8.247E-6	5.122E-5	1.2756	42.8044	5.4197	up	LevelBii	HMDB0001509	397.2214	4.2411
10,11-Dihydro-12R-Hydroxy-Leukotriene C4	0.0004232	0.001234	1.2793	1.8824	0.9126	up	LevelBii	HMDB0060155	702.3246	2.4348
Fumonisin B1	0.003541	0.007152	1.2921	2.4625	1.3001	up	LevelBii	HMDB0034702	722.3993	4.4138
Phe-Leu-Ile	0.0001434	0.0005443	1.2703	1.4807	0.5662	up	LevelBi	-	392.2519	4.1879
6-(1-Naphthyl)-5-Nitro-2-Piperidinone	0.0005152	0.00145	1.2945	0.588	-0.7661	down	LevelBi	-	325.1183	3.4686
Capsianoside Iv	0.001532	0.003542	1.2699	1.9131	0.9359	up	LevelBii	HMDB0030736	627.3411	3.0142
Glu-Leu-Leu	0.01339	0.02221	1.299	1.6642	0.7348	up	LevelBi	-	374.2258	3.4295
Phe-Val-Leu	0.0002944	0.0009187	1.2693	1.529	0.6126	up	LevelBi	-	378.2366	3.9713
Coenzyme Q9	1.011E-5	6.077E-5	1.2707	1.8113	0.857	up	LevelBii	HMDB0006707	827.648	8.8233
Pyroglutamylleucine	0.01585	0.02572	1.311	2.1708	1.1182	up	LevelBii	HMDB0341382	241.1185	2.2165
17-Aminogeldanamycin	0.001923	0.004269	1.2751	1.9903	0.993	up	LevelBii	HMDB0259661	563.3062	3.3012
Nordihydroguaiaretic Acid	5.516E-5	0.0002497	1.3035	0.4221	-1.2445	down	LevelBi	HMDB0014325	341.1146	3.3603
Ala-Gly-Leu-Val-Ser	0.0005592	0.001549	1.2687	2.9853	1.5779	up	LevelBi	-	444.2446	3.273
Core Oligosaccharide	1.356E-6	1.835E-5	1.2758	0.4396	-1.1858	down	LevelBii	HMDB0250452	755.2449	1.1003
Imigliptin	0.002517	0.005343	1.2652	1.6774	0.7462	up	LevelBii	HMDB0253428	341.191	2.2211
Ethyl 2-Methyl-2-Phenylpropanoate	5.838E-5	0.0002619	1.2678	2.0291	1.0209	up	LevelBi	-	175.1108	6.2803
Lucyoside K	0.01417	0.02334	1.295	2.2784	1.188	up	LevelBii	HMDB0041353	613.3731	7.3685
Phe-Val	0.003617	0.00728	1.2781	1.6486	0.7212	up	LevelBi	HMDB0029008	529.2982	2.6767

Metabolite	P_value	FDR	VIP_PLS-DA	FC(A/E)	Log2FC (A/E)	Regulate	level	Library ID	M/Z	Retention time
Tyr-Ala	0.0008048	0.002088	1.2727	1.5589	0.6405	up	LevelBi	HMDB0029098	253.1169	1.9323
N-Formyl-Met-Leu-Phe-Lys	7.7E-7	1.198E-5	1.2638	1.6232	0.6988	up	LevelBii	HMDB0252432	600.2638	3.2431
1,4-Dihydro-3(2H)-Isoquinolinone	0.0009355	0.00237	1.2537	2.0466	1.0332	up	LevelBi	-	130.0642	2.4
Val-Ile-Val	0.000194	0.0006882	1.2661	1.6358	0.71	up	LevelBi	-	330.2368	2.8357
Deacetylchloronectrin	5.366E-8	1.613E-6	1.2707	0.7057	-0.5029	down	LevelBi	-	423.1967	2.0512
Tyr-Arg	0.001454	0.003392	1.274	1.5347	0.618	up	LevelBi	HMDB0005768	338.1808	1.2161
3-Cyclopentylalanine	6.433E-5	0.0002826	1.2626	1.6095	0.6866	up	LevelBi	-	199.1431	3.321
Aspartame	0.004652	0.009012	1.2868	1.5927	0.6715	up	LevelBii	HMDB0001894	293.113	2.1593
Pg(Pgf1Alpha/16:0)	0.01461	0.02395	1.2909	1.5216	0.6056	up	LevelBii	HMDB0268652	840.5676	7.7639
Gly-Phe	0.0003147	0.0009686	1.2686	1.5054	0.5902	up	LevelBi	HMDB0028848	223.1071	2.3204
16,17-Dihydro-16Alpha,17-Dihydroxygibberellin A4 17-Glucoside	1.425E-5	8.14E-5	1.2575	3.2605	1.7051	up	LevelBii	HMDB0040647	527.2094	1.9876
1-Phenyl-2,4-Pentadiyn-1-One	9.751E-5	0.0003956	1.2553	3.8083	1.9292	up	LevelBii	HMDB0032945	353.0794	2.9979
Leu-Ile-Leu	4.469E-5	0.0002086	1.2554	1.4163	0.5022	up	LevelBi	-	358.2682	3.912
Dn-Isobutylamide	3.674E-5	0.0001776	1.2547	1.6474	0.7202	up	LevelBii	HMDB0251551	401.2259	2.6369
Haloperidol	1.778E-5	9.735E-5	1.2701	1.9117	0.9349	up	LevelBii	HMDB0014645	420.1387	2.5476
Lobucavir	0.001465	0.003414	1.3039	0.1242	-3.0096	down	LevelBii	HMDB0254142	310.1182	4.7981
Licoricesaponin J2	0.0003054	0.0009457	1.2879	2.0285	1.0204	up	LevelBi	HMDB0039448	825.4205	3.0833
Asp-Gly-Leu	5.475E-6	3.847E-5	1.2588	0.6395	-0.6449	down	LevelBi	-	304.1478	2.5069
Ethyl L-Tryptophanate	2.375E-6	2.789E-5	1.26	1.5124	0.5968	up	LevelBi	HMDB0252047	233.1269	3.3505
1H-Indole-4-Carboxaldehyde	0.001641	0.003742	1.2634	1.5116	0.5961	up	LevelBi	-	146.0596	2.3901
Methyl Benzoylformate	3.688E-5	0.0001781	1.2543	1.4732	0.559	up	LevelBi	HMDB0062605	165.0538	0.7856
Heptanohydrazide	0.000254	0.0008129	1.2504	1.6718	0.7414	up	LevelBi	-	199.143	3.133
Mibefradil	0.001936	0.004293	1.2597	1.3891	0.4742	up	LevelBii	HMDB0254702	500.2718	7.8512
Paromomycin I	0.01586	0.02574	1.2918	1.6561	0.7278	up	LevelBii	HMDB0242512	633.3348	4.5797
6-Guanidino-2-Oxocaproic Acid	2.558E-8	9.694E-7	1.2538	3.8663	1.951	up	LevelBii	HMDB0247067	373.1872	2.7365
Dodec-2-Enedioic Acid	1.441E-5	8.216E-5	1.2576	0.5661	-0.8209	down	LevelBii	HMDB0340663	246.1693	5.5564
Asn-Trp	0.000288	0.0009029	1.2549	1.4727	0.5584	up	LevelBi	HMDB0028742	319.1395	2.6069
1-Dodecyl-3-Phenyl-2-Thiourea	0.0007975	0.002073	1.2378	0.703	-0.5084	down	LevelBi	-	343.2189	7.7349
Inuline	0.0004408	0.001273	1.2474	1.6734	0.7428	up	LevelBii	HMDB0248466	585.3231	4.3337
beta-Asp-Leu	0.005475	0.01037	1.2661	1.7275	0.7887	up	LevelBii	HMDB0011166	245.1136	2.2638
Lucidenic Acid E2	4.736E-5	0.0002194	1.2422	1.4593	0.5453	up	LevelBi	HMDB0036434	517.2746	2.6865
O-Oxalylhomoserine	5.162E-6	3.847E-5	1.265	0.5163	-0.9537	down	LevelBii	HMDB0302143	250.0563	1.9309
Tyrosine, Ethyl Ester	2.758E-6	3.124E-5	1.2489	1.4871	0.5725	up	LevelBi	-	210.1116	2.3402
(2R)-1-(2-Hydroxyethyl)-2,5,5,8A-Tetramethyldecahydronaphthalen-2-Ol	0.001294	0.003077	1.25	0.6873	-0.541	down	LevelBi	-	277.2154	6.641
7,8-Dihydro-3B,6A-Dihydroxy-Alpha-Ionol 9-[Apiosyl-(1->6)-Glucoside]	2.574E-10	6.061E-8	1.2522	1.559	0.6406	up	LevelBii	HMDB0041579	540.2995	2.2706
N-Tryptophylglycine	0.001431	0.003347	1.2556	1.4734	0.5592	up	LevelBi	-	262.1174	2.3104
2-(4-Chlorophenyl)-1-[3-(3-Cyclopropyl-1,2,4-Triazol-4-Yl)Azetidin-1-Yl]Ethanone	4.329E-5	0.0002027	1.2437	0.6015	-0.7333	down	LevelBi	-	317.1194	3.0735
Myotoxin A	0.0005223	0.001467	1.2492	1.6807	0.7491	up	LevelBii	HMDB0254743	531.2578	2.6567
Lauric Acid	0.001047	0.002587	1.2307	0.5338	-0.9056	down	LevelBi	HMDB0000638;LMFA01010012	201.1841	8.0084
N-Acetyl-L-Phenylalanine	1.367E-5	7.866E-5	1.252	0.6689	-0.5801	down	LevelBii	HMDB0000512	206.0807	3.7311
Abscisic Alcohol 11-Glucoside	2.455E-6	2.862E-5	1.2391	1.4592	0.5452	up	LevelBii	HMDB0039636	476.2244	2.1616
Glucosyl (2E,6E,10X)-10,11-Dihydroxy-2,6-Farnesadienoate	0.004061	0.008023	1.2546	1.857	0.893	up	LevelBii	HMDB0037823	437.2159	2.4872
Pyroglutamylisoleucine	2.828E-5	0.0001429	1.2414	1.4267	0.5126	up	LevelBii	HMDB0341381	241.1185	3.273
Leu-Phe-Asp	0.001676	0.003809	1.2468	1.6555	0.7273	up	LevelBi	-	394.1968	2.905
Cucurbitacin S	0.000363	0.001088	1.2428	0.5724	-0.8049	down	LevelBii	HMDB0250583	537.2566	5.9078
Butaclamol	0.0008174	0.002117	1.2453	1.7388	0.7981	up	LevelBii	HMDB0242247	396.2102	3.1301
Fluorescin	4.228E-5	0.0001989	1.2545	15.5391	3.9578	up	LevelBii	HMDB0252365	393.1011	1.9876
Atorvastatin	0.003554	0.007172	1.2556	0.4239	-1.2381	down	LevelBii	HMDB0005006	539.232	4.1298
Pe(O-16:2/2:0)	0.02542	0.03869	1.2528	1.5387	0.6217	up	LevelBi	-	953.564	7.7388
Metildigoxin	5.313E-5	0.000242	1.2269	1.8501	0.8876	up	LevelBii	HMDB0254674	815.4232	2.7555
Lpe(18:3)	0.002202	0.00478	1.2277	1.4952	0.5804	up	LevelBi	HMDB0011478	474.2603	7.4709
N-((4-(Propan-2-Yl)Cyclohexyl)Carbonyl)Phenylalanine	7.979E-5	0.0003368	1.2355	2.1819	1.1256	up	LevelBi	-	356.165	2.6369
Pgp(Txb2/A-13:0)	0.000	0.000	1.241	1.531	0.6149	up	Leve	HMDB0274305	855.4	3.0922

Metabolite	P_value	FDR	VIP_PLS-DA	FC(A/E)	Log2FC (A/E)	Regulate	level	Library ID	M/Z	Retention time
	1348	5178	8	4			lBii		096	
Tyr-Tyr	0.0002327	0.00078	1.2318	1.6121	0.6889	up	LevelBi	HMDB0029117	345.1426	2.2012
1-[2-[1-(Benzenesulfonyl)Piperidin-4-Yl]Ethyl]-3-Propan-2-Ylurea	8.318E-6	5.158E-5	1.2328	1.5206	0.6046	up	LevelBi	-	354.1872	2.3104
Glu-Trp	0.001046	0.002587	1.2396	1.6022	0.6801	up	LevelBii	HMDB0028830	332.1244	2.2353
Ciprostene	1.055E-5	6.306E-5	1.2299	0.6536	-0.6135	down	LevelBii	HMDB0250270	399.2332	6.0621
Leu-Leu-Gly	0.002541	0.005381	1.2234	1.5713	0.6519	up	LevelBi	-	302.2059	2.4971
Gymnodimine	0.003533	0.007139	1.217	0.5767	-0.7942	down	LevelBii	HMDB0041430	488.3125	8.5476
Thr-Lys	1.016E-6	1.485E-5	1.2361	0.6841	-0.5478	down	LevelBii	HMDB0029066	270.1432	1.9821
Lpc(22:6)	0.003047	0.006291	1.2395	1.4597	0.5457	up	LevelBi	HMDB0010404	612.3286	7.5551
Lpc(20:1)	6.094E-6	3.965E-5	1.2296	1.3851	0.47	up	LevelBi	HMDB0010391	594.3749	8.5476
N1-(2-Hexanoyl-4,5-Dimethylphenyl)Acetamide	0.021	0.0328	1.2381	1.9605	0.9712	up	LevelBi	-	325.19	3.0042
Lpc(17:1)	0.0008292	0.002145	1.23	1.5976	0.6759	up	LevelBi	LMGP01050002	552.3282	7.8848
Dlcl(18:2/22:7)	0.00158	0.003628	1.2353	1.5937	0.6724	up	LevelBi	-	991.4699	3.1865
3,10-Dihydroxydodecanoylcarnitine	0.001009	0.002511	1.2348	0.6073	-0.7196	down	LevelBii	HMDB0241292	417.2966	6.4371
5-Methoxysalicylic Acid	0.001064	0.002623	1.2511	0.5254	-0.9284	down	LevelBi	HMDB0001868	167.0337	3.1018
Diprotin A	4.164E-5	0.0001967	1.2222	1.4805	0.566	up	LevelBi	HMDB0251473	340.2227	3.3296
Lpe(20:2)	0.0006652	0.001786	1.2243	1.3688	0.4529	up	LevelBi	HMDB0011483	504.3072	8.2539
(9Z)-5,8,11-Trihydroxyoctadec-9-Enoic Acid	0.0001856	0.0006665	1.2268	0.6153	-0.7006	down	LevelBi	-	331.246	5.6542
Leu-Leu-Lys	0.03842	0.05561	1.2337	1.6558	0.7275	up	LevelBi	-	373.2793	2.4197
Pleconaril	0.002819	0.00589	1.2362	1.8829	0.913	up	LevelBii	HMDB0256637	402.1083	3.1018
Biotin-Xx Hydrazide	0.001725	0.003901	1.216	0.6928	-0.5295	down	LevelBi	-	523.2417	8.1247
3-O-Acetylepisamarcandin	4.877E-5	0.0002248	1.223	1.6529	0.725	up	LevelBi	HMDB0031958	497.2532	2.9348
Dolichosterone	0.00321	0.00658	1.2217	1.8283	0.8705	up	LevelBii	HMDB0034336	497.3074	8.6411
Leustroducsin B	0.002608	0.00551	1.2356	1.8248	0.8678	up	LevelBii	HMDB0254044	668.3609	3.0639
Furosine	0.01116	0.01898	1.2597	1.8211	0.8648	up	LevelBii	HMDB0029390	259.1039	2.3104
Urolithin C 3-Glucuronide	1.91E-9	1.765E-7	1.2225	0.3955	-1.3384	down	LevelBii	HMDB0142405	441.0449	0.5569
Bidisomide	0.0001143	0.000452	1.2178	1.6791	0.7476	up	LevelBii	HMDB0249194	408.2435	3.745
Phe-Asp-Ile	0.003733	0.007472	1.2257	1.5303	0.6138	up	LevelBi	-	394.1949	3.735
Pip(Pgf2Alpha/16:2(9Z,12Z))	0.0004993	0.001414	1.2164	0.6408	-0.6421	down	LevelBii	HMDB0278654	1029.4502	6.896
Clarithromycin	2.041E-6	2.505E-5	1.2239	1.3939	0.4791	up	LevelBii	HMDB0015342	802.4858	7.6582
Licarin C	0.01261	0.0211	1.2282	1.9842	0.9886	up	LevelBii	HMDB0033319	369.174	1.3148
N,N-Di-N-Propyldopamine	3.778E-6	3.847E-5	1.2127	1.6245	0.7	up	LevelBii	HMDB0255249	296.1855	3.7766
Met-Trp	0.002327	0.005005	1.2227	1.4364	0.5225	up	LevelBi	HMDB0028984	336.1363	3.1132
Lpe(22:4)	0.0003564	0.001073	1.2124	1.2566	0.3296	up	LevelBi	HMDB0011493	528.307	8.1252
Crustecdysone	0.003636	0.007313	1.2267	1.6155	0.692	up	LevelBii	HMDB0030180	481.3159	7.7154
Gln-Arg-Thr	0.0005002	0.001415	1.2208	0.6414	-0.6407	down	LevelBi	-	445.2503	6.035
Sibrafiban	0.002155	0.004692	1.2228	1.8363	0.8768	up	LevelBii	HMDB0258287	465.1973	2.1105
Muridienin 4	0.03004	0.04468	1.2405	0.5216	-0.9389	down	LevelBii	HMDB0031906	577.4807	9.5225
3'-N-Acetyl-4'-O-(10,12-Octadecadienoyl)Fusarochromanone	2.106E-5	0.0001122	1.207	1.774	0.827	up	LevelBii	HMDB0038566	641.3865	3.5082
Exaprolol	0.0004975	0.00141	1.2012	1.6665	0.7368	up	LevelBii	HMDB0252141	314.2063	2.587
9-((2-Hydroxyethoxy)Methyl)Hypoxanthine	1.704E-5	9.409E-5	1.218	0.7121	-0.4898	down	LevelBi	-	211.0819	1.9821
(2E,4E)-N-(1-Hydroxy-3-Methylpentan-2-Yl)-2-Methylhexa-2,4-Dienamide	0.02734	0.04119	1.2616	2.9524	1.5619	up	LevelBi	-	208.169	5.6249
Tyr-Gly	0.0001157	0.0004562	1.2077	1.427	0.513	up	LevelBi	HMDB0259337	239.1013	1.9424
Lpe(22:3)	0.002473	0.005262	1.2035	1.5504	0.6327	up	LevelBi	-	530.3209	8.409
Lpc(18:1)	0.0009316	0.002361	1.212	1.2631	0.337	up	LevelBi	HMDB0002815	566.3439	8.0526
(5Alpha,17Beta)-17-Benzoyl-4-Azaandrost-1-En-3-One	0.003348	0.006824	1.2135	2.0832	1.0588	up	LevelBii	HMDB0253725	412.2048	3.4141
Convallatoxin	0.001335	0.003159	1.2088	1.4932	0.5784	up	LevelBi	HMDB0034362;LMST01120013	551.2821	2.6865
Lpe(14:0)	0.007998	0.01427	1.2061	1.8714	0.9041	up	LevelBi	HMDB0011470	424.2451	7.5177
Lpc(O-15:0)	0.0002246	0.0007661	1.1987	0.7488	-0.4174	down	LevelBi	LMGP01060009	512.3329	8.0341
6-Methyl-1-(3-Sulfanylpropanoyl)-2-Piperidinecarboxylic Acid	0.001505	0.003488	1.2075	1.347	0.4297	up	LevelBi	-	295.11	3.991
Oxyglutinosone	1.483E-7	3.502E-6	1.2063	0.6496	-0.6225	down	LevelBii	HMDB0302978	281.138	3.2337
Thr-Ala-Phe	0.02086	0.03261	1.1814	0.4961	-1.0114	down	LevelBi	-	338.1684	2.5772
Prolinamide, 5-Oxo-L-Prolyl-L-Norvalyl-	0.0035	0.007082	1.2067	1.7769	0.8293	up	LevelBii	HMDB0253911	383.1925	2.0345

Metabolite	P_value	FDR	VIP_PLS-DA	FC(A/E)	Log2FC (A/E)	Regulate	level	Library ID	M/Z	Retention time
13,14-Dihydro Pgf-1A	0.002354	0.005053	1.2028	0.588	-0.766	down	LevelBii	HMDB0005076	357.2632	6.6837
Milbemycin Alpha9	0.0006939	0.00185	1.1958	1.6381	0.712	up	LevelBii	HMDB0254726	618.3026	2.3114
Lpg(18:2)	0.0134	0.02222	1.2192	1.6621	0.733	up	LevelBi	HMDB0240600	507.2702	10.8525
Pantetheine	0.01015	0.0175	1.246	0.5261	-0.9267	down	LevelBii	HMDB0003426	277.1215	2.623
(11E,13Z)-10-Hydroxyoctadeca-11,13-Dienoylcarnitine	0.002467	0.005251	1.1998	1.8483	0.8862	up	LevelBii	HMDB0241544	484.325	6.5452
Phe-Ile-Ser	0.000362	0.001086	1.1904	1.4259	0.5119	up	LevelBi	-	366.2013	2.4775
Achyranthoside D	0.0002654	0.0008424	1.1916	0.6604	-0.5987	down	LevelBii	HMDB0031023	1177.5225	7.5177
Pa(5-Iso Pgf2Vi/18:3(6Z,9Z,12Z))	0.00474	0.009164	1.2038	0.5443	-0.8775	down	LevelBii	HMDB0264142	785.4291	7.8848
Ala-Thr-Trp-Leu-Pro-Pro-Arg	0.002207	0.004788	1.1732	2.7849	1.4776	up	LevelBii	HMDB0249908	838.4663	3.9438
Trp-Glu	0.000478	0.001362	1.1932	1.4213	0.5072	up	LevelBi	HMDB0029082	334.1395	2.2309
Glycerophosphoinositol	0.03312	0.04875	1.2595	0.3249	-1.6218	down	LevelBii	HMDB0011649	333.0592	4.0091
Linalool Oxide D 3-[Apiosyl-(1->6)-Glucoside]	0.0009611	0.002421	1.196	1.734	0.7941	up	LevelBii	HMDB0031367	485.1997	3.1396
Leu-Ser-Pro	0.002321	0.004996	1.1986	0.6711	-0.5755	down	LevelBi	-	316.1864	6.349
Sulfolithocholic Acid	2.769E-5	0.0001405	1.195	1.6933	0.7599	up	LevelBii	HMDB0000907	457.261	2.9348
Thymine	3.157E-6	3.453E-5	1.1885	0.618	-0.6942	down	LevelBi	HMDB0000262;HMDB0010337;HMDB0000262;PW_C000179	127.0491	1.9821
Xanthine	6.791E-6	4.35E-5	1.1846	0.7229	-0.4682	down	LevelBi	HMDB0000292;PW_C000198	153.0396	1.8336
Tyr-Ala-Leu	9.626E-5	0.0003912	1.1781	1.4423	0.5283	up	LevelBi	-	366.2002	2.5772
Ile-Gln-Phe	0.0142	0.02336	1.2097	2.1309	1.0915	up	LevelBi	-	407.2267	3.4783
Glu-Tyr	0.0001001	0.0004043	1.187	1.629	0.704	up	LevelBii	HMDB0028831	309.1104	2.1202
6-(1-(3,5,5,8,8-Pentamethyl-5,6,7,8-Tetrahydronaphthalen-2-Yl)Cyclopropyl)Nicotinic Acid	3.102E-8	1.124E-6	1.1835	1.744	0.8024	up	LevelBii	HMDB0244694	344.2051	4.0371
16-Hydroxyhexadecanoylcarnitine	0.02438	0.03731	1.1759	1.8561	0.8923	up	LevelBii	HMDB0241458	398.3247	6.9441
(3-Ethyl-3-Hydroxy-7-Azaspiro[3.5]Nonan-7-Yl)-Thiophen-2-Ylmethanone	5.779E-7	9.617E-6	1.1896	0.7065	-0.5013	down	LevelBi	-	280.1382	1.1576
Trans-P-Coumaroyl Beta-D-Glucopyranoside	2.554E-7	5.174E-6	1.1819	1.7607	0.8161	up	LevelBii	HMDB0302089	385.1123	3.4425
Guanadrel	3.085E-5	0.0001537	1.1814	1.3467	0.4294	up	LevelBii	HMDB0014371	258.1448	1.8555
Cenisertib	0.00627	0.01164	1.1722	5.4951	2.4581	up	LevelBii	HMDB0249799	452.253	2.8357
Prostaglandin A2	0.0001156	0.0004559	1.1804	0.5887	-0.7644	down	LevelBi	HMDB0002752;PW_C001699	335.2202	5.3319
Protoveratrine	0.0001737	0.0006299	1.1967	2.9071	1.5396	up	LevelBii	HMDB0256876	838.4291	3.703
Temocaprilat	1.648E-7	3.768E-6	1.1815	0.3604	-1.4724	down	LevelBii	HMDB0061721	429.0965	3.0733
Gly-Phe-Val	2.95E-5	0.0001484	1.1779	1.5294	0.613	up	LevelBi	-	322.1742	2.3901
N-Acetyl-Dl-Tryptophan	0.0001186	0.0004664	1.1762	0.5646	-0.8246	down	LevelBi	-	245.0917	3.8598
Vertilmicin	0.0001074	0.000428	1.1717	1.414	0.4998	up	LevelBii	HMDB0242753	528.2835	2.8952
1-Cyclohexyl-3-(1-Propylsulfonyl-3,4-Dihydro-2H-Quinolin-7-Yl)Urea	6.853E-5	0.0002982	1.1713	3.5454	1.8259	up	LevelBi	-	380.2025	5.1562
Trp-Ala	0.00115	0.002793	1.1797	1.445	0.531	up	LevelBi	HMDB0029076	276.1328	2.3004
Dg(Lte4/I-12:0/0:0)	0.0006015	0.001644	1.179	0.6994	-0.5159	down	LevelBii	HMDB0298379	695.4447	5.8592
Asp-Pro-Ile	2.081E-5	0.0001114	1.1787	1.4368	0.5229	up	LevelBi	-	344.1828	2.4099
Phe-Ile-Ala	0.000802	0.002083	1.1733	1.4801	0.5657	up	LevelBi	-	350.2074	2.786
7-T-Butoxyiminomethyl-Camptothecin	0.003468	0.00703	1.1836	2.1106	1.0776	up	LevelBii	HMDB0247287	482.151	3.433
Cyclopassifloside I	1.716E-6	2.195E-5	1.1716	1.5979	0.6761	up	LevelBii	HMDB0035944	697.4234	2.8497
Lpc(O-17:1)	0.000966	0.002431	1.1594	0.7248	-0.4643	down	LevelBi	-	538.348	8.5568
Pacific Ciguatoxin 4A	0.01239	0.02079	1.1941	1.4359	0.522	up	LevelBii	HMDB0033124	1025.5542	7.7154
2-(5,8-Tetradecadienyl)Cyclobutanone	0.00537	0.01021	1.176	0.7363	-0.4417	down	LevelBii	HMDB0037519	263.2354	8.7842
Lps(22:4)	1.872E-5	0.0001016	1.1687	1.3608	0.4444	up	LevelBi	LMGP03050014	572.2972	9.0571
(Z)-2-(5-Tetradecenyl)Cyclobutanone	0.003407	0.006923	1.1682	0.7668	-0.3831	down	LevelBii	HMDB0037543	265.2516	8.115
Octanamide	0.003153	0.006486	1.1699	1.7213	0.7835	up	LevelBii	HMDB0255903	185.1636	2.2904
Lpe(20:3)	0.001088	0.002668	1.1676	1.2548	0.3274	up	LevelBi	HMDB0011484	502.2917	7.9407
Rolitetracycline	0.000379	0.001128	1.1588	2.1247	1.0872	up	LevelBii	HMDB0015414	508.2044	2.3777
Rutin	5.773E-6	3.847E-5	1.1655	0.5845	-0.7746	down	LevelBii	HMDB0003249	631.1236	3.4236
2,3-Dimethyl-7-Nitro-1H-Indole	0.0008518	0.002195	1.1694	1.3996	0.485	up	LevelBi	-	245.0912	2.3104
Milbemycin Alpha10	0.003545	0.007156	1.1716	1.7491	0.8066	up	LevelBii	HMDB0254722	652.3417	4.0106
Lappaconitine	2.596E-5	0.0001332	1.162	1.3094	0.3889	up	LevelBi	-	585.3223	2.8555
Vaccenic Acid	0.00401	0.007935	1.1628	0.7952	-0.3307	down	LevelBi	HMDB0003231;HMDB0244344	283.2625	8.115
Asp-Pro-Arg	0.0004095	0.001199	1.1609	1.3488	0.4317	up	LevelBi	-	419.2265	3.1428
2-(2-Methylphenoxy)Acetohydrazide	1.144	1.625	1.169	0.746	-0.422	down	Leve	-	213.1	1.8534

Metabolite	P_value	FDR	VIP_PLS-DA	FC(A/E)	Log2FC(A/E)	Regulate	level	Library ID	M/Z	Retention time
	E-6	E-5	9	3	2		lBi		223	
Fahfa(18:3/3:0)	0.001416	0.003319	1.1626	0.6803	-0.5558	down	LevelBi	–	349.2363	8.5663
7-O-Succinyl Macrolactin A	0.003723	0.007456	1.1755	1.666	0.7364	up	LevelBii	HMDB0247277	547.2531	2.6609
Pa(2:0/16:4)	0.002174	0.004725	1.1558	3.4507	1.7869	up	LevelBi	–	489.1936	3.0261
gamma-Glu-Phe	6.501E-5	0.0002853	1.1601	1.4135	0.4992	up	LevelBii	HMDB0000594	275.1027	3.48
(2S)-2-[[(2R,3S)-2-Amino-3-Methylpentanoyl]Amino]-4-Methylpentanoic Acid	6.217E-5	0.0002756	1.1583	1.2288	0.2972	up	LevelBi	–	245.1859	2.796
12,15-Epoxy-13,14-Dimethyloctadeca-12,14,16-Trienoic Acid	0.0001678	0.0006149	1.1538	0.4506	-1.15	down	LevelBii	HMDB0112071	355.2076	6.8774
3-O-Methylniveusin A	0.0001052	0.0004214	1.158	0.7048	-0.5047	down	LevelBii	HMDB0039084	409.1821	1.9424
10-Hydroxy-3-Methoxy-1,3,5,7-Cadinatetraen-9-One	0.002796	0.00585	1.1779	1.9833	0.9879	up	LevelBii	HMDB0036456	565.2841	8.6598
Atropine	2.912E-6	3.239E-5	1.1592	0.7198	-0.4744	down	LevelBi	HMDB0000779	322.1992	3.7153
8-Aminooctanoic Acid	2.433E-6	2.844E-5	1.1553	1.3074	0.3867	up	LevelBii	HMDB0247418	160.1324	2.6965
Fumonisin A2	0.00338	0.006879	1.1576	1.5348	0.6181	up	LevelBii	HMDB0034699	712.3915	3.6363
Thr-Gln-Lys	0.00659	0.01213	1.1423	0.6447	-0.6333	down	LevelBi	–	376.2208	2.7262
[6]-Gingerdiol 3,5-Diacetate	0.0006425	0.001738	1.1577	0.7148	-0.4844	down	LevelBi	HMDB0040568	379.2086	6.6467
Melleolide	0.002748	0.005758	1.1543	0.7251	-0.4637	down	LevelBii	HMDB0035689	399.1847	8.1252
Trp-Gln	3.04E-5	0.000152	1.146	1.5693	0.6501	up	LevelBii	HMDB0029081	331.1394	2.416
Cannabigerolate	2.63E-5	0.0001348	1.1549	0.7046	-0.5052	down	LevelBii	HMDB0304284	395.2028	5.5172
Betavulgaroside Ix	0.0002648	0.000841	1.1502	0.6854	-0.5449	down	LevelBii	HMDB0034869	1147.512	7.5737
Fa(22:6)	0.0001388	0.0005305	1.1561	0.6858	-0.5441	down	LevelBi	–	655.47	8.5757
13-Docosenamide	0.03147	0.04656	1.1643	0.562	-0.8313	down	LevelBii	HMDB0244507	370.3657	7.4624
2-[Methyl(3-Phenylpropanoyl)Amino]Benzoic Acid	2.347E-5	0.000123	1.151	0.5728	-0.8039	down	LevelBii	HMDB0032845	342.1322	4.1484
3-Oxohexadecanoic Acid	0.00967	0.01681	1.1333	0.5424	-0.8825	down	LevelBii	HMDB0010733	269.2121	9.0383
Amphotericin B Methyl Ester	0.0009836	0.002465	1.1554	1.7311	0.7917	up	LevelBii	HMDB0248359	960.4956	4.6876
Angiotensin Iv	0.0001294	0.0004999	1.1469	0.6216	-0.686	down	LevelBii	HMDB0001038	819.3978	5.5264
Terfenadine	0.0005832	0.001604	1.1476	1.3108	0.3904	up	LevelBii	HMDB0014486	470.3035	8.6131
Glucose	4.386E-7	7.758E-6	1.1571	0.7311	-0.452	down	LevelBii	HMDB0000122	179.0552	0.7177
Trp-Lys	0.0006913	0.001845	1.1487	1.4046	0.4902	up	LevelBii	HMDB0029088	391.197	3.4141
Ethyl 2-Amino-3-Phenylpropanoate	2.017E-6	2.482E-5	1.1467	1.332	0.4136	up	LevelBi	HMDB0252046	194.1167	2.8952
Annomuricatin B	0.04816	0.0679	1.1968	1.7449	0.8032	up	LevelBii	HMDB0303182	722.3621	4.0206
Pectic Acid	0.0003976	0.00117	1.1479	0.6206	-0.6883	down	LevelBii	HMDB0003363	175.0234	1.8648
Cosmosiin	0.002193	0.004764	1.1452	0.6068	-0.7206	down	LevelBi	HMDB0037340	433.109	4.1388
Trp-Val-Phe	0.002725	0.00572	1.1569	1.5662	0.6473	up	LevelBi	–	451.2308	4.2174
3-O-Beta-D-Galactopyranosyl-L-Arabinose	0.04627	0.06558	1.2348	0.2549	-1.9722	down	LevelBii	HMDB0038854	347.0747	4.6386
Stevioside	6.149E-5	0.000273	1.1432	0.6663	-0.5857	down	LevelBii	HMDB0034945	849.3723	5.7568
Taurodeoxycholic Acid 3-Glucuronide	0.004744	0.00917	1.1633	1.3489	0.4318	up	LevelBii	HMDB0240734	656.3162	7.611
Mangostanol	2.764E-6	3.124E-5	1.1436	0.6223	-0.6842	down	LevelBii	HMDB0029868	461.1379	2.576
Canrenoic Acid	7.876E-5	0.0003332	1.1644	0.653	-0.6148	down	LevelBii	HMDB0249591	393.1831	1.9876
Lpe(20:5)	0.001996	0.004407	1.1478	1.3273	0.4085	up	LevelBi	HMDB0011489	498.2614	7.3591
Gly-Val-Leu	0.0004511	0.001297	1.1396	1.3859	0.4708	up	LevelBi	–	288.1909	2.8059
Ala-Arg-Tyr	2.0E-5	0.0001077	1.1465	0.6903	-0.5347	down	LevelBi	–	409.2163	3.9615
Prostaglandin A1	0.0001545	0.0005763	1.137	0.5378	-0.8948	down	LevelBii	HMDB0002656	381.2259	5.5725
Kaempferol 3-(3'',6''-Diacetyl-2'',4''-Di-P-Coumaroylrhamnoside)	0.0008933	0.002279	1.1433	0.6185	-0.6931	down	LevelBii	HMDB0029503	805.1708	4.139
Azacitidine	0.0004172	0.001217	1.1378	1.5689	0.6497	up	LevelBii	HMDB0015063	225.0619	1.3521
Tuberoside F	0.01202	0.02026	1.1709	0.4977	-1.0067	down	LevelBii	HMDB0302986	1077.5425	6.5081
5-Methyl-N-(2-Oxo-3-Azepanyl)-2-(Trifluoromethyl)-3-Furansulfonamide	0.001316	0.00312	1.1604	0.5186	-0.9472	down	LevelBi	–	305.0558	0.5797
Ala-Arg	7.794E-6	4.894E-5	1.1383	0.7501	-0.4149	down	LevelBii	HMDB0028681	268.1392	1.5719
Tryptophol	0.004811	0.009283	1.1563	1.8339	0.8749	up	LevelBii	HMDB0003447	142.0654	2.3969
Val-Lys	0.00655	0.01207	1.1433	1.429	0.515	up	LevelBi	HMDB0029132	246.1799	1.8926
Gentamicin B	0.0001554	0.000579	1.1315	1.4799	0.5655	up	LevelBii	HMDB0252687	517.2238	2.3208
(2'E,4'Z,8E)-Colneleic Acid	0.008069	0.01437	1.1326	0.5924	-0.7554	down	LevelBii	HMDB0030995	329.1868	7.415
Cucurbitacin B	4.494E-8	1.437E-6	1.1342	1.6352	0.7095	up	LevelBii	HMDB0034927	613.3316	3.9514
Pyroglutamyllysine	9.894E-5	0.0004004	1.1284	0.6146	-0.7024	down	LevelBii	HMDB0341383	302.1348	2.5098
Cyclopropanecarboxylic Acid	0.000	0.000	1.121	0.620	-0.688	down	Leve	HMDB0250675	51.02	2.1616

Metabolite	P_value	FDR	VIP_PLS-DA	FC(A/E)	Log2FC(A/E)	Regulate	level	Library ID	M/Z	Retention time
	3149	9689	5	6	3		lBii		28	
Pro-Phe-Gly	0.0008473	0.002185	1.1307	1.2682	0.3427	up	LevelBi	-	320.1599	2.2607
Pe(Pgf1Alpha/24:1(15Z))	0.01294	0.02157	1.1346	0.6159	-0.6991	down	LevelBii	HMDB0284643	882.6224	8.9077
Kaempferide 3-[Rhamnopyranosyl-(1->6)-Glucoside] 7-Rhamnoside	0.004822	0.009297	1.1375	1.916	0.9381	up	LevelBii	HMDB0040804	789.2016	10.8525
Phe-Asn	0.001241	0.002971	1.1355	1.383	0.4678	up	LevelBi	HMDB0028990	280.1279	2.0116
Bolandiol	0.00306	0.006313	1.1181	0.7294	-0.4553	down	LevelBii	HMDB0249338	277.2151	7.5209
Tyr-Phe	0.0002585	0.0008243	1.1289	1.3019	0.3806	up	LevelBi	HMDB0029112	329.1482	2.8653
N-(2-Hydroxy-3-Methylbutanoyl)Leucine	1.592E-5	8.899E-5	1.1288	0.6683	-0.5815	down	LevelBi	-	230.1385	5.0382
Carnosic Acid Diacetate Methyl Ester	7.99E-6	4.996E-5	1.122	1.4392	0.5252	up	LevelBi	-	431.2397	2.3602
Methyl Oxindole-3-Acetate	0.0001022	0.0004114	1.1237	1.8088	0.8551	up	LevelBii	HMDB0303691	250.0709	3.1772
Met-Phe	0.0008774	0.002248	1.1258	1.3739	0.4583	up	LevelBi	HMDB0028980	297.126	2.9645
Dictyoquinazol C	0.00115	0.002793	1.1255	1.7028	0.7679	up	LevelBii	HMDB0040442	363.0977	2.9602
Lpe(20:4)	0.0007404	0.001952	1.1271	1.197	0.2594	up	LevelBi	HMDB0011487	500.2764	7.7115
Moexipril	4.857E-5	0.0002241	1.1231	0.5866	-0.7695	down	LevelBii	HMDB0014829	995.4645	4.4631
Toluene	0.0003966	0.001169	1.1122	0.7266	-0.4607	down	LevelBii	HMDB0034168	93.0693	1.8633
Ginsenoside Rh4	0.001068	0.00263	1.1153	1.6583	0.7297	up	LevelBi	HMDB0041534	643.4131	5.0389
9S,11R-Dihydroxy-15-Oxo-13E-Prostenoic Acid	0.003617	0.00728	1.1219	0.6178	-0.6948	down	LevelBi	-	377.2276	6.1235
Canavalioside	0.0009548	0.002411	1.1164	2.4573	1.2971	up	LevelBii	HMDB0036317	545.2574	3.9717
Norzolmitripan	0.001692	0.003837	1.1243	1.36	0.4437	up	LevelBii	HMDB0060966	318.1449	2.2638
Cyclocalamin	1.662E-7	3.794E-6	1.1247	0.7903	-0.3396	down	LevelBii	HMDB0035863	537.1916	2.1688
Lpe(20:1)	0.002696	0.005672	1.1225	1.3701	0.4543	up	LevelBi	HMDB0011482	506.3226	8.6225
Ile-Arg-Leu	0.0108	0.01844	1.1254	1.5256	0.6094	up	LevelBi	-	401.284	2.7164
2,3-Butanediol Apiosylglucoside	0.000747	0.001967	1.1216	1.8876	0.9166	up	LevelBii	HMDB0033063	429.1643	3.5082
Trans-Zeatin	0.0001979	0.0006987	1.1143	0.7031	-0.5082	down	LevelBi	HMDB0012204	274.1278	1.6006
Gentian Violet Cation	0.007843	0.01405	1.1323	1.5586	0.6403	up	LevelBi	-	373.2528	2.0512
7-Hydroxyoct-2-Enoylglycine	6.646E-5	0.0002905	1.1214	0.6923	-0.5305	down	LevelBii	HMDB0094774	236.0919	2.1593
Cyclo(Prolylvalyl)	0.0004895	0.001389	1.1051	0.6505	-0.6204	down	LevelBi	-	197.1275	2.7362
Paeonol	2.671E-5	0.0001365	1.1316	0.669	-0.5799	down	LevelBii	HMDB0256081	225.0755	3.4613
6|A-Hydroxy-7|A-(Thiomethyl)Spirolactone	0.0007358	0.001943	1.1137	2.2428	1.1653	up	LevelBii	HMDB0246665	403.1979	3.4894
Leu-Leu-Phe	2.752E-5	0.0001399	1.1085	1.345	0.4276	up	LevelBi	-	392.2519	4.0598
Ac-Thr-D-Trp(Cho)-Phe-Nmebzl	0.00205	0.004504	1.1156	0.4738	-1.0776	down	LevelBii	HMDB0257639	670.2936	2.1492
Fahfa(2:0/18:1)	0.01385	0.02287	1.0997	0.6735	-0.5703	down	LevelBi	-	321.242	7.8481
11-Hydroxy-9-Tridecenoic Acid	1.032E-5	6.194E-5	1.123	0.6397	-0.6445	down	LevelBii	HMDB0035881	273.1693	5.0382
Tamsulosin	3.13E-5	0.0001553	1.1141	0.7279	-0.4581	down	LevelBi	HMDB0014844;HMDB0259951	409.1811	3.133
Leu-Asp-Phe	0.0008771	0.002248	1.112	1.3182	0.3986	up	LevelBi	-	394.1962	3.4196
Ile-Ser-Leu	7.432E-6	4.702E-5	1.107	1.38	0.4647	up	LevelBi	-	332.2164	3.3703
Gly-Leu	0.01406	0.02318	1.1336	1.389	0.4741	up	LevelBi	HMDB0000759	189.1228	2.1912
1-O-Hexadecyl-2-O-Ethyl-Sn-Glycero-3-Phosphocholine	0.0008721	0.002239	1.112	0.8346	-0.2608	down	LevelBi	-	510.3887	8.8826
Secoeremopetasitolide B	1.56E-5	8.769E-5	1.1036	1.4798	0.5654	up	LevelBii	HMDB0041363	427.2291	3.123
12-Oxo Phytodienoic Acid	0.0004435	0.001279	1.1099	0.7107	-0.4927	down	LevelBi	-	293.2096	5.5174
Thr-Ile	3.276E-5	0.0001613	1.1104	1.2678	0.3423	up	LevelBii	HMDB0029064	231.1339	1.846
Hophe-Lys-Oh	0.0005848	0.001606	1.1105	1.527	0.6107	up	LevelBi	-	414.1628	3.4236
Pip(5-Iso Pgf2Vi/20:4(5Z,8Z,11Z,14Z))	0.009069	0.01591	1.1363	0.6147	-0.702	down	LevelBii	HMDB0280017	989.4475	5.9605
Pro-Ile-Leu	8.097E-6	5.049E-5	1.1034	1.2687	0.3433	up	LevelBi	-	342.237	3.3308
4-Deoxytetronic Acid	6.009E-7	9.908E-6	1.1082	0.7653	-0.3858	down	LevelBi	HMDB0000549	87.0436	0.6887
Sumithrin	0.002731	0.00573	1.1061	1.5676	0.6485	up	LevelBi	HMDB0242366	368.221	2.3004
Citrusin I	0.003701	0.007421	1.1214	2.5074	1.3262	up	LevelBii	HMDB0303680	762.4023	3.8692
Tyr-Gln-Ile	5.565E-5	0.0002516	1.1026	1.31	0.3896	up	LevelBi	-	423.2209	3.0339
Phe-Pro-Thr	7.44E-5	0.0003189	1.1009	1.3193	0.3997	up	LevelBi	-	364.1857	2.537
Gln-Val-Ile	0.01209	0.02036	1.1132	1.6725	0.742	up	LevelBi	-	359.2274	2.6665
Ile-Ile-Thr	0.0001106	0.0004387	1.104	1.2615	0.3351	up	LevelBi	-	346.2315	2.35
4-(4-Nitrobenzyl)Pyridine	2.369E-5	0.000124	1.1051	1.4614	0.5473	up	LevelBii	HMDB0246294	259.0742	2.4535
Cdp	2.258E-7	4.741E-6	1.1062	0.6785	-0.5596	down	LevelBii	HMDB0001546	407.9955	1.7849

Metabolite	P_value	FDR	VIP_PLS-DA	FC(A/E)	Log2FC (A/E)	Regulate	level	Library ID	M/Z	Retention time
Glycinoeclepin C	0.001724	0.0039	1.1156	3.1012	1.6328	up	LevelBii	HMDB0037036	495.2424	2.2638
N-Cycloheptyl-1-[2-(4-Fluorophenyl)Pyrimidin-4-Yl]Piperidine-3-Carboxamide	4.982E-5	0.0002287	1.1013	1.3239	0.4048	up	LevelBi	-	397.2429	2.5469
Tunicamycin A	0.001667	0.003791	1.1106	0.6614	-0.5965	down	LevelBii	HMDB0259327	851.3886	5.5541
Cytidine	5.705E-6	3.847E-5	1.1026	1.3712	0.4554	up	LevelBii	HMDB0000089	242.0787	0.6618
1-Isothiocyanato-8-(Methylthio)Octane	2.143E-5	0.0001138	1.1132	0.6422	-0.6389	down	LevelBii	HMDB0038447	216.0865	2.642
Gamabufotalin	0.0004722	0.00135	1.0953	1.3157	0.3958	up	LevelBii	HMDB0252604	407.2227	2.3104
5,6-Octadienoylglycine	0.000358	0.001077	1.0954	1.4351	0.5211	up	LevelBii	HMDB0094798	215.1376	2.6665
Methyl 3-(Benzylamino)-2-Methylpropanoate	1.711E-5	9.435E-5	1.1044	0.6327	-0.6603	down	LevelBi	-	262.1424	5.5272
Didemnin A	3.582E-5	0.0001738	1.0948	1.5172	0.6014	up	LevelBii	HMDB0251219	984.6067	8.144
Avenestergenin B2	7.179E-5	0.0003098	1.0961	0.5632	-0.8284	down	LevelBii	HMDB0035266	627.3422	9.2703
Ps(O-10:0/16:1)	0.0005979	0.001637	1.1058	1.5395	0.6225	up	LevelBi	-	694.4291	8.4733
Lpc(O-18:2)	4.818E-5	0.0002226	1.0998	0.7961	-0.329	down	LevelBi	-	550.3483	8.3725
(2S)-2-[[(2R)-2-Amino-3-Carboxypropanoyl]Amino]-4-Methylpentanoic Acid	0.0005276	0.001479	1.1005	1.3285	0.4098	up	LevelBi	-	247.1287	2.2706
Lpi(18:2)	0.000448	0.00129	1.0965	1.3124	0.3922	up	LevelBi	HMDB0240597	595.2867	10.8525
Ile-Thr-Ile	2.729E-5	0.000139	1.0895	1.3156	0.3958	up	LevelBi	-	346.2319	3.4196
Inosine	4.787E-5	0.0002214	1.0882	1.4475	0.5336	up	LevelBi	HMDB0000195;HMDB0247301;PW_C000127	269.0875	1.9919
Ulimorelin	0.000654	0.001762	1.0812	2.0709	1.0503	up	LevelBii	HMDB0259396	521.2889	3.656
9-Cis-Retinol	0.00155	0.003573	1.0947	0.757	-0.4017	down	LevelBi	HMDB0006217;LMPR01090009;PW_C002666	287.2342	6.447
9S-Hydroxy-10E,12Z,15Z-Octadecatrienoic Acid	0.001875	0.004179	1.098	0.7482	-0.4185	down	LevelBi	LMFA02000024	295.2256	6.641
17-Phenyl-18,19,20-Trinor-Prostaglandin D2	2.537E-5	0.0001307	1.0918	1.3204	0.401	up	LevelBii	HMDB0062679	387.2193	2.1311
Val-Val-Ala	1.324E-8	6.076E-7	1.0966	1.3507	0.4337	up	LevelBi	-	288.1921	6.5543
18R-Hepe	3.095E-5	0.000154	1.0925	0.6724	-0.5727	down	LevelBii	HMDB0012611	319.2248	5.0586
8'-Hydroxyabscisate	0.003322	0.006782	1.1024	1.4373	0.5234	up	LevelBii	HMDB0304246	278.1137	2.0157
Adenosine Monophosphate	9.75E-5	0.0003956	1.0917	0.7278	-0.4584	down	LevelBi	HMDB0000045;PW_C000032	348.0687	1.9224
4-Hydroxy-L-Isoleucine	2.071E-8	8.148E-7	1.0905	1.4553	0.5413	up	LevelBi	-	130.085	1.6103
Esmolol	0.0004823	0.001373	1.0882	1.5167	0.6009	up	LevelBii	HMDB0014333	316.1503	2.3208
11'-Carboxy-Gamma-Chromanol	0.001557	0.003583	1.0776	0.6015	-0.7333	down	LevelBii	HMDB0012517	403.2816	9.2981
7-Methylguanosine	6.425E-6	4.158E-5	1.0884	1.5452	0.6278	up	LevelBii	HMDB0001107	319.0885	2.082
Cinereain	0.001047	0.002587	1.0779	1.7011	0.7665	up	LevelBii	HMDB0035218	699.3105	4.5832
Lps(22:6)	0.0001983	0.0006997	1.0808	1.4606	0.5466	up	LevelBi	LMGP03050013	568.2636	8.3544
3-Hydroxytrideca-4,6-Dienoic Acid	0.009353	0.01633	1.0915	2.6721	1.418	up	LevelBii	HMDB0340751	225.1492	5.6277
Ser-Ile	0.005986	0.01118	1.1031	1.3701	0.4543	up	LevelBi	HMDB0029042	219.1332	2.1616
Withanolide A	0.01742	0.0279	1.1069	1.4752	0.5609	up	LevelBii	HMDB0034415	435.2566	2.796
Pa(Pgf2Alpha/12:0)	0.0006141	0.001674	1.0803	1.5812	0.661	up	LevelBii	HMDB0262777	711.3836	2.3777
Lps(20:4)	0.0004151	0.001212	1.0817	1.2613	0.3349	up	LevelBi	LMGP03050007	544.265	8.4733
Phe-Met	0.001658	0.003776	1.0827	1.3323	0.4139	up	LevelBi	HMDB0029001	297.1254	2.6965
(22E, 24X)-Ergosta-4,6,8,22-Tetraen-3-One	0.0003122	0.0009624	1.0721	1.2922	0.3698	up	LevelBii	HMDB0030898	413.2822	8.917
Ergosine	0.01666	0.02684	1.1026	1.7152	0.7784	up	LevelBii	HMDB0251913	582.2518	3.4425
Pe(O-8:0/10:0)	0.01486	0.02432	1.089	1.3767	0.4613	up	LevelBi	-	961.6266	8.669
Trans-13-Octadecenoic Acid	0.005391	0.01024	1.0774	0.7762	-0.3654	down	LevelBi	-	247.2412	8.115
Trp-Asn	1.766E-5	9.685E-5	1.0859	1.4112	0.497	up	LevelBii	HMDB0029078	317.1283	2.5949
Hepta-2,4-Dienoylcarnitine	1.497E-5	8.474E-5	1.0734	1.4425	0.5286	up	LevelBii	HMDB0241690	270.1689	4.1978
1-(1-Pentyl-1H-Indol-3-Yl)-2-Phenylethanone	0.0002828	0.0008895	1.1071	1.5875	0.6667	up	LevelBi	-	347.2095	1.8237
Blumenol C Glucoside	0.002637	0.005561	1.0899	0.3932	-1.3466	down	LevelBii	HMDB0040668	417.2111	4.2132
Floionolic Acid	0.006214	0.01156	1.0809	0.6259	-0.6761	down	LevelBii	HMDB0034295	353.2311	6.9054
Val-Val-Phe	0.0001113	0.0004409	1.0723	1.2653	0.3394	up	LevelBi	-	364.2213	3.321
Citreoviridin D	1.526E-6	2.001E-5	1.0746	1.3947	0.4799	up	LevelBii	HMDB0030522	417.2309	2.5469
Alfentanil	0.01518	0.02477	1.0712	0.2962	-1.7555	down	LevelBii	HMDB0014940	451.2264	7.6016
His-gamma-Glu	0.0001476	0.0005562	1.0771	0.6338	-0.658	down	LevelBii	HMDB0028899	264.1078	0.727
2-Hydroxy-4-(4-Methoxyphenyl)-6-Phenylnicotinonitrile	8.299E-5	0.0003474	1.0688	0.7639	-0.3885	down	LevelBi	-	320.1424	2.6865
Lpe(24:4)	0.01452	0.02382	1.0975	1.4693	0.5551	up	LevelBi	-	556.3374	8.5289
N-Carbobenzoxyglycyl-Prolyl-4-Methylcoumarinyl Amide	0.002667	0.005619	1.0755	1.5737	0.6541	up	LevelBii	HMDB0259966	522.1848	2.1593
5(S),14(R)-Lipoxin B4	0.001	0.004	1.074	0.677	-0.561	down	Leve	HMDB0005082	351.2	6.4154

Metabolite	P_value	FDR	VIP_PLS-DA	FC(A/E)	Log2FC(A/E)	Regulate	level	Library ID	M/Z	Retention time
	999	41	3	5	7		lBi		135	
Tyr-Ala-Asp	0.0001635	0.000603	1.0647	1.4052	0.4908	up	LevelBi	-	368.1439	2.0017
(4E,7E,10E)-Hexadeca-4,7,10-Trienedioic Acid	0.0001677	0.0006148	1.0736	1.5553	0.6372	up	LevelBii	HMDB0340904	263.1657	2.0314
M2 Di-Hydroxylated Metabolite	0.02895	0.04324	1.1012	1.9048	0.9296	up	LevelBii	HMDB0061046	406.1961	3.5738
Trp-Ile-Asp	0.0001357	0.0005211	1.0683	1.5084	0.593	up	LevelBi	-	433.2071	3.1428
Ile-Leu-Gln	0.0009592	0.002418	1.0753	1.2866	0.3636	up	LevelBi	-	373.2422	2.1616
Casomorphin	9.855E-5	0.0003992	1.066	0.5284	-0.9204	down	LevelBii	HMDB0059787	828.4309	3.9615
Morphiceptin	0.001086	0.002665	1.0702	1.3801	0.4648	up	LevelBii	HMDB0005777	486.254	3.1925
Ile-Val-Leu	3.199E-5	0.0001583	1.0601	1.2243	0.2919	up	LevelBi	-	344.2533	3.5475
Ile-Asn	0.0002796	0.0008807	1.0647	1.3183	0.3987	up	LevelBii	HMDB0028902	244.1292	1.3521
Hernandezine	0.0009977	0.00249	1.0672	1.4163	0.5021	up	LevelBi	-	653.3253	3.0142
Phe-Tyr	0.000441	0.001273	1.0582	1.2546	0.3272	up	LevelBi	HMDB0256384	329.1486	2.5772
Asp-Tyr	0.005009	0.009611	1.0743	1.8794	0.9103	up	LevelBi	HMDB0028765	297.1072	2.1311
Rotundifoline	1.187E-5	6.987E-5	1.0724	1.5278	0.6115	up	LevelBii	HMDB0302761	459.21	3.1772
Trp-Asp	0.001595	0.003656	1.0616	1.3744	0.4588	up	LevelBii	HMDB0029079	318.1081	2.1879
Leu-Ile-Glu	4.77E-5	0.0002207	1.0587	1.3018	0.3805	up	LevelBi	-	374.2265	2.3901
Methyl (2R)-Piperidine-2-Carboxylate	0.0002815	0.0008861	1.0467	0.682	-0.5521	down	LevelBi	-	144.1011	0.9516
N-[(3A,5B,7A)-3-Hydroxy-24-Oxo-7-(Sulfooxy)Cholan-24-Yl]-Glycine	0.004101	0.00809	1.0672	1.5743	0.6547	up	LevelBii	HMDB0002496	530.2746	2.6069
[4-(4-Nitrophenyl)Piperazin-1-Yl](4-Propoxyphenyl)Methanone	0.002675	0.005633	1.0399	1.49	0.5753	up	LevelBi	-	370.1748	3.2222
(13Z,16Z,19Z)-Docosa-13,16,19-Trienoylcarnitine	0.0467	0.0661	1.0893	0.2437	-2.0367	down	LevelBii	HMDB0241605	522.377	7.3685
Ile-Met	0.0001027	0.0004128	1.054	1.2823	0.3587	up	LevelBii	HMDB0028913	261.1268	2.2829
His-Ala-Ile	6.339E-5	0.0002797	1.0528	1.272	0.3471	up	LevelBi	-	340.1955	2.0712
Histidylphenylalanine	6.793E-5	0.0002956	1.0544	1.4751	0.5608	up	LevelBii	HMDB0028892	301.1302	2.0063
Trans-2-Hydroxycinnamic Acid	3.061E-5	0.0001528	1.049	1.2273	0.2954	up	LevelBi	HMDB0002641	165.0538	1.8436
Maltotetraose	0.01707	0.02741	1.0671	0.448	-1.1584	down	LevelBi	HMDB0001296	667.2244	1.0985
(5Ar,10Ar)-Octahydrodipyrrolo[1,2-A:1',2'-D]Pyrazine-5,10-Dione	1.441E-8	6.388E-7	1.0588	0.7125	-0.489	down	LevelBi	-	195.1124	2.3104
Lys-Trp	2.931E-5	0.0001475	1.0511	0.6936	-0.5279	down	LevelBii	HMDB0028962	355.1708	4.3449
4-(4-Methylcyclohexyl)-4-Oxobutanoic Acid	0.0005015	0.001418	1.0462	1.4911	0.5764	up	LevelBii	HMDB0253742	199.1325	5.7423
Lnape(2:0/N-20:0)	0.0006782	0.001815	1.0489	0.8085	-0.3068	down	LevelBi	-	550.3488	8.0059
12,13-Epome	0.01114	0.01894	1.0374	0.7815	-0.3556	down	LevelBi	HMDB0004702	297.2411	7.8121
11-Hydroxyhexadeca-6,12-Dienoic Acid	7.82E-5	0.0003318	1.0484	0.6023	-0.7314	down	LevelBii	HMDB0340891	313.2003	5.9696
Val-Ile-Phe	0.0001542	0.0005756	1.0449	1.2827	0.3592	up	LevelBi	-	378.2366	3.7941
N-Lauroyl Serine	0.0004853	0.00138	1.0397	1.4304	0.5164	up	LevelBii	HMDB0242040	308.1854	6.2769
Palmitoyl Ethanolamide	0.0001651	0.0006072	1.0416	0.7518	-0.4117	down	LevelBi	HMDB0002100	300.2882	8.513
Pe(O-16:1/20:5)	0.006468	0.01195	1.0667	0.5083	-0.9762	down	LevelBi	-	720.5021	9.9073
Tetrahydrocortisol	0.03341	0.04912	1.0324	0.6337	-0.6582	down	LevelBii	HMDB0000949	387.2128	7.5829
9-Hpode	0.003126	0.006436	1.0616	0.8057	-0.3117	down	LevelBi	HMDB0062434;HMDB0242602	311.2213	6.6467
Val-Ile-Thr	0.0007648	0.002005	1.0481	1.2421	0.3128	up	LevelBi	-	332.2154	2.1616
Smgdg(O-16:4/22:6)	0.002459	0.005243	1.0447	2.5072	1.3261	up	LevelBi	-	905.4685	4.1204
2-[2-(4-Aminophenyl)Ethylamino]Ethanol	0.0001819	0.0006543	1.0462	1.239	0.3092	up	LevelBi	-	213.1588	2.1512
Ile-Ile-Glu	0.001093	0.002679	1.0363	1.355	0.4383	up	LevelBi	-	374.2252	2.517
Linoelaidic Acid	0.005517	0.01044	1.0498	0.7747	-0.3684	down	LevelBi	HMDB0006270;LMFA01030123	281.2457	8.7745
Methylmalonic Acid	6.541E-7	1.051E-5	1.0512	0.8244	-0.2787	down	LevelBi	HMDB0000202;LMFA01170118;PW_C000130	117.0189	1.7808
Val-Phe-Ser	1.152E-7	2.893E-6	1.0421	1.3027	0.3816	up	LevelBi	-	393.211	3.6265
Mavatrep	0.004404	0.008606	1.0326	0.5586	-0.84	down	LevelBii	HMDB0254350	443.1384	2.6988
Lpc(16:1)	0.00372	0.007454	1.0554	1.2911	0.3686	up	LevelBi	HMDB0010383	538.3126	7.5829
(4-Phenylbut-3-En-2-Ylideneamino) 2-Chlorobenzoate	0.008058	0.01435	1.0315	0.6462	-0.6299	down	LevelBi	-	282.0688	3.9514
Gly-Ser-Pro-Met-Phe	0.0005841	0.001605	1.0261	1.7628	0.8179	up	LevelBii	HMDB0033242	616.2923	3.273
2-(4-Fluorophenoxy)-N-[2-Oxo-2-(4-Pyridin-2-Ylpiperazin-1-Yl)Ethyl]Acetamide	0.0001395	0.000533	1.039	1.4794	0.565	up	LevelBi	-	373.1703	4.9414
Cauloside C	3.77E-6	3.847E-5	1.0342	1.4262	0.5122	up	LevelBi	-	765.4511	3.4518
N6-Galacturonyl-L-Lysine	0.001131	0.002755	1.0424	1.5434	0.6261	up	LevelBii	HMDB0033106	343.1105	1.3148
Amc Arachidonoyl Amide	0.01225	0.02058	1.0236	0.7695	-0.3781	down	LevelBi	-	460.2811	8.0799
Leu-Gln	3.809E-5	0.0001829	1.0365	1.228	0.2964	up	LevelBi	HMDB0028927	260.1594	1.8534

Metabolite	P_value	FDR	VIP_PLS-DA	FC(A/E)	Log2FC(A/E)	Regulate	level	Library ID	M/Z	Retention time
Amikacin	0.001758	0.003963	1.0473	1.3991	0.4845	up	LevelBii	HMDB0014622	590.2664	3.745
Uridine	0.002199	0.004774	1.0232	1.6849	0.7527	up	LevelBi	HMDB0000296;PW_C000202	243.0603	1.1746
gamma-Glu-Ala	1.821E-5	9.948E-5	1.0352	1.3064	0.3856	up	LevelBii	HMDB0006248	217.0824	0.7365
Dehydroascorbic Acid	7.362E-8	2.045E-6	1.042	0.724	-0.4659	down	LevelBii	HMDB0001264	173.0075	0.7458
Adenosine 2',3'-Cyclic Phosphate	0.008307	0.01473	1.0754	0.547	-0.8704	down	LevelBii	HMDB0011616	364.0246	1.4267
Thr-Phe	0.0005395	0.001506	1.0375	1.3221	0.4029	up	LevelBii	HMDB0029068	265.1183	2.1297
Quinoline-4,8-Diol	0.001369	0.003228	1.0312	1.7683	0.8223	up	LevelBii	HMDB0060289	160.0396	3.4048
4-O-Beta-D-Glucosyl-4-Coumaric Acid	0.02238	0.03468	1.0529	0.4545	-1.1377	down	LevelBi	HMDB0039509	344.1329	2.7562
Adenosine	0.004471	0.008712	1.0179	1.4518	0.5378	up	LevelBi	HMDB0000050;HMDB0247583;PW_C000034	268.1025	1.9224
Lucidenic Acid F	0.0005202	0.001462	1.034	1.2495	0.3214	up	LevelBii	HMDB0036436	457.2623	2.35
Leu-Pro-Ala	0.002724	0.005718	1.0405	1.326	0.4071	up	LevelBi	-	300.1901	2.1616
Trp-Arg-Leu	0.02799	0.04201	1.0595	1.6781	0.7468	up	LevelBi	-	474.2802	3.2222
Cdp-Dg(6 Keto-Pgf1Alpha/18:0)	0.01533	0.02497	1.0582	0.741	-0.4324	down	LevelBii	HMDB0291000	1096.5449	6.8269
Jwh 015-D7	0.006019	0.01123	1.0257	0.6345	-0.6563	down	LevelBi	-	333.2028	7.5643
Tyr-Val-Phe	0.0003446	0.001045	1.0272	1.3876	0.4726	up	LevelBi	-	428.215	3.4783
Anlotinib	0.006941	0.01267	1.0468	1.4696	0.5554	up	LevelBii	HMDB0248447	388.1471	3.0639
Thr-Trp	0.003549	0.007164	1.0392	1.3419	0.4243	up	LevelBi	HMDB0029072	306.1439	2.6567
Ile-Val-Phe	1.136E-5	6.733E-5	1.0254	1.2619	0.3356	up	LevelBi	-	378.2365	3.6661
3-Carboxy-4-Methyl-5-Pentyl-2-Furanpropanoic Acid	0.01897	0.03005	1.0673	1.9567	0.9684	up	LevelBii	HMDB0061643	289.1044	5.9051
Lpe(O-18:2)	0.001439	0.003364	1.0133	0.8425	-0.2473	down	LevelBi	-	462.297	8.4549
Prenyl Glucoside	2.4E-5	0.0001252	1.0257	0.7178	-0.4783	down	LevelBii	HMDB0031876	293.1228	3.0733
Corymboside	0.0001627	0.0006007	1.0228	0.7646	-0.3872	down	LevelBi	HMDB0030803;LMPK12110260	565.1507	3.1925
1-[(3-Phenyl-3-Cyclohexenyl)Methyl]-4-Phenyl-1,2,3,6-Tetrahydropyridine	0.005837	0.01095	1.0382	1.598	0.6762	up	LevelBii	HMDB0260239	374.2096	3.6657
Phe-Thr	0.000272	0.0008601	1.0223	1.2458	0.3171	up	LevelBi	HMDB0029005	267.1332	2.1311
13(S)-Hydroperoxylinolenic Acid	0.002504	0.005319	1.0278	0.7379	-0.4385	down	LevelBii	HMDB0301803	309.2054	6.6189
Ile-Val-Val	7.528E-5	0.000322	1.0163	1.2952	0.3732	up	LevelBii	HMDB0304801	328.2226	3.1301
N-Linoleoyl Glutamine	8.684E-5	0.00036	1.0115	1.2583	0.3314	up	LevelBii	HMDB0241975	447.2588	2.2904
Desacetyl-Alacepril	0.02485	0.03794	1.0657	4.0223	2.008	up	LevelBii	HMDB0251027	385.1206	5.0198
Pro-Val-Leu	1.351E-6	1.833E-5	1.0151	1.2416	0.3122	up	LevelBi	-	328.2216	2.915
Crenolanib	0.0002417	0.0007827	1.0184	1.2877	0.3648	up	LevelBii	HMDB0250534	442.2288	2.3777
S-(9-Deoxy-Delta9,12-Pgd2)-Glutathione	0.01212	0.02041	1.0206	1.3374	0.4195	up	LevelBii	HMDB0013058	662.2682	7.8848
Penitrem D	0.0191	0.03023	1.0291	2.2832	1.191	up	LevelBii	HMDB0256240	612.3353	3.196
1,1'-(Phenylazanediyl)Dipropan-2-Ol	3.088E-5	0.0001537	1.0124	1.2477	0.3193	up	LevelBi	-	227.1744	2.3602
Hophe-Trp-Oh	0.001056	0.002605	1.0161	1.9333	0.9511	up	LevelBi	-	472.1565	2.1976
2-Hydroxy-2-Phenylpropionamide	0.0007683	0.002012	1.0102	1.5139	0.5983	up	LevelBi	-	130.0636	5.6053
4,17-Dimethyltrilostane	2.667E-6	3.047E-5	1.0079	1.3299	0.4113	up	LevelBii	HMDB0242688	378.2021	3.4613
Amastatin	0.01188	0.02004	1.0107	0.4623	-1.1131	down	LevelBii	HMDB0248277	455.255	9.2981
Kaempferol 3-O-Alpha-L-Rhamnofuranoside	0.005309	0.0101	1.0298	0.6572	-0.6056	down	LevelBii	HMDB0040542	431.0953	4.5003
Mimosine	0.008078	0.01438	1.0284	1.6417	0.7152	up	LevelBii	HMDB0015188	243.0609	1.8648
Gly-Lys-Phe	0.01703	0.02736	1.0349	1.3901	0.4751	up	LevelBi	-	351.2026	1.9026
Silodosin	0.007551	0.0136	1.0171	1.4208	0.5067	up	LevelBii	HMDB0258291	516.2137	3.7495
Car(5:0)	0.0003654	0.001094	1.0083	1.2766	0.3523	up	LevelBi	-	246.1696	2.4775
Physagulin G	0.0005648	0.001562	1.0101	0.5681	-0.8159	down	LevelBii	HMDB0039694	703.3015	2.1593
Neoandrographolide	0.0004867	0.001383	1.0014	1.3236	0.4045	up	LevelBii	HMDB0255516	519.2379	2.3204
2'-Fluoro O-Fluorofentanyl	0.0002118	0.0007364	1.0047	0.7436	-0.4274	down	LevelBi	-	373.2087	8.5723
3-Ethenylphenol	6.489E-5	0.0002849	0.9997	0.8662	-0.2072	down	LevelBi	-	121.0646	1.8633
Dienogest	0.002134	0.004653	0.9953	1.4408	0.5269	up	LevelBii	HMDB0251234	346.1598	2.5383
Caffeic Acid	2.509E-8	9.531E-7	1.0082	0.6876	-0.5403	down	LevelBii	HMDB0001964	179.0336	3.2431
3A,6B,7A,12A-Tetrahydroxy-5B-Cholanoic Acid	0.02315	0.03573	1.0475	0.6444	-0.6339	down	LevelBii	HMDB0000399	447.2718	6.6123
Galactitol	0.0002543	0.0008137	1.0209	0.627	-0.6734	down	LevelBii	HMDB0000107	181.0708	0.671
Phytosphingosine-1-P	0.0002431	0.0007865	0.9937	1.1838	0.2434	up	LevelBii	HMDB0012280	415.2962	8.9216
Fahfa(20:3/22:3)	0.01089	0.01856	1.0125	0.7308	-0.4525	down	LevelBi	-	637.5167	8.9731
Hexadecanedioic Acid	0.004	0.008	1.011	0.700	-0.513	down	Leve	HMDB0000672	285.2	7.3405

Metabolite	P_value	FDR	VIP_PLS-DA	FC(A/E)	Log2FC(A/E)	Regulate	level	Library ID	M/Z	Retention time
	402	605	3	5	6		lBii		057	
Paliperidone	0.0001612	0.0005963	0.9931	0.6968	-0.5212	down	LevelBii	HMDB0015396	447.1824	2.0631
Ascofuranone	2.252E-5	0.0001187	0.9999	0.7421	-0.4302	down	LevelBii	HMDB0248651	385.1586	4.0796
Ile-Hyp	0.001533	0.003542	0.989	0.7743	-0.369	down	LevelBii	HMDB0028908	245.1486	2.1112
16-Acetylpriverogenin A	0.004957	0.009528	1.006	1.2484	0.3201	up	LevelBii	HMDB0034529	514.3633	9.1283
N-Trans-Feruloyltyramine	6.426E-5	0.0002824	0.9938	0.7416	-0.4312	down	LevelBii	HMDB0029365	312.1222	5.6277
Gly-Leu-Phe	6.0E-5	0.0002677	0.9955	1.2395	0.3097	up	LevelBi	-	336.1898	3.5573
Tricoumaroyl Spermidine	0.005552	0.01049	1.0074	1.3623	0.446	up	LevelBii	HMDB0304518	628.2671	1.8555
Lpe(18:2)	0.003739	0.007482	0.993	1.175	0.2326	up	LevelBi	HMDB0011477	478.2908	7.7446
Ser-Ile-Leu	9.353E-5	0.0003824	0.9919	1.2501	0.322	up	LevelBi	-	332.2164	2.7762
Leu-Ile-Ala	0.0004255	0.001238	0.9954	1.2575	0.3306	up	LevelBi	-	316.2215	2.5469
3-(4-Methylphenyl)-2-Propenenitrile	0.0001455	0.00055	0.9865	0.8754	-0.1919	down	LevelBi	-	144.0808	2.4099
Leu-Met	2.032E-5	0.0001091	0.9916	1.2026	0.2661	up	LevelBi	HMDB0028935	263.1412	2.2806
Val-Phe-Arg	0.001983	0.004382	0.997	1.2802	0.3563	up	LevelBi	-	421.2523	2.3204
Betaine	0.002667	0.005619	0.9995	1.296	0.374	up	LevelBii	HMDB0000043	237.1831	7.4132
Dihydroergocristine	0.0003652	0.001093	0.9957	1.3289	0.4103	up	LevelBii	HMDB0251309	634.3044	7.6097
Ile-Lys	0.0003437	0.001043	0.9936	1.2905	0.368	up	LevelBii	HMDB0028912	258.1814	1.0538
Isopentyl Isopentanoate	0.0003926	0.001159	0.997	0.578	-0.7908	down	LevelBii	HMDB0030029	231.1588	4.5557
Abu-Lys-Oh	0.003213	0.006584	1.0189	1.976	0.9825	up	LevelBi	-	338.1327	2.0441
Leu-Ile-Thr	0.0005259	0.001474	0.9933	1.2206	0.2875	up	LevelBi	-	346.2318	2.4679
Gly-Val-Phe	0.003757	0.007512	1.0002	1.2691	0.3438	up	LevelBi	-	322.1747	3.0635
1-O-Hexadecyl-Sn-Glycero-3-Phosphocholine	0.0001692	0.0006186	0.9855	0.889	-0.1698	down	LevelBii	HMDB0243890	482.3586	8.3392
Trp-Met	0.004258	0.008353	1.0049	1.2997	0.3782	up	LevelBi	HMDB0029089	336.1362	2.9844
Wedelolide A	1.466E-5	8.33E-5	0.988	1.3416	0.424	up	LevelBi	-	465.2462	4.5602
Lps(22:5)	4.196E-5	0.0001979	0.9852	1.2483	0.3199	up	LevelBi	-	570.2816	8.5757
Lps(18:2)	0.001538	0.00355	0.9915	1.1985	0.2613	up	LevelBi	HMDB0240604	520.2657	8.5195

Table S2 Statistics of Differential Metabolites between HNSM and LSM

Metabolite	P_value	FDR	VIP_PLS-DA	FC(B/E)	Log2FC(B/E)	Regulate	level	Library ID	M/Z	Retention time	Score
Pi(13:0/16:4)	1.515E-7	1.853E-6	2.3789	0.047	-4.4098	down	LevelBi	-	805.4202	6.4339	52.7
8-Deoxy-11-Hydroxy-13-Chlorogrosheimin	1.501E-6	1.048E-5	2.3724	0.0523	-4.2572	down	LevelBii	HMDB0041037	357.114	2.8403	49.9
Cyclohexylsulfamate	4.518E-7	4.349E-6	2.3454	0.2086	-2.2612	down	LevelBi	HMDB0031340	178.0541	2.8592	49.6
Kanamycin	1.255E-7	1.596E-6	2.2918	0.0043	-7.8623	down	LevelBii	HMDB0015303	967.4702	5.7845	49.3
Lyciumoside Iv	4.009E-11	8.723E-9	2.2562	0.0029	-8.4494	down	LevelBii	HMDB0033499	821.415	5.462	53.4
Pinolidoxin	1.093E-8	2.624E-7	2.2364	0	-14.9304	down	LevelBii	HMDB0301836	359.1483	4.0277	54.1
17-Hydroxy-15-Kauren-19-Oic Acid	9.593E-8	1.296E-6	2.2017	0.0236	-5.4056	down	LevelBi	-	301.2143	5.8592	47.3
Protoporphyrin Ix	1.466E-6	1.048E-5	2.1952	0.0496	-4.3324	down	LevelBii	HMDB0000241	1123.5138	6.8225	43.5
Isovitexin 2''-O-(6'''-Feruloyl)Glucoside	2.534E-7	2.783E-6	2.1872	0.0048	-7.703	down	LevelBii	HMDB0038042	769.1958	4.139	46.4
Emodin	3.539E-6	1.993E-5	2.1846	0.0321	-4.9603	down	LevelBi	HMDB0035214;LMPK13040008	269.0449	5.2503	48.6
Tenatoprazole	3.005E-9	1.104E-7	2.1765	0.0534	-4.2265	down	LevelBii	HMDB0258812	737.2202	2.8683	47.2
(7'R,8'R)-4,7'-Epoxy-3',5-Dimethoxy-4',9,9'-Lignanetriol 9'-Glucoside	3.783E-9	1.29E-7	2.169	0.0076	-7.046	down	LevelBii	HMDB0038711	521.2002	3.4425	50.2
4-Demethylsimmondsin 2'-(E)-Ferulate	4.774E-10	3.344E-8	2.1492	0.0595	-4.0719	down	LevelBii	HMDB0034773	558.1572	2.8683	50.8
Isoeruboside B	1.403E-5	6.311E-5	2.1428	0.2216	-2.1738	down	LevelBii	HMDB0029769	1125.5302	7.044	52.1
Neogitogenin 3-[Glucosyl-(1->2)-Glucosyl-(1->4)-Galactoside]	3.01E-6	1.744E-5	2.1161	0.1912	-2.3865	down	LevelBii	HMDB0041134	963.4787	7.1181	52.8
Vanillyl Alcohol	1.4E-5	6.304E-5	2.1118	0.0724	-3.7881	down	LevelBi	HMDB0032012	137.0591	6.3883	56.6
Apigenin	1.459E-5	6.513E-5	2.0915	0	-14.9916	down	LevelBi	HMDB0002124;LMPK12110005	271.0596	5.2537	57.8
Araloside A	3.176E-5	0.0001243	2.0807	0.0725	-3.7865	down	LevelBii	HMDB0034535	961.4617	6.9054	50.8
Cdp-Dg(Lte4/I-18:0)	0.0001532	0.0004669	2.0712	0.117	-3.0957	down	LevelBii	HMDB0293917	1145.5212	5.1303	52
Pip(Pgf1Alpha/16:2(9Z,12Z))	9.27E-7	7.573E-6	2.0702	0.0994	-3.33	down	LevelBii	HMDB0278720	1031.466	7.1273	50

Norcisapride	2.802E-8	5.206E-7	2.0606	0.1322	-2.9187	down	LevelBii	HMDB0061018	671.2319	3.9904	41.3
Capsianoside Ii	3.464E-7	3.52E-6	2.0594	0.144	-2.7957	down	LevelBii	HMDB0031892	1085.5305	5.4492	47.4
Jujuboside B	5.033E-7	4.715E-6	2.0537	0.1357	-2.8813	down	LevelBii	HMDB0035745	1079.5244	7.5737	46.3
N-Fructosyl Tyrosine	3.247E-11	7.694E-9	2.0524	0.0045	-7.8078	down	LevelBi	-	344.1321	1.8729	46.5
23-O-Beta-D-Glucopyranosyl-25-Methyldolichosterone	4.301E-10	3.201E-8	2.0472	63.2897	5.9839	up	LevelBii	HMDB0301798	677.3672	8.0379	44.7
Dihydrocapsaicin	1.281E-5	5.84E-5	2.0479	0.1951	-2.358	down	LevelBi	HMDB0038457;LMFA08020318	308.2215	6.7388	54.8
Ganolucidic Acid C	9.417E-6	4.527E-5	2.0362	13.1515	3.7172	up	LevelBii	HMDB0039691	551.3617	2.6169	44
1-Ribityl-6,7-Dimethyl-1,2,3,4-Tetrahydroquinoxalin-2,3-Dione	1.41E-10	1.745E-8	2.0276	0.0385	-4.6987	down	LevelBi	-	325.1378	3.133	49.2
6-Carboxy-5,6,7,8-Tetrahydropterin	3.154E-12	2.384E-9	2.0273	0.1245	-3.0054	down	LevelBii	HMDB0060410	246.0396	2.9046	54
N-(1-Deoxy-1-Fructosyl)Phenylalanine	3.852E-9	1.305E-7	2.0256	0.0244	-5.3562	down	LevelBii	HMDB0037846	326.1233	2.1593	54.2
Malvidin 3-(6''-Acetyl-Galactoside)	7.199E-10	4.344E-8	2.0183	0.1231	-3.0225	down	LevelBii	HMDB0301662	580.1383	2.8865	47
Fumonisin A1	4.058E-6	2.239E-5	2.0077	0.0327	-4.9351	down	LevelBii	HMDB0034713	822.4102	6.4339	46
Cinncassiol D4 2-Glucoside	1.424E-9	6.795E-8	1.9903	0.143	-2.8062	down	LevelBii	HMDB0035065	559.2727	4.8811	50.6
1-Myristoyl-2-Hydroxy-Sn-Glycero-3-Phosphoethanolamine	1.332E-8	3.007E-7	1.9885	4.6116	2.2053	up	LevelBi	HMDB0011500	426.2593	7.5209	56.5
2Alpha-Methyl-5Alpha-Androstan-3Alpha-Ol-17-One	2.846E-6	1.663E-5	1.9878	0.1035	-3.272	down	LevelBi	-	269.2252	6.4371	50.3
Picrocrocin	3.185E-8	5.696E-7	1.9825	0.0038	-8.0512	down	LevelBii	HMDB0005796	705.3302	5.7015	47.3
Capsaicin	1.366E-5	6.168E-5	1.9814	0.3227	-1.6316	down	LevelBi	HMDB0002227;HMDB0302729;LMFA08020085	306.2059	6.3883	58.4
Phaseollinisoflavan	6.483E-7	5.714E-6	1.9721	0.0541	-4.2073	down	LevelBii	HMDB0034411	325.142	4.0206	47.8
N-Fructosyl Phenylalanine	2.004E-11	6.188E-9	1.9688	0.2168	-2.2056	down	LevelBi	-	328.138	2.1616	51.5
N-Acetylneuraminic Acid	8.438E-11	1.287E-8	1.9658	0.1385	-2.8518	down	LevelBii	HMDB0000230	290.0868	1.6041	48.8
Gm3(30:2_2O)	4.242E-5	0.0001586	1.9594	0.1361	-2.8777	down	LevelBi	-	1139.6317	7.0625	47.2
Gitonin	7.684E-6	3.811E-5	1.9582	0.2125	-2.2348	down	LevelBii	HMDB0301779	1109.5349	7.5177	48.2

Phenylacetylglutamine	1.338E-10	1.685E-8	1.9513	0.0753	-3.7319	down	LevelBii	HMDB0006344	323.1235	3.1301	51.1
Loquatifolin A	2.319E-6	1.416E-5	1.9455	0.1731	-2.5305	down	LevelBii	HMDB0030434	821.4139	6.0435	50.3
Gibberellin A3	8.695E-6	4.245E-5	1.9462	0.1488	-2.749	down	LevelBii	HMDB0003559	391.1368	3.6382	44.9
17(18)-Epoxy-5Z,8Z,11Z,14Z-Eicosatetraenoic Acid	4.115E-6	2.266E-5	1.9307	0.2182	-2.1963	down	LevelBi	HMDB0010212	319.2255	5.8592	54.3
Etiocholanolone Glucuronide	0.0005585	0.001408	1.9324	4.145	2.0514	up	LevelBii	HMDB0004484	508.2924	3.6363	46.8
Fructosylvaline	1.465E-10	1.798E-8	1.9281	0.0008	-10.2935	down	LevelBii	HMDB0252498	278.1238	1.156	54.8
Dgdg(O-18:5/6:0)	1.132E-5	5.267E-5	1.9239	0.0018	-9.1527	down	LevelBi	-	801.4241	7.3685	46.4
Fa(18:3)	1.508E-6	1.048E-5	1.9232	0.1758	-2.5077	down	LevelBi	-	555.4375	8.4549	48.7
Fisetin	2.592E-5	0.000106	1.9225	0.0638	-3.9711	down	LevelBi	LMPK12111566	285.0393	4.8996	51.8
Dihydroouabain	7.236E-8	1.068E-6	1.9216	4.2733	2.0953	up	LevelBii	HMDB0251322	641.3158	2.6369	46
Meticillin	5.663E-9	1.653E-7	1.9218	0.3511	-1.51	down	LevelBii	HMDB0015541	379.0959	2.8775	48.3
Trans-3'-Hydroxycotinine O-Beta-D-Glucuronide	4.482E-8	7.315E-7	1.9184	0.0641	-3.9628	down	LevelBii	HMDB0341403	427.1347	4.0277	46.9
Milbemycin Beta1	8.147E-6	4.008E-5	1.914	0.013	-6.2701	down	LevelBii	HMDB0254727	608.3514	6.7388	43.6
1,2-Diphenylhydrazine	3.701E-7	3.704E-6	1.9109	0.0631	-3.9866	down	LevelBi	-	217.1321	2.9844	51
Gingerglycolipid B	0.0001813	0.0005358	1.9069	0.2148	-2.219	down	LevelBii	HMDB0041094	659.3616	6.4618	53.5
(3Beta,5Alpha,6Beta,9Alpha,22E,24R)-23-Methylergosta-7,22-Diene-3,5,6,9-Tetrol	1.239E-5	5.673E-5	1.9035	3.6682	1.8751	up	LevelBii	HMDB0033634	499.3203	2.2409	47.5
Chenodeoxycholyltryptophan	0.0001819	0.0005372	1.9045	0.2195	-2.1874	down	LevelBii	HMDB0242395	633.3839	6.3883	49
Carbadox	1.711E-7	2.053E-6	1.8944	0.0025	-8.663	down	LevelBii	HMDB0031762	307.0707	4.7218	45.7
(3Ar,4S,7S,7As)-3A,4,5,6,7,7A-Hexahydro-1H-4,7-Methanoinden-6-Yl Propionate	8.327E-7	6.969E-6	1.8943	0.0245	-5.3529	down	LevelBi	-	207.1372	5.8592	51.6
Fludrocortisone Acetate	7.652E-12	3.925E-9	1.8935	26.5514	4.7307	up	LevelBii	HMDB0252329	440.2447	4.1978	46.4
Tuberoside B (Ullucus Tuberosus)	1.293E-10	1.658E-8	1.8843	12.1483	3.6027	up	LevelBii	HMDB0031027	1069.5227	3.2337	45.3
S-(Formylmethyl)Glutathione	2.833E-12	2.384E-9	1.8807	0.1984	-2.3335	down	LevelBii	HMDB0060507	314.0829	1.6103	43.4

Carmine Red	1.797E-7	2.133E-6	1.875	0.0386	-4.6959	down	LevelBii	HMDB0030658	513.0674	4.2781	53.8
Tylosin	1.749E-8	3.622E-7	1.8739	7.2115	2.8503	up	LevelBii	HMDB0034108	936.4894	2.2926	47.7
Proscillaridin	1.521E-8	3.305E-7	1.8696	0.0781	-3.6793	down	LevelBii	HMDB0256851	565.2597	5.8957	43.8
4,4-Dimethyl-2-[3-Carboxylatopropyl]-2-Tridecyloxazolidine 3-Oxide	6.85E-7	5.951E-6	1.867	0.1928	-2.3748	down	LevelBii	HMDB0246830	366.2991	8.5851	47
Ceanothine B	0.0003184	0.0008695	1.8663	4.4337	2.1485	up	LevelBii	HMDB0029339	568.2915	4.2569	43.5
Lpe(30:8)	5.981E-10	3.834E-8	1.8524	14.9404	3.9011	up	LevelBi	-	678.3789	2.9885	45.7
Leukotriene D4	4.402E-5	0.0001637	1.8484	0.2552	-1.9702	down	LevelBii	HMDB0003080	541.2626	5.2412	50.3
1-(2,6-Dihydroxy-4-Methoxyphenyl)-3-Phenyl-1-Propanone	4.966E-7	4.671E-6	1.8467	0	-18.457	down	LevelBii	HMDB0034435	331.1169	4.0648	46.9
Esculetin	2.452E-8	4.739E-7	1.8403	0.1304	-2.9387	down	LevelBi	HMDB0030819	177.0185	2.9885	46.8
Hypoglycin B	1.174E-6	9.135E-6	1.8396	0.1949	-2.3589	down	LevelBii	HMDB0029428	309.0856	4.7167	50.3
Mocimycin	0.0008457	0.002004	1.8442	5.0239	2.3288	up	LevelBii	HMDB0030465	797.4239	3.3112	44.4
Ubiquinone-1	2.33E-7	2.598E-6	1.8296	0.0016	-9.2675	down	LevelBii	HMDB0002012	314.1369	4.4039	49.7
gamma-Glu-Cys-Gly	3.226E-8	5.74E-7	1.8284	2.5678	1.3605	up	LevelBi	-	308.0903	1.2553	53.5
Amprenavir	1.191E-9	5.978E-8	1.8225	0.1919	-2.3814	down	LevelBii	HMDB0014839	1055.4407	3.602	41.9
Lnape(16:3/N-5:0)	9.443E-10	5.118E-8	1.8203	12.605	3.6559	up	LevelBi	-	576.299	2.9979	50
Lpc(19:1)	8.03E-6	3.959E-5	1.8191	5.2816	2.401	up	LevelBi	LMGP01050130	580.3593	8.309	47.3
Tangeraxanthin	8.411E-6	4.123E-5	1.8171	10.9218	3.4491	up	LevelBii	HMDB0039015	505.3056	8.3635	46.7
5-Pentyltetrahydro-2-Oxo-3-Furancarboxylic Acid	1.031E-7	1.376E-6	1.8137	0.1862	-2.4248	down	LevelBii	HMDB0030989	221.0784	4.7981	50.2
Pa(Pgf2Alpha/2:0)	3.448E-5	0.0001335	1.812	10.0515	3.3293	up	LevelBii	HMDB0266516	568.2861	3.3308	42
Diisodityrosine	8.558E-8	1.205E-6	1.8072	0.1964	-2.3484	down	LevelBii	HMDB0029798	739.2182	3.9904	48.8
Miglitol	6.485E-9	1.825E-7	1.8081	0.0875	-3.5152	down	LevelBii	HMDB0014634	188.0919	2.7083	48.7
Flavone Base+3O,C-Hex-Feruloylhex	7.723E-6	3.827E-5	1.8062	0.2901	-1.7854	down	LevelBi	-	771.2079	4.1388	44.4

Tragopogonsaponin D	3.744E-6	2.091E-5	1.8058	0.2587	-1.9505	down	LevelBii	HMDB0037915	1099.5133	5.6277	51
Penitrem B	7.406E-10	4.432E-8	1.8042	21.408	4.4201	up	LevelBii	HMDB0256239	642.3448	3.4425	46.8
Cenisertib	3.544E-5	0.0001366	1.8067	11.7144	3.5502	up	LevelBii	HMDB0249799	452.253	2.8357	43.3
Beta-D-Glucosaminyl-(1->4)-Beta-D-Glucosamine	6.674E-8	1.001E-6	1.8033	0.0765	-3.7077	down	LevelBii	HMDB0249120	305.1332	1.1771	47.9
Lpe(14:0)	0.0001399	0.0004329	1.7983	2.9368	1.5543	up	LevelBi	HMDB0011470	424.2451	7.5177	53.4
Tenuazonic Acid	3.047E-8	5.506E-7	1.7989	0.037	-4.7575	down	LevelBi	HMDB0036074	198.1115	4.9511	47.1
Dlcl(16:4/22:7)	2.253E-6	1.383E-5	1.799	0.0735	-3.7655	down	LevelBi	-	983.4332	4.8351	43.5
Marinobufagenin	1.893E-5	8.113E-5	1.7944	15.543	3.9582	up	LevelBii	HMDB0254337	421.1989	7.9873	47.6
Hydroxygaleon	3.13E-7	3.255E-6	1.7955	0.2371	-2.0767	down	LevelBii	HMDB0031535	343.152	4.0206	48.8
9-Hydroxy-3,4-Dihydroacridin-1(2H)-One	2.473E-9	9.913E-8	1.7954	0.0455	-4.4591	down	LevelBi	-	214.0848	3.1428	48.1
20-Hydroxy-Leukotriene B4	3.064E-12	2.384E-9	1.795	94.0959	6.5561	up	LevelBii	HMDB0001509	397.2214	4.2411	51.4
Clindamycin	9.342E-7	7.622E-6	1.7923	0.2653	-1.9142	down	LevelBii	HMDB0015321	425.1893	2.915	42.4
Tryptoline	5.013E-9	1.545E-7	1.791	0.0959	-3.3823	down	LevelBi	HMDB0012488	173.1065	2.8555	54.6
Histamine	4.437E-8	7.255E-7	1.7911	0.3217	-1.6364	down	LevelBi	HMDB0000870;PW_C000693	112.0865	0.6298	58.1
Tryptophan N-Glucoside	0.0004119	0.001082	1.7835	0.1968	-2.3449	down	LevelBii	HMDB0341443	367.1483	2.3402	51.1
2-Cyclopropyl-4-Quinolinecarboxylic Acid	1.734E-9	7.723E-8	1.7853	0.2009	-2.3157	down	LevelBi	-	214.0853	2.9844	47.6
Neoacrimarine A	2.098E-7	2.395E-6	1.7845	6.5212	2.7051	up	LevelBii	HMDB0040384	716.2617	7.443	44.3
Fahfa(20:4/6:0)	7.036E-5	0.0002429	1.779	0.1213	-3.0434	down	LevelBi	-	417.2982	9.121	52.3
Genistein 4'-O-Glucoside	9.953E-6	4.728E-5	1.7781	0.1204	-3.0544	down	LevelBi	HMDB0252680;LMPK12050168	431.0955	4.139	47.8
Dehydroisoandrosterone 3-Glucuronide	9.604E-7	7.801E-6	1.7741	0.1747	-2.517	down	LevelBii	HMDB0010327	497.2706	4.8829	45.8
Gallopamil	2.309E-5	9.615E-5	1.7726	4.9405	2.3046	up	LevelBii	HMDB0252599	529.2887	7.443	46.1
1-Myristoyl-Sn-Glycero-3-Phosphocholine	1.616E-5	7.11E-5	1.7742	1.9577	0.9692	up	LevelBi	HMDB0010379	468.3063	7.4429	51.9

Pesticide4_Ivermectin_C48H74O14_22,23-Dihydroavermectin B1	9.315E-5	0.0003079	1.7727	3.2705	1.7095	up	LevelBii	HMDB0247302	839.4932	4.1288	41.7
2,6,6-Trimethyl-3-Oxocyclohexene-1-Carboxylic Acid	4.012E-9	1.34E-7	1.7694	0.004	-7.9572	down	LevelBi	-	183.1003	4.7951	49.1
Zearalanone	1.537E-7	1.874E-6	1.7651	0.0095	-6.7223	down	LevelBii	HMDB0259993	341.1366	4.0277	53.5
Pgp(Pge2/I-14:0)	1.534E-6	1.048E-5	1.7589	0.0028	-8.5006	down	LevelBii	HMDB0275018	905.3961	6.1478	45
Bn-Ncc-2	1.161E-5	5.382E-5	1.76	0.045	-4.4749	down	LevelBii	HMDB0038230	837.3169	4.0091	45.9
Dgdg(O-16:3/2:0)	2.81E-9	1.06E-7	1.7583	8407.356	13.0374	up	LevelBi	-	721.3654	7.3964	44.9
N-Palmitoyl Gaba	1.564E-5	6.898E-5	1.7551	0.2261	-2.1452	down	LevelBii	HMDB0062338	340.2829	8.5103	48.3
Hovenoside D	1.005E-5	4.766E-5	1.7543	5.7792	2.5309	up	LevelBii	HMDB0035746	1191.5889	2.9979	41.9
Delta-Valerolactone	3.688E-7	3.696E-6	1.7468	0.2772	-1.851	down	LevelBii	HMDB0250981	199.0967	4.7981	48.4
Convalloside	7.265E-6	3.647E-5	1.7489	0.1564	-2.6765	down	LevelBii	HMDB0034195	711.3171	6.2492	45.6
9-((2-Hydroxyethoxy)Methyl)Hypoxanthine	0.0002272	0.0006508	1.7454	0.307	-1.7036	down	LevelBi	-	211.0819	1.9821	50.7
3-Phenylpropyl Acetate	7.421E-8	1.086E-6	1.7462	0.0031	-8.3253	down	LevelBii	HMDB0033386	223.096	5.6553	55.5
Kievitone Hydrate	4.114E-5	0.0001549	1.7452	0.0922	-3.4394	down	LevelBii	HMDB0038111	373.127	4.2596	46.4
Ser-Ser	6.018E-10	3.841E-8	1.7437	0.0013	-9.5626	down	LevelBii	HMDB0029048	383.143	2.2829	47.7
Phe-Hyp	1.0E-6	8.048E-6	1.7406	0.2209	-2.1786	down	LevelBii	HMDB0011176	261.1224	3.2023	47.3
Stearidonic Acid Ethyl Ester	7.031E-5	0.0002428	1.7412	0.2444	-2.0325	down	LevelBi	-	305.2458	7.6196	45.1
Smgdg(O-13:1/7:0)	5.992E-5	0.0002132	1.7395	0.0689	-3.8603	down	LevelBi	-	671.3259	6.9054	50.5
(S)-3-Hydroxy-N-Methylcoclaurine	3.43E-7	3.496E-6	1.7353	0.0538	-4.2171	down	LevelBii	HMDB0006921	316.1536	4.0598	45
Androsterone Glucuronide	0.0001061	0.0003438	1.7379	0.2827	-1.8227	down	LevelBii	HMDB0002829	525.2677	6.6005	47.8
Magnolol	0.0001448	0.0004453	1.7384	0.0329	-4.9257	down	LevelBi	HMDB0254305	265.1217	7.0719	43.7
Thiopalmitic Acid	0.001712	0.003684	1.7301	2.4014	1.2639	up	LevelBii	HMDB0259021	331.2305	7.978	47.1
7(14)-Bisabolene-2,3,10,11-Tetrol	3.27E-8	5.787E-7	1.7334	0.1029	-3.2803	down	LevelBii	HMDB0035918	317.1952	4.8996	46.9

Cdp-Dg(Pgf2Alpha/22:5(4Z,7Z,10Z,13Z,16Z))	1.513E-6	1.048E-5	1.7333	0.3147	-1.6681	down	LevelBii	HMDB0292458	1146.516	5.4527	48.3
Apo-12'-Zeaxanthinal	2.255E-8	4.448E-7	1.7324	0.1764	-2.5031	down	LevelBii	HMDB0302023	347.2417	4.8442	44.1
N-Monodemethyl Roxithromycin	2.022E-8	4.073E-7	1.7326	3.0971	1.6309	up	LevelBii	HMDB0060845	791.4929	7.6677	45.9
Cyclocalamin	2.284E-7	2.561E-6	1.7284	0.4335	-1.206	down	LevelBii	HMDB0035863	537.1916	2.1688	49.3
Licoricesaponin J2	4.309E-6	2.357E-5	1.7294	3.106	1.635	up	LevelBi	HMDB0039448	825.4205	3.0833	41.5
N-(1-Deoxy-1-Fructosyl)Proline	9.167E-7	7.497E-6	1.7263	0.179	-2.4822	down	LevelBii	HMDB0038493	278.1215	0.737	49.9
3-Hydroxynevirapine Glucuronide	3.603E-9	1.247E-7	1.7234	0.1703	-2.554	down	LevelBii	HMDB0060743	479.1177	4.3429	55.3
11Beta-Hydroxytestosterone	1.054E-5	4.955E-5	1.7219	0.2374	-2.0745	down	LevelBii	HMDB0012533	349.1997	5.8494	46.4
Vinyl Ether	2.787E-7	2.978E-6	1.7241	0.0413	-4.5967	down	LevelBii	HMDB0259824	185.0807	5.3422	47.4
Torvonin A	6.339E-6	3.257E-5	1.7217	0.2613	-1.936	down	LevelBii	HMDB0030732	759.414	6.4339	43.9
25-Hydroxyvitamin D3-26,23-Lactone	0.0001252	0.0003942	1.723	4.8323	2.2727	up	LevelBii	HMDB0060126	451.2811	2.7262	48.1
(6E)-Heptadeca-6,16-Diene-1,2,4-Triol	0.0003817	0.001013	1.726	2.172	1.119	up	LevelBi	-	317.2677	7.1087	47.7
Alpha-Tocopheryloxyacetic Acid	0.0003753	0.001001	1.7167	0.1709	-2.5487	down	LevelBii	HMDB0248242	506.4175	7.7057	44.4
Ps(Pgf2Alpha/20:5(5Z,8Z,11Z,14Z,17Z))	8.587E-10	4.817E-8	1.7161	4.6232	2.2089	up	LevelBii	HMDB0282804	884.4665	4.1189	48.2
Apiin	3.432E-6	1.945E-5	1.7143	0.1976	-2.3392	down	LevelBi	HMDB0030843;LMPK12110337	565.1509	3.991	52.9
Pi(6 Keto-Pgf1Alpha/18:3(6Z,9Z,12Z))	0.0005869	0.001468	1.7204	0.1729	-2.5319	down	LevelBii	HMDB0276784	981.466	6.6929	51.2
Pengitoxin	1.202E-5	5.539E-5	1.7121	6.4994	2.7003	up	LevelBii	HMDB0256225	1011.4531	2.2735	45.2
Concanamycin	1.16E-5	5.377E-5	1.7086	4.3054	2.1062	up	LevelBii	HMDB0250429	751.4694	3.2148	44.9
17-Debromo-Aplysiatoxin,	3.369E-5	0.0001306	1.705	4.1746	2.0616	up	LevelBii	HMDB0248517	627.2984	2.4348	44.3
Halometasone	2.911E-7	3.094E-6	1.7047	0.0365	-4.7775	down	LevelBii	HMDB0253041	479.1176	4.8627	48.6
Destruxin E	3.736E-9	1.283E-7	1.7038	12.7123	3.6682	up	LevelBii	HMDB0251067	628.308	2.5476	51.7
14-Hdohe	2.961E-5	0.0001176	1.7011	0.0951	-3.3941	down	LevelBii	HMDB0060044	391.2059	2.4066	47.7

Pgp(Pgf2Alpha/22:6(4Z,7Z,10Z,13Z,16Z,19Z))	1.249E-6	9.575E-6	1.7018	0.1093	-3.1935	down	LevelBii	HMDB0274193	977.4479	5.322	41.1
Hetisine	3.098E-5	0.0001217	1.7027	0.4017	-1.3158	down	LevelBi	-	330.2035	6.7388	47.7
4-(2,2,6,6-Tetramethyl-1-Oxylpiperidin-4-Ylamino)-4'-Demethylepipodophyllotoxin	3.11E-7	3.246E-6	1.6982	0.426	-1.2309	down	LevelBii	HMDB0252920	537.2621	4.7364	45.7
Phenylbutyric Acid	6.329E-5	0.0002226	1.6937	0.1455	-2.7806	down	LevelBi	-	165.09	4.7951	49.5
(R)-Dihydroactinidiolide	1.596E-8	3.407E-7	1.6954	0.2949	-1.7615	down	LevelBi	-	181.1216	5.3904	49.3
Cdp-Dg(Pgf2Alpha/18:1(11Z))	2.709E-8	5.106E-7	1.6935	22.3954	4.4851	up	LevelBii	HMDB0291106	1122.5379	3.527	46.5
1,2-Cyclohexanedicarboxylic Acid, 4-Methyl-	3.013E-10	2.655E-8	1.6939	0.1509	-2.7281	down	LevelBii	HMDB0253138	204.1219	3.5573	47.2
15(S)-Hpete	1.515E-8	3.304E-7	1.6909	0.0074	-7.0689	down	LevelBii	HMDB0004244	371.2025	4.9828	44.5
Matairesinol	0.0002046	0.0005951	1.6928	0.1701	-2.5557	down	LevelBii	HMDB0035698	357.1324	4.2873	50.7
Coroloside	4.311E-6	2.357E-5	1.6907	3.1554	1.6578	up	LevelBii	HMDB0033703	699.3896	3.6068	45.8
Nona-5,7-Dienoic Acid	1.82E-9	7.967E-8	1.6881	0.1832	-2.4484	down	LevelBii	HMDB0341026	137.0953	4.7951	47.9
N-(2-Hydroxy-3-Methylbutanoyl)Leucine	4.207E-6	2.309E-5	1.6901	0.1537	-2.7014	down	LevelBi	-	230.1385	5.0382	45.3
Steviobioside	7.663E-5	0.0002618	1.6914	0.2257	-2.1473	down	LevelBi	HMDB0036707	641.3133	6.0435	46.6
Cilengitide	1.418E-5	6.357E-5	1.6865	0.2869	-1.8016	down	LevelBii	HMDB0250242	606.3357	6.398	49.2
Alpha-Cyperone	8.799E-5	0.0002933	1.6877	0.1811	-2.4648	down	LevelBi	-	219.174	6.447	51.3
Fluphenazine Decanoate	3.621E-8	6.21E-7	1.6869	29.7311	4.8939	up	LevelBii	HMDB0252400	650.3271	6.6559	46.7
Misoprostol Acid	5.568E-5	0.0001995	1.6875	0.118	-3.0834	down	LevelBii	HMDB0254756	389.2282	7.6204	46.4
1-Methylene-5Alpha-Androstan-3Alpha-Ol-17-One	8.62E-5	0.0002883	1.6808	0.4513	-1.1479	down	LevelBi	-	303.2307	7.0116	49.8
Pi(Pgf2Alpha/20:0)	2.391E-8	4.659E-7	1.6794	2.8834	1.5278	up	LevelBii	HMDB0276994	997.6175	8.1832	42.1
Pectic Acid	4.982E-5	0.0001816	1.6748	0.0928	-3.429	down	LevelBii	HMDB0003363	175.0234	1.8648	47.1
N-Isobutyl-2-(2-Methyl-1H-Indol-1-Yl)Acetamide	9.903E-8	1.333E-6	1.6746	0.0393	-4.6696	down	LevelBi	-	308.174	4.1978	47.4
Oxoglutaric Acid	3.097E-10	2.712E-8	1.6754	0.1786	-2.485	down	LevelBii	HMDB0000208	205.0345	2.1593	49.9

3-O-Trans-Feruloyleuscaphic Acid	0.0007204	0.001746	1.6772	6.7482	2.7545	up	LevelBii	HMDB0031837	685.3677	2.6326	43.7
28-Glucosyloleanolic Acid 3-[Rhamnosyl-(1->2)-Galactosyl-(1->3)-Glucuronide]	2.725E-8	5.122E-7	1.6729	0.2261	-2.1452	down	LevelBii	HMDB0040859	1151.5092	5.4436	45.4
Eriojaposide B	1.928E-5	8.246E-5	1.6731	0.2528	-1.9841	down	LevelBii	HMDB0038029	1077.5079	7.3405	43.3
Smgdg(O-16:4/22:6)	4.592E-9	1.459E-7	1.6693	4.9494	2.3072	up	LevelBi	-	905.4685	4.1204	44.9
Fluorescin	3.375E-9	1.2E-7	1.6686	29.8362	4.899	up	LevelBii	HMDB0252365	393.1011	1.9876	47.6
25-Cinnamoyl-Vulgaroside	0.0006054	0.00151	1.6698	4.7744	2.2553	up	LevelBii	HMDB0041367	625.3415	8.2258	50.5
Pulchinenoside A	2.277E-9	9.344E-8	1.6661	4.0752	2.0269	up	LevelBii	HMDB0256922	771.4243	2.8309	44.2
N-(1-Deoxy-1-Fructosyl)Isoleucine	1.409E-5	6.328E-5	1.6672	0.3519	-1.5069	down	LevelBii	HMDB0039780	292.1391	1.9403	51.9
6-Hydroxy-4,4,7A-Trimethyl-5,6,7,7A-Tetrahydrobenzofuran-2(4H)-One	8.761E-10	4.895E-8	1.6651	0.3078	-1.6999	down	LevelBi	-	197.1165	3.9318	54.1
Vindoline	6.752E-6	3.432E-5	1.6621	10.394	3.3777	up	LevelBii	HMDB0259814	498.2594	2.7562	43.2
Pe(Pgf2Alpha/22:5(4Z,7Z,10Z,13Z,16Z))	1.152E-7	1.487E-6	1.6616	5.0288	2.3302	up	LevelBii	HMDB0284161	898.506	7.4244	42.1
Cdp-Dg(6 Keto-Pgf1Alpha/A-17:0)	2.742E-9	1.056E-7	1.6612	12.383	3.6303	up	LevelBii	HMDB0292976	1080.5232	3.2337	44.7
Aconitine	0.0006516	0.001604	1.6648	3.5684	1.8353	up	LevelBi	-	646.3166	2.6069	45.6
Taurohyocholic Acid	0.0003728	0.0009957	1.6634	2.9382	1.555	up	LevelBi	LMST05040010	538.2826	2.2211	43.2
Ala-Thr-Trp-Leu-Pro-Pro-Arg	4.491E-9	1.445E-7	1.6594	4.6625	2.2211	up	LevelBii	HMDB0249908	838.4663	3.9438	45.1
Asp-Gly-Leu	3.431E-10	2.915E-8	1.6584	0.2948	-1.762	down	LevelBi	-	304.1478	2.5069	46.1
Milbemycin Alpha5	8.343E-8	1.179E-6	1.6563	2.9199	1.5459	up	LevelBii	HMDB0254723	736.4017	2.6467	43.1
Abscisic Acid	3.079E-9	1.126E-7	1.6562	0.1512	-2.7256	down	LevelBi	HMDB0035140;HMDB0036093	263.1273	4.6849	45.9
Eugenosedin-A	2.075E-11	6.208E-9	1.6562	25.892	4.6944	up	LevelBii	HMDB0252125	475.2026	1.8648	39.3
Phorbol 12-Myristate 13-Acetate	0.000128	0.0004013	1.6529	6.7934	2.7641	up	LevelBii	HMDB0244744	621.3744	4.4724	46.7
Lyciumoside Vii	1.228E-6	9.455E-6	1.6536	0.4103	-1.2852	down	LevelBii	HMDB0033501	983.4666	4.8996	51.3
19-Noretiocholanolone 3-O-Glucuronide	0.000	0.001	1.6582	0.13	-2.8755	dow	Lev	HMDB0341226	453.2	6.0806	52.

	4562	181		63		n	elBii		456		3
Atropine	1.097E-8	2.624E-7	1.653	0.3567	-1.4871	down	LevelBi	HMDB0000779	322.1992	3.7153	54.7
Queuine	1.443E-9	6.859E-8	1.6509	0.215	-2.2174	down	LevelBii	HMDB0001495	278.1223	1.02	45.1
Capsianoside Iv	3.219E-5	0.0001258	1.6499	2.6616	1.4123	up	LevelBii	HMDB0030736	627.3411	3.0142	47
Managlinat Dialanetil	2.419E-6	1.468E-5	1.6453	0.2678	-1.9007	down	LevelBii	HMDB0254319	467.1862	2.5469	43.8
Gingerglycolipid C	3.001E-7	3.15E-6	1.6489	3.0518	1.6096	up	LevelBii	HMDB0041095	725.3974	8.1796	51.7
Methyl Cinnamate	2.475E-6	1.496E-5	1.6464	0.1525	-2.7127	down	LevelBi	HMDB0303905;HMDB0033833	163.0744	4.0206	51.9
Tri-N-Acetylchitotriose	1.175E-7	1.51E-6	1.6389	0.0032	-8.2966	down	LevelBii	HMDB0006698	648.2239	2.416	45.6
Lys-Ala-Arg	3.449E-8	6.018E-7	1.6367	4.7478	2.2473	up	LevelBii	HMDB0341172	408.2102	3.3296	49
Cyclohexanesulfamic Acid	5.049E-11	9.417E-9	1.6359	0.398	-1.329	down	LevelBi	HMDB0031340	180.068	2.8357	45.7
(3-Ethyl-3-Hydroxy-7-Azaspiro[3.5]Nonan-7-Yl)-Thiophen-2-Ylmethanone	3.421E-9	1.204E-7	1.6347	0.364	-1.4579	down	LevelBi	-	280.1382	1.1576	46.1
Vellein	1.429E-8	3.152E-7	1.6332	0.0356	-4.8135	down	LevelBii	HMDB0303360	413.1191	3.6936	47.9
N-3-Hydroxyoctanoyl-L-Homoserine Lactone	7.342E-6	3.674E-5	1.6293	0.0879	-3.5081	down	LevelBii	HMDB0341343	264.1231	5.1765	46.2
Cer(8:0_3O/14:1_(2Oh))	0.0006297	0.00156	1.6368	0.2094	-2.2555	down	LevelBi	-	384.3093	7.1967	48.7
Mifepristone	1.418E-5	6.357E-5	1.6327	0.2033	-2.2981	down	LevelBii	HMDB0014972	434.247	6.3883	44.8
Timosaponin B Ii	0.0002427	0.0006883	1.6319	0.4277	-1.2254	down	LevelBi	-	925.4683	7.6294	42.7
[8]-Shogaol	0.000345	0.0009325	1.6316	0.2576	-1.9566	down	LevelBii	HMDB0031463	322.2359	7.0989	53.9
Thr-Arg-Lys-Glu	0.0001144	0.0003663	1.6232	0.0322	-4.9576	down	LevelBi	-	531.2957	5.9789	43.9
N-[4-(Aminosulfonyl)Phenyl]Propanamide	1.283E-9	6.266E-8	1.6207	0.305	-1.7133	down	LevelBi	-	229.063	2.9249	51.9
Agnuside	5.742E-10	3.748E-8	1.6187	0.211	-2.245	down	LevelBii	HMDB0036561	431.1355	4.5992	44.4
2-Hydroxy-4-(4-Methoxyphenyl)-6-Phenylnicotinonitrile	6.249E-5	0.0002203	1.6202	0.3862	-1.3728	down	LevelBi	-	320.1424	2.6865	43.6
Glycochenodeoxycholic Acid 3-Glucuronide	6.154E-9	1.764E-7	1.6162	2.8593	1.5157	up	LevelBii	HMDB0002579	624.3341	2.6988	45.7
5-(3-Pyridyl)-2-Hydroxytetrahydrofuran	2.872	5.301	1.6166	0.19	-2.3877	dow	Lev	HMDB0062407	210.0	3.0167	47.

	E-8	E-7		11		n	elBii		761		4
Meta-O-Dealkylated Flecainide Lactam	2.094E-7	2.393E-6	1.6147	0.2034	-2.2975	down	LevelBii	HMDB0060832	391.11	3.1301	47.3
1,2,3,4-Tetrahydroisoquinoline-3-Carboxylic Acid	3.126E-7	3.255E-6	1.6147	0.4249	-1.2349	down	LevelBii	HMDB0244117	236.0917	3.7311	48.2
Adenosyl Cobyrinic Acid A,C Diamide	3.097E-6	1.787E-5	1.6089	0.2118	-2.2395	down	LevelBii	HMDB0001083	1167.4386	7.1364	44.2
Acetyldiphenylalanyl-Leucyl-Aspartyl-Isoleucyl-Isoleucyl-Tryptophan	2.139E-5	9.012E-5	1.6078	0.2625	-1.9298	down	LevelBii	HMDB0256173	968.4728	5.9973	48.5
N-Eicosapentaenoyl Cysteine	0.0001128	0.0003617	1.6059	4.06	2.0215	up	LevelBii	HMDB0242068	450.233	6.4803	45.2
Mg(5-Iso Pgf2Vi/0:0/0:0)	4.441E-8	7.255E-7	1.6025	0.1618	-2.6277	down	LevelBii	HMDB0260485	421.2186	5.4251	47.6
Halosulfuron-Methyl	2.417E-12	2.384E-9	1.601	0.2316	-2.1102	down	LevelBii	HMDB0034859	493.0592	2.5098	42.9
(2R)-2-[[(2R)-2,6-Diaminohexanoyl]Amino]-3-Phenylpropanoic Acid	6.5E-11	1.085E-8	1.6003	9.4879	3.2461	up	LevelBi	-	316.1608	1.6103	42.8
Npc	4.31E-5	0.0001606	1.5993	0.1199	-3.0602	down	LevelBii	HMDB0060499	1081.4294	5.2134	47.5
Tyr-Gly-Gly-Trp-Leu	2.607E-8	4.964E-7	1.6	0.0285	-5.1337	down	LevelBii	HMDB0259342	615.2601	3.48	48.4
Retinol	6.521E-7	5.732E-6	1.5963	0.1743	-2.5206	down	LevelBi	LMPR01090001	269.225	4.6482	46.9
Pip(6 Keto-Pgf1Alpha/18:2(9Z,12Z))	1.744E-9	7.732E-8	1.5959	5.2878	2.4027	up	LevelBii	HMDB0279170	1049.4639	3.3763	50.1
1-Cyclohexyl-3-(1-Propylsulfonyl-3,4-Dihydro-2H-Quinolin-7-Yl)Urea	6.467E-7	5.705E-6	1.5929	6.2148	2.6357	up	LevelBi	-	380.2025	5.1562	51
(8Z,10E)-Dodeca-8,10-Dienoylcarnitine	5.504E-8	8.517E-7	1.5939	7.1751	2.843	up	LevelBii	HMDB0241245	374.2099	4.0742	46.7
Trp-Val-Ala	0.0002894	0.000803	1.5942	4.6713	2.2238	up	LevelBi	-	375.2035	2.7362	45.6
Prednisolone Valerate Acetate	2.551E-9	1.007E-7	1.5898	4.5944	2.1999	up	LevelBii	HMDB0256750	451.2512	3.4686	41.3
Pc(Pgf2Alpha/16:0)	0.00102	0.002353	1.5934	3.755	1.9088	up	LevelBii	HMDB0285926	812.5441	7.3964	45.5
Tamsulosin	1.16E-8	2.722E-7	1.5875	0.3832	-1.384	down	LevelBi	HMDB0014844;HMDB0259951	409.1811	3.133	52.3
Vignatic Acid B	0.0001827	0.0005388	1.5875	2.2596	1.1761	up	LevelBii	HMDB0033617	574.3078	3.133	44.4
(9Z,11E)-Octadeca-9,11-Dienoylcarnitine	3.526E-5	0.0001361	1.5902	0.2226	-2.1675	down	LevelBii	HMDB0241539	468.3306	6.85	46.5
1-Deoxy-1-Morpholino-D-Fructose	4.267E-8	7.095E-7	1.5862	0	-15.8606	down	LevelBii	HMDB0243858	270.0939	3.4894	46.3
Gibberellin A28	6.501	1.825	1.5829	0.04	-4.4518	dow	Lev	HMDB0303573	375.1	3.48	45.

	E-9	E-7		57		n	elBii		415		8
Quadranoside Iii	0.001187	0.002685	1.5844	2.5124	1.3291	up	LevelBi	-	705.3549	3.5968	46.5
Phytosphingosine	0.00125	0.002806	1.5816	2.2096	1.1438	up	LevelBi	HMDB0004610;HMDB0004628;HMDB0004610;LMSP01030001	318.2988	6.7485	52.7
(2'E,4'Z,8E)-Colneleic Acid	0.0001546	0.0004706	1.5817	0.1921	-2.3799	down	LevelBii	HMDB0030995	329.1868	7.415	50.2
Ulimorelin	4.701E-7	4.482E-6	1.5807	3.4552	1.7888	up	LevelBii	HMDB0259396	521.2889	3.656	47.6
Angiotensin Iii	0.0002864	0.0007952	1.5826	3.516	1.8139	up	LevelBii	HMDB0001036	989.5287	4.4538	46.9
Apc	5.129E-6	2.732E-5	1.5801	0.2242	-2.157	down	LevelBii	HMDB0060661	677.2796	4.2873	45.8
1-Phenyl-2,4-Pentadiyn-1-One	3.599E-8	6.189E-7	1.5777	5.9032	2.5615	up	LevelBii	HMDB0032945	353.0794	2.9979	44.5
Pravastatin	2.261E-6	1.387E-5	1.5784	0.1991	-2.3281	down	LevelBii	HMDB0005022	483.2561	5.9789	44.6
Kaempferol 3-O-Alpha-L-Rhamnofuranoside	2.549E-5	0.0001045	1.5777	0.1684	-2.5701	down	LevelBii	HMDB0040542	431.0953	4.5003	50.2
1-Linoleoyl Glycerol	0.01874	0.03011	1.5973	2.3695	1.2446	up	LevelBi	-	355.2829	8.3582	48.7
2-{1-[2-(1-Benzothiophen-5-Ylamino)-2-Oxoethyl]Cyclohexyl}Acetic Acid	3.194E-11	7.694E-9	1.5751	20.6283	4.3666	up	LevelBi	-	332.1332	1.8237	48.9
Prolinamide,D-Tyrosyl-L-Arginylglycyl-4-Nitro-L-Phenylalanyl-	2.153E-7	2.44E-6	1.5747	0.4115	-1.281	down	LevelBii	HMDB0247954	727.3134	5.3238	49.3
Desmethyldihydrocapsaicin	9.145E-5	0.000303	1.5754	0.1099	-3.186	down	LevelBi	-	292.19	6.3508	48.7
Strobilurin	1.201E-5	5.539E-5	1.5687	0.1052	-3.2488	down	LevelBii	HMDB0258515	441.1953	3.9344	49.4
2',4',6'-Trihydroxydihydrochalcone 2'-Glucoside	1.029E-6	8.245E-6	1.5698	0.1365	-2.8732	down	LevelBii	HMDB0037483	441.1133	4.2596	47.4
Kaempferol-7-O-Glucoside	1.002E-5	4.755E-5	1.5691	0.3775	-1.4054	down	LevelBi	-	447.0909	4.1669	45.4
Pip(Pgf2Alpha/16:0)	1.563E-5	6.892E-5	1.5697	0.1792	-2.4803	down	LevelBii	HMDB0278446	1023.4594	5.5172	50.8
Glycerophosphocholine	1.043E-7	1.387E-6	1.5669	1.8301	0.8719	up	LevelBi	HMDB0000086;PW_C000059	258.1092	0.6595	55.4
Epothilone A	0.0001623	0.000489	1.5688	2.3714	1.2457	up	LevelBii	HMDB0251873	458.2408	3.4588	42
Lpg(20:5)	2.492E-9	9.913E-8	1.5674	0.2014	-2.3121	down	LevelBi	LMGP04050030	575.2677	3.48	43.2
Serofendic Acid	2.409E-7	2.671E-6	1.5649	0.1836	-2.4453	down	LevelBii	HMDB0258253	417.1866	5.8587	45.5
N-Lactoyl-Tryptophan	2.688	1.591	1.563	0.31	-1.679	dow	Lev	HMDB0062178	297.0	2.979	47.

	E-6	E-5		23		n	elBii		842		9
N-Isobutyloctadeca-Trans-2-Trans-4-Dienamide	1.956 E-7	2.271 E-6	1.5648	0.33 86	-1.5622	dow n	Lev elBii	HMDB0302880	380.3 154	8.8985	50
Fahfa(18:1/6:0)	0.000 1666	0.000 4999	1.5619	0.34 16	-1.5496	dow n	Lev elBi	-	395.3 141	9.4389	55. 1
Glucosyl (2E,6E,10X)-10,11-Dihydroxy-2,6-Farnesadien oate	0.000 3332	0.000 9052	1.5675	2.44 17	1.2879	up	Lev elBii	HMDB0037823	437.2 159	2.4872	46. 1
Sipoglitazar	7.337 E-6	3.673 E-5	1.5641	0.07 15	-3.8058	dow n	Lev elBii	HMDB0258310	444.1 378	4.4817	45. 3
9-Octadecynoic Acid	0.000 3419	0.000 9253	1.5674	1.84 94	0.8871	up	Lev elBi	LMFA01030455	281.2 466	7.1087	53. 4
Streptozocin	9.496 E-8	1.288 E-6	1.563	0.16 94	-2.5619	dow n	Lev elBii	HMDB0014572	264.0 855	3.675	48. 7
Capsianoside Iii	3.207 E-6	1.839 E-5	1.5596	0.53 48	-0.903	dow n	Lev elBii	HMDB0031891	1145. 521	4.657	52
7-[(3-Methoxy-4-Propan-2-Yloxyphenyl)Methy l]-7-Azaspiro[3.5]Nonan-3-Ol	0.000 4126	0.001 083	1.5629	0.30 73	-1.7025	dow n	Lev elBi	-	320.2 202	6.7681	50. 6
3'-Amp	3.523 E-7	3.569 E-6	1.5582	0.33 41	-1.5816	dow n	Lev elBii	HMDB0003540	346.0 539	1.9214	52. 3
Yessotoxin	8.959 E-9	2.298 E-7	1.5562	0.38 13	-1.391	dow n	Lev elBii	HMDB0033320	1187. 4818	5.4527	46
Pi(O-10:0/14:1)	2.567 E-9	1.007 E-7	1.5556	2.42 42	1.2775	up	Lev elBi	-	727.4 107	8.5663	47. 5
Ps(5-Iso Pgf2Vi/18:3(6Z,9Z,12Z))	5.488 E-10	3.649 E-8	1.5554	3.18 83	1.6728	up	Lev elBii	HMDB0281779	866.4 287	3.912	40. 9
Spinorphin	0.000 6398	0.001 581	1.5597	0.16 27	-2.62	dow n	Lev elBii	HMDB0059790	875.4 637	6.7023	44. 6
Dodecyl-Beta-D-Maltoside	3.634 E-5	0.000 1394	1.5547	0.33 13	-1.5938	dow n	Lev elBii	HMDB0247203	491.2 835	5.2964	48. 2
2',6'-Dihydroxy-4'-Methoxyacetophenone	5.437 E-11	1.001 E-8	1.553	6.02 39	2.5907	up	Lev elBii	HMDB0029646	181.0 497	4.8811	54. 1
Cyclosquamosin D	7.659 E-6	3.801 E-5	1.5474	0.26 72	-1.9039	dow n	Lev elBii	HMDB0303369	835.3 926	5.5541	44. 5
Mlcl(14:0/14:1/18:3)	0.000 613	0.001 524	1.5493	0.35 56	-1.4917	dow n	Lev elBi	-	1123. 6365	7.5551	45
7Beta-Hydroxy-7-Desacetoxykhivorinic Acid, Methyl Ester	0.000 1297	0.000 4059	1.544	0.12 5	-2.9997	dow n	Lev elBi	-	571.2 377	4.8627	48
N-Acetylhistamine	5.305 E-10	3.56E -8	1.5453	0.31 48	-1.6674	dow n	Lev elBii	HMDB0013253	154.0 966	1.0886	50. 3
Ps(Pgf2Alpha/14:1(9Z))	1.314 E-8	2.972 E-7	1.5433	3.73 01	1.8992	up	Lev elBii	HMDB0280933	784.4 384	2.7933	43. 7
Lpc(12:0)	7.244 E-9	1.982 E-7	1.5431	4.24 77	2.0867	up	Lev elBi	LMGP01050009	484.2 66	6.7394	48. 9

Dibenzepin	4.552E-5	0.0001683	1.5422	0.1989	-2.3298	down	LevelBii	HMDB0251161	635.3319	7.3405	46.1
Ps(5-Iso Pgf2Vi/14:0)	8.339E-12	4.0E-9	1.5421	4.2044	2.0719	up	LevelBii	HMDB0280844	760.434	4.4724	39.7
Protoveratrine	8.478E-9	2.206E-7	1.5416	4.485	2.1651	up	LevelBii	HMDB0256876	838.4291	3.703	44
Pg(Pgf1Alpha/16:0)	0.001764	0.003785	1.546	1.7826	0.834	up	LevelBii	HMDB0268652	840.5676	7.7639	50.4
1-Methylene-5Alpha-Androstan-3Alpha-Ol-17-One Glucuronide	7.481E-7	6.391E-6	1.5415	0.3482	-1.5221	down	LevelBii	HMDB0341213	479.2605	5.2342	45.9
Cis-3-Methyl Thiofentanyl	2.206E-5	9.247E-5	1.5401	0.2258	-2.1467	down	LevelBi	-	357.2009	5.7522	42.9
Leucic Acid	4.317E-8	7.13E-7	1.5359	0.0564	-4.1489	down	LevelBii	HMDB0000624	113.0599	5.3422	53.2
Palmitoyl Glucuronide	0.01286	0.02147	1.5511	2.216	1.1479	up	LevelBii	HMDB0010331	399.2736	8.3544	53
N-Feruloylglycyl-L-Phenylalanine	0.0001307	0.0004088	1.5291	0.3051	-1.7128	down	LevelBii	HMDB0029356	397.1437	2.5853	45
N-Phenethylacetamide	4.258E-7	4.148E-6	1.5312	0.2952	-1.7601	down	LevelBi	HMDB0255222	164.1061	4.1783	54.6
7-Hydroxybutylidenephthalide 7-(6-Malonylglucoside)	0.0002087	0.0006045	1.5347	0.1795	-2.4783	down	LevelBii	HMDB0034753	497.13	4.7428	50
Val-Phe-Val-Tyr	5.928E-6	3.075E-5	1.5324	0.4218	-1.2454	down	LevelBii	HMDB0304812	571.2819	2.0535	45.6
Amphibine H	0.0005426	0.001374	1.53	2.3994	1.2627	up	LevelBii	HMDB0029333	650.3139	2.0535	51.2
Pg(O-12:0/22:4)	4.536E-5	0.000168	1.5308	0.2775	-1.8494	down	LevelBi	-	727.4903	8.9731	41
N-(3,5-Dimethylphenyl)-2-(5-Pyrazin-2-Yl-1,3,4-Oxadiazol-2-Yl)Acetamide	4.275E-8	7.098E-7	1.5324	0.5026	-0.9926	down	LevelBi	-	310.1281	2.1616	46.6
Cyclovirobuxine D	0.001251	0.002808	1.5337	0.2253	-2.1502	down	LevelBi	HMDB0250688	407.3388	7.0892	49.2
1,2,3,4-Tetrahydro-6-Propanoylpyridine	2.331E-8	4.573E-7	1.5285	0.1837	-2.4447	down	LevelBii	HMDB0034884	198.1124	4.38	55.8
10-Formyldihydrofolate	7.875E-7	6.668E-6	1.5241	0.3159	-1.6624	down	LevelBii	HMDB0006485	470.1423	2.5853	49.4
Ala-Gly-Tyr	0.001129	0.002567	1.5228	2.3119	1.2091	up	LevelBi	-	310.138	1.8336	48.9
Tyr-Hyp	5.913E-9	1.709E-7	1.5179	0.1022	-3.29	down	LevelBii	HMDB0029106	339.1181	2.2258	46.8
3-O-Acetylepisamarcandin	1.96E-6	1.229E-5	1.5174	2.1206	1.0845	up	LevelBi	HMDB0031958	497.2532	2.9348	45.7
Arg-Glu-Leu	3.013E-5	0.0001191	1.5184	3.4307	1.7785	up	LevelBi	-	417.2473	4.1586	46

Cdp-Dg(Lte4/I-14:0)	1.605E-5	7.067E-5	1.517	0.3646	-1.4557	down	LevelBii	HMDB0293501	1167.4993	5.6368	47.3
20,26-Dihydroxyecdysone	0.003102	0.006191	1.5224	2.3725	1.2464	up	LevelBii	HMDB0245570	555.3161	3.2619	53.2
Avenacin B2	1.787E-10	2.021E-8	1.5151	4.5702	2.1923	up	LevelBii	HMDB0303400	1093.5297	2.8403	39.5
Citrusin I	9.116E-5	0.0003021	1.5157	3.8983	1.9629	up	LevelBii	HMDB0303680	762.4023	3.8692	45.2
2-[4-(4-Tert-Butylphenyl)Sulfonylpiperazin-1-Yl]-1-Pyrrolidin-1-Ylpropan-1-One	5.492E-9	1.633E-7	1.5139	5.6982	2.5105	up	LevelBi	-	430.2152	5.8787	45.4
Dg(Pge1/A-17:0/0:0)	6.145E-7	5.478E-6	1.5128	4.0425	2.0152	up	LevelBii	HMDB0297871	679.5167	9.5596	47.3
17-Aminogeldanamycin	0.000233	0.0006653	1.5135	2.5239	1.3357	up	LevelBii	HMDB0259661	563.3062	3.3012	49.3
Alpha-Zearalenol	1.104E-6	8.709E-6	1.5113	0.3425	-1.5458	down	LevelBii	HMDB0041824	362.1941	3.7845	49.7
Atorvastatin	2.344E-6	1.428E-5	1.511	0.141	-2.8266	down	LevelBii	HMDB0005006	539.232	4.1298	45.3
Salidroside	1.501E-5	6.659E-5	1.5088	0.1148	-3.1232	down	LevelBii	HMDB0257463	359.1342	3.7218	50.1
Gln-Thr	1.251E-6	9.579E-6	1.5115	4.3093	2.1075	up	LevelBii	HMDB0028807	493.2281	2.7839	46.6
(4E,15Z)-Bilirubin	1.172E-5	5.428E-5	1.5091	8.8908	3.1523	up	LevelBii	HMDB0000488	605.2421	8.5195	44.2
Ile-Hyp	4.06E-12	2.915E-9	1.507	0.4533	-1.1416	down	LevelBii	HMDB0028908	245.1486	2.1112	46.5
Uridine	2.883E-7	3.067E-6	1.5062	2.5291	1.3386	up	LevelBi	HMDB0000296;PW_C000202	243.0603	1.1746	43.9
Pe(O-8:0/8:0)	7.129E-7	6.136E-6	1.506	1.9327	0.9506	up	LevelBi	-	905.5692	8.1614	50.4
Ala-Gly-Leu-Val-Ser	4.709E-5	0.0001731	1.5058	4.0766	2.0274	up	LevelBi	-	444.2446	3.273	46.5
Carpinontriol B	4.713E-7	4.488E-6	1.5035	0.4117	-1.2804	down	LevelBi	-	345.1304	3.6363	42.6
Kudzusaponin Sa2	3.677E-5	0.0001409	1.5052	0.3908	-1.3554	down	LevelBii	HMDB0041241	979.472	5.2227	51.5
Soybean Saponin Bg	0.0001691	0.0005064	1.5057	0.3395	-1.5583	down	LevelBii	HMDB0302658	945.4665	7.3871	50.8
Quercetin 3-O-Glucoside	4.37E-7	4.243E-6	1.5047	0.0274	-5.1895	down	LevelBi	HMDB0037362	463.0876	3.8321	49.3
Fahfa(16:0/6:0)	2.622E-5	0.000107	1.4981	0.3291	-1.6035	down	LevelBi	-	369.2989	9.4389	52.8
Pro-Ile	8.884E-10	4.936E-8	1.5009	0.4312	-1.2136	down	LevelBi	HMDB0304810	211.143	2.1912	44.2

2-Amino-1-Phenylethanol	0.000 1487	0.000 4555	1.502	2.56 39	1.3583	up	Lev elBi	HMDB0001065	120.0 797	1.6103	51. 3
Formyl-5-Hydroxykynurenamine	0.000 3372	0.000 9143	1.4999	4.56 93	2.192	up	Lev elBii	HMDB0012948	267.0 971	1.5948	49. 2
Aconitic Acid	7.311 E-8	1.075 E-6	1.4975	0.21 39	-2.2253	dow n	Lev elBi	HMDB0247961	173.0 079	1.9403	50. 8
19-Oxoandrost-4-Ene-3,17-Dione	0.001 551	0.003 386	1.5001	0.23 49	-2.09	dow n	Lev elBii	HMDB0006768	645.3 448	6.5175	48. 7
Kentsin	0.000 1963	0.000 574	1.4979	5.37 51	2.4263	up	Lev elBii	HMDB0005776	564.3 258	6.2803	44. 9
Huperzine A	6.243 E-5	0.000 2202	1.4927	2.33 41	1.2229	up	Lev elBii	HMDB0242212	243.1 51	2.0017	45
Amp-Deoxynojirimycin	3.165 E-5	0.000 124	1.4922	3.77 6	1.9169	up	Lev elBii	HMDB0248353	420.2 691	2.5269	42. 3
Gambogic Acid	5.459 E-5	0.000 1963	1.4941	0.55 58	-0.8473	dow n	Lev elBii	HMDB0034095	687.3 194	6.0435	48
Asparaginylglycyl-L-Arginine	8.836 E-7	7.306 E-6	1.4938	0.46 57	-1.1027	dow n	Lev elBii	HMDB0253872	326.1 57	3.5833	50. 2
Acetyl-Dl-Valine	1.181 E-6	9.161 E-6	1.4915	0.33 46	-1.5794	dow n	Lev elBi	-	158.0 812	2.6136	51. 9
1-Ethyl-2-Pyrrolidinone	2.045 E-8	4.107 E-7	1.4934	0.39 12	-1.3539	dow n	Lev elBi	HMDB0243878	114.0 909	2.6567	49. 9
Cucurbitacin D	0.000 8672	0.002 046	1.4946	2.72 85	1.4481	up	Lev elBii	HMDB0034695	516.3 104	2.0614	47. 1
3-O-Methylkaempferol	0.000 364	0.000 9761	1.4936	0.11 43	-3.1291	dow n	Lev elBii	HMDB0302564	299.0 545	5.1673	51. 5
Etimicin	0.001 048	0.002 405	1.4916	3.54 94	1.8276	up	Lev elBii	HMDB0252101	458.2 964	2.7933	49. 1
Ethyl Acrylate	5.026 E-9	1.546 E-7	1.4864	0.37 25	-1.4248	dow n	Lev elBi	HMDB0033978	101.0 593	2.2706	53. 2
Cyclolinopeptide B	0.007 048	0.012 67	1.4928	2.60 59	1.3818	up	Lev elBii	HMDB0302161	1078. 587	3.2619	42. 7
D[-Arg-2]Kyotorphan	0.000 7168	0.001 74	1.4903	2.97 78	1.5743	up	Lev elBi	-	336.1 666	1.2213	48. 7
4-O-Beta-D-Glucosyl-4-Coumaric Acid	5.574 E-8	8.589 E-7	1.4823	0.02 87	-5.1242	dow n	Lev elBi	HMDB0039509	344.1 329	2.7562	55. 7
2-{[2-(1H-Indol-3-Yl)Ethyl]Ethanimidoyl}Phen ol	5.333 E-5	0.000 1925	1.4752	14.1 137	3.819	up	Lev elBi	-	333.1 545	2.4099	45. 1
Paliperidone	4.663 E-8	7.546 E-7	1.4768	0.26 36	-1.9235	dow n	Lev elBii	HMDB0015396	447.1 824	2.0631	50. 9
Xi-7-Hydroxyhexadecanedioic Acid	1.993 E-5	8.49E -5	1.4761	0.41 81	-1.258	dow n	Lev elBii	HMDB0037830	301.2 006	5.7845	46. 9
Tetrahydroharman-3-Carboxylic Acid	1.092 E-7	1.431 E-6	1.4754	0.55 92	-0.8384	dow n	Lev elBi	-	231.1 123	2.9844	55. 7

Rosamicin	0.004309	0.008237	1.4877	2.4131	1.2709	up	LevelBii	HMDB0257306	580.3443	2.576	52.5
Mikamycin B	9.631E-7	7.811E-6	1.4757	0.5056	-0.9839	down	LevelBii	HMDB0256777	901.374	3.6111	42.5
Trp-Ser	4.034E-7	3.976E-6	1.4743	0.186	-2.4269	down	LevelBii	HMDB0029092	336.1183	2.6326	50.1
Peridinin	0.0003543	0.0009535	1.4792	2.2631	1.1783	up	LevelBii	HMDB0256341	635.3364	2.517	48.7
Arg-His	2.305E-7	2.575E-6	1.4728	0.4736	-1.0782	down	LevelBii	HMDB0028711	356.1686	3.5552	53.9
2-[[2-(1H-Indol-3-Yl)Acetyl]Amino]Glutaric Acid	0.0009564	0.002226	1.4728	2.1536	1.1067	up	LevelBi	-	305.1148	3.6068	45.9
Methyl 3-Hydroxy-2-[(3-Hydroxybenzoyl)Amino]Propanoate	2.921E-5	0.0001163	1.4676	2.953	1.5622	up	LevelBi	-	257.1119	2.0414	50.7
2-Phenylethyl Acetate	2.984E-9	1.102E-7	1.4682	0.1467	-2.7694	down	LevelBii	HMDB0033945	209.0808	3.6564	52
Trans-O-Coumaric Acid 2-Glucoside	2.289E-7	2.563E-6	1.4676	0.2285	-2.1298	down	LevelBi	HMDB0033581	325.0917	2.7555	55.9
Gly-Leu-Lys	6.324E-7	5.61E-6	1.4651	3.7058	1.8898	up	LevelBi	-	317.2171	1.6589	44.8
Avenacoside A	0.0001457	0.0004473	1.468	0.4181	-1.2581	down	LevelBii	HMDB0033815	1107.5183	7.2847	49.4
Dodec-2-Enedioic Acid	6.409E-6	3.286E-5	1.4633	0.3328	-1.5873	down	LevelBii	HMDB0340663	246.1693	5.5564	46.4
Multinoside A	2.241E-6	1.378E-5	1.4652	0.2755	-1.8598	down	LevelBii	HMDB0037935	609.1427	3.9998	51.6
Harman	1.046E-10	1.415E-8	1.4656	0.4528	-1.143	down	LevelBi	HMDB0035196	183.0905	3.0437	52.6
Alpha-Hydroxymetoprolol	0.0002603	0.0007317	1.4708	0.3921	-1.3508	down	LevelBii	HMDB0060994	284.1846	3.123	49.3
5-Methoxysalicylic Acid	1.59E-9	7.354E-8	1.4649	0.3051	-1.7125	down	LevelBi	HMDB0001868	167.0337	3.1018	48.4
Temsirolimus	0.0004032	0.001062	1.4659	2.6421	1.4017	up	LevelBii	HMDB0015632	1047.6327	7.7929	46.2
Arg-Met-His	2.269E-9	9.344E-8	1.4642	0.3803	-1.3948	down	LevelBi	-	460.2478	1.1086	42.3
19-Noretiocholanolone Glucuronide	1.601E-7	1.939E-6	1.4565	3.0222	1.5956	up	LevelBi	-	485.2731	3.8331	43.1
8-Aminooctanoic Acid	0.0001135	0.0003637	1.4561	0.4308	-1.2148	down	LevelBii	HMDB0247418	160.1324	2.6965	53.1
2-Deoxy-2,3-Dehydro-N-Acetyl-Neuraminic Acid	9.757E-7	7.894E-6	1.455	0.5013	-0.9961	down	LevelBi	HMDB0245680	314.0825	0.7467	48.9
N-Docosahexaenoyl Valine	0.000	0.000	1.454	0.48	-1.0389	dow	Lev	HMDB0242025	460.3	5.2049	48.

	1197	3801		67		n	elBii		389		5
Gonane	1.514E-7	1.853E-6	1.451	2.7013	1.4336	up	LevelBii	HMDB0252914	233.2259	7.2066	44.8
Valtrate	2.927E-5	0.0001165	1.4472	0.2858	-1.807	down	LevelBii	HMDB0034493	233.0416	3.4613	50
Gly-Ser-Pro-Met-Phe	4.759E-8	7.622E-7	1.4465	2.6181	1.3885	up	LevelBii	HMDB0033242	616.2923	3.273	46.2
Secoeremopetasitolide B	1.154E-7	1.488E-6	1.4464	1.9231	0.9434	up	LevelBii	HMDB0041363	427.2291	3.123	43.1
Ps(6 Keto-Pgf1Alpha/16:1(9Z))	8.151E-9	2.148E-7	1.4458	2.6636	1.4134	up	LevelBii	HMDB0281243	882.456	4.1204	47.6
Dlcl(14:0/16:4)	0.002051	0.004318	1.4507	0.3807	-1.3933	down	LevelBi	-	885.4197	5.9512	48.3
Mangostanol	3.866E-5	0.0001472	1.4417	0.3018	-1.7283	down	LevelBii	HMDB0029868	461.1379	2.576	47.3
Canavalioside	2.013E-5	8.559E-5	1.4434	3.6345	1.8618	up	LevelBii	HMDB0036317	545.2574	3.9717	49.4
Prostaglandin H2 2-Glyceryl Ester	1.057E-7	1.398E-6	1.444	2.3515	1.2336	up	LevelBii	HMDB0062663	426.259	7.3937	45.3
Wikstromol	1.878E-6	1.191E-5	1.4429	0.2047	-2.2882	down	LevelBii	HMDB0030598	355.116	4.2596	47.2
Tyr-Ile-Leu	0.00721	0.0129	1.4496	3.4204	1.7741	up	LevelBi	HMDB0013024	408.2464	3.6165	50
11'-Carboxy-Gamma-Chromanol	1.135E-6	8.899E-6	1.4414	0.2547	-1.9731	down	LevelBii	HMDB0012517	403.2816	9.2981	46.9
Pa(5-Iso Pgf2Vi/10:0)	1.909E-5	8.174E-5	1.4443	3.4945	1.8051	up	LevelBii	HMDB0262688	679.3437	4.4169	48.1
Ile-Glu-Ile	0.0001102	0.0003545	1.4438	2.9826	1.5766	up	LevelBi	-	396.2085	4.3253	45.3
15-Epi Prostaglandin A1	5.197E-5	0.0001885	1.442	0.4586	-1.1247	down	LevelBi	-	337.2354	5.8592	49.5
4-Hydroxyphenyl-2-Propionic Acid	1.293E-7	1.633E-6	1.4409	0.067	-3.8995	down	LevelBii	HMDB0041683	165.0544	5.0198	45.8
3-(Benzoyloxy)-2-Hydroxypropyl ?-D-Glucopyranosiduronic Acid	1.544E-9	7.197E-8	1.4407	2.7195	1.4433	up	LevelBi	-	371.0969	3.3483	48.4
Heptanohydrazide	1.719E-5	7.491E-5	1.441	1.9875	0.9909	up	LevelBi	-	199.143	3.133	45.2
Kaempferol 3-[Apiosyl-(1->2)-Galactoside]	0.0002355	0.0006713	1.4408	0.1042	-3.2625	down	LevelBii	HMDB0037430	579.1333	3.6657	47.9
(4-Formyl-9-Methoxy-5-Methylnaphtho[2,3-B]Furan-3-Yl)Methyl Acetate	8.78E-6	4.283E-5	1.4384	0.4034	-1.3097	down	LevelBi	-	345.1303	4.2569	45.8
Leukotriene F4	1.268E-6	9.663E-6	1.437	1.7768	0.8293	up	LevelBii	HMDB0006465	586.311	3.4686	42.8
Lisinopril	8.907	1.236	1.4346	6.03	2.5944	up	Lev	HMDB0001938	440.1	4.3244	48.

	E-8	E-6		93			elBii		994		8
Erythro-L-Galacto-Nonulose	2.667E-7	2.884E-6	1.4343	0.4166	-1.2633	down	LevelBii	HMDB0029955	275.0723	0.776	47.1
Gamithromycin	0.0003331	0.000905	1.4363	2.4079	1.2678	up	LevelBii	HMDB0252606	797.5135	7.8296	49.6
3-Ureidopropionic Acid	1.047E-8	2.554E-7	1.4338	0.3484	-1.5212	down	LevelBi	HMDB0000026	115.0495	1.1285	52
N-Oleoyl Glutamic Acid	3.665E-5	0.0001404	1.4354	2.0098	1.007	up	LevelBii	HMDB0241959	392.279	8.198	51.8
Adipate Semialdehyde	6.801E-9	1.892E-7	1.433	0.357	-1.4861	down	LevelBii	HMDB0012882	151.0389	2.9979	53.6
Pip(Pgf2Alpha/22:4(10Z,13Z,16Z,19Z))	0.0002161	0.0006223	1.4351	0.2292	-2.1251	down	LevelBii	HMDB0280418	1085.4952	4.1484	44.5
Phe-His	0.0001155	0.0003691	1.4307	2.092	1.0649	up	LevelBi	HMDB0028892	303.1433	1.6688	48
Americine	0.006947	0.01251	1.4353	2.1093	1.0767	up	LevelBii	HMDB0034441	528.299	2.4582	37.5
(3S,7E,9R)-4,7-Megastigmadiene-3,9-Diol 9-[Apiosyl-(1->6)-Glucoside]	8.218E-8	1.168E-6	1.4296	2.2084	1.143	up	LevelBii	HMDB0029766	527.2498	4.0009	44.2
Cdp-Dg(Pge2/A-21:0)	0.002961	0.005942	1.4353	2.6745	1.4192	up	LevelBii	HMDB0293076	1100.5692	3.2619	45.2
Ile-Gln-Phe	0.00226	0.004697	1.432	2.6784	1.4214	up	LevelBi	-	407.2267	3.4783	44.9
Ile-Thr-Trp	0.000213	0.0006147	1.4299	2.1136	1.0797	up	LevelBi	-	451.2517	2.3402	47
Lys-Asp-Tyr	0.0001905	0.0005586	1.4277	0.2084	-2.2626	down	LevelBii	HMDB0304806	459.1635	6.1478	54.5
Ganosporeric Acid A	3.695E-5	0.0001415	1.4257	3.2475	1.6993	up	LevelBii	HMDB0033022	527.2611	3.2715	48.5
Maltitol	0.0004963	0.001271	1.4301	2.9853	1.5779	up	LevelBi	HMDB0002928	343.128	2.207	49.4
Pyroglutamyllysine	4.233E-5	0.0001583	1.4288	0.3013	-1.7308	down	LevelBii	HMDB0341383	302.1348	2.5098	54
6-Guanidino-2-Oxocaproic Acid	3.848E-8	6.501E-7	1.4255	5.232	2.3874	up	LevelBii	HMDB0247067	373.1872	2.7365	48.5
Thymopentin	4.56E-7	4.377E-6	1.4211	2.5215	1.3343	up	LevelBii	HMDB0259057	644.3574	1.9124	46.1
Cys-Trp-Ala	5.136E-8	8.068E-7	1.4215	0.4607	-1.1181	down	LevelBii	HMDB0341149	437.1536	2.9509	50.1
3-Methylcyclopentene	0.0007408	0.001789	1.4148	0.4269	-1.228	down	LevelBii	HMDB0031544	83.0857	2.8357	47.5
N-Cyclooctyl-1-(4-Fluorobenzoyl)Piperidine-4-Carboxamide	7.06E-6	3.556E-5	1.4198	0.4642	-1.1071	down	LevelBi	-	361.2309	5.5077	43.2
Pc(Pge1/P-16:0)	0.000	0.001	1.4226	3.59	1.844	up	Lev	HMDB0289518	814.5	7.8112	41

	5392	367		01			elBii		626		
Testosterone Glucuronide	1.361E-5	6.149E-5	1.4179	1.8518	0.889	up	LevelBii	HMDB0003193	465.2439	2.9447	43.7
Pa(2:0/16:4)	0.0001962	0.000574	1.4189	4.9747	2.3146	up	LevelBi	-	489.1936	3.0261	46.3
Gingerglycolipid A	1.136E-7	1.472E-6	1.4169	0.4502	-1.1513	down	LevelBii	HMDB0041093	675.3567	5.4988	49.9
(9S,10S)-9,10-Dihydroxyoctadecanoate	0.000239	0.0006796	1.4189	1.4445	0.5306	up	LevelBii	HMDB0059633	315.2529	7.1091	47.3
Calenduloside H Methyl Ester	0.0009323	0.002178	1.4191	0.2519	-1.9891	down	LevelBii	HMDB0041021	1005.4864	7.2568	45.5
Pgp(Txb2/I-18:0)	0.0001487	0.0004556	1.4164	0.357	-1.4861	down	LevelBii	HMDB0275447	979.4694	5.6646	49.7
Met-Gly	1.101E-6	8.696E-6	1.4096	3.7991	1.9256	up	LevelBi	HMDB0028973	207.0785	1.6199	44.6
Nifekalant	9.003E-8	1.244E-6	1.4072	4.6928	2.2304	up	LevelBii	HMDB0255595	464.2128	3.0074	44.6
Plazomicin	0.001613	0.0035	1.4047	1.7745	0.8274	up	LevelBii	HMDB0256636	615.3316	3.4686	47.7
Azepan-1-Yl-(6-Thiophen-2-Ylpyridin-3-Yl)Methanone	2.133E-5	8.99E-5	1.4028	0.3932	-1.3466	down	LevelBi	-	287.1236	4.6876	44.5
Congmunoside Xiv	2.613E-5	0.0001067	1.4034	3.3822	1.758	up	LevelBii	HMDB0039418	1165.5825	3.1208	45.8
2-Amino-4-Oxopentanoic Acid	1.412E-7	1.751E-6	1.4019	2.9316	1.5517	up	LevelBii	HMDB0244991	152.034	2.6799	51.8
Lnape(13:1/N-7:0)	0.001219	0.002747	1.4073	2.1127	1.0791	up	LevelBi	-	580.3235	6.4803	53.9
Byssochlamic Acid	1.629E-5	7.154E-5	1.4005	0.2314	-2.1114	down	LevelBii	HMDB0034262	353.1007	4.3708	49.3
N-[(5-Hydroxy-2-Pyridinyl)Methyl]Adenosine	9.28E-6	4.474E-5	1.4	0.4309	-1.2147	down	LevelBii	HMDB0040912	397.1227	4.2569	48.2
Pyropheophorbide A	8.109E-5	0.0002746	1.4012	0.4388	-1.1884	down	LevelBii	HMDB0256993	1113.5288	5.8032	50.3
Pgp(Txb2/A-13:0)	9.796E-6	4.665E-5	1.3972	1.7507	0.808	up	LevelBii	HMDB0274305	855.4096	3.0922	46.1
Isocolumbin	2.0E-6	1.252E-5	1.3979	0.3405	-1.5542	down	LevelBii	HMDB0036837	339.1213	4.1855	47.4
N-Fructosyl Isoleucine	7.609E-8	1.111E-6	1.3978	0.6349	-0.6554	down	LevelBi	-	294.1541	1.9424	55
Grazoprevir	2.005E-7	2.313E-6	1.3972	0.5838	-0.7765	down	LevelBii	HMDB0252939	747.3119	3.6111	45.6
Rebaudioside A	0.003456	0.006797	1.4032	0.2619	-1.9327	down	LevelBii	HMDB0034950	967.4383	5.9668	52.5
(E,E)-Futoamide	1.244	9.551	1.392	0.50	-0.9902	dow	Lev	HMDB0032654	322.1	2.2353	44.

	E-6	E-6		34		n	elBii		397		2
Cyclo(Pro-Phe-Trp-Lys-Thr-Phe)	0.007099	0.01274	1.3981	1.7332	0.7935	up	LevelBii	HMDB0250003	787.3943	2.1593	47.5
Tyr-Asp	0.0002725	0.0007621	1.3922	2.3057	1.2052	up	LevelBi	HMDB0029101	297.1059	1.8336	51.6
Fumonisin A2	0.0004078	0.001072	1.3935	1.8408	0.8803	up	LevelBii	HMDB0034699	712.3915	3.6363	43.6
Roxindole	0.001698	0.003658	1.3911	0.2668	-1.9059	down	LevelBii	HMDB0257319	405.2205	2.7743	47
2-Hydroxyadipic Acid	0.00045	0.001168	1.3847	0.4117	-1.2804	down	LevelBii	HMDB0000321	221.0657	2.0441	56.7
Tyr-Asn	0.001557	0.003395	1.39	2.1369	1.0955	up	LevelBi	HMDB0029100	296.1229	1.4008	51.3
Mevalonic Acid	3.414E-7	3.486E-6	1.3844	0.5173	-0.9509	down	LevelBii	HMDB0000227	212.0884	2.9943	48.6
Cyclo(Leu-Phe)	0.0001494	0.0004572	1.3838	3.5724	1.8369	up	LevelBii	HMDB0094673	319.1646	4.9274	48.6
Pg(7:0/18:5)	6.812E-5	0.0002373	1.38	2.1804	1.1246	up	LevelBi	-	635.3004	3.4705	46.8
(2E)-3-(1H-Indol-2-Yl)-2-Propenoic Acid	0.002465	0.005072	1.3819	0.4089	-1.2903	down	LevelBi	-	188.0695	3.2023	52.4
N-Succinyl-L,L-2,6-Diaminopimelate	3.659E-5	0.0001403	1.38	2.6779	1.4211	up	LevelBii	HMDB0012267	325.0828	3.9344	46.9
Garcimangosone C	0.0005972	0.001492	1.3793	0.2111	-2.2442	down	LevelBii	HMDB0036984	471.1686	2.5194	49.2
13,14-Dihydro Pgf-1A	0.0005976	0.001493	1.3809	0.3972	-1.3321	down	LevelBii	HMDB0005076	357.2632	6.6837	48.2
2-Arachidonoylglycerol	0.001847	0.003943	1.3776	1.586	0.6654	up	LevelBi	HMDB0004666	379.2825	8.3007	44.6
Fahfa(22:4/22:3)	0.0009435	0.002201	1.3703	0.5263	-0.9261	down	LevelBi	-	663.5327	8.9824	50.1
Diisobutyl Cyclohexane-1,2-Dicarboxylate	1.688E-5	7.377E-5	1.3728	1.6302	0.7051	up	LevelBi	-	348.2121	2.7064	44.1
Histidyltryptophan	1.665E-6	1.081E-5	1.3756	0.2586	-1.951	down	LevelBii	HMDB0028896	376.1159	2.5853	48
5-O-Desmethyldonepezil	0.0004203	0.001101	1.3741	3.0964	1.6306	up	LevelBii	HMDB0013958	346.1786	3.2053	44.3
6-Hydroxypseudooxynicotine	1.521E-7	1.856E-6	1.3712	0.327	-1.6127	down	LevelBii	HMDB0240264	253.1187	2.2353	51.4
Lucidenic Acid A	0.00202	0.004262	1.3762	2.3435	1.2287	up	LevelBii	HMDB0037611	463.2489	2.35	45.2
Glucose	2.201E-7	2.481E-6	1.3698	0.565	-0.8236	down	LevelBii	HMDB0000122	179.0552	0.7177	53.2
3-Cyclopentylalanine	1.447	6.47E	1.3698	1.80	0.8522	up	Lev	-	199.1	3.321	47.

	E-5	-5		53			elBi		431		7
Ethyl Arachidonate	4.237E-5	0.0001584	1.3684	0.5417	-0.8845	down	LevelBi	HMDB0062725	333.2781	9.207	49.3
Fahfa(20:3/22:3)	0.001526	0.003337	1.3649	0.4927	-1.0213	down	LevelBi	-	637.5167	8.9731	50.1
Val-Cys	0.008975	0.0156	1.3826	2.2612	1.1771	up	LevelBi	HMDB0029124	221.094	1.6493	52.2
(5Alpha,6Beta,14Alpha,20R,22R)-5,6,14,20,27-Pentahydroxy-1-Oxowith-24-Enolide	4.65E-5	0.0001712	1.3675	1.7498	0.8071	up	LevelBii	HMDB0033198	471.2786	3.5177	42.2
Pi(Pgf1Alpha/20:0)	0.0004432	0.001152	1.3695	1.5389	0.6219	up	LevelBii	HMDB0277060	1021.619	7.6962	47.1
Fumonisin B1	0.001152	0.002612	1.3696	2.827	1.4993	up	LevelBii	HMDB0034702	722.3993	4.4138	44.1
Oxiracetam	3.223E-6	1.846E-5	1.3673	2.722	1.4447	up	LevelBii	HMDB0255986	123.0538	1.3528	48.9
Cyasterone	2.851E-7	3.037E-6	1.3602	1.5455	0.6281	up	LevelBi	HMDB0250618;LMST01040153	521.3108	2.7064	45
Ketoleucine	5.571E-7	5.099E-6	1.3575	0.3566	-1.4875	down	LevelBi	HMDB0000695;PW_C000547	130.0643	2.9943	44.5
Tropic Acid	0.0002804	0.0007809	1.3544	0.4607	-1.118	down	LevelBi	HMDB0062590	167.0686	3.7548	54.1
Corchoroside A	3.74E-6	2.091E-5	1.3545	40.2321	5.3303	up	LevelBii	HMDB0033846	593.2934	2.8497	44.5
(S)-2-((2-Hydroxy-2-Phenylethyl)Amino)-2-Methylpropan-1-Ol	0.0002054	0.0005968	1.3565	0.2463	-2.0214	down	LevelBi	-	242.1747	3.9416	55.2
1-Nitro-5-Glutathionyl-6-Hydroxy-5,6-Dihydronaphthalene	1.633E-5	7.17E-5	1.3542	0.0784	-3.673	down	LevelBii	HMDB0060326	531.0914	1.6973	46.1
6-(1-Naphthyl)-5-Nitro-2-Piperidinone	1.301E-5	5.914E-5	1.3548	0.4967	-1.0095	down	LevelBi	-	325.1183	3.4686	41.9
Oxytocin 1-8	6.522E-7	5.732E-6	1.3517	0.5288	-0.9193	down	LevelBii	HMDB0013033	935.3714	4.9551	45
Arg-Pro-Pro	1.526E-5	6.753E-5	1.3537	0.4285	-1.2226	down	LevelBi	-	373.1943	5.7323	48
Nifedipine	6.952E-6	3.512E-5	1.3511	0.5146	-0.9586	down	LevelBi	HMDB0015247;PW_C009534	347.1219	2.905	44.8
Glycerophosphoinositol	0.007849	0.01392	1.364	0.1399	-2.8371	down	LevelBii	HMDB0011649	333.0592	4.0091	53.5
Val-Asp-Arg	1.301E-5	5.914E-5	1.3495	1.5944	0.673	up	LevelBi	-	389.2122	2.2211	47.4
Desmethyldeschlorobenzoyl Indomethacin	3.702E-6	2.073E-5	1.348	0.1471	-2.7654	down	LevelBi	-	190.0499	3.2431	44.8
Polyporusterone B	0.006205	0.01134	1.3604	1.9753	0.9821	up	LevelBii	HMDB0038496	997.6247	7.9407	46.4
Mgdg(O-22:5/2:0)	0.000	0.000	1.3504	0.55	-0.8554	dow	Lev	-	595.3	7.0504	42.

	1766	5244		27		n	elBi		806		4
P-Salicylic Acid	4.999E-7	4.686E-6	1.3466	0.4385	-1.1895	down	LevelBi	HMDB0000500	137.0235	2.9979	52.6
Tyr-gamma-Glu	3.594E-5	0.000138	1.3456	1.8759	0.9076	up	LevelBii	HMDB0029119	308.1239	1.8366	47.9
20-Hete Ethanolamide	0.003495	0.006863	1.3536	2.396	1.2606	up	LevelBii	HMDB0013630	725.5506	6.7394	47.4
Mabioside D	7.325E-7	6.291E-6	1.3429	4.0097	2.0035	up	LevelBii	HMDB0040655	819.4577	3.2148	47.3
N-Cyclohexyl-3-(1-Phenyltetrazol-5-Yl)Piperidine-1-Carboxamide	0.0003116	0.0008551	1.3416	1.6701	0.74	up	LevelBi	-	355.2264	7.9787	48.4
Ile-Ala-Leu	1.931E-6	1.215E-5	1.3431	2.1409	1.0982	up	LevelBi	-	316.2226	3.686	48.3
3,4-Dihydroxyhydrocinnamic Acid	2.803E-5	0.0001129	1.3388	2.4995	1.3216	up	LevelBii	HMDB0000423	163.0389	1.846	54
Coumarin	0.003383	0.006668	1.3402	2.0683	1.0484	up	LevelBi	HMDB0001218	147.0427	1.8436	47.5
Dgdg(4:0/16:4)	7.08E-5	0.0002441	1.3435	5.153	2.3654	up	LevelBi	-	737.3427	3.3388	41.9
Uridine 5'-Monophosphate	7.695E-7	6.532E-6	1.3368	0.1501	-2.7361	down	LevelBii	HMDB0000288	323.0275	1.6973	54.7
Vinblastine	0.000367	0.0009824	1.3377	0.3991	-1.3253	down	LevelBii	HMDB0014710	845.3841	5.0198	47.8
3,4-Dihydro-2H-1-Benzopyran-2-One	1.578E-5	6.953E-5	1.3345	0.2305	-2.1173	down	LevelBii	HMDB0036626	193.0494	3.9066	54.5
Pyrrolidino Paf C-16	6.019E-5	0.0002141	1.3355	1.6807	0.7491	up	LevelBi	-	550.3834	8.6503	42
Tomatine	0.001022	0.002358	1.3362	0.5308	-0.9139	down	LevelBii	HMDB0034103	1092.5546	5.0105	47.9
4-Pregnen-17Alpha, 20Alpha-Diol-3-One	0.02204	0.03475	1.3251	2.117	1.0821	up	LevelBi	HMDB0011653	333.2439	7.9885	54.4
Deoxyloganic Acid	5.746E-7	5.223E-6	1.3285	0.2393	-2.0632	down	LevelBii	HMDB0037028	359.133	3.9531	50
Penitrem E	0.002927	0.005882	1.3351	5.5147	2.4633	up	LevelBii	HMDB0256241	620.3041	2.6894	46.6
5-Methoxy-6-(2-Propenyl)-1,3-Benzodioxole	6.341E-5	0.000223	1.3319	0.3167	-1.6589	down	LevelBii	HMDB0303894	227.0482	2.9322	46.9
Mimosine	0.0003191	0.000871	1.3289	2.1308	1.0914	up	LevelBii	HMDB0015188	243.0609	1.8648	53.2
Fa(22:6)	0.005135	0.009601	1.3239	0.4747	-1.0748	down	LevelBi	-	655.47	8.5757	53.5
(6E,9E)-Hexadeca-6,9-Dienedioylcarnitine	4.148E-5	0.0001559	1.3237	1.6853	0.753	up	LevelBii	HMDB0241489	430.2556	3.2715	44.5
6-Piperazin-1-Yl-Isoquinoline	4.782	2.577	1.324	2.21	1.1446	up	Lev	-	313.1	2.1912	43

	E-6	E-5		08			elBi		192		
1-Beta-Hydroxymedroxyprogesterone	0.0001913	0.0005607	1.3274	0.5324	-0.9095	down	LevelBii	HMDB0061065	424.2453	2.8159	43
Ziziphin	2.46E-8	4.742E-7	1.3207	1.8867	0.9158	up	LevelBii	HMDB0035750	1013.5755	4.4724	43.1
Pe(Pgf1Alpha/P-16:0)	3.17E-7	3.289E-6	1.3201	1.5314	0.6148	up	LevelBii	HMDB0285267	776.5416	7.7349	42.8
Oxyglutinosone	1.556E-7	1.893E-6	1.3194	0.5192	-0.9458	down	LevelBii	HMDB0302978	281.138	3.2337	47.6
Tyr-His-Asp-Asp	9.609E-5	0.000316	1.3212	0.4392	-1.1872	down	LevelBi	-	547.1772	2.4066	44.9
Apiosylglucosyl 4-Hydroxybenzoate	6.252E-6	3.218E-5	1.3143	0.4583	-1.1255	down	LevelBii	HMDB0035318	431.12	3.8785	47.7
Leukotriene E4	0.002209	0.004607	1.3222	2.5521	1.3517	up	LevelBii	HMDB0002200	474.2058	5.8865	50.8
Deoxynivalenol 3-Glucoside	0.0001075	0.0003471	1.3179	2.0756	1.0535	up	LevelBii	HMDB0039852	476.2132	3.0437	45.6
Pyroglutamylthreonine	1.893E-6	1.198E-5	1.3155	0.4614	-1.116	down	LevelBii	HMDB0341387	211.0713	2.1297	50.5
Lys-Pro	4.722E-5	0.0001734	1.3111	1.8787	0.9098	up	LevelBi	HMDB0028959	244.1644	2.1311	49.7
Ps(Monome(11,3)/Monome(13,5))	0.01495	0.02458	1.3213	2.4435	1.2889	up	LevelBii	HMDB0061604	894.5472	7.978	44.8
Dihydrogenistein	0.001027	0.002367	1.3146	0.3852	-1.3765	down	LevelBii	HMDB0005897	271.0597	5.0474	50.4
Ilekudinchoside D	0.003133	0.006249	1.3128	3.3556	1.7466	up	LevelBi	-	801.4466	4.2174	38.7
Bezitramide	0.0005062	0.001294	1.3107	1.6331	0.7076	up	LevelBii	HMDB0249171	534.2895	3.2418	46.2
Cofaryloside	0.01558	0.02551	1.3241	2.9235	1.5477	up	LevelBii	HMDB0035096	497.2766	3.0437	45.2
Pegorgotein	0.0004417	0.001149	1.3024	0.3224	-1.6329	down	LevelBii	HMDB0256203	174.0764	1.9403	56.6
Ethylestrenol	0.0004445	0.001155	1.3083	0.5642	-0.8258	down	LevelBii	HMDB0252076	271.2408	6.4371	46.4
Styrene	1.475E-6	1.048E-5	1.3047	0.703	-0.5083	down	LevelBi	HMDB0034240	105.0693	2.1616	55
Urofollitropin	5.401E-8	8.397E-7	1.3028	1.8119	0.8575	up	LevelBii	HMDB0259726	960.4092	2.6799	48.3
Isorhamnetin	0.000994	0.002302	1.3046	0.3067	-1.7053	down	LevelBi	HMDB0002655;LMPK12110002	315.0495	5.1949	52.2
Adomeglivant	6.065E-6	3.136E-5	1.3015	0.6359	-0.6531	down	LevelBii	HMDB0248020	573.2949	2.0512	48.3
Pi(3:0/18:5)	0.000	0.001	1.3024	5.76	2.5278	up	Lev	-	691.2	3.4141	45.

	5143	311		7			elBi		705		1
Isopentyl Isopentanoate	4.999<br>E-7	4.686<br>E-6	1.3003	0.22<br>13	-2.1757	dow<br>n	Lev<br>elBii	HMDB0030029	231.1<br>588	4.5557	55.<br>4
Sumithrin	0.000<br>2309	0.000<br>6602	1.2994	1.84<br>59	0.8843	up	Lev<br>elBi	HMDB0242366	368.2<br>21	2.3004	45.<br>4
Lippioside Ii	2.956<br>E-6	1.715<br>E-5	1.2951	0.51<br>69	-0.952	dow<br>n	Lev<br>elBii	HMDB0034268	589.1<br>278	2.5476	44.<br>7
Anandamide	0.002<br>185	0.004<br>564	1.3122	1.63<br>17	0.7064	up	Lev<br>elBi	HMDB0004080	348.2<br>867	8.2027	46
Lpc(O-14:0)	0.000<br>4265	0.001<br>116	1.2957	0.58<br>98	-0.7617	dow<br>n	Lev<br>elBi	LMGP01060007	498.3<br>165	7.7115	52.<br>8
1-Linoleoyl-Sn-Glycero-3-Phosphorylcholine	0.001<br>102	0.002<br>512	1.3002	1.31	0.3896	up	Lev<br>elBi	HMDB0010386	520.3<br>381	7.6582	51.<br>4
(7E,10E,13E,16E)-3-Hydroxydocosa-7,10,13,16<br>-Tetraenoylcarnitine	0.000<br>4342	0.001<br>133	1.2978	0.52<br>1	-0.9407	dow<br>n	Lev<br>elBii	HMDB0241607	546.3<br>722	6.729	47.<br>1
N-Acetyl-Dl-Tryptophan	2.443<br>E-6	1.479<br>E-5	1.2943	0.41<br>17	-1.2804	dow<br>n	Lev<br>elBi	-	245.0<br>917	3.8598	47.<br>1
Hyp-Pro	4.214<br>E-5	0.000<br>1579	1.2966	0.29<br>58	-1.7573	dow<br>n	Lev<br>elBii	HMDB0028871	227.1<br>018	2.6609	54.<br>6
2-Pyrrolidinone	2.197<br>E-6	1.355<br>E-5	1.2953	0.60<br>73	-0.7196	dow<br>n	Lev<br>elBi	HMDB0002039	86.05<br>98	0.6887	55.<br>5
N-Stearoyl Valine	2.315<br>E-5	9.631<br>E-5	1.292	0.25<br>24	-1.9863	dow<br>n	Lev<br>elBii	HMDB0241952	382.3<br>303	9.2051	53.<br>7
Lpc(18:3)	0.000<br>9753	0.002<br>264	1.2975	1.51<br>57	0.5999	up	Lev<br>elBi	HMDB0010387	562.3<br>13	7.294	55.<br>3
Arg-Thr-Lys-Arg	2.893<br>E-5	0.000<br>1155	1.2914	1.84<br>56	0.8841	up	Lev<br>elBii	HMDB0304796	524.3<br>406	3.4882	47.<br>5
(2E,4E)-3,5,7-Trimethylocta-2,4-Dienedioic<br>Acid	8.204<br>E-7	6.894<br>E-6	1.2899	0.44<br>9	-1.1552	dow<br>n	Lev<br>elBi	-	195.0<br>998	3.6661	43.<br>2
(4-Phenylbut-3-En-2-Ylideneamino)<br>2-Chlorobenzoate	0.000<br>2153	0.000<br>6203	1.2856	0.44<br>19	-1.1781	dow<br>n	Lev<br>elBi	-	282.0<br>688	3.9514	40.<br>2
Phe-Glu	0.004<br>633	0.008<br>773	1.2985	1.80<br>95	0.8556	up	Lev<br>elBi	HMDB0028994	295.1<br>273	2.1512	51.<br>9
Neoeriocitrin	2.04E<br>-7	2.342<br>E-6	1.2881	0.34<br>03	-1.5552	dow<br>n	Lev<br>elBii	HMDB0255521	577.1<br>529	3.5082	53.<br>6
Amobarbital	3.321<br>E-9	1.186<br>E-7	1.2885	0.58<br>55	-0.7722	dow<br>n	Lev<br>elBii	HMDB0015440	227.1<br>379	2.9249	46
Abu-Abu-Oh	0.003<br>018	0.006<br>039	1.2944	2.24<br>64	1.1676	up	Lev<br>elBi	-	295.0<br>924	1.8366	45.<br>2
Pc(Pgf1Alpha/P-18:0)	0.010<br>72	0.018<br>26	1.299	2.09<br>63	1.0679	up	Lev<br>elBii	HMDB0289626	866.5<br>955	7.7297	41.<br>7
Blumenol C O-[Rhamnosyl-(1->6)-Glucoside]	0.003<br>478	0.006<br>833	1.2926	2.26<br>06	1.1767	up	Lev<br>elBii	HMDB0031935	483.2<br>616	4.0498	48.<br>3
Tyrphostin B42	0.002	0.005	1.2883	0.13	-2.8906	dow	Lev	HMDB0244549	312.1	4.8049	50

	96	941		49		n	elBi		325		
(1-(3-Oxo-2-Aza-Spiro(4.5)Dec-2-Ylmethyl)-Cyclohexyl)-Acetic Acid	9.699E-5	0.0003187	1.2855	0.4596	-1.1215	down	LevelBi	-	308.2195	4.6876	43.3
1-Methyl-3-(2-Methyl-1-Propenyl)Cyclopentane	7.382E-5	0.0002532	1.2877	1.9247	0.9447	up	LevelBii	HMDB0061798	139.1478	7.1186	46.9
Pi(5-Iso Pgf2Vi/22:6(4Z,7Z,10Z,13Z,16Z,19Z))	0.001698	0.003658	1.2861	2.5142	1.3301	up	LevelBii	HMDB0278358	987.4594	3.4518	42.6
Thr-Lys	1.792E-8	3.676E-7	1.2819	0.6133	-0.7054	down	LevelBii	HMDB0029066	270.1432	1.9821	48.5
Trans-P-Coumaroyl Beta-D-Glucopyranoside	1.367E-7	1.707E-6	1.2818	2.0205	1.0147	up	LevelBii	HMDB0302089	385.1123	3.4425	45.6
Pyroglutamic Acid	0.0001234	0.0003892	1.2794	1.5806	0.6605	up	LevelBi	HMDB0000267;PW_C000182	130.0491	0.6692	55.5
3,3-Dimethylacrylic Acid	0.002202	0.004594	1.283	0.5986	-0.7403	down	LevelBi	HMDB0000509	101.0588	0.7273	48.2
Pgp(Lte4/I-16:0)	0.000356	0.0009577	1.2822	0.5281	-0.9212	down	LevelBii	HMDB0275235	984.4618	5.7845	48.2
Methyl Dl-Pyroglutamate	4.637E-5	0.0001708	1.2786	0.0838	-3.5777	down	LevelBi	-	176.0905	1.6493	52
Vertilmicin	1.524E-5	6.748E-5	1.277	1.5501	0.6323	up	LevelBii	HMDB0242753	528.2835	2.8952	39.1
Asp-Pro-Lys-Ile-Ala	0.0001755	0.0005217	1.2774	1.751	0.8082	up	LevelBi	-	543.3085	1.9424	49.6
2,3-Dinor-Thromboxane B1	0.009566	0.01652	1.2779	1.7294	0.7902	up	LevelBii	HMDB0245413	363.2143	8.2907	52
Lauric Acid	0.0001045	0.0003394	1.2786	0.45	-1.1519	down	LevelBi	HMDB0000638;LMFA01010012	201.1841	8.0084	44.3
11-Hydroxyprogesterone 11-Glucuronide	0.0002783	0.0007758	1.276	1.9299	0.9485	up	LevelBii	HMDB0010364	487.2388	3.4048	45.6
Dihydrozeatin O-Beta-D-Glucoside	7.376E-5	0.0002531	1.2782	2.9309	1.5513	up	LevelBii	HMDB0302649	428.1783	3.5738	52.6
10-Hydroxy-3-Methoxy-1,3,5,7-Cadinatetraen-9-One	2.512E-5	0.0001035	1.2775	2.2113	1.1449	up	LevelBii	HMDB0036456	565.2841	8.6598	43.6
(2S,3R,4R,5S,6S)-2-(Hydroxymethyl)-6-Phenylmethoxyoxane-3,4,5-Triol	0.0002106	0.0006089	1.2768	0.4481	-1.158	down	LevelBii	HMDB0243491	269.1015	3.1396	46.1
Lys-Phe	0.01185	0.01997	1.2837	1.7386	0.7979	up	LevelBi	HMDB0028958	294.1805	1.5909	44.5
3-Hydroxybutyrylcarnitine	2.125E-7	2.422E-6	1.2716	0.5634	-0.8278	down	LevelBi	HMDB0013127;LMFA07070037	248.1485	1.5244	50.2
Pyrodone	6.95E-7	6.027E-6	1.2693	0.4524	-1.1445	down	LevelBii	HMDB0256988	320.1844	4.7335	51.2
Glu-Ile-Lys	3.132E-6	1.802E-5	1.2693	1.6383	0.7122	up	LevelBi	-	371.2278	2.1616	46.2
3,7-Dihydroxy-25-Methoxycucurbita-5,23-Dien	0.001	0.002	1.2704	0.49	-1.0277	dow	Lev	HMDB0039362	491.3	7.3049	43.

-19-Al	034	379		05		n	elBii		547		5
Pollenin A	0.001338	0.002976	1.2722	0.2176	-2.2004	down	LevelBii	HMDB0303704	301.0336	4.8166	46.1
Ps(6 Keto-Pgf1Alpha/16:0)	1.75E-8	3.622E-7	1.2664	2.0697	1.0494	up	LevelBii	HMDB0281139	830.4752	2.765	43.8
Acemetacin	0.001589	0.003457	1.2663	3.1029	1.6336	up	LevelBii	HMDB0247898	460.0804	1.7993	45.2
Asn-Met-Gly	0.0001535	0.0004679	1.2641	2.2041	1.1402	up	LevelBii	HMDB0341135	355.0863	7.8388	45.9
1,2-Dioctanoylglycerol	0.002454	0.005053	1.2644	0.3829	-1.385	down	LevelBii	HMDB0244086	403.2683	6.7394	43.9
(2Z,4'Z)-2-(5-Methylthio-4-Penten-2-Ynylidene)-1,6-Dioxaspiro[4.4]Non-3-Ene	2.926E-6	1.7E-5	1.2603	0.4626	-1.1123	down	LevelBii	HMDB0032660	234.0717	3.0238	44.3
Ile-Val-Asp	4.011E-5	0.0001517	1.2575	0.507	-0.98	down	LevelBi	-	346.1965	5.029	47.6
Nordihydrocapsaicin	0.001675	0.003616	1.2618	0.5691	-0.8132	down	LevelBi	HMDB0036328;LMFA08020319	294.2054	6.349	55.4
Mucronine D	0.001466	0.00322	1.266	1.6877	0.7551	up	LevelBii	HMDB0029335	666.3645	3.8235	43.7
Pe-Cer(12:2_2O/12:0)	0.01488	0.02447	1.2654	0.2785	-1.8441	down	LevelBi	-	1035.6996	9.5133	47.5
2',3',4'-Trihydroxyacetophenone	1.365E-8	3.062E-7	1.254	0.5432	-0.8805	down	LevelBi	-	167.034	2.4348	48.8
3-Chloro-N-(4-Imidazo[1,2-A]Pyrimidin-2-Ylphenyl)Benzamide	5.758E-7	5.229E-6	1.2551	0.3843	-1.3797	down	LevelBi	-	349.0883	2.7562	48.9
Sweroside	6.8E-6	3.449E-5	1.2519	2.0789	1.0558	up	LevelBii	HMDB0258643	417.1418	2.6326	46.6
7-Methylinosine	8.641E-7	7.173E-6	1.2536	0.5482	-0.8672	down	LevelBii	HMDB0003950	342.1176	1.8648	51.9
Cyclo(Trp-Asp-Pro-Ile-Leu)	0.004302	0.008225	1.2589	2.0719	1.051	up	LevelBii	HMDB0250629	642.3557	4.3943	42.3
2-(4-Chlorophenyl)-1-[3-(3-Cyclopropyl-1,2,4-Triazol-4-Yl)Azetidin-1-Yl]Ethanone	1.8E-7	2.134E-6	1.2499	0.5528	-0.8553	down	LevelBi	-	317.1194	3.0735	44
10,11-Dihydro-12R-Hydroxy-Leukotriene C4	0.0003447	0.0009322	1.2497	1.9527	0.9654	up	LevelBii	HMDB0060155	702.3246	2.4348	43.1
Benzoic Acid	0.0003313	0.000901	1.2488	1.6731	0.7425	up	LevelBi	HMDB0001870	123.0432	1.8436	52.1
Jwh 015-D7	0.0001423	0.0004392	1.2515	0.4382	-1.1904	down	LevelBi	-	333.2028	7.5643	41.4
Trp-Ile-Leu	0.01153	0.0195	1.2614	1.998	0.9985	up	LevelBi	-	431.2634	4.3843	45
Leu-Ser-Phe	0.001067	0.002444	1.2491	1.9685	0.9771	up	LevelBi	-	366.2006	3.41	43.5
Avenic Acid A	0.000	0.000	1.2487	0.35	-1.4764	dow	Lev	HMDB0030416	357.1	2.4066	44.

	2681	7509		94		n	elBii		049		1
Val-Pro-Ala	0.003597	0.007033	1.2505	0.1877	-2.4137	down	LevelBi	-	286.1769	6.2803	50.3
Astromicin	1.703E-6	1.1E-5	1.2425	1.6962	0.7623	up	LevelBii	HMDB0248678	428.2498	3.5573	47.9
2-[4-(Dimethylamino)Phenyl]-1,2,3,4-Tetrahydroquinazolin-4-One	3.298E-6	1.882E-5	1.244	0.5168	-0.9525	down	LevelBi	-	268.144	5.1367	51.7
[(4-Hydroxy-1,1-Dioxidotetrahydro-3-Thienyl)Amino]Acetic Acid	2.59E-7	2.825E-6	1.2428	1.6724	0.7419	up	LevelBi	-	192.0316	1.0788	49
Delimotecan	0.005009	0.009392	1.2518	2.8941	1.5331	up	LevelBii	HMDB0250970	655.2244	2.3969	45.4
Pc(Pgf2Alpha/18:1(11Z))	0.0005014	0.001282	1.2376	1.6796	0.7481	up	LevelBii	HMDB0286342	862.5501	7.7542	38.5
Phe-Val-Leu	0.0001899	0.0005573	1.2438	1.5734	0.6539	up	LevelBi	-	378.2366	3.9713	51.6
13-Hydroxy-5'-O-Methylmelledonal	0.001055	0.00242	1.2475	0.052	-4.2651	down	LevelBii	HMDB0035864	443.1675	2.2353	50.4
Hydroxypioglitazone	0.0001736	0.0005172	1.2409	3.2908	1.7184	up	LevelBii	HMDB0253285	407.0872	3.3483	43.3
Core Oligosaccharide	9.279E-7	7.576E-6	1.2376	0.3962	-1.3357	down	LevelBii	HMDB0250452	755.2449	1.1003	53.1
Stearic Acid Ethyl Ester	2.265E-5	9.469E-5	1.2382	0.5168	-0.9524	down	LevelBi	HMDB0034156	311.2941	7.8296	47.1
Trans-Hex-2-Enoic Acid	3.593E-6	2.019E-5	1.2394	0.6172	-0.6962	down	LevelBii	HMDB0010719	113.0598	3.6111	55.7
Cyclo(Prolylvalyl)	5.402E-8	8.397E-7	1.234	0.5324	-0.9093	down	LevelBi	-	197.1275	2.7362	52.5
Pe(O-8:0/10:0)	0.00238	0.004919	1.2387	1.5024	0.5873	up	LevelBi	-	961.6266	8.669	55.3
Mg(20:5)	0.0009621	0.002237	1.236	2.0572	1.0407	up	LevelBi	LMGL01010026	440.2744	6.7388	51.3
1-Acetyl-3,27-Dihydroxywitha-5,24-Dienolide 3-Glucoside	0.005517	0.01022	1.2387	2.2021	1.1389	up	LevelBii	HMDB0033729	707.3707	2.416	42.7
Fahfa(18:2/6:0)	3.869E-5	0.0001472	1.2287	0.6148	-0.7018	down	LevelBi	-	393.2989	9.2145	56.1
(2S)-2-Amino-7-(1-Aminoethylideneamino)-5-Sulfanylideneheptanoic Acid	0.001693	0.00365	1.236	2.4115	1.2699	up	LevelBii	HMDB0252980	276.1014	1.3241	46.8
Sulfolithocholic Acid	1.354E-6	1.018E-5	1.2309	1.8317	0.8732	up	LevelBii	HMDB0000907	457.261	2.9348	44.7
Bufotalin	9.307E-8	1.27E-6	1.2301	1.9132	0.936	up	LevelBii	HMDB0249432	462.2821	4.4527	43.7
Phe-Ile-Ser	0.0001603	0.0004845	1.2308	1.5112	0.5957	up	LevelBi	-	366.2013	2.4775	53.8
Cdp-Dg(5-Iso Pgf2Vi/18:2(9Z,11Z))	7.608	6.468	1.2286	2.02	1.0179	up	Lev	HMDB0291328	1068.	4.2688	54

	E-7	E-6		5			elBii		4625		
Flupirtine	1.084E-6	8.598E-6	1.2295	3.1725	1.6656	up	LevelBii	HMDB0252403	339.1002	3.5738	48.7
8-(3-Oxo-2-(Pent-2-Enyl) Cyclopentenyl)Octanoic Acid	4.109E-5	0.0001548	1.2292	0.6163	-0.6983	down	LevelBi	-	293.2096	5.7323	46.8
Jasmonic Acid	5.702E-6	2.979E-5	1.2286	0.5394	-0.8906	down	LevelBi	HMDB0032797;LMFA02020001	211.1316	5.0194	45.3
Val-Ile-Leu	0.0001143	0.000366	1.2287	1.4316	0.5177	up	LevelBi	-	344.253	3.4	56.4
Agavasaponin C	0.001664	0.003598	1.2321	0.5996	-0.7379	down	LevelBii	HMDB0033353	917.4671	6.9051	44.8
3-Dehydrosphinganine (C20)	0.0001321	0.0004122	1.2251	0.679	-0.5585	down	LevelBii	HMDB0304123	383.3397	7.4819	51
Fruleuile	8.612E-7	7.166E-6	1.2276	0.6354	-0.6542	down	LevelBi	-	407.2371	2.7762	44.6
Tigecycline	5.82E-5	0.0002079	1.2243	0.1979	-2.3373	down	LevelBii	HMDB0014700	584.2702	2.7839	50.6
Shexcer(10:0_2O/20:1_O)	0.003919	0.00757	1.2267	1.8398	0.8795	up	LevelBi	-	762.44	2.796	41.5
Retaspimycin	0.0003527	0.0009495	1.2293	1.5107	0.5952	up	LevelBii	HMDB0257169	570.3168	3.6363	44.6
Cholylcitrulline	0.02091	0.03317	1.2408	1.8135	0.8588	up	LevelBii	HMDB0242383	610.3703	8.1796	48.6
N-Arachidonoyl Alanine	3.04E-5	0.00012	1.2193	0.7047	-0.505	down	LevelBii	HMDB0241989	358.276	8.2812	44.8
Pinoquercetin	0.001205	0.00272	1.2257	0.4952	-1.0138	down	LevelBii	HMDB0256564	337.0309	5.2503	50.9
Annomuricatin A	7.455E-7	6.376E-6	1.2214	0.7027	-0.509	down	LevelBii	HMDB0303181	593.2504	3.6111	53.9
7-Hydroxyoct-2-Enoylglycine	1.228E-5	5.633E-5	1.2209	0.5838	-0.7765	down	LevelBii	HMDB0094774	236.0919	2.1593	43.1
Enkephalinamide-Leu, Ala(2)-	0.0003047	0.0008387	1.2205	1.6834	0.7513	up	LevelBii	HMDB0244970	551.3006	3.5276	42
Tyr-Glu	0.002491	0.005118	1.226	1.5623	0.6437	up	LevelBi	HMDB0029104	311.1224	1.9124	52.5
Mgdg(O-16:4/2:0)	0.0006287	0.001558	1.2221	2.5158	1.331	up	LevelBi	-	557.2937	4.4169	47.2
Phe-Asp	0.01583	0.02588	1.234	1.8536	0.8903	up	LevelBi	HMDB0028991	281.1135	2.1311	54.3
2'-Guanylic Acid	0.0001739	0.0005179	1.2211	0.5157	-0.9553	down	LevelBi	HMDB0240586	364.0632	2.0116	50.8
Asp-Pro-Ile	1.971E-6	1.235E-5	1.2149	1.5233	0.6072	up	LevelBi	-	344.1828	2.4099	44.4
Rifamycin	2.317	4.552	1.2153	2.39	1.2588	up	Lev	LMPK05000005	696.2	3.1583	45.

	E-8	E-7		3			elBi		951		2
Penitrem D	0.001372	0.003041	1.2182	2.8569	1.5145	up	LevelBii	HMDB0256240	612.3353	3.196	43.4
Candesartan Cilexetil	4.71E-8	7.576E-7	1.2134	2.8185	1.4949	up	LevelBii	HMDB0249581	609.2494	3.273	47.6
Ala-Ile-Phe	0.0002503	0.0007069	1.2134	1.4616	0.5475	up	LevelBi	-	350.206	3.5078	55.1
Goyasaponin Iii	0.003262	0.006465	1.2162	2.24	1.1635	up	LevelBii	HMDB0038342	949.4814	3.0829	47.9
2Beta-Hydroxytestosterone	0.0002052	0.0005962	1.2105	0.4461	-1.1646	down	LevelBii	HMDB0012654	349.1992	5.5908	54.7
Tyr-Tyr	0.0001215	0.0003845	1.2125	1.668	0.7381	up	LevelBi	HMDB0029117	345.1426	2.2012	46.7
Gly-Trp	0.0005179	0.001319	1.2115	2.62	1.3896	up	LevelBii	HMDB0028852	282.0852	2.3208	55.9
N-Linoleoyl Glutamine	1.596E-8	3.407E-7	1.2097	1.4084	0.4941	up	LevelBii	HMDB0241975	447.2588	2.2904	45.8
Leustroducsin B	0.002247	0.004676	1.2138	1.911	0.9343	up	LevelBii	HMDB0254044	668.3609	3.0639	45.4
Val-Ile-Val	0.0002668	0.0007477	1.2122	1.6656	0.736	up	LevelBi	-	330.2368	2.8357	47.2
Lobucavir	0.001225	0.002756	1.2207	0.0809	-3.6278	down	LevelBii	HMDB0254142	310.1182	4.7981	46.4
Glu-Leu-Leu	0.01201	0.0202	1.2188	1.6858	0.7535	up	LevelBi	-	374.2258	3.4295	44.8
Glucosyl (E)-2,6-Dimethyl-2,5-Heptadienoate	1.736E-6	1.118E-5	1.2073	0.5206	-0.9417	down	LevelBii	HMDB0035136	361.1479	3.8507	49.2
Methyl (2R)-Piperidine-2-Carboxylate	7.687E-7	6.528E-6	1.2065	0.5397	-0.8898	down	LevelBi	-	144.1011	0.9516	55.9
Tyr-Arg	0.001256	0.002817	1.2075	1.5485	0.6309	up	LevelBi	HMDB0005768	338.1808	1.2161	45.8
Trp-Asp-Asp	5.502E-5	0.0001976	1.2033	0.6093	-0.7148	down	LevelBi	-	435.1488	2.2607	45.6
Daglutril	1.249E-6	9.574E-6	1.2009	1.6988	0.7645	up	LevelBii	HMDB0250830	593.2911	2.0345	45.3
4-Phenyl-1,2,3-Thiadiazole	0.0001329	0.0004148	1.1984	1.7759	0.8285	up	LevelBi	-	204.0604	0.7955	43.1
[2-Amino-2-(Hydroxymethyl)-5-(4-Octylphenyl)Pentyl] Dihydrogen Phosphate	7.511E-6	3.745E-5	1.1991	3.3064	1.7253	up	LevelBii	HMDB0252505	400.2291	1.846	45.9
Trimethylamine N-Oxide	9.508E-5	0.0003135	1.2008	0.6357	-0.6537	down	LevelBi	HMDB0000925	58.0652	0.6983	54.8
Trp-Val-Phe	0.001144	0.002596	1.2006	1.6868	0.7543	up	LevelBi	-	451.2308	4.2174	47.3
Gly-Lys-Phe	0.003	0.006	1.1997	1.56	0.6447	up	Lev	-	351.2	1.9026	44.

	296	516		34			elBi		026		9
Subaphylline	0.004147	0.007962	1.1998	1.5389	0.6219	up	LevelBii	HMDB0033463	527.2849	2.6799	48.9
Pip(Pgf2Alpha/16:2(9Z,12Z))	0.0003112	0.0008541	1.1966	0.6071	-0.7199	down	LevelBii	HMDB0278654	1029.4502	6.896	52.4
3,4-Diethoxybenzaldehyde	8.289E-5	0.0002797	1.1957	0.537	-0.8969	down	LevelBi	-	217.0842	5.0781	46.6
5-Amino-1-Pentanol	1.707E-9	7.636E-8	1.193	0.7376	-0.4391	down	LevelBi	-	104.1064	0.6692	46.6
Butaclamol	0.0007071	0.001721	1.194	1.7712	0.8247	up	LevelBii	HMDB0242247	396.2102	3.1301	46.3
Starch, Pregelatinized	2.66E-5	0.0001081	1.1924	2.0224	1.0161	up	LevelBii	HMDB0303308	691.2599	3.6564	48.4
N-[(3A,5B,7A)-3-Hydroxy-24-Oxo-7-(Sulfooxy)Cholan-24-Yl]-Glycine	0.0008436	0.002	1.1933	1.7881	0.8384	up	LevelBii	HMDB0002496	530.2746	2.6069	42.1
Sulfolithocholylglycine	0.004608	0.008732	1.1939	1.8859	0.9152	up	LevelBii	HMDB0002639	512.2671	2.2926	43.9
Cucurbitacin S	0.0002002	0.0005835	1.1917	0.5579	-0.8419	down	LevelBii	HMDB0250583	537.2566	5.9078	48.4
3,10-Dihydroxydodecanoylcarnitine	0.0008138	0.001938	1.1926	0.5831	-0.7781	down	LevelBii	HMDB0241292	417.2966	6.4371	51.4
Pc-M6	2.533E-5	0.000104	1.1885	2.3375	1.225	up	LevelBii	HMDB0038568	402.247	5.0474	48.3
Corticosterone	0.001432	0.003156	1.1899	2.0202	1.0145	up	LevelBi	HMDB0001547;LMST02030186;PW_C001190	347.223	7.6196	45.3
Abu-Lys-Oh	0.0001143	0.000366	1.1907	2.4922	1.3174	up	LevelBi	-	338.1327	2.0441	47.8
6|A-Hydroxy-7|A-(Thiomethyl)Spirolactone	0.000156	0.0004742	1.1848	2.554	1.3528	up	LevelBii	HMDB0246665	403.1979	3.4894	51
Lpc(O-20:2)	0.0118	0.01989	1.1911	0.3973	-1.3316	down	LevelBi	-	578.3781	8.5195	43.5
Linalool Oxide D 3-[Apiosyl-(1->6)-Glucoside]	0.0005568	0.001404	1.1881	1.8175	0.862	up	LevelBii	HMDB0031367	485.1997	3.1396	49.1
6-(2-Hydroxyethoxy)-6-Oxohexanoic Acid	3.721E-7	3.716E-6	1.1837	0.6032	-0.7294	down	LevelBii	HMDB0061681	254.0984	3.38	42.6
10,11-Dihydro-12R-Hydroxy-Leukotriene E4	0.00128	0.002862	1.189	2.178	1.123	up	LevelBii	HMDB0012501	458.2566	1.9224	48.3
Hophe-Trp-Oh	8.379E-5	0.0002817	1.1801	2.3874	1.2554	up	LevelBi	-	472.1565	2.1976	44.8
2-Methyl-4-(Pyridin-2-Yl)But-3-Yn-2-Ol	0.0001728	0.0005147	1.1822	0.6708	-0.576	down	LevelBi	-	144.0799	2.8653	46.8
Lentialexin	4.847E-5	0.0001771	1.1798	0.4166	-1.2634	down	LevelBii	HMDB0038997	121.0638	5.0781	48.1
Hemicalyculin A	0.013	0.022	1.1956	1.52	0.613	up	Lev	HMDB0253083	1006.	8.0959	46.

	76	81		94			elBii		6312		2
Octadec-9-Enedioic Acid	0.0008214	0.001954	1.1841	0.683	-0.55	down	LevelBi	-	313.2361	6.641	51.8
Pi(Pgf2Alpha/22:2(13Z,16Z))	0.0004451	0.001157	1.1823	1.5077	0.5923	up	LevelBii	HMDB0277722	1043.6008	7.6962	44.7
(11E,15Z)-9,10,13-Trihydroxy-11,15-Octadecadienoic Acid	1.66E-5	7.277E-5	1.1791	0.7344	-0.4453	down	LevelBi	-	327.2161	5.7291	49.9
Diprotin A	3.015E-5	0.0001192	1.1785	1.5059	0.5906	up	LevelBi	HMDB0251473	340.2227	3.3296	45.4
Guanosine	1.145E-8	2.702E-7	1.1785	1.7521	0.8091	up	LevelBi	HMDB0000133;PW_C000087	284.0977	1.9721	55.7
Shexcer(32:4_3O)	6.71E-7	5.861E-6	1.1751	1.6648	0.7354	up	LevelBi	-	806.4381	2.7743	42.8
Phe-Val	0.004001	0.007707	1.1799	1.6341	0.7085	up	LevelBi	HMDB0029008	529.2982	2.6767	43.3
Pro-Pro	2.505E-6	1.51E-5	1.174	0.6652	-0.5881	down	LevelBi	HMDB0011180	213.1222	0.737	44.8
Thr-Gln-Lys	0.001032	0.002376	1.171	0.6068	-0.7206	down	LevelBi	-	376.2208	2.7262	47.9
Phe-Leu-Ile	0.0001988	0.00058	1.177	1.4711	0.5569	up	LevelBi	-	392.2519	4.1879	48.4
Clarithromycin	1.088E-6	8.615E-6	1.1742	1.4149	0.5007	up	LevelBii	HMDB0015342	802.4858	7.6582	43.1
Achyranthoside D	0.0001605	0.0004849	1.1751	0.6269	-0.6737	down	LevelBii	HMDB0031023	1177.5225	7.5177	45.3
3-Ketosphingosine	0.0005136	0.00131	1.1757	1.4512	0.5373	up	LevelBii	HMDB0245914	280.2623	6.5446	47.9
Gabexate	9.198E-6	4.451E-5	1.171	1.684	0.7519	up	LevelBii	HMDB0252569	380.18	2.1688	45.1
Pa(5-Iso Pgf2Vi/18:3(6Z,9Z,12Z))	0.003192	0.006346	1.1763	0.5047	-0.9865	down	LevelBii	HMDB0264142	785.4291	7.8848	51.5
Dehydroxynocardamine	2.734E-5	0.0001107	1.1734	0.6686	-0.5808	down	LevelBi	-	585.357	2.1616	46.7
Nordihydroguaiaretic Acid	9.442E-5	0.0003114	1.17	1.5584	0.6401	up	LevelBi	HMDB0014325	341.1146	3.3603	44.3
Bioallethrin	9.378E-5	0.0003096	1.172	1.7362	0.796	up	LevelBii	HMDB0243551	366.2063	2.2806	49.3
(9E,12E)-N-(2-Hydroxyethyl)Octadeca-9,12-Dienamide	0.001422	0.003136	1.1788	1.357	0.4404	up	LevelBi	HMDB0247196	324.2882	8.2322	53.4
Dibekacin	0.0004025	0.001061	1.1687	0.6507	-0.6198	down	LevelBii	HMDB0251157	515.2809	4.7364	48.4
6-Oxooctadecanoic Acid	0.02919	0.04466	1.1679	1.822	0.8656	up	LevelBi	-	299.2568	7.5999	47.2
4-(4-Methylcyclohexyl)-4-Oxobutanoic Acid	0.000	0.000	1.1651	0.31	-1.6473	dow	Lev	HMDB0253742	199.1	5.7423	50.

	1566	4752		92		n	elBii		325		5
Scillipheosidin 3-[Glucosyl-(1->2)-Rhamnoside]	9.587E-5	0.0003156	1.1658	0.5313	-0.9125	down	LevelBii	HMDB0036946	743.3083	4.3522	45.2
Ile-Ser-Leu	7.244E-6	3.641E-5	1.1649	1.4785	0.5641	up	LevelBi	-	332.2164	3.3703	52.3
Mibefradil	0.001837	0.003924	1.1693	1.3855	0.4704	up	LevelBii	HMDB0254702	500.2718	7.8512	44.1
Sibrafiban	0.001947	0.004131	1.1644	1.8561	0.8923	up	LevelBii	HMDB0258287	465.1973	2.1105	46
Asp-Pro-Arg	0.0002045	0.0005949	1.1641	1.3951	0.4804	up	LevelBi	-	419.2265	3.1428	50.9
Azacitidine	0.0001852	0.0005453	1.1601	1.6621	0.733	up	LevelBii	HMDB0015063	225.0619	1.3521	50.1
Dn-Isobutylamide	2.146E-5	9.034E-5	1.159	1.6265	0.7017	up	LevelBii	HMDB0251551	401.2259	2.6369	45.2
His-gamma-Glu	9.057E-7	7.428E-6	1.1606	0.5247	-0.9305	down	LevelBii	HMDB0028899	264.1078	0.727	45
Val-Ser-Leu	3.724E-5	0.0001424	1.1576	1.5104	0.595	up	LevelBi	-	318.2009	2.905	51.5
Citrulline	1.703E-8	3.555E-7	1.1583	0.6685	-0.5811	down	LevelBi	HMDB0000904;PW_C000723	140.081	1.9523	48
Adenosine	1.154E-6	9.021E-6	1.1569	1.5884	0.6676	up	LevelBi	HMDB0000050;HMDB0247583;PW_C000034	268.1025	1.9224	54.7
Phe-Ile-Phe	0.004253	0.008146	1.1628	1.7108	0.7747	up	LevelBi	-	426.2365	4.3746	46
Pc(Pgf1Alpha/18:1(9Z))	0.02111	0.03344	1.1736	1.6794	0.748	up	LevelBii	HMDB0286512	840.5753	7.7297	44.7
N-Eicosapentaenoyl Aspartic Acid	0.0005864	0.001468	1.1586	0.6096	-0.7141	down	LevelBii	HMDB0242067	438.2292	5.4712	44.8
Tyr-Phe	0.0001073	0.0003468	1.1558	1.3558	0.4391	up	LevelBi	HMDB0029112	329.1482	2.8653	55.4
Beta-Sitosterol 3-O-Beta-D-Galactopyranoside	0.003576	0.007	1.1583	0.4269	-1.2279	down	LevelBii	HMDB0033949	597.4083	8.6411	43.6
Abeado	6.863E-6	3.475E-5	1.1514	0.2076	-2.2679	down	LevelBii	HMDB0247746	370.1592	2.6894	45.9
Pc(Pgf2Alpha/P-18:0)	0.003194	0.006349	1.1509	1.369	0.4531	up	LevelBii	HMDB0289560	844.5993	8.1635	43.3
Rutin	3.495E-6	1.972E-5	1.1468	0.5424	-0.8826	down	LevelBii	HMDB0003249	631.1236	3.4236	54
Verbasoside	1.37E-6	1.029E-5	1.1468	0.5735	-0.8022	down	LevelBii	HMDB0039233	463.1855	3.6068	42.8
N-[[3-Hydroxy-2-(2-Pentenyl)Cyclopentyl]Acetyl]Isoleucine	0.0007244	0.001755	1.1477	1.6961	0.7623	up	LevelBii	HMDB0041248	360.1927	2.979	45
Trans-Zeatin	1.87E	8.026	1.144	0.65	-0.6201	dow	Lev	HMDB0012204	274.1	1.6006	46.

	-5	E-5		06		n	elBi		278		9
(4E,6Z)-3-Hydroxydeca-4,6-Dienoylcarnitine	0.003843	0.007444	1.1581	1.5617	0.6431	up	LevelBii	HMDB0241110	345.2383	7.8512	49.9
Pterosin J	0.0001021	0.0003328	1.144	0.4368	-1.1949	down	LevelBii	HMDB0038161	273.0687	2.9979	41.2
Pirbuterol	0.0001043	0.000339	1.1395	0.4482	-1.1578	down	LevelBii	HMDB0015407	275.1183	2.3019	47.1
Spirolide E	0.007493	0.01335	1.1396	0.5205	-0.9421	down	LevelBii	HMDB0030494	760.4606	8.5851	48.4
Ethyl Cellulose	0.0007716	0.001853	1.1426	1.4356	0.5217	up	LevelBii	HMDB0033370	455.253	2.786	43.4
Roridin A	0.001735	0.003729	1.144	0.6357	-0.6535	down	LevelBi	-	533.2705	4.1586	43.3
Pro-Lys-Ile	0.001937	0.004112	1.1454	2.2749	1.1858	up	LevelBii	HMDB0341190	355.2337	2.0914	44.5
N6-Methyl-2'-Deoxyadenosine	0.0008464	0.002005	1.1438	1.9234	0.9437	up	LevelBii	HMDB0255300	529.2276	3.1583	50.1
Tyr-Pro-Leu	0.0009896	0.002294	1.1458	0.6661	-0.5863	down	LevelBi	-	392.2158	2.9645	53.3
(R)-6'-O-(4-Geranyloxy-2-Hydroxycinnamoyl)-Marmin	0.01143	0.01936	1.1425	2.2228	1.1524	up	LevelBii	HMDB0039061	635.2958	2.3204	42.7
4-Hydroxycoumarin	2.629E-6	1.563E-5	1.1384	0.1131	-3.1448	down	LevelBi	-	161.0235	3.6936	45.2
Cytidine	1.473E-6	1.048E-5	1.1386	1.4476	0.5337	up	LevelBii	HMDB0000089	242.0787	0.6618	50.2
Ethyl 2-Amino-3-Phenylpropanoate	0.0009755	0.002264	1.1381	0.5993	-0.7387	down	LevelBi	HMDB0252046	194.1167	2.8952	57.2
Betavulgaroside Ix	0.0001546	0.0004706	1.1384	0.6522	-0.6167	down	LevelBii	HMDB0034869	1147.512	7.5737	46.1
Lys-Pro-Ile	0.00991	0.01704	1.1469	4.7044	2.234	up	LevelBi	-	357.2464	2.3901	43.3
Val-Val-Phe	3.085E-5	0.0001213	1.137	1.3347	0.4166	up	LevelBi	-	364.2213	3.321	54.9
Pa(Pgf2Alpha/12:0)	0.0001824	0.0005381	1.1372	1.7196	0.7821	up	LevelBii	HMDB0262777	711.3836	2.3777	47.7
Plantainoside C	3.714E-8	6.342E-7	1.1368	0.6688	-0.5803	down	LevelBi	-	639.2265	3.6068	43.6
Leucomycin	0.0003045	0.0008385	1.1331	1.7495	0.8069	up	LevelBii	HMDB0254035	724.3903	3.38	41.2
Cinnamyl Anthranilate	0.0003154	0.0008636	1.1369	0.5023	-0.9933	down	LevelBii	HMDB0041319	312.1218	4.4169	48.6
Hyp-Val	0.008329	0.01464	1.1449	1.6874	0.7548	up	LevelBii	HMDB0028876	251.1021	2.082	48.2
Angiotensin Iv	7.552	0.000	1.1341	0.58	-0.7816	dow	Lev	HMDB0001038	819.3	5.5264	50.

	E-5	2584		17		n	elBii		978		3
Isoflupredone Acetate	1.847E-5	7.948E-5	1.1316	1.8744	0.9064	up	LevelBii	HMDB0253640	465.1936	2.491	44.4
Ile-Asn-Ile	2.432E-6	1.473E-5	1.1321	1.3275	0.4087	up	LevelBi	-	359.2275	3.3012	55.3
Stevioside	3.539E-5	0.0001364	1.1322	0.63	-0.6666	down	LevelBii	HMDB0034945	849.3723	5.7568	49
3-O-Beta-D-Galactopyranosyl-L-Arabinose	0.03378	0.0507	1.1568	0.2124	-2.2355	down	LevelBii	HMDB0038854	347.0747	4.6386	53.2
Leu-Leu-Phe	1.114E-5	5.201E-5	1.1305	1.4046	0.4902	up	LevelBi	-	392.2519	4.0598	52.1
Dopamine	6.915E-6	3.498E-5	1.1271	3.6623	1.8727	up	LevelBii	HMDB0000073	351.156	3.3671	50.4
Pe(16:0/0:0)	0.0002833	0.0007883	1.1284	1.2636	0.3375	up	LevelBi	LMGP02050002	454.2904	8.1537	56.5
Ile-Lys-Tyr	2.139E-6	1.325E-5	1.1275	0.7628	-0.3906	down	LevelBi	-	423.2575	2.3204	47.3
Cucurbitacin I	9.729E-5	0.0003191	1.1268	1.4177	0.5035	up	LevelBii	HMDB0250582	479.2828	3.9713	49.2
Epothilone B	1.464E-6	1.048E-5	1.1253	8.2643	3.0469	up	LevelBii	HMDB0251874	506.2583	3.1865	52.3
Dolichodial	0.000135	0.0004202	1.1277	0.4846	-1.0452	down	LevelBii	HMDB0251578	225.1119	5.3238	46.8
Deacetylchloronectrin	4.698E-7	4.482E-6	1.1265	0.7341	-0.4459	down	LevelBi	-	423.1967	2.0512	41.8
3-Butyn-1-Al	0.03603	0.05333	1.1258	0.55	-0.8626	down	LevelBii	HMDB0006853	69.0334	0.6887	51.5
Diferuloylputrescine	6.754E-7	5.892E-6	1.127	0.7639	-0.3885	down	LevelBii	HMDB0033468	439.1904	3.6111	50.8
Kaempferol 3-(3",6"-Diacetyl-2",4"-Di-P-Coumaroylrhamnoside)	0.0005363	0.001361	1.1287	0.5796	-0.787	down	LevelBii	HMDB0029503	805.1708	4.139	43.4
Cassiaside B	0.0002192	0.0006304	1.1271	0.5798	-0.7864	down	LevelBii	HMDB0038477	547.151	2.101	46
Ser-Ile	0.00288	0.005798	1.1325	0.5624	-0.8305	down	LevelBi	HMDB0029042	219.1332	2.1616	50.5
Lpc(11:0)	0.0001219	0.0003855	1.1225	1.4218	0.5077	up	LevelBi	LMGP01050007	448.2412	2.915	45.2
Tuberoside F	0.008407	0.01475	1.131	0.4549	-1.1364	down	LevelBii	HMDB0302986	1077.5425	6.5081	41.2
3,4',5-Trihydroxy-3',7-Dimethoxyflavanone	3.24E-7	3.338E-6	1.1198	0.7098	-0.4945	down	LevelBii	HMDB0037503	353.0663	2.5287	49.4
Ile-Met	3.125E-5	0.0001225	1.1186	1.3574	0.4409	up	LevelBii	HMDB0028913	261.1268	2.2829	47.3

3-Amino-2-Hydroxyheptanoic Acid	0.003729	0.007253	1.1223	1.7497	0.8071	up	LevelBi	-	194.1401	1.9026	43.9
Lys-Ile-Asn	0.011	0.01871	1.125	2.8799	1.526	up	LevelBi	-	374.2368	1.9026	43.7
N-Trans-Feruloyltyramine	6.732E-6	3.426E-5	1.112	0.6387	-0.6467	down	LevelBii	HMDB0029365	312.1222	5.6277	50.4
Moexipril	2.808E-5	0.000113	1.1119	0.5423	-0.8829	down	LevelBii	HMDB0014829	995.4645	4.4631	46.9
Imigliptin	0.00374	0.007272	1.1136	1.609	0.6862	up	LevelBii	HMDB0253428	341.191	2.2211	51.8
Val-Ala-Leu	0.000372	0.000994	1.1092	1.4827	0.5682	up	LevelBi	-	302.2055	2.8754	55.5
Traumatic Acid	3.276E-6	1.871E-5	1.1101	0.6331	-0.6595	down	LevelBii	HMDB0000933	227.1275	5.0198	45.9
Lps(18:2)	0.0003458	0.000934	1.1133	1.2756	0.3512	up	LevelBi	HMDB0240604	520.2657	8.5195	52.6
Maltotetraose	0.005013	0.009398	1.1076	0.3811	-1.3919	down	LevelBi	HMDB0001296	667.2244	1.0985	54.2
2-Phenylacetamide	8.389E-5	0.000282	1.1089	1.3745	0.4589	up	LevelBi	HMDB0010715	136.075	1.8436	49.8
5Beta-Androstan-3Alpha-Ol-17-One Sulfate	1.648E-8	3.48E-7	1.1092	2.4673	1.303	up	LevelBi	-	369.1765	2.463	41.7
Temocaprilat	6.424E-6	3.29E-5	1.1101	0.3472	-1.526	down	LevelBii	HMDB0061721	429.0965	3.0733	46.7
Purothionin Aii	0.0001248	0.000393	1.1124	1.7659	0.8204	up	LevelBii	HMDB0039001	553.2774	2.9249	45.8
Prenyl Glucoside	5.964E-7	5.36E-6	1.1074	0.6308	-0.6648	down	LevelBii	HMDB0031876	293.1228	3.0733	49.4
Lipoyllysine	0.000553	0.001397	1.1105	1.8185	0.8628	up	LevelBii	HMDB0012996	713.2731	3.2619	46.6
Phe-Gly	0.0006714	0.001646	1.1111	1.4074	0.4931	up	LevelBi	HMDB0028995	223.107	2.1616	55.4
7-Hydroxy-5-Methoxy-6,8-Dimethylflavanone	5.238E-5	0.0001897	1.1059	0.5279	-0.9218	down	LevelBii	HMDB0030695	333.0899	4.7243	47.1
2-Hydroxy-4-(Methylthio)Butanoic Acid	1.322E-7	1.662E-6	1.1082	0.746	-0.4227	down	LevelBi	LMFA01050480	149.0273	2.5194	54.8
Taurocholic Acid	8.809E-8	1.229E-6	1.1068	1.5586	0.6402	up	LevelBii	HMDB0000036	1075.5878	2.7176	45.7
4'-Hydroxycarvedilol	1.746E-6	1.123E-5	1.1086	0.6566	-0.6068	down	LevelBii	HMDB0013947	403.1696	2.5571	47.5
2-[[9-(1-Methylethyl)-6-[(Phenylmethyl)Amino]-9H-Purin-2-Yl]Amino]-1-Butanol	2.542E-6	1.528E-5	1.1088	1.4807	0.5663	up	LevelBii	HMDB0244822	355.2224	7.1186	45.8
[4-(4-Nitrophenyl)Piperazin-1-Yl](4-Propoxyphenyl)Methanone	0.0002157	0.0006215	1.1099	1.594	0.6727	up	LevelBi	-	370.1748	3.2222	50.1

Amphotericin B Methyl Ester	0.0009166	0.002146	1.109	1.7675	0.8217	up	LevelBii	HMDB0248359	960.4956	4.6876	42.8
Trp-Ala-Asn	2.228E-6	1.373E-5	1.1042	1.421	0.5069	up	LevelBi	-	390.1752	2.3204	43.5
Ethyl Palmitoleate	0.0002351	0.0006703	1.1032	0.7243	-0.4654	down	LevelBi	-	283.2614	9.0795	46.3
Dehydrotomatine	1.061E-6	8.45E-6	1.1031	1.8228	0.8661	up	LevelBii	HMDB0032002	1030.5128	3.2337	41.5
Henatinib	2.673E-8	5.051E-7	1.1029	1.9711	0.979	up	LevelBii	HMDB0253087	513.2161	1.2774	45.8
3-Oxo-5Beta-Cholanoic Acid	0.0002903	0.0008047	1.1005	0.614	-0.7037	down	LevelBii	HMDB0304950	355.2623	8.852	44.4
N-Cyclopropyl-2-Quinolinecarboxamide	2.627E-5	0.0001071	1.1017	0.645	-0.6326	down	LevelBi	-	245.1278	3.0833	46.4
2''-O-Acetylisoorientin	1.075E-8	2.608E-7	1.1	0.634	-0.6574	down	LevelBii	HMDB0038816	525.0822	2.5287	48.4
Pip(5-Iso Pgf2Vi/20:4(5Z,8Z,11Z,14Z))	0.005939	0.01092	1.1088	0.5767	-0.794	down	LevelBii	HMDB0280017	989.4475	5.9605	47.2
Withangulatin A	0.0004601	0.001189	1.0983	1.6336	0.7081	up	LevelBii	HMDB0037383	527.2627	2.8357	42.5
Pe(O-12:0/20:4)	4.777E-7	4.54E-6	1.0977	0.7288	-0.4564	down	LevelBi	-	668.4662	8.6598	42.6
Capsoside A	2.479E-5	0.0001024	1.1005	0.6321	-0.6619	down	LevelBii	HMDB0037613	659.3593	2.2111	40.4
Ethyl L-Tryptophanate	3.885E-6	2.158E-5	1.0953	0.5562	-0.8462	down	LevelBi	HMDB0252047	233.1269	3.3505	52.3
7,8-Epoxydihydrogrindelic Acid	2.352E-5	9.772E-5	1.0983	1.4928	0.5781	up	LevelBi	-	337.236	5.1952	49.5
Asn-Trp	0.001548	0.00338	1.0934	0.5782	-0.7903	down	LevelBi	HMDB0028742	319.1395	2.6069	51.3
Tunicamycin A	0.000985	0.002284	1.0986	0.6244	-0.6794	down	LevelBii	HMDB0259327	851.3886	5.5541	50.6
Isotonitazene	1.154E-7	1.488E-6	1.0942	1.3755	0.46	up	LevelBi	-	415.2092	2.2904	46.6
1-Isothiocyanato-8-(Methylthio)Octane	1.202E-5	5.539E-5	1.0933	0.6011	-0.7343	down	LevelBii	HMDB0038447	216.0865	2.642	47.5
Lpc(O-15:0)	0.0002467	0.0006983	1.0906	0.7638	-0.3887	down	LevelBi	LMGP01060009	512.3329	8.0341	55.9
Lpe(16:0)	0.0006734	0.00165	1.0929	1.2469	0.3183	up	LevelBi	HMDB0011473	452.2762	8.1523	54.2
Pro-Tyr-Ile	0.005253	0.009795	1.0973	1.5161	0.6004	up	LevelBi	-	392.2156	3.0537	48.2
(Z)-15-Oxo-11-Eicosenoic Acid	0.003294	0.006515	1.0901	0.5714	-0.8075	down	LevelBii	HMDB0029797	323.2576	8.5568	47.7

Methyl Benzoylformate	8.816E-5	0.0002936	1.0878	1.4073	0.4929	up	LevelBi	HMDB0062605	165.0538	0.7856	47.5
Trimetrexate	1.351E-7	1.691E-6	1.0879	0.7385	-0.4374	down	LevelBii	HMDB0015288	390.1516	3.7948	48.2
Ergosine	0.01029	0.01764	1.0977	0.2016	-2.3105	down	LevelBii	HMDB0251913	582.2518	3.4425	46.7
Ala-Ile-Leu	0.0006522	0.001605	1.0876	1.3661	0.4501	up	LevelBi	-	316.222	3.4588	44.4
Hetacillin	0.0001408	0.000435	1.0882	0.637	-0.6505	down	LevelBii	HMDB0014877	388.135	3.8134	52.3
Lpg(16:0)	0.01164	0.01966	1.0879	1.3048	0.3839	up	LevelBi	HMDB0240601	483.2707	7.6016	47.5
Karanjachromene	0.0003411	0.0009235	1.0874	1.4074	0.493	up	LevelBi	LMPK12111544	339.0994	4.2764	45.3
Ile-Val-Val	2.949E-5	0.0001173	1.0847	1.3787	0.4633	up	LevelBii	HMDB0304801	328.2226	3.1301	48.4
Cyclopassifloside I	6.226E-5	0.0002198	1.0811	1.5922	0.671	up	LevelBii	HMDB0035944	697.4234	2.8497	46
Trp-Pro-Trp	0.004876	0.009175	1.0957	0.6054	-0.7239	down	LevelBi	-	488.2276	4.2569	48.2
Ginsenoside Rh4	0.0008285	0.001968	1.0857	1.7076	0.7719	up	LevelBi	HMDB0041534	643.4131	5.0389	44.1
Dg(Lte4/I-12:0/0:0)	0.0007192	0.001744	1.0853	0.7094	-0.4953	down	LevelBii	HMDB0298379	695.4447	5.8592	46.2
13-Hode	0.002156	0.004511	1.0873	1.3142	0.3942	up	LevelBii	HMDB0004667	295.2269	8.2907	46.1
Beta-Isorhodomycinone	0.005549	0.01027	1.0889	2.3987	1.2622	up	LevelBii	HMDB0249134	822.2999	3.9158	47.6
Beta-D-Xylopyranosyl-(1->5)-Alpha-L-Arabinofuranosyl-(1->5)-L-Arabinose	0.0004308	0.001126	1.0816	0.2696	-1.8911	down	LevelBii	HMDB0038860	413.1287	2.0063	53.8
Gliadins	0.0001657	0.0004976	1.0837	0.4302	-1.2168	down	LevelBii	HMDB0252744	652.2694	2.6515	45.2
Ala-Arg	4.308E-5	0.0001606	1.0837	0.7367	-0.4409	down	LevelBii	HMDB0028681	268.1392	1.5719	47.6
Ile-Arg-Leu	0.00859	0.01502	1.0912	1.566	0.647	up	LevelBi	-	401.284	2.7164	47.6
Lpe(19:0)	0.03163	0.04789	1.0862	2.2987	1.2008	up	LevelBi	LMGP02050028	494.3244	8.9077	43.2
Sacubitrilat	0.001128	0.002567	1.0802	0.4019	-1.315	down	LevelBii	HMDB0257440	404.1514	1.688	43.6
Phe-Ile-Ala	0.001013	0.00234	1.0803	1.4668	0.5526	up	LevelBi	-	350.2074	2.786	47.9
Formononetin 7-(6''-Methylmalonylglucoside)	0.0001537	0.0004683	1.0802	0.6948	-0.5254	down	LevelBii	HMDB0029494	1105.2822	3.7404	47.9

Canrenoic Acid	1.066E-6	8.485E-6	1.0786	0.6622	-0.5946	down	LevelBii	HMDB0249591	393.1831	1.9876	46.3
Lpe(18:3)	0.001283	0.002868	1.0798	1.4304	0.5165	up	LevelBi	HMDB0011478	474.2603	7.4709	54.4
(2R)-1-(2-Hydroxyethyl)-2,5,5,8A-Tetramethyl decahydronaphthalen-2-Ol	0.001513	0.003314	1.0814	0.7311	-0.4519	down	LevelBi	-	277.2154	6.641	46.7
Biotin-Xx Hydrazide	0.002911	0.005855	1.08	0.7184	-0.4771	down	LevelBi	-	523.2417	8.1247	49.6
Piriprost	1.165E-6	9.086E-6	1.0698	1.4464	0.5324	up	LevelBii	HMDB0256603	467.2888	3.0437	42.1
Glycocholate Glucuronide	0.01508	0.02479	1.0742	2.939	1.5553	up	LevelBii	HMDB0341324	661.3858	3.9713	40.6
Carnosic Acid Diacetate Methyl Ester	3.09E-6	1.783E-5	1.0693	1.4516	0.5377	up	LevelBi	-	431.2397	2.3602	39.2
Fahfa(18:3/3:0)	0.002192	0.004575	1.0734	0.6841	-0.5478	down	LevelBi	-	349.2363	8.5663	52.1
Dodecanamide,N-[3-(Dimethyloxidoamino)Propyl]	0.01974	0.03152	1.0609	0.5411	-0.886	down	LevelBi	-	301.2839	5.9375	52
Val-Val-Ile	6.09E-6	3.145E-5	1.0681	1.2895	0.3668	up	LevelBi	-	330.2374	3.133	56.5
Harmol Glucuronide	0.000932	0.002178	1.0694	1.4056	0.4911	up	LevelBii	HMDB0253051	339.0992	3.7845	49.1
Pe(Pgf2Alpha/P-18:1(11Z))	0.0003179	0.0008687	1.0653	1.3514	0.4344	up	LevelBii	HMDB0285409	800.5378	7.7251	43.8
Uridine, 2'-Deoxy-2'-Fluoro-2'-Methyl-, (2'R)-	4.897E-5	0.0001788	1.0652	0.7107	-0.4927	down	LevelBii	HMDB0247529	305.0799	2.3304	48.2
Virginiamycin M1	0.009792	0.01686	1.0673	2.1796	1.1241	up	LevelBii	HMDB0259836	560.2191	1.9687	44.9
Pro-Ile-Leu	5.69E-6	2.975E-5	1.064	1.2835	0.3601	up	LevelBi	-	342.237	3.3308	53
(2S,5R)-5-Hydroxy-2-Piperidinecarboxylic Acid	7.971E-7	6.734E-6	1.0623	0.735	-0.4443	down	LevelBi	-	146.081	0.679	56.9
2-(2-Methylphenoxy)Acetohydrazide	8.187E-8	1.166E-6	1.0619	0.7611	-0.3938	down	LevelBi	-	213.1223	1.8534	47.8
Asp-Asp-Ile	0.0202	0.03217	1.0683	0.5276	-0.9224	down	LevelBi	-	362.1541	2.3104	41.2
Verdamicin	0.0255	0.03955	1.0691	1.5361	0.6193	up	LevelBii	HMDB0259789	444.2786	2.6069	43.6
Puromycin Aminonucleoside	1.479E-5	6.581E-5	1.0598	1.4679	0.5537	up	LevelBii	HMDB0256927	329.1135	2.2829	50.2
Leu-Met	4.807E-6	2.587E-5	1.0582	1.26	0.3334	up	LevelBi	HMDB0028935	263.1412	2.2806	55.2
Prostaglandin A1	0.0002583	0.0007268	1.058	0.5423	-0.8829	down	LevelBii	HMDB0002656	381.2259	5.5725	53.4

Ethyl Beta-D-Fructofuranoside	9.632E-7	7.811E-6	1.057	1.3815	0.4662	up	LevelBii	HMDB0303035	229.0684	2.0063	54
Phe-Val-Phe	0.006509	0.01182	1.0629	1.5423	0.6251	up	LevelBi	-	412.2207	4.0303	49.6
Thr-Val-Phe	5.562E-6	2.921E-5	1.0557	1.2733	0.3486	up	LevelBi	-	366.2006	2.9844	56.3
Dimethisterone	9.656E-6	4.613E-5	1.0543	0.8066	-0.3101	down	LevelBii	HMDB0251378	358.2762	8.3772	48.4
Pl	0.00141	0.003115	1.0592	1.3854	0.4703	up	LevelBii	HMDB0302594	547.2853	3.0238	50.1
Patuletin 7-Glucoside	0.0006351	0.001571	1.0577	0.5964	-0.7457	down	LevelBii	HMDB0303788	515.0775	4.1669	48
Lpc(20:5)	0.01746	0.02824	1.0705	1.5127	0.5971	up	LevelBi	HMDB0010397	586.3126	7.2754	54
Cosmosiin	0.002592	0.005296	1.0546	0.6198	-0.6901	down	LevelBi	HMDB0037340	433.109	4.1388	50.1
2-Amino-5-[(1-Carboxy-2-Phenylethyl)Amino]-5-Oxopentanoic Acid	7.754E-6	3.837E-5	1.0525	0.6803	-0.5557	down	LevelBi	-	295.1272	2.766	51.6
Alfentanil	0.006988	0.01258	1.0577	1.7471	0.8049	up	LevelBii	HMDB0014940	451.2264	7.6016	52.4
Ala-Arg-Tyr	5.122E-5	0.0001861	1.0495	0.7007	-0.513	down	LevelBi	-	409.2163	3.9615	49.1
Amikacin	0.001147	0.002602	1.0525	1.455	0.5411	up	LevelBii	HMDB0014622	590.2664	3.745	45.5
Ciprostene	6.957E-5	0.0002407	1.0502	0.7094	-0.4953	down	LevelBii	HMDB0250270	399.2332	6.0621	44.3
Thr-Ala-Ile	0.000394	0.001042	1.0502	1.4621	0.5481	up	LevelBi	-	304.1854	2.3901	52.6
Myotoxin A	0.002407	0.004965	1.0489	1.5749	0.6552	up	LevelBii	HMDB0254743	531.2578	2.6567	53.2
Fahfa(20:5/19:1)	0.01662	0.02702	1.0573	1.5285	0.6121	up	LevelBi	-	655.4969	8.3817	41.3
Furosine	0.02095	0.03322	1.0573	1.7142	0.7775	up	LevelBii	HMDB0029390	259.1039	2.3104	44.9
(3B,20R,22R)-3,20,27-Trihydroxy-1-Oxowitha-5,24-Dienolide 3-Glucoside	0.002376	0.004913	1.0441	1.5523	0.6344	up	LevelBii	HMDB0033573	676.3647	4.5896	44
Diethylphosphate	3.394E-6	1.927E-5	1.0427	0.6671	-0.5841	down	LevelBii	HMDB0012209	199.0363	1.1097	46.3
Thr-Pro	9.97E-6	4.735E-5	1.0385	1.7946	0.8437	up	LevelBii	HMDB0029069	215.1028	1.846	56.3
Adenosine Monophosphate	4.266E-5	0.0001592	1.0418	0.7174	-0.4792	down	LevelBi	HMDB0000045;PW_C000032	348.0687	1.9224	52.9
Aspartame	0.0116	0.01959	1.0514	1.4805	0.5661	up	LevelBii	HMDB0001894	293.113	2.1593	46.8

N-Eicosapentaenoyl Tryptophan	0.03644	0.05348	1.0498	0.6713	-0.575	down	LevelBii	HMDB0242081	530.3381	8.9906	45.8
Bidisomide	0.000577	0.001448	1.0413	1.5791	0.6591	up	LevelBii	HMDB0249194	408.2435	3.745	41.4
4-(Dimethylamino)Azobenzene N-Oxide	0.00499	0.009361	1.0346	0.6564	-0.6074	down	LevelBi	-	281.0942	3.2322	44
Ser-Ile-Leu	1.017E-5	4.81E-5	1.0385	1.3079	0.3872	up	LevelBi	-	332.2164	2.7762	40.4
8-Methyl-2-(Trifluoromethyl)Quinolin-4-Ol	2.826E-5	0.0001134	1.0379	0.7216	-0.4707	down	LevelBi	-	228.0614	3.8723	45
Blumenol C Glucoside	0.000103	0.0003352	1.0348	0.4109	-1.2832	down	LevelBii	HMDB0040668	417.2111	4.2132	48.9
Pe(O-16:4/4:0)	0.03712	0.0543	1.0497	1.3979	0.4832	up	LevelBi	-	1001.5616	7.7115	50.8
Cdp-Dg(Pgj2/I-16:0)	0.0005124	0.001307	1.0298	2.0206	1.0147	up	LevelBii	HMDB0293707	1012.4714	2.576	45.4
Lactobionic Acid	7.531E-6	3.754E-5	1.0347	2.8568	1.5144	up	LevelBii	HMDB0253945	379.0852	1.9687	46.1
Abscisic Alcohol 11-Glucoside	1.85E-5	7.955E-5	1.0347	1.367	0.451	up	LevelBii	HMDB0039636	476.2244	2.1616	55.2
Quinceoxepine	1.683E-6	1.09E-5	1.0358	1.5283	0.612	up	LevelBii	HMDB0038107	179.1422	7.1087	45.5
17-Phenyl-18,19,20-Trinor-Prostaglandin D2	3.759E-5	0.0001435	1.0353	1.33	0.4114	up	LevelBii	HMDB0062679	387.2193	2.1311	39.1
Lpc(20:3-Sn1)	0.003163	0.006297	1.0396	1.2534	0.3259	up	LevelBi	-	546.3534	7.8512	46.6
Quercetin 3-(6"-Malonyl-Glucoside)	0.0006152	0.001529	1.0416	0.1979	-2.3369	down	LevelBii	HMDB0037368	531.0744	3.8321	44.1
Phe-His-Glu	1.038E-6	8.296E-6	1.0323	1.3207	0.4014	up	LevelBi	-	432.188	2.0614	48
(2S)-2-[[(2R,3S)-2-Amino-3-Methylpentanoyl]Amino]-4-Methylpentanoic Acid	9.21E-5	0.000305	1.0324	1.207	0.2714	up	LevelBi	-	245.1859	2.796	59
Lys-Trp	0.000285	0.0007922	1.0337	0.6506	-0.6201	down	LevelBii	HMDB0028962	355.1708	4.3449	46
Kalkitoxin	0.02141	0.03389	1.0473	1.4986	0.5836	up	LevelBi	-	367.2795	7.8804	44.4
11-Hydroxy-9-Tridecenoic Acid	5.888E-5	0.00021	1.0284	0.6468	-0.6287	down	LevelBii	HMDB0035881	273.1693	5.0382	46.9
Pyroglutamylisoleucine	5.443E-5	0.0001958	1.0277	1.3333	0.415	up	LevelBii	HMDB0341381	241.1185	3.273	52.3
Gln-Arg-Thr	0.001596	0.00347	1.0305	0.7006	-0.5133	down	LevelBi	-	445.2503	6.035	46.6
Gly-Phe-Val	3.403E-5	0.0001318	1.0264	1.4565	0.5425	up	LevelBi	-	322.1742	2.3901	44.2

9-Cis-Retinol	0.001128	0.002567	1.0297	0.7556	-0.4043	down	LevelBi	HMDB0006217;LMPR01090009;PW_C002666	287.2342	6.447	50.5
Tyr-Lys	0.003387	0.006675	1.0306	1.4365	0.5226	up	LevelBi	HMDB0029110	310.1754	1.0591	50.9
Convallatoxin	0.003025	0.006051	1.0285	1.4176	0.5034	up	LevelBi	HMDB0034362;LMST01120013	551.2821	2.6865	43.7
(2S)-2-Amino-6-[(3-Formylpiperidin-1-Yl)Amino]Hexanoic Acid	8.95E-6	4.354E-5	1.0246	1.4407	0.5268	up	LevelBii	HMDB0255325	316.1867	2.7743	48.4
Methylpyrazine	1.825E-8	3.723E-7	1.0249	0.7526	-0.4101	down	LevelBii	HMDB0033112	95.06	0.6298	49.4
(5Alpha,17Beta)-17-Benzoyl-4-Azaandrost-1-En-3-One	0.007957	0.01408	1.029	1.9333	0.951	up	LevelBii	HMDB0253725	412.2048	3.4141	46.4
Tyr-Ala-Leu	0.0001172	0.0003735	1.0226	1.379	0.4636	up	LevelBi	-	366.2002	2.5772	44.3
Lpc(20:4-Sn1)	0.00494	0.00928	1.0303	1.2271	0.2952	up	LevelBi	-	544.3365	7.6294	51.9
12-Hydroxy-12-Octadecanoylcarnitine	0.01818	0.02929	1.0338	0.5406	-0.8873	down	LevelBii	HMDB0013154	488.3563	7.1456	53.7
Dlcl(18:2/22:7)	0.003881	0.007509	1.0277	1.4856	0.5711	up	LevelBi	-	991.4699	3.1865	42.8
Pe(Pgf1Alpha/P-18:0)	0.002071	0.004354	1.0244	1.4513	0.5374	up	LevelBii	HMDB0285371	858.5805	8.1635	46.1
1H-Indole-4-Carboxaldehyde	0.00504	0.009444	1.0276	1.4049	0.4905	up	LevelBi	-	146.0596	2.3901	54.7
1-(4-O-Beta-D-Glucopyranosyl-3-Methoxyphenyl)-3,5-Dihydroxydecane	7.903E-5	0.0002686	1.0195	0.6491	-0.6234	down	LevelBii	HMDB0303085	453.2833	6.4896	49.8
2-(5,8-Tetradecadienyl)Cyclobutanone	0.008538	0.01495	1.0231	0.7639	-0.3886	down	LevelBii	HMDB0037519	263.2354	8.7842	46.4
Gentamicin C	0.03769	0.05497	1.0247	1.8884	0.9172	up	LevelBii	HMDB0252688	470.2597	2.5665	49.4
Paeonol	7.347E-6	3.675E-5	1.0176	0.6929	-0.5292	down	LevelBii	HMDB0256081	225.0755	3.4613	54.2
Ile-Ile-Ile-Pro	0.0305	0.04641	1.0268	1.6201	0.6961	up	LevelBii	HMDB0304800	455.3204	3.843	42.9
Cer(8:0_2O/10:0)	0.03405	0.05106	1.0329	1.4056	0.4912	up	LevelBi	-	316.2835	6.4763	47.8
3-Phenyllactic Acid	5.871E-7	5.31E-6	1.0156	0.8418	-0.2485	down	LevelBi	HMDB0000779	165.0553	3.7495	50.7
Met-Trp	0.005671	0.01047	1.0216	1.3644	0.4483	up	LevelBi	HMDB0028984	336.1363	3.1132	53.7
Methyl 3-(Benzylamino)-2-Methylpropanoate	2.114E-5	8.92E-5	1.0138	0.6469	-0.6284	down	LevelBi	-	262.1424	5.5272	51.7
Phenylalanyl-L-2-Piperidinecarbonyl-N-(4-Nitro Phenyl)-L-Argininamide	1.73E-6	1.115E-5	1.0117	1.3631	0.4469	up	LevelBii	HMDB0253010	585.3097	3.5475	41.3

Trans-Delta-Viniferin 3''-Glucoside	8.645E-5	0.000289	1.0126	0.4466	-1.1628	down	LevelBii	HMDB0036366	597.1789	3.7123	46.1
Pc(Pgf1Alpha/P-18:1(9Z))	7.188E-5	0.0002475	1.0133	1.3358	0.4177	up	LevelBii	HMDB0289834	842.5929	7.8481	45.7
Glu-Trp	0.003267	0.006471	1.0171	1.4806	0.5662	up	LevelBii	HMDB0028830	332.1244	2.2353	48.8
Thalrugosine	0.006137	0.01122	1.0207	0.55	-0.8624	down	LevelBi	HMDB0242612	609.295	2.1112	40.6
N-(5-Aminopentyl)Acetamide	2.253E-6	1.383E-5	1.0093	0.7419	-0.4307	down	LevelBi	HMDB0002284	145.1329	1.1771	50.7
Flurithromycin	0.0004346	0.001134	1.0093	2.1205	1.0844	up	LevelBii	HMDB0252409	732.4362	8.8614	43.4
Odalasvir	0.00705	0.01267	1.0175	1.7298	0.7906	up	LevelBii	HMDB0255919	999.5436	7.8296	41.2
Anapear	0.001323	0.002947	1.0133	0.6351	-0.655	down	LevelBii	HMDB0040285	155.1059	6.0938	48.4
Farnesylcysteine	4.571E-7	4.385E-6	1.0078	0.6814	-0.5534	down	LevelBii	HMDB0011627	326.213	6.7878	47.5
2-[3-(4-Phenylpiperazino)Propoxy]Benzamide	0.01232	0.02068	1.012	1.7033	0.7683	up	LevelBi	-	322.1903	4.6091	44
N-Tryptophylglycine	0.004917	0.009241	1.0142	1.3693	0.4534	up	LevelBi	-	262.1174	2.3104	55.3
Hexanoyl-L-Carnitine	0.002805	0.005666	1.0131	0.6902	-0.5349	down	LevelBi	-	260.1853	3.2517	56.9
Spinacetin 3-Gentiobioside	1.325E-6	9.997E-6	1.0073	0.7936	-0.3335	down	LevelBii	HMDB0037469	729.1887	3.7495	46.5
Citreoviridin D	1.188E-5	5.489E-5	1.0057	1.3986	0.484	up	LevelBii	HMDB0030522	417.2309	2.5469	46.3
Pro-Pro-Pro	1.196E-8	2.772E-7	1.007	0.8073	-0.3088	down	LevelBii	HMDB0256689	310.175	2.0614	47
Caffeic Acid	2.416E-8	4.687E-7	1.0056	0.6431	-0.6368	down	LevelBii	HMDB0001964	179.0336	3.2431	54.5
Posatirelin	0.006298	0.01148	1.0122	1.4312	0.5172	up	LevelBii	HMDB0256710	385.2421	2.3402	48.2
Methacycline	6.027E-6	3.12E-5	1.006	0.6733	-0.5706	down	LevelBii	HMDB0015066	463.1164	2.7365	45.9
Cdp-Dg(6 Keto-Pgf1Alpha/18:1(11Z))	1.964E-7	2.278E-6	1.0046	1.4344	0.5204	up	LevelBii	HMDB0291104	1092.5256	3.2337	48.9
De-O-Methylsimmondsin	2.624E-6	1.562E-5	1.0055	0.7272	-0.4596	down	LevelBii	HMDB0041208	360.1281	1.912	47.4
18R-Hepe	3.854E-5	0.0001468	1.0031	0.6856	-0.5446	down	LevelBii	HMDB0012611	319.2248	5.0586	45.9
Apstatin	6.018E-7	5.393E-6	1.0037	1.281	0.3573	up	LevelBii	HMDB0248539	459.2453	2.2012	49.6

Sphingosine	3.863E-5	0.0001471	1.0043	0.8134	-0.2979	down	LevelBi	HMDB0000252;LMSP01010001;PW_C000172	300.2877	6.8173	56
Leu-Ser-Pro	0.004663	0.008825	1.0106	0.7231	-0.4678	down	LevelBi	-	316.1864	6.349	45.4
Olamufloxacin	0.002966	0.005949	1.0131	0.4442	-1.1706	down	LevelBii	HMDB0255928	385.1673	3.3107	46.2
Vigabatrin	7.911E-5	0.0002688	1.0005	0.7036	-0.5072	down	LevelBii	HMDB0015212	174.0763	2.3398	48.8
(3E,5E,8Z)-Deca-3,5,8-Trienedioylcarnitine	3.29E-5	0.0001281	1.0032	0.5638	-0.8267	down	LevelBii	HMDB0241127	398.1791	3.804	53
Indinavir	8.062E-5	0.0002733	0.9965	2.1756	1.1214	up	LevelBi	HMDB0014369	612.3493	2.7461	45.2
S-Formylglutathionate(1-)	1.734E-5	7.541E-5	1.0005	0.7207	-0.4725	down	LevelBii	HMDB0062625	370.048	2.491	46.5
N-Carbobenzoxyglycyl-Prolyl-4-Methylcoumarinyl Amide	0.002968	0.005952	1.0002	1.5711	0.6517	up	LevelBii	HMDB0259966	522.1848	2.1593	45
Glyceryl Monolinoleate	0.02608	0.04038	1.0034	1.3829	0.4677	up	LevelBi	HMDB0242115	372.3092	8.4253	48.9
1-O-Hexadecyl-2-O-Ethyl-Sn-Glycero-3-Phosphocholine	0.001281	0.002865	0.999	0.8464	-0.2405	down	LevelBi	-	510.3887	8.8826	48.7
8-Chloroinosine	0.000231	0.0006604	0.9966	0.5773	-0.7926	down	LevelBii	HMDB0247428	347.0375	2.0157	42.4
Benzodioxole Fentanyl	1.511E-7	1.851E-6	0.9958	1.4632	0.5491	up	LevelBi	-	429.2157	3.1032	41.1
Met-Phe	0.002114	0.004434	1.0001	1.3456	0.4282	up	LevelBi	HMDB0028980	297.126	2.9645	47.9
O-Oxalylhomoserine	3.577E-5	0.0001376	0.998	0.6481	-0.6256	down	LevelBii	HMDB0302143	250.0563	1.9309	53.2
Ile-Val-Leu	4.834E-5	0.0001767	0.9968	1.226	0.2939	up	LevelBi	-	344.2533	3.5475	56.9

Table S3 Statistics of Differential Metabolites between GZSM and LSM

Metabolite	P_value	FDR	VIP_pred_OPLS-DA	FC(C/E)	Log2FC(C/E)	Regulate	level	Library ID	M/Z	Retention time
8-Deoxy-11-Hydroxy-13-Chlorogrosheimin	5.551E-9	8.147E-7	2.9691	0.0357	-4.807	down	LevelBii	HMDB0041037	357.114	2.8403
Meticillin	2.505E-7	1.019E-5	2.8226	0.0802	-3.6394	down	LevelBii	HMDB0015541	379.0959	2.8775
Sucrose	2.498E-8	2.266E-6	2.7093	22.848	4.514	up	LevelBi	HMDB0000258;PW_C000177	381.0771	0.6983
Tenatoprazole	3.011E-9	5.543E-7	2.6896	0.0536	-4.221	down	LevelBii	HMDB0258812	737.2202	2.8683
Inosinic Acid	2.215E-7	9.479E-6	2.5387	3440.0083	11.7482	up	LevelBi	HMDB0000175;HMDB0257686;PW_C000114	349.0531	1.4865
2Alpha-Methyl-5Alpha-Androstan-3Alpha-Ol-17-One	2.091E-6	4.142E-5	2.5239	0.0549	-4.1863	down	LevelBi	-	269.2252	6.4371
17-Hydroxy-15-Kauren-19-Oic Acid	0.0001611	0.0009747	2.5089	0.1519	-2.719	down	LevelBi	-	301.2143	5.8592
Malvidin 3-(6''-Acetyl-Galactoside)	7.258E-10	2.2E-7	2.494	0.1234	-3.0184	down	LevelBii	HMDB0301662	580.1383	2.8865
4-Demethylsimmondsin 2'-(E)-Ferulate	2.201E-6	4.263E-5	2.4725	0.1726	-2.5344	down	LevelBii	HMDB0034773	558.1572	2.8683
1-(2-Isopropylphenoxy)-3-Morpholin-4-Ylpropan-2-Ol	4.105E-9	7.064E-7	2.4359	38.7692	5.2768	up	LevelBi	-	280.1891	3.912
N-(5-Aminopentyl)Acetamide	1.411E-11	2.132E-8	2.4231	0.0363	-4.7822	down	LevelBi	HMDB0002284	145.1329	1.1771
Leucrose	3.359E-7	1.231E-5	2.4112	9.6947	3.2772	up	LevelBi	-	360.148	0.8735
4-(Difluoromethyl)-2-Hydrazinyl-6-(3-Methoxyphenyl)Pyrimidine	8.691E-7	2.255E-5	2.4082	4.7054	2.2343	up	LevelBi	-	231.0829	1.6688
Cyclohexylsulfamate	4.868E-9	7.755E-7	2.4035	0.4596	-1.1216	down	LevelBi	HMDB0031340	178.0541	2.8592
Poncirin	3.221E-8	2.597E-6	2.3975	4.6631	2.2213	up	LevelBii	HMDB0037487	593.1916	0.9324
15-Stachen-19-Ol	3.415E-5	0.0003061	2.3671	0.1832	-2.4486	down	LevelBi	-	289.2511	7.5701
2-{1-[2-(1-Benzothiophen-5-Ylamino)-2-Oxoethyl]Cyclohexyl}Acetic Acid	2.672E-13	3.837E-9	2.364	29.9323	4.9036	up	LevelBi	-	332.1332	1.8237
Fahfa(20:4/6:0)	1.272E-6	2.963E-5	2.3629	0.0067	-7.2116	down	LevelBi	-	417.2982	9.121
Rosuvastatin 5 S-Lactone	2.961E-6	5.282E-5	2.3509	7.8139	2.966	up	LevelBii	HMDB0060941	508.1532	0.727
Hypoglycin B	1.893E-6	3.858E-5	2.3454	0.1452	-2.7844	down	LevelBii	HMDB0029428	309.0856	4.7167
Apigenin	0.0002024	0.001162	2.3411	0.1583	-2.6593	down	LevelBi	HMDB0002124;LMPK12110005	271.0596	5.2537
3-Dehydrosphinganine (C20)	5.001E-5	0.0004106	2.3372	0.2403	-2.0568	down	LevelBii	HMDB0304123	383.3397	7.4819
Steviobioside	2.515E-6	4.679E-5	2.3283	0.0605	-4.0472	down	LevelBi	HMDB0036707	641.3133	6.0435
4,4-Dimethyl-2-[3-Carboxylatopropyl]-2-Tridecyloxazolidine 3-Oxide	4.293E-7	1.437E-5	2.3267	0.181	-2.4661	down	LevelBii	HMDB0246830	366.2991	8.5851
Proscillaridin	1.525E-8	1.614E-6	2.3108	0.0783	-3.6744	down	LevelBii	HMDB0256851	565.2597	5.8957
Gm3(30:2_2O)	0.0002466	0.001357	2.2948	0.2087	-2.2608	down	LevelBi	-	1139.6317	7.0625
12-Hydroxystearic Acid	0.0001223	0.0007934	2.2823	0.3781	-1.4031	down	LevelBi	HMDB0061706	301.2724	8.115

Fahfa(16:0/6:0)	1.052E-8	1.27E-6	2.2819	0.001	-9.9129	down	LevelBi	-	369.2989	9.4389
Fahfa(18:1/6:0)	2.355E-8	2.163E-6	2.2788	0.1175	-3.0898	down	LevelBi	-	395.3141	9.4389
Oxiracetam	1.933E-7	8.648E-6	2.2757	4.087	2.0311	up	LevelBii	HMDB0255986	123.0538	1.3528
(9Z,12Z)-7,8,17-Trihydroxyoctadeca-9,12-Dienoic Acid	2.447E-5	0.000236	2.2689	0.0706	-3.8234	down	LevelBi	-	329.2306	6.2901
(2E)-3-(1H-Indol-2-Yl)-2-Propenoic Acid	1.197E-9	2.914E-7	2.2688	0.0633	-3.9817	down	LevelBi	-	188.0695	3.2023
(3Ar,4S,7S,7As)-3A,4,5,6,7,7A-Hexahydro-1H-4,7-Methanoinden-6-Yl Propionate	4.144E-5	0.0003564	2.2595	0.0831	-3.5881	down	LevelBi	-	207.1372	5.8592
Cer(8:0_3O/14:1_(2Oh))	0.0001355	0.0008544	2.25	0.0504	-4.3093	down	LevelBi	-	384.3093	7.1967
Phaseollinisoflavan	7.961E-6	0.0001067	2.2473	0.1899	-2.3971	down	LevelBii	HMDB0034411	325.142	4.0206
Mabioside C	8.903E-5	0.0006195	2.2259	0.1213	-3.0437	down	LevelBii	HMDB0040653	757.4143	3.4491
5-Hydroxydecanedioylcarnitine	4.466E-10	1.527E-7	2.2222	16.787	4.0693	up	LevelBii	HMDB0241133	342.1907	3.6564
N-Trans-Feruloyltyramine	5.356E-6	7.991E-5	2.2195	0.0601	-4.0568	down	LevelBii	HMDB0029365	312.1222	5.6277
Inosine	7.415E-8	4.427E-6	2.2163	2.3431	1.2284	up	LevelBi	HMDB0000195;HMDB0247301;PW_C000127	269.0875	1.9919
Fluorescin	1.484E-11	2.132E-8	2.2154	34.2098	5.0963	up	LevelBii	HMDB0252365	393.1011	1.9876
Trans-Zeatin	1.395E-8	1.523E-6	2.2152	0.1569	-2.672	down	LevelBi	HMDB0012204	274.1278	1.6006
17(18)-Epoxy-5Z,8Z,11Z,14Z-Eicosatetraenoic Acid	0.0005027	0.002375	2.2135	0.3073	-1.7022	down	LevelBi	HMDB0010212	319.2255	5.8592
Apiosylglucosyl 4-Hydroxybenzoate	9.794E-8	5.389E-6	2.209	0.0073	-7.0933	down	LevelBii	HMDB0035318	431.12	3.8785
Chlorogenic Acid	1.675E-6	3.579E-5	2.1997	6.1438	2.6191	up	LevelBii	HMDB0003164	353.0863	2.7461
Tridec-10-Enedioylcarnitine	1.546E-7	7.552E-6	2.1939	0.0507	-4.3021	down	LevelBii	HMDB0241320	406.2189	5.8865
Oleic Acid Methyl Ester	0.00211	0.007294	2.1921	0.2408	-2.054	down	LevelBi	HMDB0034451	297.2768	8.5625
Cyclohexanesulfamic Acid	1.091E-10	7.211E-8	2.1842	0.2987	-1.7433	down	LevelBi	HMDB0031340	180.068	2.8357
Virginiamycin M1	0.0001111	0.0007408	2.1793	3.8358	1.9395	up	LevelBii	HMDB0259836	560.2191	1.9687
1,2-Cyclohexanedicarboxylic Acid, 4-Methyl-	2.19E-10	9.249E-8	2.1779	0.0817	-3.6139	down	LevelBii	HMDB0253138	204.1219	3.5573
(S)-3-Hydroxy-N-Methylcoclaurine	2.874E-7	1.104E-5	2.1763	0.0251	-5.3177	down	LevelBii	HMDB0006921	316.1536	4.0598
Benazeprilat	2.654E-8	2.31E-6	2.1643	2.3995	1.2628	up	LevelBii	HMDB0060582	431.1391	0.886
3-Phenylpropyl Acetate	7.187E-8	4.355E-6	2.1604	0.0007	-10.5589	down	LevelBii	HMDB0033386	223.096	5.6553
N-(2-Hydroxy-3-Methylbutanoyl)Leucine	6.259E-9	8.642E-7	2.1597	0.1047	-3.255	down	LevelBi	-	230.1385	5.0382
N-(1-Naphthyl)Ethylenediamine	9.96E-6	0.0001233	2.1565	0.3819	-1.3889	down	LevelBi	HMDB0254982	187.1221	3.0142
Asp-Trp	3.261E-5	0.0002945	2.1547	2.82	1.4957	up	LevelBi	HMDB0028764	320.1235	2.7762
N-Palmitoyl Gaba	2.871E-5	0.0002653	2.1521	0.2431	-2.0404	down	LevelBii	HMDB0062338	340.2829	8.5103

gamma-Glu-Cys-Gly	5.405E-8	3.633E-6	2.1471	2.4131	1.2709	up	LevelBi	-	308.0903	1.2553
Asp-Phe	1.769E-7	8.123E-6	2.1463	2.848	1.5099	up	LevelBi	HMDB0000706	281.1124	2.4294
Tetraethylene Glycol Monododecyl Ether	2.667E-5	0.0002515	2.143	0.0002	-12.4367	down	LevelBii	HMDB0246913	383.276	7.5643
Trans-13-Octadecenoic Acid	0.0002593	0.001413	2.1335	0.4109	-1.283	down	LevelBi	-	247.2412	8.115
Zearalanone	6.517E-7	1.863E-5	2.1312	0.0525	-4.2503	down	LevelBii	HMDB0259993	341.1366	4.0277
Serotonin	1.973E-6	3.973E-5	2.1259	0.3155	-1.6641	down	LevelBi	HMDB0000259;PW_C000178	177.1013	2.1311
Picrocrocin	0.001005	0.004091	2.1213	0.2098	-2.2526	down	LevelBii	HMDB0005796	705.3302	5.7015
Lactobionic Acid	9.456E-10	2.515E-7	2.1188	6.0148	2.5885	up	LevelBii	HMDB0253945	379.0852	1.9687
Alpha-Cyperone	0.0001062	0.000713	2.1183	0.1454	-2.7823	down	LevelBi	-	219.174	6.447
Kentsin	0.0001231	0.0007977	2.114	6.7206	2.7486	up	LevelBii	HMDB0005776	564.3258	6.2803
Kievitone Hydrate	0.000252	0.001384	2.1138	0.1224	-3.03	down	LevelBii	HMDB0038111	373.127	4.2596
(13R,14R)-7-Labdene-13,14,15-Triol	0.0006196	0.002805	2.1116	0.12	-3.0591	down	LevelBii	HMDB0034958	369.263	7.9313
Agnuside	4.014E-10	1.478E-7	2.11	0.1227	-3.027	down	LevelBii	HMDB0036561	431.1355	4.5992
Pa(Pgf1Alpha/20:0)	2.325E-6	4.435E-5	2.1029	3.2058	1.6807	up	LevelBii	HMDB0264713	822.5781	8.9021
Matairesinol	0.0002053	0.001176	2.0935	0.1705	-2.5518	down	LevelBii	HMDB0035698	357.1324	4.2873
Retaspimycin	6.276E-6	8.959E-5	2.0919	0.0601	-4.0573	down	LevelBii	HMDB0257169	570.3168	3.6363
Thr-Gln-Lys	4.828E-10	1.612E-7	2.0906	0.2425	-2.044	down	LevelBi	-	376.2208	2.7262
Adomeglivant	3.731E-6	6.2E-5	2.0859	0.3936	-1.3452	down	LevelBii	HMDB0248020	573.2949	2.0512
1-[[3-(3-Fluorophenyl)-1,2,4-Oxadiazol-5-Yl]Methyl]-4-Phenylpyrazine-2,3-Dione	2.096E-8	2.012E-6	2.0845	1.7852	0.8361	up	LevelBi	-	365.1044	0.708
2,6,10,10-Tetramethyl-1-Oxaspiro[4.5]Decan-6-Ol	0.000108	0.000723	2.0835	0.3806	-1.3935	down	LevelBii	HMDB0037907	213.1835	8.115
1-O-Caffeoylglucose	1.314E-6	3.031E-5	2.0815	4.6454	2.2158	up	LevelBii	HMDB0036937	341.0861	2.7176
Dodec-2-Enedioic Acid	1.626E-8	1.67E-6	2.0801	0.1293	-2.9516	down	LevelBii	HMDB0340663	246.1693	5.5564
N-Docosahexaenoyl Valine	0.0001786	0.001057	2.0785	0.309	-1.6943	down	LevelBii	HMDB0242025	460.3389	5.2049
15-Epi Prostaglandin A1	4.814E-5	0.000399	2.0775	0.2717	-1.8799	down	LevelBi	-	337.2354	5.8592
Rimcazole	1.615E-5	0.0001752	2.0774	2.8868	1.5295	up	LevelBii	HMDB0257233	342.192	3.3483
19-Noretiocholanolone 3-O-Glucuronide	0.0004197	0.002071	2.0769	0.1156	-3.1132	down	LevelBii	HMDB0341226	453.2456	6.0806
Fahfa(18:2/6:0)	4.024E-7	1.386E-5	2.0714	0.2964	-1.7545	down	LevelBi	-	393.2989	9.2145
St(24:1_O4_T/19:2)	5.18E-5	0.0004198	2.0711	4.367	2.1266	up	LevelBi	-	834.5484	9.0846
Tryptamine	0.000191	0.001114	2.066	0.4972	-1.0081	down	LevelBi	HMDB0000303;PW_C000207	161.1067	2.4197

16,17-Dihydro-16Alpha,17-Dihydroxygibberellin A4 17-Glucoside	1.173E-8	1.36E-6	2.0641	5.4532	2.4471	up	LevelBii	HMDB0040647	527.2094	1.9876
Pengitoxin	3.574E-5	0.0003184	2.0635	6.3204	2.66	up	LevelBii	HMDB0256225	1011.4531	2.2735
Lpc(O-20:2)	6.534E-8	4.115E-6	2.0623	0.023	-5.44	down	LevelBi	-	578.3781	8.5195
1-Methylene-5Alpha-Androstan-3Alpha-Ol-17-One Glucuronide	3.228E-7	1.199E-5	2.0619	0.2386	-2.0673	down	LevelBii	HMDB0341213	479.2605	5.2342
1-Methylene-5Alpha-Androstan-3Alpha-Ol-17-One	0.0001063	0.0007131	2.0601	0.4602	-1.1198	down	LevelBi	-	303.2307	7.0116
N-Acetylhistamine	4.405E-10	1.527E-7	2.0565	0.2066	-2.2752	down	LevelBii	HMDB0013253	154.0966	1.0886
Pgp(Pge2/I-14:0)	0.000136	0.0008567	2.0545	0.0936	-3.4174	down	LevelBii	HMDB0275018	905.3961	6.1478
Methyl Cinnamate	5.762E-6	8.434E-5	2.0523	0.1364	-2.8738	down	LevelBi	HMDB0303905;HMDB0033833	163.0744	4.0206
Formononetin 7-(6''-Methylmalonylglucoside)	7.387E-7	2.04E-5	2.0511	0.3024	-1.7257	down	LevelBii	HMDB0029494	1105.2822	3.7404
Delimotecan	0.0004048	0.002022	2.0464	4.0947	2.0338	up	LevelBii	HMDB0250970	655.2244	2.3969
Gambogic Acid	0.001591	0.005841	2.0428	0.4335	-1.2057	down	LevelBii	HMDB0034095	687.3194	6.0435
8-Hydroxylinoleic Acid	0.0001113	0.0007413	2.0409	0.3701	-1.4342	down	LevelBi	-	297.2411	7.0116
Vinyl Ether	5.289E-6	7.92E-5	2.0346	0.1146	-3.1254	down	LevelBii	HMDB0259824	185.0807	5.3422
Canrenoic Acid	0.0001448	0.0008982	2.0344	0.1754	-2.5109	down	LevelBii	HMDB0249591	393.1831	1.9876
N-Phenethylacetamide	1.97E-7	8.731E-6	2.0333	0.188	-2.4111	down	LevelBi	HMDB0255222	164.1061	4.1783
Monomenthyl Glutarate	1.574E-5	0.0001719	2.0332	60.7387	5.9245	up	LevelBii	HMDB0303264	334.1971	4.2074
Ethyl Beta-D-Fructofuranoside	2.068E-6	4.115E-5	2.0317	0.0706	-3.8235	down	LevelBii	HMDB0303035	229.0684	2.0063
Gingerglycolipid B	0.0002938	0.001565	2.0308	0.4195	-1.2533	down	LevelBii	HMDB0041094	659.3616	6.4618
Val-Phe-Val-Tyr	2.568E-7	1.032E-5	2.0237	0.3495	-1.5167	down	LevelBii	HMDB0304812	571.2819	2.0535
Carbadox	0.0001776	0.001052	2.0231	0.2368	-2.0782	down	LevelBii	HMDB0031762	307.0707	4.7218
N-(Oxolan-2-Ylmethyl)-2-[(2,3,5,6-Tetramethylphenyl)Sulfonylamino]Acetamide	6.629E-6	9.342E-5	2.0143	3.2615	1.7055	up	LevelBi	-	355.1711	3.6068
Emodin	0.00135	0.005129	2.0123	0.4287	-1.2219	down	LevelBi	HMDB0035214;LMPK13040008	269.0449	5.2503
Sipoglitazar	5.293E-7	1.654E-5	2.0122	0.0013	-9.5902	down	LevelBii	HMDB0258310	444.1378	4.4817
Ubiquinone-1	5.108E-5	0.0004158	2.0119	0.187	-2.4186	down	LevelBii	HMDB0002012	314.1369	4.4039
Cyclovirobuxine D	0.0006932	0.003058	2.0106	0.1328	-2.9128	down	LevelBi	HMDB0250688	407.3388	7.0892
(Z)-2-(5-Tetradecenyl)Cyclobutanone	0.00202	0.007058	2.0073	0.5078	-0.9776	down	LevelBii	HMDB0037543	265.2516	8.115
Fahfa(20:3/22:3)	0.0004889	0.002331	2.0048	0.2932	-1.7701	down	LevelBi	-	637.5167	8.9731
3-(4-Methylphenyl)-2-Propenenitrile	1.709E-8	1.728E-6	2.0042	0.6324	-0.6612	down	LevelBi	-	144.0808	2.4099
Epothilone B	4.207E-8	3.047E-6	2.0025	15.9985	3.9999	up	LevelBii	HMDB0251874	506.2583	3.1865

Lnape(18:3/N-3:0)	2.09E-5	0.0002106	2.0019	5.1443	2.363	up	LevelBi	-	590.3071	7.415
Val-Pro-Ala	0.0004112	0.002045	1.9987	2.2883	1.1943	up	LevelBi	-	286.1769	6.2803
Pro-Ile	2.227E-8	2.107E-6	1.9933	0.341	-1.5523	down	LevelBi	HMDB0304810	211.143	2.1912
Asp-Tyr	2.868E-7	1.104E-5	1.9915	2.9747	1.5727	up	LevelBi	HMDB0028765	297.1072	2.1311
1,2-Diphenylhydrazine	0.0009583	0.003938	1.9904	0.28	-1.8364	down	LevelBi	-	217.1321	2.9844
2-Hydroxyadipic Acid	2.692E-5	0.000253	1.9901	0.2283	-2.131	down	LevelBii	HMDB0000321	221.0657	2.0441
Acetyldiphenylalanyl-Leucyl-Aspartyl-Isoleucyl-Isoleucyl-Tryptophan	2.151E-5	0.0002146	1.9864	0.2631	-1.9265	down	LevelBii	HMDB0256173	968.4728	5.9973
Muridienin 4	0.00105	0.004224	1.9814	0.2097	-2.2533	down	LevelBii	HMDB0031906	577.4807	9.5225
Tropic Acid	7.148E-5	0.00053	1.9797	0.2709	-1.8839	down	LevelBi	HMDB0062590	167.0686	3.7548
Halosulfuron-Methyl	2.796E-12	1.194E-8	1.9787	0.2322	-2.1067	down	LevelBii	HMDB0034859	493.0592	2.5098
Pyrovalerone	0.001567	0.005775	1.9724	2.4749	1.3074	up	LevelBi	HMDB0256994	246.1846	6.2803
Cyclo(Arg-Gly-Asp-Phe-Val)	1.774E-7	8.123E-6	1.9703	3.3494	1.7439	up	LevelBii	HMDB0250680	595.2606	3.2919
(2'E,4'Z,8E)-Colneleic Acid	0.0001945	0.001127	1.9696	0.1762	-2.5046	down	LevelBii	HMDB0030995	329.1868	7.415
3-Ureidopropionic Acid	9.044E-8	5.094E-6	1.9659	0.2004	-2.3187	down	LevelBi	HMDB0000026	115.0495	1.1285
Isomaltotriose	1.96E-6	3.958E-5	1.9629	3.5203	1.8157	up	LevelBii	HMDB0341571	503.1597	1.0818
Isomaltulose	9.936E-9	1.22E-6	1.9623	1.6288	0.7038	up	LevelBii	HMDB0341167	377.0842	0.7177
Manninotriose	5.318E-6	7.955E-5	1.9607	6.0554	2.5982	up	LevelBi	HMDB0060468	487.1624	1.0985
Tramadol N-Oxide	0.0001414	0.0008805	1.9563	2.5781	1.3663	up	LevelBi	-	280.1892	3.7548
Capecitabine	8.216E-9	1.054E-6	1.9547	43.1064	5.4298	up	LevelBii	HMDB0015233	418.1634	4.1947
11Beta-Hydroxytestosterone	0.0007578	0.00327	1.9546	0.3361	-1.5729	down	LevelBii	HMDB0012533	349.1997	5.8494
N-3-Hydroxyoctanoyl-L-Homoserine Lactone	1.807E-6	3.734E-5	1.9516	0.1411	-2.8251	down	LevelBii	HMDB0341343	264.1231	5.1765
Pravastatin	2.273E-6	4.352E-5	1.9497	0.1997	-2.324	down	LevelBii	HMDB0005022	483.2561	5.9789
Pinoquercetin	0.0001903	0.001111	1.9494	0.1915	-2.3845	down	LevelBii	HMDB0256564	337.0309	5.2503
Cyclosquamosin D	0.0005146	0.002419	1.9422	0.2219	-2.1718	down	LevelBii	HMDB0303369	835.3926	5.5541
Ps(Monome(11,3)/Monome(13,5))	0.003542	0.009239	1.9391	2.8826	1.5274	up	LevelBii	HMDB0061604	894.5472	7.978
2',4',6'-Trihydroxydihydrochalcone 2'-Glucoside	1.033E-6	2.518E-5	1.9391	0.1369	-2.8685	down	LevelBii	HMDB0037483	441.1133	4.2596
Pip(Pgf2Alpha/16:0)	1.569E-5	0.0001715	1.9383	0.1797	-2.4759	down	LevelBii	HMDB0278446	1023.4594	5.5172
(4-Formyl-9-Methoxy-5-Methylnaphtho[2,3-B]Furan-3-Yl)Methyl Acetate	3.315E-6	5.706E-5	1.9361	0.2942	-1.7651	down	LevelBi	-	345.1303	4.2569
Liquiritin	0.0002122	0.001209	1.9343	2.0035	1.0025	up	LevelBii	HMDB0029520	383.1139	0.6692

Peonidin 3-(6''-P-Coumaroyl-Glucoside)	7.648 E-9	9.984 E-7	1.9323	2.795 7	1.4832	up	Leve lBii	HMDB0301664	630.1 294	1.9969
11-Hydroxyhexadeca-6,12-Dienoic Acid	2.061 E-7	8.97E -6	1.9261	0.030 7	-5.0246	dow n	Leve lBii	HMDB0340891	313.2 003	5.9696
Tryptoline	0.002 258	0.007 691	1.9259	0.226 1	-2.1452	dow n	Leve lBi	HMDB0012488	173.1 065	2.8555
Pe(Pgf1Alpha/24:1(15Z))	5.563 E-5	0.000 4433	1.9249	0.292 2	-1.7747	dow n	Leve lBii	HMDB0284643	882.6 224	8.9077
Ciprostene	0.000 3068	0.001 623	1.9237	0.358 8	-1.4789	dow n	Leve lBii	HMDB0250270	399.2 332	6.0621
Spinorphin	0.000 6419	0.002 887	1.9223	0.163 2	-2.6156	dow n	Leve lBii	HMDB0059790	875.4 637	6.7023
Trp-Trp	3.179 E-8	2.58E -6	1.9221	0.271 4	-1.8815	dow n	Leve lBi	HMDB0029094	391.1 739	3.804
Fumonisin A1	0.004 236	0.010 71	1.9161	0.366 5	-1.448	dow n	Leve lBii	HMDB0034713	822.4 102	6.4339
Timosaponin B Ii	0.002 449	0.008 119	1.9157	0.459 6	-1.1214	dow n	Leve lBi	-	925.4 683	7.6294
3-Ketosphingosine	2.539 E-5	0.000 2435	1.9146	0.241 7	-2.049	dow n	Leve lBii	HMDB0245914	280.2 623	6.5446
3'-Sialyllactose	2.068 E-8	2.007 E-6	1.9125	2.547 9	1.3493	up	Leve lBii	HMDB0000825	632.2 026	0.7365
1-Beta-Hydroxymedroxyprogesterone	5.56E -6	8.201 E-5	1.9119	0.386 6	-1.3711	dow n	Leve lBii	HMDB0061065	424.2 453	2.8159
N-[(5-Hydroxy-2-Pyridinyl)Methyl]Adenosine	3.168 E-6	5.539 E-5	1.9105	0.309 6	-1.6915	dow n	Leve lBii	HMDB0040912	397.1 227	4.2569
Dg(Pge2/I-14:0/0:0)	0.000 2236	0.001 258	1.9096	0.365 1	-1.4538	dow n	Leve lBii	HMDB0298774	681.4 538	5.8125
Arg-Pro-Pro-Gly-Phe-Ser-Pro	0.000 4789	0.002 289	1.9096	0.121 1	-3.0453	dow n	Leve lBii	HMDB0248578	761.3 786	3.3603
Octadec-9-Enedioic Acid	0.000 1235	0.000 7994	1.9074	0.481 3	-1.0551	dow n	Leve lBi	-	313.2 361	6.641
19-Oxoandrost-4-Ene-3,17-Dione	0.000 3524	0.001 81	1.9073	0.209 7	-2.2537	dow n	Leve lBii	HMDB0006768	645.3 448	6.5175
Guanosine	3.539 E-8	2.762 E-6	1.9073	2.287 3	1.1936	up	Leve lBi	HMDB0000133;PW_C000087	284.0 977	1.9721
Tri-N-Acetylchitotriose	5.156 E-5	0.000 4183	1.9055	0.085 8	-3.5422	dow n	Leve lBii	HMDB0006698	648.2 239	2.416
Tyr-His-Asp-Asp	6.08E -6	8.758 E-5	1.9044	0.255 4	-1.9693	dow n	Leve lBi	-	547.1 772	2.4066
Dgdg(4:0/16:4)	2.308 E-5	0.000 2261	1.9031	6.468 4	2.6934	up	Leve lBi	-	737.3 427	3.3388
Methyl (2S)-2-Amino-6-[(Tert-Butoxycarbonyl)Amino]Hexanoate	7.767 E-7	2.097 E-5	1.9014	0.296 7	-1.7531	dow n	Leve lBi	-	243.1 699	2.6467
Androsterone Glucuronide	0.000 3893	0.001 963	1.8992	0.422 5	-1.2428	dow n	Leve lBii	HMDB0002829	525.2 677	6.6005
Serofendic Acid	7.598 E-6	0.000 1037	1.8988	0.210 5	-2.2483	dow n	Leve lBii	HMDB0258253	417.1 866	5.8587
Fisetin	0.001 188	0.004 637	1.898	0.372 2	-1.426	dow n	Leve lBi	LMPK12111566	285.0 393	4.8996
Leucic Acid	4.325 E-8	3.09E -6	1.8978	0.056 6	-4.1437	dow n	Leve lBii	HMDB0000624	113.0 599	5.3422
N-Isobutyl-2-(2-Methyl-1H-Indol-1-Yl)Acetamide	2.384 E-5	0.000 2312	1.8917	0.174 4	-2.5192	dow n	Leve lBi	-	308.1 74	4.1978
Pg(O-12:0/22:4)	4.562 E-5	0.000 3845	1.8914	0.278 1	-1.8463	dow n	Leve lBi	-	727.4 903	8.9731

Methoxpropamine	4.205E-9	7.105E-7	1.8894	2.1347	1.0941	up	LevelBi	-	262.1791	3.4295
Glycinoeclepin C	8.204E-6	0.0001084	1.8883	5.099	2.3502	up	LevelBii	HMDB0037036	495.2424	2.2638
1,2,3,4-Tetrahydro-6-Propanoylpyridine	2.352E-8	2.163E-6	1.8882	0.1842	-2.4405	down	LevelBii	HMDB0034884	198.1124	4.38
Diphenylsulfone	0.001178	0.004609	1.8862	4.2063	2.0726	up	LevelBi	HMDB0251453	219.0463	5.3024
4-O-Beta-D-Glucosyl-4-Coumaric Acid	7.008E-8	4.319E-6	1.8833	2.0214	1.0153	up	LevelBi	HMDB0039509	344.1329	2.7562
Dg(Pgf1Alpha/15:0/0:0)	5.951E-7	1.759E-5	1.8802	0.4929	-1.0206	down	LevelBii	HMDB0295384	637.4986	8.1247
3-Chloro-N-(4-Imidazo[1,2-A]Pyrimidin-2-Ylphenyl)Benzamide	1.325E-7	6.812E-6	1.8788	1.901	0.9268	up	LevelBi	-	349.0883	2.7562
Cefadroxil	8.074E-9	1.045E-6	1.8767	3.0099	1.5897	up	LevelBii	HMDB0015271	427.1058	0.776
Diisodityrosine	0.0006995	0.003078	1.8746	0.4091	-1.2896	down	LevelBii	HMDB0029798	739.2182	3.9904
Sagittariol	3.393E-5	0.0003049	1.8708	0.5749	-0.7986	down	LevelBii	HMDB0036835	339.2884	8.7076
Ouabain	0.0001634	0.0009853	1.8698	0.2401	-2.0583	down	LevelBii	HMDB0015224	549.2719	3.3902
Pinacidil	4.861E-9	7.755E-7	1.8678	4.6854	2.2282	up	LevelBii	HMDB0256556	266.1383	4.0648
4-Methyl-2-Phenylpyridine	1.597E-7	7.724E-6	1.8673	0.5428	-0.8814	down	LevelBi	-	170.096	3.0142
Nifedipine	2.118E-6	4.172E-5	1.8666	0.3964	-1.335	down	LevelBi	HMDB0015247;PW_C009534	347.1219	2.905
Ser-Tyr	0.004853	0.01201	1.8651	3.036	1.6022	up	LevelBi	HMDB0258243	269.1109	2.0414
Raticate	3.919E-6	6.432E-5	1.8627	3.4105	1.77	up	LevelBii	HMDB0257112	546.1624	2.7555
N-Monodemethyl Roxithromycin	1.315E-6	3.031E-5	1.8606	2.602	1.3796	up	LevelBii	HMDB0060845	791.4929	7.6677
Kudzusaponin Sa2	3.704E-5	0.0003264	1.8592	0.3915	-1.3529	down	LevelBii	HMDB0041241	979.472	5.2227
13,14-Dihydro Pgf-1A	0.0002536	0.001389	1.8587	0.3021	-1.727	down	LevelBii	HMDB0005076	357.2632	6.6837
Kaempferol 3-O-Alpha-L-Rhamnofuranoside	0.0001177	0.0007707	1.8578	0.238	-2.0712	down	LevelBii	HMDB0040542	431.0953	4.5003
Ethyl Oleate	0.002172	0.007466	1.8535	0.597	-0.7441	down	LevelBi	HMDB0034451;LMFA07010880	311.293	8.7553
3-O-Feruloylquinic Acid	7.343E-8	4.427E-6	1.8524	3.8412	1.9416	up	LevelBi	HMDB0030669	367.1016	3.196
Lauric Acid	5.934E-6	8.607E-5	1.8501	0.2617	-1.9339	down	LevelBi	HMDB0000638;LMFA01010012	201.1841	8.0084
Zapoterin	8.379E-7	2.192E-5	1.8443	133.7643	7.0635	up	LevelBii	HMDB0035984	505.1668	1.9592
(9Z)-5,8,11-Trihydroxyoctadec-9-Enoic Acid	5.117E-6	7.711E-5	1.844	0.3737	-1.4202	down	LevelBi	-	331.246	5.6542
Ethyl Acrylate	0.0001972	0.001139	1.8392	0.3533	-1.5012	down	LevelBi	HMDB0033978	101.0593	2.2706
Trans-O-Coumaric Acid 2-Glucoside	3.24E-7	1.199E-5	1.8381	1.7908	0.8406	up	LevelBi	HMDB0033581	325.0917	2.7555
7,8-Epoxydihydrogrindelic Acid	6.426E-6	9.127E-5	1.8373	0.102	-3.2927	down	LevelBi	-	337.236	5.1952
Thr-Arg-Lys-Glu	0.001381	0.005228	1.8364	0.1399	-2.8375	down	LevelBi	-	531.2957	5.9789

Leukotriene D4	0.001231	0.004781	1.8351	0.5028	-0.9918	down	LevelBii	HMDB0003080	541.2626	5.2412
Pc(Pge2/P-16:0)	0.0184	0.0367	1.8304	2.8971	1.5346	up	LevelBii	HMDB0289450	796.5419	9.4026
Xanthosine	1.776E-7	8.123E-6	1.8301	3.8972	1.9625	up	LevelBi	HMDB0000299;PW_C000203	283.0674	2.101
Leustroducsin B	0.00026	0.001415	1.8296	2.2929	1.1972	up	LevelBii	HMDB0254044	668.3609	3.0639
Norcisapride	0.002379	0.007964	1.8283	0.5167	-0.9527	down	LevelBii	HMDB0061018	671.2319	3.9904
Glu-Phe	0.0006009	0.002742	1.8283	2.2946	1.1983	up	LevelBi	HMDB0029156	295.1274	2.4
Met-Gly	1.693E-7	7.997E-6	1.8281	4.0578	2.0207	up	LevelBi	HMDB0028973	207.0785	1.6199
Ethylestrenol	0.0001298	0.0008263	1.826	0.4531	-1.1422	down	LevelBii	HMDB0252076	271.2408	6.4371
beta-Asp-Leu	0.0007122	0.003125	1.8238	2.0667	1.0473	up	LevelBii	HMDB0011166	245.1136	2.2638
Asperulosidic Acid	1.318E-5	0.0001515	1.8237	9.1631	3.1958	up	LevelBii	HMDB0248668	491.1389	2.4817
Mlcl(14:0/14:1/18:3)	0.001129	0.00446	1.8228	0.4067	-1.298	down	LevelBi	-	1123.6365	7.5551
Val-Ile-Arg	0.004189	0.01061	1.8216	0.2771	-1.8515	down	LevelBi	-	387.2688	1.9026
N-Eicosapentaenoyl Tryptophan	0.002479	0.008192	1.821	0.453	-1.1424	down	LevelBii	HMDB0242081	530.3381	8.9906
2-[4-(Dimethylamino)Phenyl]-1,2,3,4-Tetrahydroquinazolin-4-One	4.615E-5	0.0003878	1.82	0.304	-1.7178	down	LevelBi	-	268.144	5.1367
Chenodeoxycholylaspartic Acid	0.0002595	0.001413	1.8168	0.1376	-2.8616	down	LevelBii	HMDB0242402	552.3135	2.3969
3-Butyn-1-Al	0.00349	0.00913	1.8155	0.3789	-1.4	down	LevelBii	HMDB0006853	69.0334	0.6887
11'-Carboxy-Gamma-Chromanol	2.935E-7	1.121E-5	1.8129	0.2305	-2.1172	down	LevelBii	HMDB0012517	403.2816	9.2981
1-Cyclohexylcyclohexanecarboxylic Acid	0.005423	0.01319	1.81	0.3909	-1.3553	down	LevelBi	-	211.1687	7.7542
Lnape(20:5/N-3:0)	0.001492	0.005568	1.8097	7.2568	2.8593	up	LevelBi	-	614.3054	7.3871
3,3-Dimethylacrylic Acid	1.111E-5	0.0001337	1.8072	0.527	-0.9242	down	LevelBi	HMDB0000509	101.0588	0.7273
Asp-Gly-Leu	2.156E-5	0.0002147	1.8037	0.436	-1.1974	down	LevelBi	-	304.1478	2.5069
Arg-Met-His	0.0001726	0.001027	1.8009	0.3703	-1.4331	down	LevelBi	-	460.2478	1.1086
Eugenosedin-A	5.288E-9	8.147E-7	1.7986	20.2734	4.3415	up	LevelBii	HMDB0252125	475.2026	1.8648
6-Carboxy-5,6,7,8-Tetrahydropterin	4.084E-7	1.4E-5	1.7919	0.5472	-0.8699	down	LevelBii	HMDB0060410	246.0396	2.9046
Acemetacin	0.0004082	0.002036	1.7917	3.6897	1.8835	up	LevelBii	HMDB0247898	460.0804	1.7993
Met-Leu-Phe	2.129E-5	0.0002136	1.791	0.3221	-1.6345	down	LevelBii	HMDB0254492	410.2085	3.7746
Dehydroxynocardamine	1.731E-6	3.655E-5	1.791	0.5024	-0.9931	down	LevelBi	-	585.357	2.1616
Pi(6 Keto-Pgf1Alpha/18:3(6Z,9Z,12Z))	0.006047	0.01445	1.7905	0.3452	-1.5347	down	LevelBii	HMDB0276784	981.466	6.6929
Metoprolol	1.967E-5	0.0002031	1.7903	3.767	1.9134	up	LevelBii	HMDB0001932	312.1804	3.045

12,13-Dihydroxy-9Z-Octadecenoic Acid	6.325E-5	0.0004857	1.789	0.542	-0.8836	down	LevelBi	LMFA02000230	315.2516	7.0116
Mimosine	0.0001447	0.0008978	1.7872	2.3255	1.2176	up	LevelBii	HMDB0015188	243.0609	1.8648
Vaccenic Acid	0.001241	0.004813	1.782	0.6612	-0.5969	down	LevelBi	HMDB0003231;HMDB0244344	283.2625	8.115
Benzodioxole Fentanyl	2.984E-10	1.158E-7	1.7817	0.0346	-4.8545	down	LevelBi	-	429.2157	3.1032
Kalkitoxin	0.002642	0.008592	1.7812	0.1375	-2.8622	down	LevelBi	-	367.2795	7.8804
Trans-3-Feruloylcorosolic Acid	0.0002421	0.001337	1.781	2.3558	1.2362	up	LevelBii	HMDB0040496	666.438	8.3297
Wikstromol	1.884E-6	3.849E-5	1.7796	0.2072	-2.2706	down	LevelBii	HMDB0030598	355.116	4.2596
Tomatine	0.0003505	0.001804	1.7765	0.4755	-1.0726	down	LevelBii	HMDB0034103	1092.5546	5.0105
Cdp-Dg(6 Keto-Pgf1Alpha/18:2(9Z,11Z))	0.0004261	0.002097	1.7757	0.3752	-1.4143	down	LevelBii	HMDB0291312	1074.5159	3.0238
1-(2,6-Dihydroxy-4-Methoxyphenyl)-3-Phenyl-1-Propanone	0.0002057	0.001177	1.7691	0.3828	-1.3853	down	LevelBii	HMDB0034435	331.1169	4.0648
(2R)-2-[[(2R)-2,6-Diaminohexanoyl]Amino]-3-Phenylpropanoic Acid	2.477E-6	4.642E-5	1.7688	7.8798	2.9782	up	LevelBi	-	316.1608	1.6103
(2R)-1-(2-Hydroxyethyl)-2,5,5,8A-Tetramethyldecahydronaphthalen-2-Ol	0.0003174	0.001666	1.7682	0.5459	-0.8732	down	LevelBi	-	277.2154	6.641
6-Tert-Butyl-1,2,3,4-Tetrahydroquinoline	0.00202	0.007058	1.7655	2.3932	1.2589	up	LevelBi	-	244.1687	3.3505
Tobramycin	1.444E-5	0.0001616	1.7624	0.2783	-1.8454	down	LevelBii	HMDB0014822	448.2445	3.5833
Temsirolimus	2.617E-5	0.0002492	1.7599	2.5065	1.3256	up	LevelBii	HMDB0015632	1047.6327	7.7929
Estradiol Valerate	5.618E-5	0.0004458	1.7597	0.3828	-1.3852	down	LevelBii	HMDB0251966	391.2062	2.2829
Torvonin A	0.001882	0.00667	1.7595	0.4569	-1.1302	down	LevelBii	HMDB0030732	759.414	6.4339
Kinetensin 1-3	0.000283	0.00152	1.7571	0.1936	-2.3687	down	LevelBii	HMDB0012983	379.2081	6.0528
Ps(O-10:0/16:1)	3.931E-6	6.444E-5	1.7565	1.9569	0.9686	up	LevelBi	-	694.4291	8.4733
(1-(3-Oxo-2-Aza-Spiro(4.5)Dec-2-Ylmethyl)-Cyclohexyl)-Acetic Acid	4.758E-5	0.000397	1.7564	0.3459	-1.5315	down	LevelBi	-	308.2195	4.6876
Uridine	7.685E-7	2.082E-5	1.7548	2.357	1.237	up	LevelBi	HMDB0000296;PW_C000202	243.0603	1.1746
Trans-3'-Hydroxycotinine O-Beta-D-Glucuronide	2.951E-6	5.272E-5	1.7508	0.4879	-1.0355	down	LevelBii	HMDB0341403	427.1347	4.0277
Calenduloside H Methyl Ester	0.0009368	0.003867	1.7498	0.2526	-1.9853	down	LevelBii	HMDB0041021	1005.4864	7.2568
16-Acetylpriverogenin A	0.000745	0.003231	1.7488	0.4599	-1.1207	down	LevelBii	HMDB0034529	514.3633	9.1283
Pgp(Txb2/I-18:0)	0.0001498	0.000923	1.7484	0.3577	-1.4833	down	LevelBii	HMDB0275447	979.4694	5.6646
Desacetyl-Alacepril	0.001822	0.006512	1.7467	6.6765	2.7391	up	LevelBii	HMDB0251027	385.1206	5.0198
Dilinoleoylphosphatidylcholine	0.01934	0.0382	1.7452	1.8361	0.8767	up	LevelBii	HMDB0244099	826.5586	7.9686
Toluene	5.326E-7	1.654E-5	1.7443	0.5261	-0.9265	down	LevelBii	HMDB0034168	93.0693	1.8633
Tyr-Met	0.003048	0.008669	1.7439	1.8975	0.9241	up	LevelBii	HMDB0029111	293.0952	4.0091

Ile-Val-Asp	1.555E-5	0.0001702	1.743	0.3851	-1.3769	down	LevelBi	-	346.1965	5.029
Melleolide	0.001015	0.004115	1.7407	0.5559	-0.847	down	LevelBii	HMDB0035689	399.1847	8.1252
Pa(Pgf2Alpha/2:0)	0.0004372	0.002136	1.7402	6.677	2.7392	up	LevelBii	HMDB0266516	568.2861	3.3308
Coumarin	0.002191	0.007517	1.7362	2.1704	1.118	up	LevelBi	HMDB0001218	147.0427	1.8436
Fahfa(22:4/22:3)	0.0008261	0.003502	1.7358	0.4937	-1.0182	down	LevelBi	-	663.5327	8.9824
Mg(18:1(9Z)/0:0/0:0)	0.00462	0.01153	1.734	0.5223	-0.9371	down	LevelBi	HMDB0011567	357.2987	8.7745
(2-Anilino-4-Methyl-1,3-Thiazol-5-Yl)(1-Benzothiophen-2-Yl)Methanone	2.027E-7	8.925E-6	1.7293	0.2562	-1.9647	down	LevelBi	-	389.0175	0.5895
Trp-Ser	4.389E-6	6.927E-5	1.7273	0.2586	-1.9511	down	LevelBii	HMDB0029092	336.1183	2.6326
Pyrano(2,3-C)Pyrazol-6(2H)-One, 3,4-Dimethyl-2-(2-Thienylmethyl)-	2.409E-5	0.0002328	1.7272	0.4303	-1.2167	down	LevelBii	HMDB0253035	319.0753	1.156
17-Debromo-Aplysiatoxin,	0.0003439	0.001778	1.725	3.2171	1.6858	up	LevelBii	HMDB0248517	627.2984	2.4348
Apc	0.0006845	0.003037	1.7235	0.3748	-1.4158	down	LevelBii	HMDB0060661	677.2796	4.2873
Phosphorylcholine	3.377E-7	1.234E-5	1.7233	0.181	-2.4657	down	LevelBii	HMDB0001565	165.0544	4.4909
N-Carbobenzoxyglycyl-Prolyl-4-Methylcoumarinyl Amide	0.0001272	0.0008152	1.7199	2.0347	1.0248	up	LevelBii	HMDB0259966	522.1848	2.1593
Pe(Pgf2Alpha/P-18:1(11Z))	1.8E-5	0.000189	1.7194	0.4206	-1.2494	down	LevelBii	HMDB0285409	800.5378	7.7251
(2S)-2-[[(2R)-2-Amino-3-Carboxypropanoyl]Amino]-4-Methylpentanoic Acid	1.237E-5	0.0001442	1.7191	1.5665	0.6476	up	LevelBi	-	247.1287	2.2706
Mg(5-Iso Pgf2Vi/0:0/0:0)	0.003492	0.009132	1.7182	0.3081	-1.6983	down	LevelBii	HMDB0260485	421.2186	5.4251
Trp-Asp-Asp	1.106E-5	0.0001335	1.7145	0.4908	-1.0268	down	LevelBi	-	435.1488	2.2607
S-Adenosyl-4-Methylthio-2-Oxobutanoate	5.39E-7	1.654E-5	1.7143	1.9095	0.9332	up	LevelBii	HMDB0304475	431.1465	2.786
Floionolic Acid	0.000263	0.001428	1.714	0.3545	-1.4963	down	LevelBii	HMDB0034295	353.2311	6.9054
N-(1-Deoxy-1-Fructosyl)Leucine	0.0003499	0.001801	1.7137	2.558	1.355	up	LevelBii	HMDB0037840	292.1396	1.1283
Paromomycin I	0.004557	0.0114	1.7125	0.1547	-2.6923	down	LevelBii	HMDB0242512	633.3348	4.5797
2-{[2-(1H-Indol-3-Yl)Ethyl]Ethanimidoyl}Phenol	0.0009444	0.003894	1.7109	13.4031	3.7445	up	LevelBi	-	333.1545	2.4099
Pi(13:0/16:4)	0.0006051	0.002758	1.7097	0.662	-0.595	down	LevelBi	-	805.4202	6.4339
Naratriptan	9.584E-8	5.335E-6	1.7095	3.1516	1.6561	up	LevelBii	HMDB0015087	336.1732	3.7746
Flupirtine	1.169E-5	0.000139	1.7082	3.7443	1.9047	up	LevelBii	HMDB0252403	339.1002	3.5738
Bisphenol S	0.003526	0.009209	1.7071	13.2417	3.727	up	LevelBi	HMDB0240712	251.0361	4.2271
Blumenol C Glucoside	4.091E-6	6.608E-5	1.7067	2.0292	1.0209	up	LevelBii	HMDB0040668	417.2111	4.2132
2Beta-Hydroxytestosterone	0.002817	0.008669	1.7062	0.2479	-2.0124	down	LevelBii	HMDB0012654	349.1992	5.5908
Glu-Ile	0.0001074	0.0007198	1.7055	2.037	1.0264	up	LevelBi	HMDB0028822	283.1253	2.2211

2-Hydroxy-4-(4-Methoxyphenyl)-6-Phenylnicotino nitrile	2.841 E-6	5.105 E-5	1.7054	0.563 2	-0.8284	dow n	Leve lBi	-	320.1 424	2.6865
Dihomo-Gamma-Linolenoylethanolamide	0.001 815	0.006 494	1.7051	0.506 2	-0.9823	dow n	Leve lBii	HMDB0013625	350.3 025	8.9808
1,4-Dihydro-3(2H)-Isoquinolinone	1.326 E-5	0.000 1522	1.7042	2.353 9	1.2351	up	Leve lBi	-	130.0 642	2.4
Paxilline	0.001 354	0.005 142	1.7029	0.461 7	-1.115	dow n	Leve lBi	HMDB0030323	458.2 331	1.6199
Benzoic Acid	0.000 1278	0.000 8176	1.7022	1.810 9	0.8567	up	Leve lBi	HMDB0001870	123.0 432	1.8436
Kanamycin	0.001 422	0.005 351	1.7019	0.616 8	-0.6972	dow n	Leve lBii	HMDB0015303	967.4 702	5.7845
3-O-Methylkaempferol	0.002 061	0.007 167	1.7008	0.205 1	-2.2859	dow n	Leve lBii	HMDB0302564	299.0 545	5.1673
Xanthoxylin	0.001 097	0.004 364	1.7006	3.654 2	1.8696	up	Leve lBi	HMDB0029645	197.0 801	5.664
Mg(Pgf2Alpha/0:0/0:0)	0.001 743	0.006 287	1.6981	0.461 2	-1.1165	dow n	Leve lBii	HMDB0260478	433.2 546	3.0833
Styrene	1.355 E-7	6.858 E-6	1.6979	0.671 9	-0.5737	dow n	Leve lBi	HMDB0034240	105.0 693	2.1616
Leu-Phe	3.524 E-6	5.995 E-5	1.6969	0.473 2	-1.0796	dow n	Leve lBi	HMDB0302841	261.1 585	4.8536
Spinacetin 3-Gentiobioside	5.462 E-6	8.087 E-5	1.6945	0.619 4	-0.691	dow n	Leve lBii	HMDB0037469	729.1 887	3.7495
Tangeraxanthin	0.000 3318	0.001 726	1.6938	6.913 5	2.7894	up	Leve lBii	HMDB0039015	505.3 056	8.3635
Lys-Asp-Tyr	0.002 324	0.007 832	1.6931	0.236 3	-2.0816	dow n	Leve lBii	HMDB0304806	459.1 635	6.1478
8-Methyl-2-(Trifluoromethyl)Quinolin-4-Ol	0.000 1114	0.000 7413	1.6898	0.516 8	-0.9524	dow n	Leve lBi	-	228.0 614	3.8723
Arg-Ile	1.884 E-7	8.507 E-6	1.6898	2.871	1.5216	up	Leve lBii	HMDB0028712	286.1 876	1.8648
N-Cyclooctyl-1-(4-Fluorobenzoyl)Piperidine-4-Car boxamide	1.633 E-5	0.000 1766	1.6881	0.498 3	-1.0048	dow n	Leve lBi	-	361.2 309	5.5077
2-Amino-3-[5-Tert-Butyl-3-(Phosphonomethoxy)Is oxazol-4-Yl]Propionic Acid	0.001 151	0.004 527	1.6852	2.785 2	1.4778	up	Leve lBi	-	323.1 035	4.7071
Hydroxypioglitazone	7.306 E-5	0.000 5375	1.683	3.723 1	1.8965	up	Leve lBii	HMDB0253285	407.0 872	3.3483
Lippioside Ii	1.479 E-7	7.394 E-6	1.6825	0.469 6	-1.0904	dow n	Leve lBii	HMDB0034268	589.1 278	2.5476
N-Cyclopropyl-2-Quinolinecarboxamide	4.807 E-7	1.544 E-5	1.6805	0.468 8	-1.0929	dow n	Leve lBi	-	245.1 278	3.0833
2-Hydroxypropyl Stearate	0.036 33	0.061 03	1.6788	0.162 1	-2.6254	dow n	Leve lBi	HMDB0029764	343.3 186	8.9216
Lnape(13:1/N-7:0)	0.001 875	0.006 653	1.6788	2.052 4	1.0373	up	Leve lBi	-	580.3 235	6.4803
Cdp-Dg(Lte4/I-14:0)	0.002 527	0.008 307	1.671	0.460 4	-1.1191	dow n	Leve lBii	HMDB0293501	1167. 4993	5.6368
Tramadol	0.002 51	0.008 275	1.6684	1.746 1	0.8041	up	Leve lBi	HMDB0014339;HMDB0259401;P W_C008740	264.1 95	6.2803
Flavone Base+3O,C-Hex-Feruloylhex	0.012 97	0.027 39	1.6661	0.54	-0.8891	dow n	Leve lBi	-	771.2 079	4.1388
N-Eicosapentaenoyl Aspartic Acid	0.001 529	0.005 675	1.6648	0.468	-1.0954	dow n	Leve lBii	HMDB0242067	438.2 292	5.4712
4-(3-Aminophenyl)-2-Methyl-3-Butyn-2-Ol	1.525 E-5	0.000 1679	1.6648	0.643 7	-0.6356	dow n	Leve lBi	-	158.0 959	3.0042
Phe-Glu	0.003 721	0.009 593	1.6634	1.870 9	0.9037	up	Leve lBi	HMDB0028994	295.1 273	2.1512

Histamine	1.261E-7	6.554E-6	1.662	0.6177	-0.695	down	LevelBi	HMDB0000870;PW_C000693	112.0865	0.6298
Dg(Pge2/A-15:0/0:0)	0.01119	0.02422	1.6611	0.0801	-3.6419	down	LevelBii	HMDB0297528	649.4638	7.0069
Ketoleucine	0.0002482	0.001364	1.6595	0.3514	-1.5088	down	LevelBi	HMDB0000695;PW_C000547	130.0643	2.9943
Vomifoliol	0.0005445	0.002531	1.6589	2.5919	1.374	up	LevelBii	HMDB0303570	259.1085	4.0277
Vulgaxanthin I	1.026E-9	2.631E-7	1.6581	2.427	1.2792	up	LevelBii	HMDB0029840	723.2118	2.3969
Cassiaside B	5.399E-6	8.018E-5	1.6566	1.5814	0.6612	up	LevelBii	HMDB0038477	547.151	2.101
1,2,3,4-Tetrahydroisoquinoline-3-Carboxylic Acid	6.707E-6	9.418E-5	1.6532	0.6038	-0.7279	down	LevelBii	HMDB0244117	236.0917	3.7311
Jwh 073 2-Hydroxyindole Metabolite-D7	0.000196	0.001135	1.651	0.4872	-1.0375	down	LevelBi	-	349.1976	6.2954
2,4,6-Phenanthrenetriol 2-O-B-D-Glucoside	1.503E-6	3.328E-5	1.6499	1.8791	0.91	up	LevelBii	HMDB0036335	387.1121	0.7365
Magnolol	0.003732	0.009613	1.6492	0.3687	-1.4393	down	LevelBi	HMDB0254305	265.1217	7.0719
Ala-Gly-Leu-Val-Ser	0.000412	0.002048	1.6472	3.4525	1.7877	up	LevelBi	-	444.2446	3.273
Genistein 4'-O-Glucoside	0.005908	0.01417	1.6463	0.4497	-1.153	down	LevelBi	HMDB0252680;LMPK12050168	431.0955	4.139
N-Linoleoyl Gaba	0.008579	0.01935	1.6444	2.8191	1.4952	up	LevelBii	HMDB0062334	386.2712	8.9262
Gailiapenta	9.158E-8	5.138E-6	1.6382	1.9878	0.9912	up	LevelBii	HMDB0252572	850.2825	1.9687
Flavone Base+3O,2Meo,O-Guaiacylglyceryl-Hex	3.182E-6	5.547E-5	1.6375	1.928	0.9471	up	LevelBi	-	689.2056	0.7565
Formyl-5-Hydroxykynurenamine	0.001244	0.004818	1.6373	3.8678	1.9515	up	LevelBii	HMDB0012948	267.0971	1.5948
Valorphin	2.347E-5	0.0002284	1.6356	3.384	1.7587	up	LevelBii	HMDB0059789	927.4112	2.7461
Diethyl 6-Methoxy-5,7-Dihydroindolo[2,3-B]Carbazole-2,10-Dicarboxylate	7.399E-5	0.0005421	1.6323	3.1861	1.6718	up	LevelBii	HMDB0258457	451.1293	2.2544
Nandrolone	0.0002561	0.001399	1.6311	0.4708	-1.0868	down	LevelBi	HMDB0002725;LMST02010044	275.1995	6.2901
Val-Cys	0.01119	0.02422	1.6286	2.2004	1.1378	up	LevelBi	HMDB0029124	221.094	1.6493
N-Acetyl-L-Tyrosine	5.401E-7	1.654E-5	1.6277	0.4874	-1.0369	down	LevelBii	HMDB0000866	188.0687	2.8754
Ile-Lys-Tyr	2.903E-5	0.0002681	1.6261	0.6765	-0.5639	down	LevelBi	-	423.2575	2.3204
Tyr-Val-Phe	3.164E-5	0.0002878	1.6255	0.3004	-1.735	down	LevelBi	-	428.215	3.4783
Stearidonic Acid Ethyl Ester	0.001312	0.00502	1.6247	0.5412	-0.8858	down	LevelBi	-	305.2458	7.6196
Lpc(18:3)	0.0008075	0.003436	1.6232	1.5331	0.6165	up	LevelBi	HMDB0010387	562.313	7.294
Menthyl Pyrrolidone Carboxylate	0.0007195	0.00315	1.6213	0.3525	-1.5041	down	LevelBii	HMDB0032368	299.1849	5.6184
Hydroxygaleon	2.683E-5	0.0002525	1.6205	0.5888	-0.7641	down	LevelBii	HMDB0031535	343.152	4.0206
Paramethasone Acetate	2.605E-6	4.779E-5	1.6184	1.7591	0.8148	up	LevelBii	HMDB0256122	433.2046	3.0639

Americine	0.011 94	0.025 6	1.6154	1.982 8	0.9875	up	Leve lBii	HMDB0034441	528.2 99	2.4582
Pro-Gln	0.000 3951	0.001 986	1.615	2.660 7	1.4118	up	Leve lBii	HMDB0029015	288.1 186	1.846
Deacetoxy(7)-7-Oxokhivorinic Acid	5.711 E-5	0.000 4492	1.615	2.247	1.168	up	Leve lBii	HMDB0341151	541.2 104	2.2735
9-Hydroxy-3,4-Dihydroacridin-1(2H)-One	0.012 73	0.026 99	1.6097	0.411 6	-1.2808	dow n	Leve lBi	-	214.0 848	3.1428
14-Hdohe	7.943 E-7	2.123 E-5	1.6089	0.478 7	-1.0627	dow n	Leve lBii	HMDB0060044	391.2 059	2.4066
Pyroglutamylleucine	0.008 998	0.020 14	1.605	2.326 3	1.218	up	Leve lBii	HMDB0341382	241.1 185	2.2165
1,2-Ethanediol Monoricinoleate	0.005 069	0.012 45	1.6021	0.568	-0.816	dow n	Leve lBii	HMDB0252073	365.2 64	7.7929
4-Phenyl-1,2,3-Thiadiazole	5.042 E-5	0.000 4131	1.5995	1.898 5	0.9249	up	Leve lBi	-	204.0 604	0.7955
[4-(4-Nitrophenyl)Piperazin-1-Yl](4-Propoxypheny l)Methanone	2.532 E-5	0.000 2429	1.5993	0.213 2	-2.23	dow n	Leve lBi	-	370.1 748	3.2222
2-Pyrrolidinone	4.255 E-8	3.055 E-6	1.5973	0.612	-0.7084	dow n	Leve lBi	HMDB0002039	86.05 98	0.6887
Silodosin	0.000 1796	0.001 062	1.5972	0.318 5	-1.6507	dow n	Leve lBii	HMDB0258291	516.2 137	3.7495
1-[2-[1-(Benzenesulfonyl)Piperidin-4-Yl]Ethyl]-3-P ropan-2-Ylurea	2.221 E-6	4.282 E-5	1.5967	1.650 6	0.723	up	Leve lBi	-	354.1 872	2.3104
Pyropheophorbide A	0.001 479	0.005 53	1.592	0.505 3	-0.9848	dow n	Leve lBii	HMDB0256993	1113. 5288	5.8032
Dg(Lte4/I-12:0/0:0)	0.000 132	0.000 8368	1.5918	0.601	-0.7346	dow n	Leve lBii	HMDB0298379	695.4 447	5.8592
10-Hydroxy-3-Methoxy-1,3,5,7-Cadinatetraen-9-O ne	1.831 E-6	3.756 E-5	1.5901	2.210 4	1.1443	up	Leve lBii	HMDB0036456	565.2 841	8.6598
Ile-Glu-Thr-Asp-Fluoromethyl Ketone	0.000 6925	0.003 058	1.5882	0.435 9	-1.1979	dow n	Leve lBii	HMDB0253396	473.2 08	2.8956
Carmine Red	0.000 6362	0.002 867	1.5857	0.529 4	-0.9176	dow n	Leve lBii	HMDB0030658	513.0 674	4.2781
Pe(O-20:5/18:0)	0.004 777	0.011 86	1.5852	2.191	1.1316	up	Leve lBi	-	810.5 637	8.6411
Pinolidoxin	1.158 E-5	0.000 1381	1.5849	0.667 8	-0.5826	dow n	Leve lBii	HMDB0301836	359.1 483	4.0277
Verbasoside	7.331 E-6	0.000 1007	1.5827	0.469 3	-1.0913	dow n	Leve lBii	HMDB0039233	463.1 855	3.6068
Pro-Ser	0.000 3377	0.001 75	1.5812	0.485 7	-1.0419	dow n	Leve lBi	HMDB0029026	203.1 014	0.7273
Milbemycin Beta3	0.000 45	0.002 183	1.5804	0.280 7	-1.833	dow n	Leve lBii	HMDB0254729	515.2 805	2.3208
2-Amino-1-Phenylethanol	0.000 8696	0.003 649	1.5803	2.182 3	1.1258	up	Leve lBi	HMDB0001065	120.0 797	1.6103
Smgdg(O-13:1/7:0)	0.005 381	0.013 1	1.5802	0.447 3	-1.1606	dow n	Leve lBi	-	671.3 259	6.9054
3,4-Dihydroxyhydrocinnamic Acid	4.051 E-5	0.000 3503	1.5793	2.376 3	1.2487	up	Leve lBii	HMDB0000423	163.0 389	1.846
Pgp(Lte4/I-16:0)	0.000 3592	0.001 839	1.5786	0.528 8	-0.9193	dow n	Leve lBii	HMDB0275235	984.4 618	5.7845
4-Acetyloxy-8-(3-Oxo-2-Pent-2-Enylcyclopenten-1 -Yl)Octanoic Acid	0.000 2768	0.001 492	1.5771	0.426 4	-1.2297	dow n	Leve lBi	-	349.1 98	6.0435
Miglitol	1.061 E-6	2.573 E-5	1.577	0.541 9	-0.884	dow n	Leve lBii	HMDB0014634	188.0 919	2.7083
Lentialexin	3.187 E-6	5.547 E-5	1.5767	0.332 7	-1.5877	dow n	Leve lBii	HMDB0038997	121.0 638	5.0781

Benzyl[(3-Methylphenyl)Methyl]Amine	0.0005079	0.002395	1.5764	1.7707	0.8243	up	LevelBi	-	244.1684	3.4295
Gingerglycolipid A	0.001186	0.004631	1.575	0.5292	-0.918	down	LevelBii	HMDB0041093	675.3567	5.4988
Carpinontriol B	0.002507	0.008269	1.5733	0.5395	-0.8904	down	LevelBi	-	345.1304	3.6363
Tragopogonsaponin D	0.01373	0.02874	1.5715	0.5704	-0.8099	down	LevelBii	HMDB0037915	1099.5133	5.6277
Metamizol	1.25E-5	0.0001453	1.5668	0.303	-1.7226	down	LevelBii	HMDB0254502	356.0922	1.5762
Sacubitrilat	5.228E-7	1.647E-5	1.565	0.2367	-2.079	down	LevelBii	HMDB0257440	404.1514	1.688
1-(1-Chloroethyl)-4-Fluorobenzene	4.019E-8	2.945E-6	1.5647	1.4395	0.5256	up	LevelBii	HMDB0243770	217.0452	0.671
Cdp-Dg(Pge2/A-21:0)	0.007056	0.01644	1.5645	2.3863	1.2548	up	LevelBii	HMDB0293076	1100.5692	3.2619
Dodecyl-Beta-D-Maltoside	0.01603	0.03265	1.5603	0.4873	-1.0372	down	LevelBii	HMDB0247203	491.2835	5.2964
Acetophenone	6.548E-6	9.267E-5	1.5584	0.6037	-0.728	down	LevelBi	HMDB0033910	121.064	3.745
Cyclolinopeptide B	0.01897	0.03758	1.558	2.2715	1.1836	up	LevelBii	HMDB0302161	1078.587	3.2619
Isovitexin 2''-O-(6'''-Feruloyl)Glucoside	0.006392	0.01513	1.5578	0.6276	-0.672	down	LevelBii	HMDB0038042	769.1958	4.139
1-Deoxy-1-Morpholino-D-Fructose	0.0008847	0.003701	1.5573	0.3339	-1.5827	down	LevelBii	HMDB0243858	270.0939	3.4894
Lys-Ile-Asn	0.002699	0.008669	1.5568	3.2303	1.6917	up	LevelBi	-	374.2368	1.9026
Palmitoyl Glucuronide	0.03872	0.06435	1.5558	1.9222	0.9428	up	LevelBii	HMDB0010331	399.2736	8.3544
Farnesyl Acetone	0.007378	0.01707	1.5552	0.6715	-0.5745	down	LevelBi	-	263.2363	7.7446
Adenosine	0.0002563	0.0014	1.5535	0.2767	-1.8534	down	LevelBi	HMDB0000050;HMDB0247583;PW_C000034	268.1025	1.9224
Cosmosiin	0.000525	0.002458	1.5517	0.4808	-1.0566	down	LevelBi	HMDB0037340	433.109	4.1388
Val-Ala	0.0002047	0.001173	1.5515	0.4625	-1.1126	down	LevelBii	HMDB0029120	187.1076	0.7365
1-Phenyl-2,4-Pentadiyn-1-One	1.705E-6	3.616E-5	1.5512	4.1269	2.0451	up	LevelBii	HMDB0032945	353.0794	2.9979
Pyroglutamyllysine	7.998E-5	0.0005754	1.5507	0.454	-1.1392	down	LevelBii	HMDB0341383	302.1348	2.5098
9S-Hydroxy-10E,12Z,15Z-Octadecatrienoic Acid	0.0002377	0.001318	1.5504	0.6363	-0.6523	down	LevelBi	LMFA02000024	295.2256	6.641
Ethyl Arachidonate	0.003759	0.009667	1.5485	0.5761	-0.7957	down	LevelBi	HMDB0062725	333.2781	9.207
6-Chloro-2,3,4,9-Tetrahydro-1H-Carbazole-1-Carboxamide	8.439E-6	0.0001101	1.5485	0.7025	-0.5094	down	LevelBii	HMDB0246699	541.143	3.7495
Trp-Val-Asn	0.002639	0.008592	1.5472	0.4862	-1.0405	down	LevelBi	-	418.2073	2.3104
Plantainoside C	3.67E-6	6.148E-5	1.5457	0.5982	-0.7412	down	LevelBi	-	639.2265	3.6068
Rebaudioside A	0.01727	0.03476	1.5457	0.3695	-1.4364	down	LevelBii	HMDB0034950	967.4383	5.9668
Dg(5-Iso Pgf2Vi/18:0/0:0)	0.007055	0.01644	1.5444	0.4921	-1.0229	down	LevelBii	HMDB0295904	701.4745	8.88
Cinnamyl Anthranilate	0.001063	0.004258	1.5424	0.3903	-1.3575	down	LevelBii	HMDB0041319	312.1218	4.4169

Arg-His	4.573E-6	7.121E-5	1.5416	0.6175	-0.6955	down	LevelBii	HMDB0028711	356.1686	3.5552
Coprine	0.002305	0.007799	1.5402	0.3947	-1.3412	down	LevelBii	HMDB0034266	201.0868	0.727
2-Amino-9-[(2R,4S,5R)-5-(Hydroperoxymethyl)-4-Hydroxyoxolan-2-Yl]-1H-Purin-6-One	4.304E-7	1.438E-5	1.5396	1.6532	0.7253	up	LevelBii	HMDB0257980	282.0831	1.9781
Succinylaminoimidazole Carboxamide Riboside	1.783E-5	0.0001877	1.5396	1.6865	0.754	up	LevelBii	HMDB0240295	339.0899	0.7565
N-Acetylaspartate	8.192E-6	0.0001083	1.5376	0.6053	-0.7243	down	LevelBi	HMDB0000812	174.0399	0.7644
1H-Indole-4-Carboxaldehyde	0.001009	0.004098	1.536	1.562	0.6434	up	LevelBi	-	146.0596	2.3901
Ser-Ile	0.001111	0.004409	1.5316	1.5187	0.6028	up	LevelBi	HMDB0029042	219.1332	2.1616
Ala-Met	7.629E-5	0.0005578	1.5309	0.5769	-0.7935	down	LevelBi	HMDB0028693	221.0938	2.0314
N,N-Di-N-Propyldopamine	1.993E-8	1.947E-6	1.5269	1.732	0.7924	up	LevelBii	HMDB0255249	296.1855	3.7766
Difructose Anhydride Iii	1.529E-8	1.614E-6	1.525	0.5703	-0.8101	down	LevelBii	HMDB0251271	345.0781	2.2638
Dgdg(O-18:5/6:0)	0.01524	0.03133	1.5241	0.5173	-0.951	down	LevelBi	-	801.4241	7.3685
Galactitol	1.425E-6	3.212E-5	1.5206	1.6058	0.6833	up	LevelBii	HMDB0000107	181.0708	0.671
Annonacin	0.007585	0.01746	1.5201	0.3996	-1.3232	down	LevelBii	HMDB0033327	617.4354	9.3543
Ethyl Palmitoleate	0.0007927	0.003386	1.5192	0.6459	-0.6307	down	LevelBi	-	283.2614	9.0795
Ala-Arg	6.338E-7	1.829E-5	1.5163	0.6685	-0.5809	down	LevelBii	HMDB0028681	268.1392	1.5719
Loquatifolin A	0.002293	0.007774	1.5146	0.6465	-0.6293	down	LevelBii	HMDB0030434	821.4139	6.0435
7,8-Dihydro-3B,6A-Dihydroxy-Alpha-Ionol 9-[Apiosyl-(1->6)-Glucoside]	1.671E-7	7.997E-6	1.513	1.6122	0.689	up	LevelBii	HMDB0041579	540.2995	2.2706
Dgts(17:2/22:6)	0.00708	0.01649	1.5122	1.6507	0.7231	up	LevelBi	-	798.5604	9.343
Methyl 3-(Benzylamino)-2-Methylpropanoate	3.186E-6	5.547E-5	1.5081	0.4957	-1.0124	down	LevelBi	-	262.1424	5.5272
Gingerenone A	0.003702	0.00956	1.5081	0.3288	-1.6048	down	LevelBii	HMDB0035403	391.1317	2.7839
Ethyl L-Tryptophanate	7.89E-5	0.0005697	1.5053	0.4467	-1.1627	down	LevelBi	HMDB0252047	233.1269	3.3505
Agavasaponin C	0.008258	0.01873	1.5018	0.5879	-0.7663	down	LevelBii	HMDB0033353	917.4671	6.9051
Casomorphin	1.351E-8	1.515E-6	1.5017	0.326	-1.6168	down	LevelBii	HMDB0059787	828.4309	3.9615
(R)-6'-O-(4-Geranyloxy-2-Hydroxycinnamoyl)-Marmin	0.0009033	0.003766	1.5016	2.2475	1.1683	up	LevelBii	HMDB0039061	635.2958	2.3204
18R-Hepe	5.385E-6	8.005E-5	1.501	0.5455	-0.8743	down	LevelBii	HMDB0012611	319.2248	5.0586
Lpc(20:5)	0.006855	0.01604	1.4975	1.639	0.7128	up	LevelBi	HMDB0010397	586.3126	7.2754
Shexcer(10:0_2O/20:1_O)	0.00397	0.01014	1.4966	1.8423	0.8815	up	LevelBi	-	762.44	2.796
Phe-Val-Trp	0.004645	0.01158	1.4942	0.1558	-2.6819	down	LevelBi	-	451.2305	4.0401
Cucurbitacin S	0.0003959	0.001988	1.4907	0.5377	-0.895	down	LevelBii	HMDB0250583	537.2566	5.9078

Fahfa(18:1/3:0)	2.141E-5	0.0002141	1.4867	0.7283	-0.4575	down	LevelBi	-	353.2678	9.1118
Citrulline	7.383E-5	0.0005412	1.4861	0.637	-0.6507	down	LevelBi	HMDB0000904;PW_C000723	140.081	1.9523
3-O-Trans-Feruloyleuscaphic Acid	0.01165	0.02507	1.4849	4.3993	2.1373	up	LevelBii	HMDB0031837	685.3677	2.6326
Tyr-Asn-Glu	0.0008074	0.003436	1.4842	0.6241	-0.6801	down	LevelBi	-	425.1641	2.0116
Protoveratrine	7.092E-6	9.793E-5	1.4821	3.1183	1.6408	up	LevelBii	HMDB0256876	838.4291	3.703
Physangulide	0.0005866	0.002686	1.482	2.271	1.1833	up	LevelBii	HMDB0037381	543.2534	6.4896
Glu-Tyr	2.568E-5	0.0002452	1.4814	1.7146	0.7778	up	LevelBii	HMDB0028831	309.1104	2.1202
6-Methyl-1-(3-Sulfanylpropanoyl)-2-Piperidinecarboxylic Acid	0.0009446	0.003894	1.4798	1.3863	0.4712	up	LevelBi	-	295.11	3.991
6-(2-Hydroxyethoxy)-6-Oxohexanoic Acid	2.554E-5	0.0002447	1.4769	0.5838	-0.7766	down	LevelBii	HMDB0061681	254.0984	3.38
Vinblastine	0.003688	0.009532	1.4738	0.4949	-1.0148	down	LevelBii	HMDB0014710	845.3841	5.0198
Trp-Ile-Asp	0.005795	0.01395	1.4696	0.2266	-2.142	down	LevelBi	-	433.2071	3.1428
O-Oxalylhomoserine	0.0002322	0.001295	1.4692	0.4965	-1.0102	down	LevelBii	HMDB0302143	250.0563	1.9309
Isocitrate	1.012E-6	2.485E-5	1.4655	1.3475	0.4302	up	LevelBi	HMDB0000193;HMDB0304324	191.0189	1.4081
4-Deoxytetronic Acid	0.000145	0.0008982	1.465	0.6891	-0.5373	down	LevelBi	HMDB0000549	87.0436	0.6887
Isocolumbin	0.0001591	0.0009672	1.4611	0.5188	-0.9468	down	LevelBii	HMDB0036837	339.1213	4.1855
Tryptophol	0.002086	0.00724	1.4609	1.991	0.9935	up	LevelBii	HMDB0003447	142.0654	2.3969
6-O-(7-Methyloctanoyl)-Alpha-D-Glucopyranosyl Alpha-D-Glucopyranoside	0.02891	0.05308	1.4598	0.1026	-3.2848	down	LevelBi	-	483.2471	4.1978
Thr-Ala-Phe	0.01609	0.03273	1.4578	0.4651	-1.1044	down	LevelBi	-	338.1684	2.5772
Phe-Asp	0.01869	0.03712	1.4562	1.8182	0.8625	up	LevelBi	HMDB0028991	281.1135	2.1311
Lomefloxacin	4.734E-6	7.27E-5	1.4559	2.306	1.2054	up	LevelBi	HMDB0015113	369.1758	1.0985
2-Phenylacetamide	4.701E-5	0.0003937	1.4556	1.4212	0.5071	up	LevelBi	HMDB0010715	136.075	1.8436
3-Hydroxybutyrylcarnitine	7.759E-7	2.097E-5	1.4535	1.3734	0.4578	up	LevelBi	HMDB0013127;LMFA07070037	248.1485	1.5244
Methyl 3-Hydroxy-2-[(3-Hydroxybenzoyl)Amino]Propanoate	0.0005039	0.00238	1.4512	2.2857	1.1926	up	LevelBi	-	257.1119	2.0414
Linoleic Acid	0.006639	0.01563	1.4504	0.7242	-0.4656	down	LevelBi	HMDB0000673;LMFA01030120;PW_C000530	281.2469	7.7446
Pa(5-Iso Pgf2Vi/18:3(6Z,9Z,12Z))	0.003219	0.008669	1.4485	0.5055	-0.9842	down	LevelBii	HMDB0264142	785.4291	7.8848
3-Ethenylphenol	4.727E-8	3.295E-6	1.444	0.7976	-0.3262	down	LevelBi	-	121.0646	1.8633
4-O-Methylascochlorin	0.001629	0.005951	1.4439	0.4634	-1.1098	down	LevelBii	HMDB0246555	417.1842	6.3045
Spirolide E	0.01636	0.0332	1.4426	0.4872	-1.0375	down	LevelBii	HMDB0030494	760.4606	8.5851

Nordihydroguaiaretic Acid	8.359E-5	0.0005919	1.4381	1.5524	0.6345	up	LevelBi	HMDB0014325	341.1146	3.3603
Pi(Pgf2Alpha/22:2(13Z,16Z))	0.000614	0.002787	1.4373	1.4998	0.5848	up	LevelBii	HMDB0277722	1043.6008	7.6962
Thromboxane B2	4.874E-6	7.43E-5	1.436	0.68	-0.5564	down	LevelBi	HMDB0003252;PW_C001820	393.2273	2.2904
Dehydroisoandrosterone 3-Glucuronide	0.02297	0.04394	1.4359	0.5686	-0.8144	down	LevelBii	HMDB0010327	497.2706	4.8829
9,10-Dihome	0.001156	0.004541	1.4354	0.738	-0.4383	down	LevelBi	LMFA02000229	313.2372	7.0162
Hexcer(19:0_3O/12:0_(2Oh))	0.003556	0.009269	1.435	1.9568	0.9685	up	LevelBi	-	750.5322	8.8332
Methylpyrazine	1.581E-6	3.434E-5	1.4318	0.6811	-0.5541	down	LevelBii	HMDB0033112	95.06	0.6298
Leu-Pro-Val-Leu-Glu	0.002645	0.008599	1.4303	0.2805	-1.8338	down	LevelBi	-	568.3365	9.5504
8-Hydroxy-Deoxyguanosine	0.0001118	0.0007427	1.43	1.6847	0.7525	up	LevelBii	HMDB0003333	316.1276	2.7064
Dg(Pgf1Alpha/17:0/0:0)	0.01961	0.03863	1.4294	0.5875	-0.7674	down	LevelBii	HMDB0295800	683.5392	8.6308
Mangostanol	2.168E-6	4.225E-5	1.4286	0.5613	-0.8332	down	LevelBii	HMDB0029868	461.1379	2.576
5-Hexyldihydro-2(3H)-Furanone	0.001154	0.004533	1.4276	0.6662	-0.586	down	LevelBi	HMDB0037217	171.1369	8.1247
Fruleuile	6.86E-5	0.000515	1.4265	0.6738	-0.5695	down	LevelBi	-	407.2371	2.7762
Pyroglutamylisoleucine	1.496E-5	0.0001655	1.4243	1.4153	0.5011	up	LevelBii	HMDB0341381	241.1185	3.273
Coagulin R 3-Glucoside	2.909E-6	5.215E-5	1.4233	0.4434	-1.1733	down	LevelBii	HMDB0033207	691.3292	2.1105
Glycyl-Histidyl-Arginyl-Proline	0.01767	0.03547	1.4224	0.3017	-1.7286	down	LevelBii	HMDB0252877	486.2234	2.7743
Mgdg(O-22:5/2:0)	0.01798	0.03601	1.4192	0.6191	-0.6917	down	LevelBi	-	595.3806	7.0504
Lpe(24:4)	0.03145	0.05684	1.4189	0.3567	-1.4874	down	LevelBi	-	556.3374	8.5289
Dlcl(14:0/16:4)	0.01743	0.03505	1.4169	0.5744	-0.7998	down	LevelBi	-	885.4197	5.9512
Alpha-Zearalenol	0.01272	0.02696	1.4166	0.56	-0.8366	down	LevelBii	HMDB0041824	362.1941	3.7845
N-[(3A,5B,7A)-3-Hydroxy-24-Oxo-7-(Sulfooxy)Cholan-24-Yl]-Glycine	0.00201	0.007029	1.4164	1.7644	0.8191	up	LevelBii	HMDB0002496	530.2746	2.6069
Cdp-Dg(Pgf2Alpha/18:1(11Z))	0.006649	0.01565	1.4157	12.4905	3.6428	up	LevelBii	HMDB0291106	1122.5379	3.527
Lps(22:6)	3.414E-5	0.0003061	1.4154	1.5804	0.6603	up	LevelBi	LMGP03050013	568.2636	8.3544
(4E,7E,10E)-Hexadeca-4,7,10-Trienedioic Acid	8.279E-5	0.0005895	1.4131	1.7129	0.7765	up	LevelBii	HMDB0340904	263.1657	2.0314
Ile-Thr-Trp	0.002332	0.007846	1.412	1.7588	0.8146	up	LevelBi	-	451.2517	2.3402
Homogentisic Acid	2.326E-5	0.0002266	1.4107	3.5867	1.8427	up	LevelBii	HMDB0000130	167.0336	4.0091
11-Cis-Retinol	0.01938	0.03826	1.4084	0.5343	-0.9042	down	LevelBi	HMDB0006216;LMPR01090005;PW_C002665	269.2254	9.4716
Ser-Val-Trp	0.003311	0.008738	1.4072	0.5529	-0.8548	down	LevelBi	-	391.1953	3.1528
Cis-3-Hexenyl Tiglate	0.00923	0.0206	1.405	0.5417	-0.8846	down	LevelBi	HMDB0038279;LMFA07010808	183.1367	7.7446

Ethyl Linoleate	0.01831	0.03656	1.405	0.7284	-0.4572	down	LevelBi	HMDB0252048	309.2774	8.4642
(R)-3,4-Dihydro-8-Hydroxy-3-Tridecyl-1H-2-Benzopyran-1-One	0.0004266	0.002097	1.4023	1.4568	0.5428	up	LevelBii	HMDB0041302	381.2234	8.1162
Ganolucidic Acid C	0.003775	0.009706	1.4017	5.293	2.4041	up	LevelBii	HMDB0039691	551.3617	2.6169
N-(3-Methylbut-2-En-1-Yl)-9H-Purin-6-Amine	9.6E-6	0.0001199	1.4	0.6524	-0.6161	down	LevelBii	HMDB0245646	204.1226	2.2904
Fahfa(18:3/3:0)	0.002088	0.007244	1.3978	0.6531	-0.6147	down	LevelBi	-	349.2363	8.5663
Stevioside	3.594E-5	0.0003197	1.3978	0.6307	-0.665	down	LevelBii	HMDB0034945	849.3723	5.7568
Moxaprindine	0.001172	0.00459	1.3962	0.5735	-0.802	down	LevelBii	HMDB0254906	387.2229	3.2919
Indolelactic Acid	1.604E-5	0.0001743	1.3955	0.7254	-0.4632	down	LevelBi	HMDB0000671	204.066	3.8785
Mesterolone	0.001159	0.004546	1.3931	0.5331	-0.9076	down	LevelBi	HMDB0006036;LMST02020107	269.225	5.361
(9S,10S)-9,10-Dihydroxyoctadecanoate	0.002576	0.008432	1.3918	0.7024	-0.5097	down	LevelBii	HMDB0059633	315.2529	7.1091
Cyclo(Prolylvalyl)	4.872E-6	7.43E-5	1.3916	0.6067	-0.7209	down	LevelBi	-	197.1275	2.7362
Amprenavir	6.392E-6	9.088E-5	1.3901	0.6889	-0.5376	down	LevelBii	HMDB0014839	1055.4407	3.602
Angiotensin Iv	8.76E-5	0.0006137	1.3897	0.5878	-0.7667	down	LevelBii	HMDB0001038	819.3978	5.5264
Kaempferol 3-(3'',6''-Diacetyl-2'',4''-Di-P-Coumaroylrhamnoside)	0.0005424	0.002524	1.3891	0.5804	-0.785	down	LevelBii	HMDB0029503	805.1708	4.139
3,10-Dihydroxydodecanoylcarnitine	0.001599	0.005865	1.3878	0.6213	-0.6867	down	LevelBii	HMDB0241292	417.2966	6.4371
Trimetrexate	1.22E-7	6.416E-6	1.3869	0.7222	-0.4695	down	LevelBii	HMDB0015288	390.1516	3.7948
Indole-3-Lactic Acid	1.746E-6	3.671E-5	1.3865	0.6969	-0.5209	down	LevelBi	HMDB0000671	206.0811	3.8823
6-Oxooctadecanoic Acid	0.03488	0.05906	1.3862	0.2206	-2.1803	down	LevelBi	-	299.2568	7.5999
3,4',5-Trihydroxy-3',7-Dimethoxyflavanone	1.58E-7	7.666E-6	1.3846	0.711	-0.4921	down	LevelBii	HMDB0037503	353.0663	2.5287
20-Hydroxyeicosatetraenoic Acid	0.003789	0.00974	1.3846	0.7731	-0.3713	down	LevelBii	HMDB0005998	321.2395	7.7446
Tyramine	3.469E-7	1.248E-5	1.3846	0.7628	-0.3906	down	LevelBi	HMDB0000306;PW_C000210	138.0905	1.8633
Abu-Lys-Oh	0.0003119	0.001645	1.3844	2.3562	1.2365	up	LevelBi	-	338.1327	2.0441
Farnesylcysteine	5.482E-5	0.0004376	1.3841	0.5978	-0.7423	down	LevelBii	HMDB0011627	326.213	6.7878
Tuberoside F	0.008468	0.01913	1.384	0.4557	-1.1337	down	LevelBii	HMDB0302986	1077.5425	6.5081
Prostaglandin A1	9.367E-5	0.0006449	1.3833	0.4926	-1.0214	down	LevelBii	HMDB0002656	381.2259	5.5725
Pi(Pgf1Alpha/20:0)	0.002747	0.008669	1.3821	1.384	0.4689	up	LevelBii	HMDB0277060	1021.619	7.6962
Lys-Tyr	0.02218	0.04275	1.3816	1.9226	0.943	up	LevelBi	HMDB0028963	310.1751	1.3911
Pisumionoside	0.001438	0.005404	1.3809	0.2666	-1.9073	down	LevelBii	HMDB0039947	449.2035	3.4518

Thr-Ala-Ile	1.08E-5	0.0001311	1.3794	1.5028	0.5876	up	LevelBi	-	304.1854	2.3901
Trp-Met	0.00393	0.01006	1.3782	0.5791	-0.788	down	LevelBi	HMDB0029089	336.1362	2.9844
1-(4-Methoxyphenyl)Cyclopropanecarboxylic Acid	8.652E-5	0.0006073	1.3778	0.6633	-0.5922	down	LevelBi	-	175.0745	4.0206
Elocalcitol	5.466E-5	0.0004368	1.3772	0.7741	-0.3693	down	LevelBii	HMDB0251753	443.3276	9.1971
Cdp-Dg(Pgj2/I-22:0)	0.03032	0.05519	1.3768	0.5698	-0.8114	down	LevelBii	HMDB0294330	1133.6129	6.2901
3-Hydroxyquinidine	5.407E-6	8.022E-5	1.3752	0.712	-0.4901	down	LevelBii	HMDB0245907	395.1972	2.0314
Dopa 3'-Glucoside	5.458E-7	1.668E-5	1.3747	0.6686	-0.5808	down	LevelBii	HMDB0029452	394.0921	2.5665
5-Tetradecynoic Acid	0.02418	0.04588	1.3747	0.6061	-0.7223	down	LevelBi	LMFA01030586	225.1841	7.7446
Karanjachromene	0.0002264	0.001271	1.3729	1.4237	0.5096	up	LevelBi	LMPK12111544	339.0994	4.2764
Misoprostol Acid	0.006909	0.01614	1.372	0.5728	-0.8039	down	LevelBii	HMDB0254756	389.2282	7.6204
Moexipril	2.852E-5	0.000264	1.3716	0.5432	-0.8805	down	LevelBii	HMDB0014829	995.4645	4.4631
Abeado	1.866E-5	0.0001949	1.3703	1.7453	0.8035	up	LevelBii	HMDB0247746	370.1592	2.6894
Sulfolithocholic Acid	0.001842	0.00657	1.3696	1.7531	0.8099	up	LevelBii	HMDB0000907	457.261	2.9348
Taurohyocholic Acid	0.01937	0.03825	1.3683	2.0347	1.0248	up	LevelBi	LMST05040010	538.2826	2.2211
Gly-Phe-Leu-Gly	1.52E-5	0.0001676	1.368	1.8429	0.882	up	LevelBii	HMDB0252827	373.1856	3.8507
3-Phenyllactic Acid	9.914E-7	2.463E-5	1.3674	0.8127	-0.2991	down	LevelBi	HMDB0000779	165.0553	3.7495
N2-(3-Hydroxysuccinoyl)Arginine	9.761E-7	2.438E-5	1.3632	0.6393	-0.6453	down	LevelBii	HMDB0032765	323.156	3.9219
Fumonisin A2	0.00221	0.007554	1.363	0.4432	-1.1739	down	LevelBii	HMDB0034699	712.3915	3.6363
Cholylcitrulline	0.03547	0.05982	1.363	1.714	0.7773	up	LevelBii	HMDB0242383	610.3703	8.1796
Adenine	7.729E-6	0.0001047	1.3627	0.7293	-0.4554	down	LevelBi	HMDB0000034;PW_C000024	136.0618	1.1086
Adenosine 2',3'-Cyclic Phosphate	9.897E-7	2.463E-5	1.3614	1.4795	0.5651	up	LevelBii	HMDB0011616	364.0246	1.4267
Avocadyne 4-Acetate	0.02569	0.04821	1.3612	0.3202	-1.643	down	LevelBi	HMDB0031049;LMFA05000645	325.2358	7.0254
Lpe(16:1)	0.009872	0.02182	1.361	1.4174	0.5032	up	LevelBi	HMDB0011474	450.2606	7.9966
2-Cyclopropyl-4-Quinolinecarboxylic Acid	0.0003236	0.00169	1.3609	0.6862	-0.5433	down	LevelBi	-	214.0853	2.9844
Gln-Arg-Thr	0.0007531	0.003257	1.36	0.6613	-0.5967	down	LevelBi	-	445.2503	6.035
N(Epsilon)-(Carboxymethyl)Hydroxylysine	0.0002139	0.001216	1.3593	0.4644	-1.1067	down	LevelBii	HMDB0250375	201.0877	2.0063
2"-O-Acetylisoorientin	1.174E-8	1.36E-6	1.3585	0.6348	-0.6557	down	LevelBii	HMDB0038816	525.0822	2.5287
Ziritaxestat	0.004619	0.01153	1.358	0.5975	-0.743	down	LevelBii	HMDB0252755	630.2794	1.8534
Merodesmosine	0.0002145	0.001216	1.3574	0.7281	-0.4578	down	LevelBii	HMDB0030407	403.2527	2.8059

2-Hydroxy-6-Pentadecylbenzoic Acid	0.002878	0.008669	1.3569	0.6407	-0.6422	down	LevelBii	HMDB0029683	383.2393	7.3777
Cdp-Dg(Pgf1Alpha/20:1(11Z))	0.0009538	0.003923	1.3544	0.6639	-0.5909	down	LevelBii	HMDB0291796	1090.5679	5.0586
Core Oligosaccharide	0.0002184	0.001233	1.3533	1.4858	0.5712	up	LevelBii	HMDB0250452	755.2449	1.1003
Gibberellin A3	0.0006064	0.002762	1.353	0.7219	-0.4702	down	LevelBii	HMDB0003559	391.1368	3.6382
Gibberellin A39	7.972E-6	0.0001067	1.3485	1.4214	0.5073	up	LevelBii	HMDB0035045	429.1339	2.7839
4-Hydroxycinnamyl Aldehyde	1.221E-6	2.861E-5	1.3479	0.7903	-0.3394	down	LevelBi	HMDB0040986	147.0446	3.7495
6-Hydroxymelatonin	0.001689	0.006124	1.3472	0.5603	-0.8357	down	LevelBii	HMDB0004081	497.2443	3.1925
(2E,4E)-N-(1-Hydroxy-3-Methylpentan-2-Yl)-2-Methylhexa-2,4-Dienamide	0.02916	0.05345	1.3471	2.8745	1.5233	up	LevelBi	-	208.169	5.6249
1-Linoleoyl-Sn-Glycero-3-Phosphorylcholine	0.004306	0.01087	1.3466	1.2327	0.3018	up	LevelBi	HMDB0010386	520.3381	7.6582
Pc(Pgf2Alpha/16:0)	0.01806	0.03615	1.3464	2.508	1.3265	up	LevelBii	HMDB0285926	812.5441	7.3964
Ile-His	2.647E-5	0.0002505	1.3435	0.6395	-0.645	down	LevelBi	HMDB0028909	269.1594	1.0591
Harmol Glucuronide	0.000606	0.002761	1.3432	1.4165	0.5023	up	LevelBii	HMDB0253051	339.0992	3.7845
Methyl Octadecadienoate	0.02966	0.05417	1.3414	0.7022	-0.51	down	LevelBi	-	327.2882	8.4447
Testosterone Glucuronide	0.001616	0.005913	1.3414	1.5497	0.6319	up	LevelBii	HMDB0003193	465.2439	2.9447
9,10-Epoxystearic Acid	0.004949	0.01221	1.3407	0.7983	-0.325	down	LevelBii	HMDB0247617	297.2428	7.7478
Esmolol	0.0001808	0.001067	1.3406	1.5726	0.6532	up	LevelBii	HMDB0014333	316.1503	2.3208
Oleyl Alcohol	0.001457	0.005461	1.3395	0.466	-1.1015	down	LevelBii	HMDB0029632	286.3099	7.0892
12,15-Epoxy-13,14-Dimethyloctadeca-12,14,16-Trienoic Acid	0.001306	0.005006	1.3369	0.4293	-1.2198	down	LevelBii	HMDB0112071	355.2076	6.8774
Cdp-Dg(6 Keto-Pgf1Alpha/18:0)	0.009864	0.02182	1.3369	0.6884	-0.5386	down	LevelBii	HMDB0291000	1096.5449	6.8269
9-Octadecynoic Acid	0.007983	0.0182	1.3363	0.5437	-0.8792	down	LevelBi	LMFA01030455	281.2466	7.1087
(E)-Zeatin Glucoside	0.001231	0.00478	1.3323	0.5397	-0.8898	down	LevelBii	HMDB0303782	440.1777	2.9885
Cinnamyl Phenylacetate	5.887E-7	1.758E-5	1.331	0.4561	-1.1325	down	LevelBii	HMDB0037707	273.0884	1.9403
Alpha-Calacorene	0.003832	0.009842	1.331	0.5795	-0.7872	down	LevelBi	HMDB0036453	201.1629	6.447
Mucronine D	0.007	0.01634	1.331	0.4463	-1.1639	down	LevelBii	HMDB0029335	666.3645	3.8235
(3S)-3-(Benzyloxy)-L-Aspartic Acid	0.0007833	0.003362	1.3308	0.5326	-0.9088	down	LevelBii	HMDB0250058	238.0742	1.156
12-O-Retinoylphorbol-13-Acetate	0.0004995	0.002366	1.3303	0.6741	-0.569	down	LevelBii	HMDB0249729	743.4157	5.7913
Roxindole	1.446E-6	3.242E-5	1.3298	0.5974	-0.7431	down	LevelBii	HMDB0257319	405.2205	2.7743
Porfiromycin	9.8E-5	0.0006683	1.3294	1.604	0.6817	up	LevelBii	HMDB0256703	393.1385	2.2353
1,4,10,13-Tetraoxa-7,16-Diazacyclooctadecane	1.356E-6	3.095E-5	1.3294	0.7611	-0.3939	down	LevelBii	HMDB0244223	263.1955	1.7269

Corchorifatty Acid F	0.0003065	0.001622	1.3286	0.7346	-0.445	down	LevelBii	HMDB0035919	327.2157	6.2954
Leu-Val-Trp	0.001357	0.005147	1.3266	0.6335	-0.6585	down	LevelBi	-	417.2467	3.7253
3-O-Beta-D-Galactopyranosyl-L-Arabinose	0.04554	0.0738	1.3261	0.247	-2.0173	down	LevelBii	HMDB0038854	347.0747	4.6386
3,4-Diethoxybenzaldehyde	4.246E-6	6.761E-5	1.3236	0.638	-0.6484	down	LevelBi	-	217.0842	5.0781
Pe(O-12:0/20:4)	1.104E-5	0.0001334	1.3232	0.7385	-0.4373	down	LevelBi	-	668.4662	8.6598
[6]-Gingerdiol 3,5-Diacetate	0.0006205	0.002808	1.3227	0.718	-0.478	down	LevelBi	HMDB0040568	379.2086	6.6467
Delprostenato	0.0006976	0.003071	1.3214	0.4834	-1.0486	down	LevelBii	HMDB0250975	435.1536	2.491
Ent-6R,16Boh,17-Trihydroxy-7-Oxo-6,7-Seco-19,6-Kauranolide 6-O-Glucoside	0.005847	0.01405	1.321	3.4245	1.7759	up	LevelBii	HMDB0038546	493.2386	2.786
Hyp-Val	0.01225	0.02612	1.3209	1.6321	0.7067	up	LevelBii	HMDB0028876	251.1021	2.082
9-Hpode	0.001314	0.005025	1.3207	0.7752	-0.3673	down	LevelBi	HMDB0062434;HMDB0242602	311.2213	6.6467
Bn-Ncc-2	0.006704	0.01576	1.3199	0.6057	-0.7234	down	LevelBii	HMDB0038230	837.3169	4.0091
6-Hydroxy-2-Aminopurine	0.000154	0.0009423	1.3193	1.3517	0.4348	up	LevelBi	HMDB0000132	152.0558	1.9821
Pro-Leu-Pro	1.316E-7	6.798E-6	1.3174	0.8134	-0.298	down	LevelBi	-	326.2059	2.1814
Val-Trp	4.606E-5	0.0003873	1.3165	0.7751	-0.3675	down	LevelBi	HMDB0029138	304.1648	2.8754
N-Linoleoyl Threonine	4.777E-5	0.0003977	1.3162	4.6004	2.2018	up	LevelBii	HMDB0241985	440.2997	6.4062
Ile-Pro-Pro	5.074E-7	1.612E-5	1.3153	0.7562	-0.4032	down	LevelBii	HMDB0253021	324.1914	2.1976
Hemicalyculin A	0.02279	0.04368	1.3142	1.4495	0.5355	up	LevelBii	HMDB0253083	1006.6312	8.0959
Pyrodone	0.001463	0.005483	1.313	0.5916	-0.7572	down	LevelBii	HMDB0256988	320.1844	4.7335
Alpha-Curcumene	0.005648	0.01364	1.3114	0.5461	-0.8727	down	LevelBi	HMDB0061838;HMDB0059878;LMPR0103060015	203.1781	6.447
Maltotetraose	0.0027	0.008669	1.3111	1.5481	0.6305	up	LevelBi	HMDB0001296	667.2244	1.0985
Withangulatin A	8.011E-5	0.0005755	1.3107	1.5755	0.6558	up	LevelBii	HMDB0037383	527.2627	2.8357
Ser-Val	0.0003016	0.001601	1.3095	0.5791	-0.788	down	LevelBii	HMDB0029052	203.1023	0.727
Anapear	0.003068	0.008669	1.3094	0.5914	-0.7577	down	LevelBii	HMDB0040285	155.1059	6.0938
Glycerophosphoinositol	0.03389	0.05764	1.3084	0.3873	-1.3683	down	LevelBii	HMDB0011649	333.0592	4.0091
N6-Galacturonyl-L-Lysine	0.0005839	0.002676	1.3062	0.3785	-1.4017	down	LevelBii	HMDB0033106	343.1105	1.3148
Sn38 Glucuronide	2.624E-6	4.801E-5	1.3047	1.5229	0.6068	up	LevelBii	HMDB0060511	567.165	3.1396
Octanamide	0.0257	0.04822	1.3046	0.2224	-2.1685	down	LevelBii	HMDB0255903	185.1636	2.2904
Alismoxide	0.0002165	0.001225	1.3007	0.6113	-0.7099	down	LevelBi	-	293.2102	6.1823
Trp-Phe	8.592E-5	0.0006046	1.2995	0.7337	-0.4467	down	LevelBi	HMDB0029090	352.1642	3.735

Vigabatrin	1.348E-5	0.0001539	1.299	0.6801	-0.5561	down	LevelBii	HMDB0015212	174.0763	2.3398
7-Methylguanosine	4.746E-6	7.282E-5	1.2962	1.5728	0.6534	up	LevelBii	HMDB0001107	319.0885	2.082
Soybean Saponin Bg	0.02202	0.04251	1.2953	0.6196	-0.6907	down	LevelBii	HMDB0302658	945.4665	7.3871
Lpc(22:6)	0.00611	0.01457	1.295	1.4026	0.4881	up	LevelBi	HMDB0010404	612.3286	7.5551
Clarithromycin	7.027E-6	9.722E-5	1.2939	1.3347	0.4166	up	LevelBii	HMDB0015342	802.4858	7.6582
Glycerophosphocholine	1.382E-5	0.0001565	1.2931	1.3755	0.4599	up	LevelBi	HMDB0000086;PW_C000059	258.1092	0.6595
B-D-Glucuronopyranosyl-(1->3)-A-D-Galacturonopyranosyl-(1->2)-L-Rhamnose	0.0004019	0.002013	1.2929	3.5512	1.8283	up	LevelBii	HMDB0039728	497.1103	2.0252
Lisinopril, Epsilon-Biotinamidocaproyl-	0.000379	0.00192	1.2927	0.6738	-0.5697	down	LevelBii	HMDB0244524	777.4202	5.5174
Fa(20:3)	0.002203	0.007539	1.2918	0.7248	-0.4643	down	LevelBi	-	611.5024	8.88
3-(1H-Indol-3-Yl)-2-(Trimethylazaniumyl)Propanoate	3.776E-6	6.24E-5	1.2912	1.511	0.5955	up	LevelBi	-	247.1424	2.4872
Citric Acid	2.129E-6	4.183E-5	1.2901	1.2496	0.3214	up	LevelBi	HMDB0000094;PW_C000063	191.0189	0.7644
Calendoflavoside	0.03512	0.05937	1.289	658.7086	9.3635	up	LevelBii	HMDB0037745	623.16	3.9158
Cys-Trp-Ala	2.229E-6	4.291E-5	1.286	0.7101	-0.4939	down	LevelBii	HMDB0341149	437.1536	2.9509
Byssochlamic Acid	0.009807	0.02171	1.2845	0.5179	-0.9492	down	LevelBii	HMDB0034262	353.1007	4.3708
Lpe(20:5)	0.003013	0.008669	1.2839	1.3183	0.3987	up	LevelBi	HMDB0011489	498.2614	7.3591
Phe-His	0.002472	0.008176	1.2826	1.622	0.6977	up	LevelBi	HMDB0028892	303.1433	1.6688
Solasodine Base + 2H + H2O, O-Hex-Hex-Hex-Pen	0.001671	0.006067	1.281	0.7418	-0.4308	down	LevelBi	-	1034.5437	5.029
Pe(O-16:1/20:5)	0.002384	0.007977	1.2804	1.5362	0.6193	up	LevelBi	-	720.5021	9.9073
Lysope(16:1(9Z)/0:0)	0.01599	0.03258	1.2793	1.3745	0.4589	up	LevelBi	HMDB0011504	452.2761	7.9885
Cis-3-Methyl Thiofentanyl	0.004002	0.01021	1.2791	0.6181	-0.6941	down	LevelBi	-	357.2009	5.7522
Lys-Phe	0.03694	0.06188	1.279	1.5593	0.6409	up	LevelBi	HMDB0028958	294.1805	1.5909
Grazoprevir	6.94E-6	9.649E-5	1.278	0.7711	-0.375	down	LevelBii	HMDB0252939	747.3119	3.6111
Ala-Gly-Tyr	0.01914	0.03786	1.2779	1.719	0.7816	up	LevelBi	-	310.138	1.8336
5Beta-Androstan-3Alpha-Ol-17-One Sulfate	0.0003928	0.001978	1.277	2.3686	1.244	up	LevelBi	-	369.1765	2.463
N-Arachidonoyl Proline	0.03431	0.05825	1.2763	0.1886	-2.4063	down	LevelBii	HMDB0242002	436.2654	7.3219
Pc(O-18:2)	0.009891	0.02185	1.2761	1.4429	0.529	up	LevelBi	-	558.2919	7.6487
2-(4-Fluorophenoxy)-N-[2-Oxo-2-(4-Pyridin-2-Ylpiperazin-1-Yl)Ethyl]Acetamide	5.459E-5	0.0004367	1.2751	0.4632	-1.1103	down	LevelBi	-	373.1703	4.9414
2-(5,8-Tetradecadienyl)Cyclobutanone	0.003008	0.008669	1.2748	0.7675	-0.3817	down	LevelBii	HMDB0037519	263.2354	8.7842
Roridin A	0.01569	0.03207	1.2703	0.6769	-0.563	down	LevelBi	-	533.2705	4.1586

Aconitic Acid	8.451E-6	0.0001101	1.2688	1.3755	0.46	up	LevelBi	HMDB0247961	173.0079	1.9403
Estradiol Enanthate	0.0002524	0.001384	1.2687	0.7262	-0.4616	down	LevelBii	HMDB0251964	419.2386	8.8332
5-[2-[(E)-4-Carboxybut-1-Enyl]-6-Hydroxy-4-Methoxybenzoyl]Oxyhexanoic Acid	3.25E-8	2.597E-6	1.2687	0.6492	-0.6232	down	LevelBi	-	381.1549	4.5992
Cis-9-Hexadecenoic Acid	0.00756	0.01741	1.2676	0.7392	-0.4359	down	LevelBi	HMDB0003229	255.2312	7.5307
Hydroxy-Lacosamide	0.0001139	0.0007538	1.2674	0.6391	-0.6458	down	LevelBii	HMDB0061146	281.1129	1.8366
Pip(5-Iso Pgf2Vi/20:4(5Z,8Z,11Z,14Z))	0.01232	0.02624	1.267	0.6197	-0.6903	down	LevelBii	HMDB0280017	989.4475	5.9605
(2S,3S)-Nemonapride	0.004692	0.01168	1.2667	0.5392	-0.891	down	LevelBii	HMDB0255510	388.182	2.9943
Tetrahydroharman-3-Carboxylic Acid	0.0001204	0.0007836	1.2651	0.7841	-0.3508	down	LevelBi	-	231.1123	2.9844
3-(2-Furanylmethylene)-2,4-Pentanedione	0.01501	0.03094	1.2638	90.2275	6.4955	up	LevelBi	-	179.0693	5.664
Mikamycin B	0.0001053	0.0007089	1.2626	0.7579	-0.4	down	LevelBii	HMDB0256777	901.374	3.6111
Ile-Ile-Asp	0.0006654	0.002967	1.2623	0.7482	-0.4186	down	LevelBi	-	360.2105	2.2507
Kaempferol	0.02127	0.04131	1.2609	0.5927	-0.7547	down	LevelBi	HMDB0005801;HMDB0032008;LMPK12110003	287.053	4.9024
Pe(O-8:0/10:0)	0.01027	0.02256	1.26	1.367	0.451	up	LevelBi	-	961.6266	8.669
Pe-Cer(12:2_2O/12:0)	0.03793	0.06326	1.2586	1.5419	0.6247	up	LevelBi	-	1035.6996	9.5133
Ala-Ile-Lys	0.0099	0.02186	1.2578	0.4595	-1.122	down	LevelBi	-	353.2185	3.2123
Tyr-Asp	0.004472	0.01121	1.2553	1.7574	0.8134	up	LevelBi	HMDB0029101	297.1059	1.8336
Butyrylcarnitine	3.903E-5	0.0003399	1.2551	1.2258	0.2937	up	LevelBi	HMDB0002013;LMFA07070054	232.1542	2.1616
2-Methyl-4-(Pyridin-2-Yl)But-3-Yn-2-Ol	7.664E-5	0.0005595	1.2548	0.7619	-0.3923	down	LevelBi	-	144.0799	2.8653
3-[5-(2-Methylpropyl)-3,6-Dioxopiperazin-2-Yl]Propanoic Acid	0.002177	0.00748	1.254	1.2839	0.3606	up	LevelBi	-	243.1335	2.2211
Methyl 5-Hydroxyoxindole-3-Acetate	2.716E-5	0.0002544	1.2528	0.4195	-1.2534	down	LevelBii	HMDB0038990	242.0423	4.3059
Methyl Benzoylformate	0.0002279	0.001276	1.2516	1.3584	0.4419	up	LevelBi	HMDB0062605	165.0538	0.7856
Pe(Pgf1Alpha/P-18:0)	0.005531	0.01341	1.2504	1.4728	0.5586	up	LevelBii	HMDB0285371	858.5805	8.1635
Ala-Tyr-Lys	3.531E-6	5.995E-5	1.2503	0.8159	-0.2936	down	LevelBi	-	381.2105	2.0614
Car(5:0)	0.0001762	0.001046	1.2494	1.3141	0.3941	up	LevelBi	-	246.1696	2.4775
1-O-Isopentyl-3-O-Octadec-2-Enoyl Glycerol	0.009422	0.02099	1.248	0.6949	-0.525	down	LevelBii	HMDB0302897	468.4018	7.6097
Zotarolimus	0.03376	0.05747	1.2479	1.6348	0.7091	up	LevelBii	HMDB0260048	1029.5873	7.8024
Pro-Pro	1.933E-7	8.648E-6	1.2461	0.7542	-0.4069	down	LevelBi	HMDB0011180	213.1222	0.737
Asn-Met-Gly	0.01968	0.03873	1.2453	1.9317	0.9498	up	LevelBii	HMDB0341135	355.0863	7.8388
Pe(Pgf1Alpha/P-16:0)	0.000724	0.003165	1.2449	0.6642	-0.5903	down	LevelBii	HMDB0285267	776.5416	7.7349

5-Hexyl-2-Furanoctanoic Acid	0.01873	0.03719	1.2412	1.8863	0.9156	up	LevelBii	HMDB0112099	315.195	7.5177
Candesartan Cilexetil	0.0001914	0.001116	1.2409	2.3058	1.2052	up	LevelBii	HMDB0249581	609.2494	3.273
Phe-Gly	0.001732	0.006256	1.2408	1.3453	0.428	up	LevelBi	HMDB0028995	223.107	2.1616
10-Formyldihydrofolate	1.647E-6	3.524E-5	1.2404	0.7012	-0.5122	down	LevelBii	HMDB0006485	470.1423	2.5853
Abu-Abu-Oh	0.01759	0.03534	1.2403	1.8512	0.8884	up	LevelBi	-	295.0924	1.8366
9-((2-Hydroxyethoxy)Methyl)Hypoxanthine	0.004964	0.01224	1.2385	0.757	-0.4016	down	LevelBi	-	211.0819	1.9821
4-(4-Hydroxyphenyl)-2-Butanone O-[2-Galloyl-6-Cinnamoylglucoside]	1.37E-5	0.0001558	1.2368	1.4615	0.5475	up	LevelBii	HMDB0039313	653.1906	2.6042
Acetyl-L-Carnitine	1.132E-7	6.086E-6	1.2357	1.2161	0.2823	up	LevelBi	HMDB0000201;LMFA07070050	204.1226	0.7273
L-Tryptophan	0.001115	0.004422	1.235	1.2461	0.3175	up	LevelBi	HMDB0000929;PW_C000741	205.0965	2.3901
N-Succinyl-Chitosan	0.003655	0.009467	1.2328	1.6087	0.6859	up	LevelBii	HMDB0255242	582.2121	2.3398
5,6,7,8-Tetrahydroquinolin-8-Ol	8.297E-5	0.0005901	1.2322	0.7871	-0.3454	down	LevelBi	-	132.0797	2.4099
N-Feruloylglycyl-L-Phenylalanine	3.461E-7	1.248E-5	1.2316	0.714	-0.486	down	LevelBii	HMDB0029356	397.1437	2.5853
(3S,5R,6R,7E)-3,5,6-Trihydroxy-7-Megastigmen-9-One	0.0001685	0.001008	1.2274	1.3903	0.4754	up	LevelBii	HMDB0038736	225.1476	3.133
Benzo[Cd]Indol-2(1H)-One	0.005796	0.01395	1.227	1.3575	0.4409	up	LevelBi	-	170.0593	2.4
Bestatin	0.04934	0.07883	1.2264	0.331	-1.5951	down	LevelBi	HMDB0242556	350.207	3.7153
Myristoleic Acid	0.008853	0.01987	1.2263	0.7545	-0.4065	down	LevelBi	HMDB0002000;LMFA01030051	227.1994	8.1247
Phe-Trp	0.001554	0.005748	1.2247	0.676	-0.565	down	LevelBi	HMDB0029006	352.1638	3.5177
Flavin Adenine Dinucleotide (Fad)	0.006726	0.0158	1.2237	1.6328	0.7074	up	LevelBi	-	784.1472	2.5287
Prostaglandin A2	0.0008924	0.003728	1.2235	0.6564	-0.6075	down	LevelBi	HMDB0002752;PW_C001699	335.2202	5.3319
Lyciumoside Iv	0.01264	0.02683	1.221	0.7762	-0.3655	down	LevelBii	HMDB0033499	821.415	5.462
3-Hydroxytrideca-4,6-Dienoic Acid	0.01456	0.03016	1.2204	2.6933	1.4294	up	LevelBii	HMDB0340751	225.1492	5.6277
Drostanolone	0.04094	0.06745	1.2193	0.5253	-0.9289	down	LevelBi	HMDB0014996	269.2237	7.8024
Aspartame	0.01593	0.03249	1.2184	1.4477	0.5337	up	LevelBii	HMDB0001894	293.113	2.1593
1-(2,2,6-Trimethylcyclohexyl)Hexan-3-Ol	0.006384	0.01512	1.217	1.4015	0.487	up	LevelBi	-	268.262	8.435
Gly-Phe	0.001739	0.006275	1.2169	1.3643	0.4482	up	LevelBi	HMDB0028848	223.1071	2.3204
Nikkomycin	0.002557	0.008383	1.2162	1.4188	0.5047	up	LevelBii	HMDB0255605	478.1608	0.708
N-L-Gamma-Glutamyl-L-Leucine	9.069E-6	0.0001153	1.2156	0.7478	-0.4193	down	LevelBi	HMDB0011171	261.1461	2.5469
Capsianoside Vi	0.01586	0.03236	1.2153	0.6393	-0.6454	down	LevelBii	HMDB0039165	923.4767	6.2803
N-Cyclohexyl-9H-Purin-6-Amine	3.14E-7	1.184E-5	1.215	1.3303	0.4118	up	LevelBi	-	218.1379	1.9523

Protoporphyrin Ix	0.01968	0.03873	1.2142	0.7674	-0.3819	down	LevelBii	HMDB0000241	1123.5138	6.8225
Glu-Ile-Lys	0.000358	0.001834	1.2134	1.4057	0.4913	up	LevelBi	-	371.2278	2.1616
Ser-Phe	0.003729	0.009607	1.2131	1.3603	0.4439	up	LevelBi	HMDB0029046	253.1175	2.2706
Avenestergenin B2	0.0001511	0.000929	1.2114	1.3974	0.4828	up	LevelBii	HMDB0035266	627.3422	9.2703
Methyl Oxindole-3-Acetate	0.0001051	0.000708	1.2097	1.6988	0.7645	up	LevelBii	HMDB0303691	250.0709	3.1772
Ethyl Cellulose	0.02195	0.04241	1.2093	0.6341	-0.6572	down	LevelBii	HMDB0033370	455.253	2.786
Thalrugosine	0.01622	0.03297	1.2087	0.563	-0.8287	down	LevelBi	HMDB0242612	609.295	2.1112
Leukotriene D4 Methyl Ester	0.000179	0.001059	1.208	0.7754	-0.3669	down	LevelBi	-	511.2843	2.4099
Celosianin	0.004276	0.0108	1.207	0.571	-0.8084	down	LevelBii	HMDB0302989	905.1695	4.1484
Sibrafiban	0.007055	0.01644	1.2059	1.6505	0.7229	up	LevelBii	HMDB0258287	465.1973	2.1105
Secoeremopetasitolide B	8.356E-6	0.0001095	1.2058	0.578	-0.7909	down	LevelBii	HMDB0041363	427.2291	3.123
Ala-Pro-Pro	2.562E-6	4.736E-5	1.2058	0.8162	-0.293	down	LevelBi	-	284.1588	1.9821
Trp-Ile	0.0004985	0.002363	1.2017	0.7463	-0.4223	down	LevelBii	HMDB0029086	316.1655	3.5738
Lobucavir	0.004914	0.01213	1.201	1.6282	0.7033	up	LevelBii	HMDB0254142	310.1182	4.7981
Xylan	0.004125	0.01047	1.2008	1.7239	0.7857	up	LevelBii	HMDB0301774	377.0941	1.912
Fahfa(18:2/3:0)	0.0008665	0.003638	1.2	0.8056	-0.3119	down	LevelBi	-	351.2523	8.8332
Beta-Sitosterol 3-O-Beta-D-Galactopyranoside	0.0268	0.04987	1.1994	0.5285	-0.92	down	LevelBii	HMDB0033949	597.4083	8.6411
1-O-Hexadecyl-2-O-Ethyl-Sn-Glycero-3-Phosphocholine	0.001202	0.004688	1.198	0.8542	-0.2273	down	LevelBi	-	510.3887	8.8826
Pro-Tyr-Ile	0.01959	0.0386	1.1976	1.4651	0.551	up	LevelBi	-	392.2156	3.0537
2-Naphthyl Laurate	4.007E-5	0.0003472	1.1975	0.8693	-0.2022	down	LevelBi	-	344.261	8.6115
Tyr-Glu	0.01315	0.0277	1.1975	1.3926	0.4778	up	LevelBi	HMDB0029104	311.1224	1.9124
Tyr-Asn	0.02143	0.04158	1.1956	1.6473	0.7201	up	LevelBi	HMDB0029100	296.1229	1.4008
Phe-Hyp	1.988E-5	0.0002041	1.1935	0.7595	-0.397	down	LevelBii	HMDB0011176	261.1224	3.2023
St(24:2_O4)	0.002189	0.007512	1.193	0.6317	-0.6628	down	LevelBi	-	435.2732	6.6005
Fleroxacin	1.887E-5	0.0001966	1.1921	0.6239	-0.6807	down	LevelBii	HMDB0252299	368.1207	2.7176
Chlorphenesin	0.006165	0.01467	1.1912	1.6063	0.6838	up	LevelBii	HMDB0014994	247.0395	0.6896
Inuline	0.002572	0.00842	1.191	0.5244	-0.9313	down	LevelBii	HMDB0248466	585.3231	4.3337
Octadecenedioic Acid	0.0034	0.008936	1.1905	0.7481	-0.4187	down	LevelBi	HMDB0304442	335.2179	6.2705
12-Oxo Phytodienoic Acid	0.001296	0.004977	1.1902	0.7484	-0.4181	down	LevelBi	-	293.2096	5.5174

Leu-Trp	0.0001414	0.0008805	1.1883	0.8034	-0.3158	down	LevelBi	HMDB0028940	318.1802	3.1925
7-Oxo-11-Dodecenoic Acid	0.0009025	0.003763	1.1875	0.7643	-0.3877	down	LevelBi	LMFA01060182	213.1477	5.801
Ile-Leu-Gln	0.0107	0.02334	1.1867	0.709	-0.4962	down	LevelBi	-	373.2422	2.1616
6-Hydroxy-5-Methoxyindole Glucuronide	0.0004428	0.002157	1.1861	1.6154	0.6919	up	LevelBii	HMDB0010362	360.072	3.4613
Val-Pro-Pro	1.461E-6	3.263E-5	1.1856	0.8374	-0.256	down	LevelBi	HMDB0253034	312.1903	2.1112
Annomuricatin A	1.227E-5	0.0001436	1.1853	0.8154	-0.2944	down	LevelBii	HMDB0303181	593.2504	3.6111
(2S)-2-[[(2R)-2-Amino-4-Carboxybutanoyl]Amino]-4-Methylpentanoic Acid	0.001515	0.005636	1.1852	1.2535	0.3259	up	LevelBi	-	261.1434	2.2211
Deoxynivalenol 3-Glucoside	0.004232	0.01071	1.1848	0.3785	-1.4017	down	LevelBii	HMDB0039852	476.2132	3.0437
Leu-Val-His	0.0002595	0.001413	1.1843	0.7807	-0.3572	down	LevelBi	-	368.2266	1.8827
20-Hydroxy-6Z,15Z-Eicosadienoic Acid	0.04164	0.06839	1.1827	0.7262	-0.4616	down	LevelBii	HMDB0245564	325.2719	8.4642
Capsianoside Ii	0.03184	0.05725	1.1819	0.7666	-0.3835	down	LevelBii	HMDB0031892	1085.5305	5.4492
16Alpha-Fluoroestradiol	6.684E-7	1.886E-5	1.1817	0.7087	-0.4968	down	LevelBii	HMDB0244757	325.1388	2.1105
Lys-Trp	1.415E-6	3.195E-5	1.1809	1.285	0.3617	up	LevelBii	HMDB0028962	355.1708	4.3449
N5-Acetyl-N2-Gamma-L-Glutamyl-L-Ornithine	0.001712	0.006196	1.1808	0.6621	-0.5948	down	LevelBii	HMDB0039423	324.1161	1.3241
Lpe(18:3)	0.004148	0.01052	1.1797	1.3578	0.4413	up	LevelBi	HMDB0011478	474.2603	7.4709
9,10,13-Trihome	0.001024	0.004138	1.1761	0.8362	-0.2581	down	LevelBii	HMDB0004710	329.2323	5.8125
Asp-Pro-Ile	0.0001182	0.0007725	1.1746	1.3275	0.4087	up	LevelBi	-	344.1828	2.4099
Paliperidone	1.751E-6	3.671E-5	1.1745	0.6942	-0.5266	down	LevelBii	HMDB0015396	447.1824	2.0631
Cyclocalamin	1.086E-6	2.605E-5	1.1745	0.8284	-0.2717	down	LevelBii	HMDB0035863	537.1916	2.1688
Eriojaposide B	0.0423	0.06936	1.1733	0.686	-0.5436	down	LevelBii	HMDB0038029	1077.5079	7.3405
3-Carboxy-4-Methyl-5-Pentyl-2-Furanpropanoic Acid	0.02015	0.0395	1.1729	1.941	0.9568	up	LevelBii	HMDB0061643	289.1044	5.9051
Desmethyldeschlorobenzoyl Indomethacin	0.0002171	0.001227	1.1716	1.4334	0.5194	up	LevelBi	-	190.0499	3.2431
Phenol	0.007326	0.01697	1.1705	1.5309	0.6144	up	LevelBi	HMDB0000228;PW_C000153	95.0484	1.8436
Vertilmicin	0.001636	0.00597	1.1688	0.6781	-0.5605	down	LevelBii	HMDB0242753	528.2835	2.8952
Lpc(22:5-Sn1)	0.02695	0.05014	1.1685	1.5227	0.6066	up	LevelBi	-	592.3326	7.8902
gamma-Glu-Phe	0.0002043	0.001171	1.1674	1.3151	0.3951	up	LevelBii	HMDB0000594	275.1027	3.48
N-Arachidonoyl Alanine	0.0004575	0.002207	1.1669	0.8147	-0.2957	down	LevelBii	HMDB0241989	358.276	8.2812
Imigliptin	0.01277	0.02705	1.1667	0.5365	-0.8982	down	LevelBii	HMDB0253428	341.191	2.2211
18-Carboxy-Dinor-Lte4	0.03656	0.06134	1.1661	0.6276	-0.6722	down	LevelBii	HMDB0012607	883.3675	6.0742

Val-Phe-Asp	0.0004044	0.002022	1.1645	0.7337	-0.4467	down	LevelBi	-	380.1804	2.37
Dihydro-2,5-Furandione	1.13E-5	0.0001355	1.1622	1.2773	0.3531	up	LevelBi	HMDB0032523	101.0226	1.7849
4'-Hydroxycarvedilol	5.647E-7	1.718E-5	1.1616	0.7543	-0.4067	down	LevelBii	HMDB0013947	403.1696	2.5571
Trp-Val-Phe	0.007834	0.01792	1.1612	0.5459	-0.8734	down	LevelBi	-	451.2308	4.2174
1-Methoxy-2-Propanol	9.188E-6	0.0001163	1.1591	0.7955	-0.33	down	LevelBi	HMDB0243915	55.0542	0.737
Ile-Ile-Glu	0.01014	0.02232	1.1577	0.6445	-0.6337	down	LevelBi	-	374.2252	2.517
1,1'-(Phenylazanediyl)Dipropan-2-Ol	0.01862	0.03702	1.1568	0.7171	-0.4798	down	LevelBi	-	227.1744	2.3602
4-[(2,4-Dihydroxy-3,3-Dimethylbutanoyl)Amino]Butanoic Acid	0.01576	0.03219	1.1561	1.5849	0.6644	up	LevelBii	HMDB0246316	254.1	3.4894
9-Cis-Retinol	0.002434	0.008084	1.1559	0.7913	-0.3377	down	LevelBi	HMDB0006217;LMPR01090009;PW_C002666	287.2342	6.447
Thymine	2.154E-5	0.0002146	1.1554	0.7297	-0.4547	down	LevelBi	HMDB0000262;HMDB0010337;HMDB0000262;PW_C000179	127.0491	1.9821
Thr-Lys	3.015E-5	0.0002761	1.1552	0.7924	-0.3357	down	LevelBii	HMDB0029066	270.1432	1.9821
3,5-Pyridinedicarboxylic Acid, 1,4-Dihydro-2,4,6-Trimethyl-, Diethyl Ester	0.0001657	0.0009954	1.1541	1.5862	0.6656	up	LevelBii	HMDB0247156	533.285	3.9438
Cdp-Dg(5-Iso Pgf2Vi/22:3(10Z,13Z,16Z))	0.04049	0.06683	1.1539	0.7148	-0.4845	down	LevelBii	HMDB0292368	1116.5494	6.5251
1-Ethyl-2-[(E)-(1-Ethyl-2(1H)-Quinolinylidene)Methyl]Quinolinium	0.002241	0.007645	1.1536	0.7472	-0.4205	down	LevelBi	-	369.2229	5.2342
Tyrphostin B42	0.007224	0.01677	1.1533	1.4832	0.5687	up	LevelBi	HMDB0244549	312.1325	4.8049
Ser-Gln-Asp-Glu	0.0002016	0.001159	1.1521	1.3195	0.4	up	LevelBi	-	476.1609	1.8366
10-Nitrolinoleic Acid	0.0227	0.04354	1.1521	2.3708	1.2454	up	LevelBii	HMDB0005049	370.2216	4.7704
Val-Gly	0.0007093	0.003114	1.1487	0.727	-0.46	down	LevelBi	HMDB0259740	197.0886	1.2846
Paeonol	0.0002731	0.001476	1.1481	0.737	-0.4402	down	LevelBii	HMDB0256081	225.0755	3.4613
Trans-2-Hydroxycinnamic Acid	6.4E-5	0.0004889	1.148	1.2042	0.268	up	LevelBi	HMDB0002641	165.0538	1.8436
Carbenicillin Indanyl	0.001185	0.004631	1.1477	1.4077	0.4934	up	LevelBii	HMDB0249675	553.1637	2.9602
Quaternary Ammonium Chloride Combination	8.319E-5	0.0005911	1.1475	0.7445	-0.4256	down	LevelBii	HMDB0303284	229.0987	3.1301
Uridine 5'-Monophosphate	0.0003118	0.001645	1.1472	1.4316	0.5176	up	LevelBii	HMDB0000288	323.0275	1.6973
Elvitegravir	3.474E-5	0.000311	1.1465	0.6681	-0.5819	down	LevelBii	HMDB0251760	492.1189	2.0063
(5Alpha,6Beta,14Alpha,20R,22R)-5,6,14,20,27-Pentahydroxy-1-Oxowith-24-Enolide	0.00603	0.01442	1.1463	1.3993	0.4847	up	LevelBii	HMDB0033198	471.2786	3.5177
Alfentanil	0.04186	0.06869	1.1459	0.2923	-1.7743	down	LevelBii	HMDB0014940	451.2264	7.6016
Asp-Asp-Ile	1.772E-5	0.0001871	1.145	1.2869	0.3638	up	LevelBi	-	362.1541	2.3104
Fahfa(18:0/14:1)	0.03205	0.05725	1.142	0.6577	-0.6045	down	LevelBi	-	529.421	8.6411
9S,11R-Dihydroxy-15-Oxo-13E-Prostenoic Acid	0.003898	0.009986	1.1419	0.6941	-0.5268	down	LevelBi	-	377.2276	6.1235

Delta-Decalactone	0.002962	0.008669	1.1411	0.7548	-0.4058	down	LevelBi	HMDB0037116;LMFA07040028	171.1369	7.7446
Trimethylamine N-Oxide	2.305E-6	4.402E-5	1.1409	0.7905	-0.3391	down	LevelBi	HMDB0000925	58.0652	0.6983
Lpc(18:1)	0.00501	0.01234	1.1406	1.1881	0.2486	up	LevelBi	HMDB0002815	566.3439	8.0526
4-(Dimethylamino)Azobenzene N-Oxide	0.002991	0.008669	1.1406	1.2666	0.3409	up	LevelBi	-	281.0942	3.2322
Biliverdin	1.067E-6	2.577E-5	1.1403	1.3846	0.4694	up	LevelBii	HMDB0001008	641.2635	3.0167
Streptozocin	0.0001304	0.0008302	1.1356	0.7022	-0.51	down	LevelBii	HMDB0014572	264.0855	3.675
Monotropein	0.0001081	0.0007234	1.1349	1.4049	0.4905	up	LevelBii	HMDB0035608	435.111	2.0252
Cyclosquamosin F	0.009028	0.02019	1.1348	0.6859	-0.5439	down	LevelBii	HMDB0303371	819.3969	6.3694
Phenmedipham	1.19E-5	0.0001406	1.1339	0.7118	-0.4904	down	LevelBii	HMDB0256405	345.1068	2.9602
Hexanoyl-L-Carnitine	6.753E-5	0.0005099	1.1333	1.2346	0.304	up	LevelBi	-	260.1853	3.2517
Convallatoxin	0.00736	0.01704	1.1325	1.3591	0.4427	up	LevelBi	HMDB0034362;LMST01120013	551.2821	2.6865
5-[[2-(3-Ethyl-1,2-Oxazol-5-Yl)Pyrrolidin-1-Yl]Methyl]-2-Methoxyphenol	0.01909	0.03778	1.1324	1.5534	0.6355	up	LevelBi	-	303.1715	6.349
Rotundifoline	1.352E-6	3.092E-5	1.132	1.4414	0.5275	up	LevelBii	HMDB0302761	459.21	3.1772
Sotrastaurin	0.0253	0.04763	1.1318	1.468	0.5538	up	LevelBii	HMDB0258398	477.1448	5.9963
(2-((3,5-Di-T-Butyl-4-Hydroxyphenyl)Thio)-1-Methylpropoxy)Acetic Acid	0.0004109	0.002044	1.1314	0.7398	-0.4347	down	LevelBii	HMDB0242455	413.2015	2.082
Pc(Txb2/P-18:1(11Z))	0.001502	0.005598	1.1307	1.4518	0.5379	up	LevelBii	HMDB0289672	856.5653	8.2352
Taurodeoxycholic Acid 3-Glucuronide	0.01439	0.02987	1.1307	1.2751	0.3506	up	LevelBii	HMDB0240734	656.3162	7.611
Gentamicin B	0.001839	0.006563	1.1292	1.3718	0.4561	up	LevelBii	HMDB0252687	517.2238	2.3208
3,7-Dihydroxy-12-Oxocholanoic Acid	0.003494	0.009132	1.1284	0.746	-0.4227	down	LevelBii	HMDB0000400	441.2452	5.8217
Ribostamycin	0.002837	0.008669	1.1283	0.711	-0.4922	down	LevelBii	HMDB0257216	953.4543	5.6277
N-Arachidonoyl Dopamine	0.0008332	0.003529	1.1277	0.8683	-0.2038	down	LevelBi	HMDB0243624;LMFA08020084	457.3435	9.2648
Lnape(20:4/N-18:0)	0.03368	0.05735	1.1267	1.2264	0.2944	up	LevelBi	-	826.5599	8.8332
Medroxyprogesterone	0.002197	0.007531	1.1255	0.7028	-0.5089	down	LevelBii	HMDB0001939	379.2079	6.2676
Uridine 2'-Phosphate	0.001047	0.004213	1.1243	2.69	1.4276	up	LevelBii	HMDB0011641	323.027	1.1097
Ile-Trp	0.005143	0.0126	1.123	0.7941	-0.3326	down	LevelBi	HMDB0028918	318.1799	3.3405
Tricoumaroyl Spermidine	0.008773	0.01972	1.1226	0.6603	-0.5987	down	LevelBii	HMDB0304518	628.2671	1.8555
Fa(20:1)	1.44E-5	0.0001614	1.1225	0.7932	-0.3342	down	LevelBi	-	355.2835	9.4109
2-Amino-2-Methyl-1,3-Propanediol	5.903E-8	3.853E-6	1.12	1.1953	0.2574	up	LevelBi	HMDB0244975	70.0648	0.7273
Strobilurin	0.001389	0.005255	1.1197	1.3244	0.4053	up	LevelBii	HMDB0258515	441.1953	3.9344

Di-8-Anepps	0.01729	0.03479	1.1194	1.3329	0.4146	up	LevelBii	HMDB0251114	615.3645	8.115
2-(1,2,3,4-Tetrahydroxybutyl)-6-(2,3,4-Trihydroxybutyl)Pyrazine	2.088E-5	0.0002106	1.1182	0.745	-0.4247	down	LevelBii	HMDB0034894	349.1248	3.5365
L-Tyrosine	6.106E-5	0.0004722	1.1177	1.1743	0.2318	up	LevelBi	HMDB0000158;PW_C000103	182.0806	1.8436
Trans-3-Indoleacrylic Acid	0.0004125	0.002049	1.1172	1.1668	0.2225	up	LevelBi	HMDB0000734	188.0706	2.3901
Hydroxyphenyllactic Acid	2.164E-6	4.225E-5	1.1171	0.8388	-0.2535	down	LevelBi	HMDB0000755	181.0503	2.5476
(Z)-2,4-Dihydroxy-6-(8-Pentadecenyl)Benzoic Acid	0.002916	0.008669	1.1168	0.7977	-0.3261	down	LevelBii	HMDB0039226	397.2183	6.0806
Lpc(O-15:0)	0.001242	0.004813	1.1167	0.8294	-0.2699	down	LevelBi	LMGP01060009	512.3329	8.0341
Salmeterol	0.003039	0.008669	1.1164	0.7192	-0.4756	down	LevelBi	HMDB0015073	414.2692	2.1297
Flumethasone	2.391E-6	4.536E-5	1.1155	1.3022	0.381	up	LevelBii	HMDB0252336	455.1908	1.9969
Diferuloylputrescine	8.676E-6	0.000112	1.1135	0.8484	-0.2371	down	LevelBii	HMDB0033468	439.1904	3.6111
3-Cyclopentylalanine	0.0147	0.03039	1.1134	0.5928	-0.7544	down	LevelBi	-	199.1431	3.321
Atorvastatin	0.0004182	0.002067	1.1133	1.3173	0.3976	up	LevelBii	HMDB0005006	539.232	4.1298
13-Hydroxy-5'-O-Methylmelledonal	0.01239	0.02637	1.1132	1.5652	0.6463	up	LevelBii	HMDB0035864	443.1675	2.2353
Odalasvir	0.017	0.03428	1.112	1.6343	0.7087	up	LevelBii	HMDB0255919	999.5436	7.8296
20-Hydroxy-Leukotriene E4	0.008158	0.01855	1.1108	0.6815	-0.5532	down	LevelBii	HMDB0012639	454.227	3.8598
Cinncassiol D4 2-Glucoside	0.004599	0.01149	1.1094	0.806	-0.3112	down	LevelBii	HMDB0035065	559.2727	4.8811
Buntanine	1.827E-5	0.0001916	1.1094	1.252	0.3242	up	LevelBii	HMDB0038590	390.1136	1.8931
1-Isothiocyanato-8-(Methylthio)Octane	0.0001499	0.000923	1.1078	0.7223	-0.4693	down	LevelBii	HMDB0038447	216.0865	2.642
(R)-2-Nitro-P-Mentha-1,5-Diene	0.000102	0.0006898	1.107	0.8039	-0.315	down	LevelBii	HMDB0036090	199.1432	2.0017
Delta-Hydroxylysylnorleucine	0.0001363	0.0008571	1.1066	0.8093	-0.3052	down	LevelBii	HMDB0253190	274.1762	1.9919
(2E,4E)-Octadeca-2,4-Dienoylcarnitine	0.006158	0.01466	1.1056	1.5604	0.6419	up	LevelBii	HMDB0241541	468.33	7.0347
Val-Pro	5.414E-8	3.633E-6	1.1047	0.8722	-0.1972	down	LevelBi	HMDB0029135	215.1386	2.1012
2-[Methyl(3-Phenylpropanoyl)Amino]Benzoic Acid	0.0003323	0.001728	1.1034	0.6983	-0.5182	down	LevelBii	HMDB0032845	342.1322	4.1484
N-Methyl(4-Pyrimidinyl)Methanamine	3.072E-6	5.439E-5	1.1025	1.2506	0.3226	up	LevelBi	-	124.0863	0.679
St(24:1_O4_T/17:1)	0.03534	0.05969	1.1018	1.2906	0.3681	up	LevelBi	-	808.5466	8.5476
N-Lactoyl-Tryptophan	0.0005763	0.002649	1.1018	0.7684	-0.3801	down	LevelBii	HMDB0062178	297.0842	2.979
Pro-Pro-Phe	2.269E-5	0.0002229	1.1005	0.8275	-0.2731	down	LevelBii	HMDB0304811	358.1777	3.0733
Ps(6 Keto-Pgf1Alpha/22:4(7Z,10Z,13Z,16Z))	0.009011	0.02016	1.1002	0.6623	-0.5945	down	LevelBii	HMDB0283217	946.4974	5.0105
N-Cyclopropyl-4-[[1-(3,4-Dimethylbenzoyl)Piperidin-4-Yl]Methoxy]Benzamide	0.01316	0.02772	1.0994	0.78	-0.3585	down	LevelBi	-	407.2367	2.4872

2-Arachidonoylglycerol	0.03249	0.05725	1.098	1.2993	0.3777	up	LevelBi	HMDB0004666	379.2825	8.3007
3-(3-Hydroxydecanoyloxy)Decanoic Acid	0.00747	0.01724	1.0965	0.7782	-0.3618	down	LevelBi	-	381.2588	6.729
1-(2-Amino-3-Phenylpropanoyl)Proline	8.238E-6	0.0001085	1.0958	0.8644	-0.2102	down	LevelBi	-	263.1386	2.4582
Pi(Pgf2Alpha/20:0)	0.04888	0.07827	1.095	1.7668	0.8211	up	LevelBii	HMDB0276994	997.6175	8.1832
Phe-Ile-Ser	0.009579	0.02126	1.0949	1.3146	0.3946	up	LevelBi	-	366.2013	2.4775
Fa(18:1)	3.854E-5	0.0003369	1.0946	0.8576	-0.2216	down	LevelBi	-	563.5015	9.0755
Fahfa(20:3/22:5)	9.966E-5	0.000677	1.0933	0.8139	-0.2972	down	LevelBi	-	633.4836	8.88
12,13-Epome	0.007952	0.01815	1.0888	0.8228	-0.2814	down	LevelBi	HMDB0004702	297.2411	7.8121
Amobarbital	1.342E-5	0.0001537	1.0878	0.8057	-0.3117	down	LevelBii	HMDB0015440	227.1379	2.9249
Cys-Phe	0.01939	0.03827	1.0875	1.3219	0.4026	up	LevelBi	HMDB0028782	269.0939	2.4775
P-Tolyl Sulfate	0.001218	0.004743	1.0873	1.6186	0.6947	up	LevelBi	-	187.006	3.6936
Carnitine	5.636E-9	8.175E-7	1.0848	1.1511	0.203	up	LevelBi	-	162.1121	0.6983
8-(3-Oxo-2-(Pent-2-Enyl) Cyclopentenyl)Octanoic Acid	0.001938	0.00683	1.0836	0.793	-0.3346	down	LevelBi	-	293.2096	5.7323
Biotin	1.159E-5	0.0001381	1.0833	0.5374	-0.896	down	LevelBii	HMDB0000030	533.1713	1.9969
1-Hexadecyl-1H-Indole-2,3-Dione	5.855E-5	0.0004571	1.082	0.8822	-0.1809	down	LevelBi	-	372.2919	9.2841
Gly-Lys-Phe	0.03543	0.05982	1.0815	0.6494	-0.6229	down	LevelBi	-	351.2026	1.9026
Spectinomycin	0.01448	0.03003	1.0812	0.5565	-0.8455	down	LevelBii	HMDB0015055	365.1944	2.4
Hyp-Pro	0.002733	0.008669	1.0811	0.6468	-0.6287	down	LevelBii	HMDB0028871	227.1018	2.6609
Capsoside A	0.04614	0.07454	1.0806	0.6912	-0.5328	down	LevelBii	HMDB0037613	659.3593	2.2111
Asparagine Ethyl Ester	0.00152	0.005651	1.0793	1.4211	0.507	up	LevelBi	-	215.1015	1.9623
Gly-Pro	0.000279	0.001502	1.0793	0.7659	-0.3847	down	LevelBi	HMDB0252876	155.0814	2.0512
3-Dehydro-6-Deoxoteasterone	0.002128	0.007346	1.0787	0.777	-0.3639	down	LevelBii	HMDB0304120	467.3285	9.2145
Pc(O-16:1/18:2)	0.009316	0.02078	1.0786	1.1688	0.225	up	LevelBi	LMGP01020257	786.5632	9.4482
N-Myristoyl Methionine	0.03864	0.06427	1.0765	1.2824	0.3589	up	LevelBii	HMDB0242056	360.2579	8.3772
Ps(Pgf2Alpha/16:1(9Z))	0.01837	0.03666	1.0747	1.3989	0.4843	up	LevelBii	HMDB0281245	814.485	7.6392
Garcimangosone C	4.481E-6	7.018E-5	1.0742	1.2998	0.3782	up	LevelBii	HMDB0036984	471.1686	2.5194
Pc(Pgf1Alpha/P-18:1(9Z))	0.0295	0.05393	1.073	0.6695	-0.5788	down	LevelBii	HMDB0289834	842.5929	7.8481
Lpc(20:4-Sn1)	0.01479	0.03056	1.072	1.1771	0.2352	up	LevelBi	-	544.3365	7.6294
Sweroside	0.0003796	0.001923	1.0709	1.5442	0.6269	up	LevelBii	HMDB0258643	417.1418	2.6326

N-Acetyl-B-Glucosaminylamine	1.31E -5	0.000 1509	1.0697	1.446 5	0.5326	up	Leve lBii	HMDB0001104	265.1 054	4.5832
Thr-Met	0.035 33	0.059 67	1.0691	1.597 3	0.6756	up	Leve lBii	HMDB0029067	249.0 909	1.9214
Crenolanib	0.008 855	0.019 87	1.0676	0.741 9	-0.4307	dow n	Leve lBii	HMDB0250534	442.2 288	2.3777
Tamsulosin	1.757 E-5	0.000 1856	1.0675	1.186	0.2461	up	Leve lBi	HMDB0014844;HMDB0259951	409.1 811	3.133
2',6'-Dihydroxy-4'-Methoxyacetophenone	0.006 527	0.015 41	1.0649	2.864 4	1.5182	up	Leve lBii	HMDB0029646	181.0 497	4.8811
Xi-7-Hydroxyhexadecanedioic Acid	0.003 573	0.009 3	1.0645	0.781 8	-0.3552	dow n	Leve lBii	HMDB0037830	301.2 006	5.7845
Chitotriose	0.004 079	0.010 38	1.0644	1.295	0.373	up	Leve lBii	HMDB0006578	560.2 301	2.3398
Pyroglutamylthreonine	1.345 E-6	3.09E -5	1.0642	0.769 1	-0.3787	dow n	Leve lBii	HMDB0341387	211.0 713	2.1297
Met-Val-Phe	0.005 804	0.013 97	1.0622	0.712 2	-0.4897	dow n	Leve lBi	-	396.1 931	3.5276
Trans-Hex-2-Enoic Acid	0.002 023	0.007 066	1.0595	0.804 8	-0.3133	dow n	Leve lBii	HMDB0010719	113.0 598	3.6111
Phe-Cys	0.005 424	0.013 19	1.0586	1.314 4	0.3944	up	Leve lBi	HMDB0028992	323.1 043	4.8829
1-Ethylpyrrolidine	1.506 E-6	3.328 E-5	1.0581	0.820 1	-0.2861	dow n	Leve lBii	HMDB0243879	122.0 934	2.8754
Phosphoribosyl Formamidocarboxamide	0.000 8434	0.003 561	1.0573	1.331 9	0.4134	up	Leve lBii	HMDB0001439	365.0 474	1.9969
1,2-Dioctanoylglycerol	0.030 68	0.055 71	1.0568	0.664 9	-0.5888	dow n	Leve lBii	HMDB0244086	403.2 683	6.7394
2-[3-(4-Phenylpiperazino)Propoxy]Benzamide	0.027 48	0.050 95	1.0566	1.562 2	0.6436	up	Leve lBi	-	322.1 903	4.6091
Nummularine A	0.047 53	0.076 42	1.0562	1.606 9	0.6842	up	Leve lBii	HMDB0029336	682.3 32	7.894
Pilocarpine	0.000 7086	0.003 112	1.056	0.777 8	-0.3626	dow n	Leve lBi	HMDB0015217	226.1 547	1.9124
Kaempferol-7-O-Glucoside	0.049 45	0.078 98	1.0551	0.755 3	-0.4048	dow n	Leve lBi	-	447.0 909	4.1669
Phe-Ser-Ser	0.003 579	0.009 311	1.0551	1.386 2	0.4712	up	Leve lBi	-	340.1 476	2.3302
3A,6B,7A,12A-Tetrahydroxy-5B-Cholanoic Acid	0.036 05	0.060 62	1.0541	0.689 2	-0.5371	dow n	Leve lBii	HMDB0000399	447.2 718	6.6123
Gibberellin A28	0.001 584	0.005 824	1.0538	0.702 9	-0.5086	dow n	Leve lBii	HMDB0303573	375.1 415	3.48
Ala-Ile	0.003 643	0.009 437	1.0533	1.210 7	0.2759	up	Leve lBii	HMDB0028690	201.1 237	2.1785
Vellein	0.000 5996	0.002 738	1.0526	0.723	-0.4678	dow n	Leve lBii	HMDB0303360	413.1 191	3.6936
(E,E)-1,7-Diphenyl-4,6-Heptadien-3-Ol	0.002 938	0.008 669	1.0516	0.698	-0.5187	dow n	Leve lBii	HMDB0040893	299.1 215	1.24
Ascofuranone	0.000 1813	0.001 068	1.05	0.786 8	-0.346	dow n	Leve lBii	HMDB0248651	385.1 586	4.0796
Estragonoside	0.012 09	0.025 86	1.0498	0.640 6	-0.6426	dow n	Leve lBii	HMDB0033650	499.0 821	4.1298
2-Hydroxyacetophenone	3.133 E-6	5.507 E-5	1.0473	0.812 9	-0.2989	dow n	Leve lBii	HMDB0245150	135.0 443	2.5476
Cholylglutamine	0.012 02	0.025 75	1.0467	1.235 7	0.3054	up	Leve lBii	HMDB0242387	557.3 183	8.0799
Araloside A	0.038 9	0.064 62	1.0463	0.801 9	-0.3186	dow n	Leve lBii	HMDB0034535	961.4 617	6.9054

Camphorsulfonic Acid	0.001012	0.00411	1.0457	1.3321	0.4137	up	LevelBii	HMDB0243496	267.0448	3.5457
Mibefradil	0.0185	0.03686	1.0455	1.2362	0.3059	up	LevelBii	HMDB0254702	500.2718	7.8512
Pc(Pgf2Alpha/P-18:0)	0.02589	0.0485	1.0455	0.774	-0.3697	down	LevelBii	HMDB0289560	844.5993	8.1635
Tunicamycin A	0.03106	0.05628	1.0449	0.733	-0.4481	down	LevelBii	HMDB0259327	851.3886	5.5541
Phe-His-Glu	1.754E-7	8.111E-6	1.0434	1.2143	0.2802	up	LevelBi	-	432.188	2.0614
Gln-Val-Ile	0.03634	0.06103	1.0428	1.499	0.584	up	LevelBi	-	359.2274	2.6665
5-O-Desmethyldonepezil	0.01947	0.03841	1.0424	0.0607	-4.0426	down	LevelBii	HMDB0013958	346.1786	3.2053
Tyr-Gly-Gly-Trp-Leu	4.424E-5	0.0003757	1.0421	1.2783	0.3542	up	LevelBii	HMDB0259342	615.2601	3.48
Val-Pro-Gly-Val-Gly	0.003067	0.008669	1.0421	0.7819	-0.355	down	LevelBii	HMDB0259877	472.239	2.3114
11-Hydroxyprogesterone 11-Glucuronide	0.01238	0.02637	1.0415	0.5265	-0.9255	down	LevelBii	HMDB0010364	487.2388	3.4048
(Z)-15-Oxo-11-Eicosenoic Acid	0.01697	0.03425	1.0375	0.7108	-0.4924	down	LevelBii	HMDB0029797	323.2576	8.5568
Ile-Ile-Thr	0.003701	0.009558	1.0365	0.8168	-0.292	down	LevelBi	-	346.2315	2.35
Tyr-Lys	0.01394	0.02907	1.0361	1.3231	0.404	up	LevelBi	HMDB0029110	310.1754	1.0591
Ile-Gln-Tyr	0.02477	0.04682	1.0346	0.7595	-0.3969	down	LevelBi	-	423.2229	2.537
Queuine	5.401E-9	8.147E-7	1.0329	1.201	0.2643	up	LevelBii	HMDB0001495	278.1223	1.02
2,3-Dihydroxycarbamazepine	0.0007455	0.003231	1.031	0.6942	-0.5266	down	LevelBii	HMDB0060673	327.0997	3.4048
Mespirenone	0.0135	0.02833	1.0308	1.2888	0.366	up	LevelBii	HMDB0254483	461.1567	6.5913
N-Arachidonoyl Gaba	4.004E-5	0.0003472	1.0302	0.891	-0.1665	down	LevelBii	HMDB0062330	372.293	9.1381
3,7-Dihydroxy-25-Methoxycucurbita-5,23-Dien-19-Al	4.386E-5	0.0003738	1.0293	0.7928	-0.335	down	LevelBii	HMDB0039362	491.3547	7.3049
4-Acetylbutyrate	0.0003666	0.00187	1.0289	1.2541	0.3266	up	LevelBii	HMDB0061881	189.0756	3.4425
2-Ethyl-2-Hydroxybutyric Acid	9.098E-6	0.0001155	1.0284	0.8904	-0.1674	down	LevelBi	HMDB0001975	131.0711	3.6111
Gly-Leu	0.04659	0.07514	1.0277	1.2882	0.3653	up	LevelBi	HMDB0000759	189.1228	2.1912
Irbesartan	4.357E-5	0.0003716	1.0275	0.8081	-0.3073	down	LevelBi	HMDB0015163;PW_C009460	427.2218	8.6225
1-Aminocyclopropanecarboxylic Acid	2.074E-5	0.0002097	1.0273	0.8162	-0.2929	down	LevelBi	HMDB0036458	102.0541	1.148
Nigellicine	0.01215	0.02596	1.0262	0.7092	-0.4956	down	LevelBii	HMDB0030269	247.1095	2.8159
4-Hydroxyretinoic Acid Glucuronide	0.000297	0.001579	1.0259	0.8124	-0.2998	down	LevelBii	HMDB0061688	493.2417	2.5069
Gamma-L-Glutamyl-L-Pipecolic Acid	2.93E-5	0.0002701	1.0246	0.7074	-0.4994	down	LevelBii	HMDB0038614	317.1337	3.1018
4-O-Methyl-Myo-Inositol	0.008229	0.01867	1.0229	0.6293	-0.6681	down	LevelBii	HMDB0029915	215.0527	1.9024
Fahfa(18:0/20:0)	0.000	0.003	1.0218	0.793	-0.3332	dow	Leve	-	593.5	9.4015

	6894	05		8		n	lBi		485	
N1-(2-Piperidinophenyl)-3,4,5-Trimethoxybenzamide	0.005439	0.01322	1.0218	1.3503	0.4333	up	LevellBi	-	388.2248	5.1367
Thr-Tyr	0.008165	0.01856	1.0217	1.3791	0.4638	up	LevellBii	HMDB0029073	303.0949	2.3495
1-Hydroxyhexanoylglycine	9.684E-6	0.0001204	1.0214	0.8089	-0.306	down	LevellBii	HMDB0094718	170.0814	3.273
Occidentoside	0.04128	0.06793	1.0212	2.4774	1.3088	up	LevellBii	HMDB0030574	705.1794	1.0886
Dibenzepin	0.006304	0.01496	1.0201	0.7405	-0.4335	down	LevellBii	HMDB0251161	635.3319	7.3405
Patuletin 7-Glucoside	0.02554	0.04798	1.0194	0.7077	-0.4989	down	LevellBii	HMDB0303788	515.0775	4.1669
Lpc(22:4)	0.01191	0.02555	1.0163	1.2205	0.2874	up	LevellBi	HMDB0010401	616.3593	8.0341
Isoeruboside B	0.03654	0.06132	1.0146	0.8532	-0.2291	down	LevellBii	HMDB0029769	1125.5302	7.044
Dehydroascorbic Acid	3.597E-6	6.069E-5	1.0123	1.1981	0.2608	up	LevellBii	HMDB0001264	173.0075	0.7458
Pa(16:3/16:4)	0.02737	0.0508	1.0113	0.6881	-0.5393	down	LevellBi	-	679.3652	5.4988
Linoelaidic Acid	0.0103	0.02262	1.0103	0.8315	-0.2662	down	LevellBi	HMDB0006270;LMFA01030123	281.2457	8.7745
Pc-M6	0.002487	0.008217	1.0103	0.3242	-1.6252	down	LevellBii	HMDB0038568	402.247	5.0474
(E,E)-Futoamide	8.671E-6	0.000112	1.0094	0.8279	-0.2725	down	LevellBii	HMDB0032654	322.1397	2.2353
Deacetylchloronectrin	2.356E-6	4.482E-5	1.0091	0.8607	-0.2165	down	LevellBi	-	423.1967	2.0512
His-Asp-Leu	0.004968	0.01225	1.0088	1.2969	0.375	up	LevellBi	-	384.1859	1.0788
Scillipheosidin 3-[Glucosyl-(1->2)-Rhamnoside]	0.002181	0.007489	1.0072	1.248	0.3196	up	LevellBii	HMDB0036946	743.3083	4.3522
Dienogest	0.002419	0.008065	1.0064	1.3453	0.4279	up	LevellBii	HMDB0251234	346.1598	2.5383
Ethyl 1-Boc-3-Allylpiperidine-3-Carboxylate	0.001868	0.006638	1.0064	1.2981	0.3764	up	LevellBi	-	330.2279	4.0993
Oxoglutaric Acid	3.026E-6	5.379E-5	1.0053	1.1954	0.2575	up	LevellBii	HMDB0000208	205.0345	2.1593
Carnosic Acid Diacetate Methyl Ester	0.0004915	0.002339	1.0046	1.2802	0.3563	up	LevellBi	-	431.2397	2.3602
Dimethisterone	0.0001584	0.0009632	1.0033	0.8818	-0.1814	down	LevellBii	HMDB0251378	358.2762	8.3772
Phe-Ser	0.0109	0.02369	1.0031	1.2448	0.3159	up	LevellBi	HMDB0029004	253.1186	2.0812
Histidyltryptophan	7.758E-6	0.000105	1.0019	1.2609	0.3345	up	LevellBii	HMDB0028896	376.1159	2.5853
1-O-Hexadecyl-Sn-Glycero-3-Phosphocholine	0.0002885	0.001542	1.0009	0.9138	-0.1301	down	LevellBii	HMDB0243890	482.3586	8.3392
Apramycin	0.0262	0.04895	1.0008	0.6696	-0.5787	down	LevellBii	HMDB0248529	572.3108	2.6267

Table S4 Statistics of Differential Metabolites between YNSM and LSM

Metabolite	P_value	FDR	VIP_PLS-DA	FC(D/E)	Log2FC(D/E)	Regulate	level	Library ID	M/Z	Retention time	Adducts
8-Deoxy-11-Hydroxy-13-Chlorogrosheimin	8.316E-10	6.453E-8	2.5658	0.0139	-6.1678	down	LevelBii	HMDB0041037	357.114	2.8403	M+Hac-H
Inosinic Acid	7.785E-12	4.3E-9	2.4482	4387.5743	12.0992	up	LevelBi	HMDB0000175;HMDB0257686;PW_C000114	349.0531	1.4865	M+H
Lyciumoside Iv	3.991E-11	1.208E-8	2.3842	0.0021	-8.909	down	LevelBii	HMDB0033499	821.415	5.462	M+FA-H
Poncirin	5.978E-9	2.388E-7	2.3805	5.9424	2.5711	up	LevelBii	HMDB0037487	593.1916	0.9324	M-H
Sucrose	1.812E-8	5.214E-7	2.3783	24.0719	4.5893	up	LevelBi	HMDB0000258;PW_C000177	381.0771	0.6983	M+K
Pi(13:0/16:4)	2.272E-5	0.0001251	2.3699	0.1381	-2.8558	down	LevelBi	-	805.4202	6.4339	M+FA-H
Meticillin	2.298E-7	3.438E-6	2.3652	0.1157	-3.1122	down	LevelBii	HMDB0015541	379.0959	2.8775	M-H
Pinolidoxin	1.093E-8	3.608E-7	2.3627	0	-15.395	down	LevelBii	HMDB0301836	359.1483	4.0277	M+Na-2H
17-Hydroxy-15-Kauren-19-Oic Acid	7.707E-8	1.505E-6	2.3472	0.0061	-7.3484	down	LevelBi	-	301.2143	5.8592	M+H-H2O
Manninotriose	2.77E-8	7.115E-7	2.3433	10.824	3.4362	up	LevelBi	HMDB0060468	487.1624	1.0985	M+H-H2O
Protoporphyrin Ix	1.368E-6	1.336E-5	2.3336	0.0381	-4.7156	down	LevelBii	HMDB0000241	1123.5138	6.8225	2M-H
Emodin	2.629E-6	2.227E-5	2.3321	0.0128	-6.286	down	LevelBi	HMDB0035214;LMPK13040008	269.0449	5.2503	M-H
Tenatoprazole	2.852E-9	1.393E-7	2.313	0.041	-4.6066	down	LevelBii	HMDB0258812	737.2202	2.8683	2M+FA-H
Leukotriene D4	8.249E-9	2.977E-7	2.2896	0.0015	-9.3444	down	LevelBii	HMDB0003080	541.2626	5.2412	M+FA-H
17(18)-Epoxy-5Z,8Z,11Z,14Z-Eicosatetraenoic Acid	9.783E-8	1.821E-6	2.2513	0.0558	-4.1648	down	LevelBi	HMDB0010212	319.2255	5.8592	M+H

9-((2-Hydroxyethoxy)Methyl)Hypoxanthine	3.736E-10	4.192E-8	2.2435	0.0204	-5.6128	down	LevelBi	-	211.0819	1.9821	M+H
Cyclohexylsulfamate	1.239E-10	2.253E-8	2.2367	0.3578	-1.4829	down	LevelBi	HMDB0031340	178.0541	2.8592	M-H
Gibberellin A3	2.683E-10	3.472E-8	2.229	0.0137	-6.1921	down	LevelBii	HMDB0003559	391.1368	3.6382	M+FA-H
3-Dehydrosphinganine (C20)	1.096E-7	1.983E-6	2.2118	0.0947	-3.4006	down	LevelBii	HMDB0304123	383.3397	7.4819	M+CH3OH+Na
Norcisapride	2.547E-8	6.76E-7	2.207	0.1083	-3.2072	down	LevelBii	HMDB0061018	671.2319	3.9904	2M+FA-H
Isovitexin 2''-O-(6'''-Feruloyl)Glucoside	6.711E-6	4.695E-5	2.1981	0.0911	-3.4565	down	LevelBii	HMDB0038042	769.1958	4.139	M-H
Val-Val-Pro	3.175E-9	1.52E-7	2.1734	0.0006	-10.7354	down	LevelBi	-	314.2058	2.2012	M+H
4-Demethylsimmondsin 2'-(E)-Ferulate	1.178E-7	2.071E-6	2.1726	0.1358	-2.8809	down	LevelBii	HMDB0034773	558.1572	2.8683	M+Na-2H
1-Ribityl-6,7-Dimethyl-1,2,3,4-Tetrahydroquinoxalin-2,3-Dione	1.219E-10	2.245E-8	2.167	0.0158	-5.9875	down	LevelBi	-	325.1378	3.133	M+H
9Z,11E,13E-Octadecatrienoic Acid Ethyl Ester	5.427E-5	0.0001955	2.1682	0.0774	-3.6922	down	LevelBi	-	307.2617	8.1733	M+H
1,2,3,4-Tetrahydroisoquinoline-3-Carboxylic Acid	1.041E-7	1.91E-6	2.1609	0.0764	-3.7106	down	LevelBii	HMDB0244117	236.0917	3.7311	M+Hac-H
2Alpha-Methyl-5Alpha-Androstan-3Alpha-Ol-17-One	2.011E-6	1.806E-5	2.164	0.0492	-4.3453	down	LevelBi	-	269.2252	6.4371	M+H-2H2O
Malvidin 3-(6''-Acetyl-Galactoside)	7.531E-10	6.073E-8	2.1594	0.0996	-3.3275	down	LevelBii	HMDB0301662	580.1383	2.8865	M+FA-H
Leucrose	1.727E-7	2.771E-6	2.1567	10.5321	3.3967	up	LevelBi	-	360.148	0.8735	M+NH4
N-(1-Naphthyl)Ethylenediamine	4.64E-5	0.0001734	2.141	0.1482	-2.7548	down	LevelBi	HMDB0254982	187.1221	3.0142	M+H
(7'R,8'R)-4,7'-Epoxy-3',5-Dimethoxy-4',9,9'-Lignanetriol 9'-Glucoside	1.638E-5	9.58E-5	2.1418	0.117	-3.0957	down	LevelBi	HMDB0038711	521.2002	3.4425	M-H

							i				
Jujuboside B	4.98E-6	3.682E-5	2.1452	0.1495	-2.7421	down	LevelBii	HMDB0035745	1079.5244	7.5737	M+Cl
Cinncassiol D4 2-Glucoside	1.475E-9	9.37E-8	2.1338	0.1173	-3.0923	down	LevelBii	HMDB0035065	559.2727	4.8811	M+FA-H
Fumonisin A1	3.864E-6	3.008E-5	2.1302	0.0245	-5.3537	down	LevelBii	HMDB0034713	822.4102	6.4339	M+Hac-H
Chenodeoxycholyltryptophan	0.0001003	0.000333	2.1279	0.132	-2.9212	down	LevelBii	HMDB0242395	633.3839	6.3883	M+CH3OH+Na
Apigenin	0.000105	0.0003459	2.115	0.0673	-3.8927	down	LevelBi	HMDB0002124;LMPK12110005	271.0596	5.2537	M+H
Pip(Pgf1Alpha/16:2(9Z,12Z))	6.735E-6	4.701E-5	2.1024	0.1622	-2.6237	down	LevelBii	HMDB0278720	1031.466	7.1273	M+Na-2H, M+Cl, M+FA-H
Gm3(30:2_2O)	3.621E-5	0.000173	2.1009	0.1109	-3.1721	down	LevelBi	-	1139.6317	7.0625	M+FA-H
Picrocrocin	3.166E-8	7.825E-7	2.0956	0.0027	-8.5105	down	LevelBii	HMDB0005796	705.3302	5.7015	2M+FA-H
Phaseollinisoflavan	7.886E-7	8.666E-6	2.0958	0.0426	-4.5541	down	LevelBii	HMDB0034411	325.142	4.0206	M+H
S-(Formylmethyl)Glutathione	1.695E-12	1.623E-9	2.0907	0.112	-3.1587	down	LevelBii	HMDB0060507	314.0829	1.6103	M+H-2H2O
Rosuvastatin 5 S-Lactone	2.057E-6	1.836E-5	2.092	8.3598	3.0635	up	LevelBii	HMDB0060941	508.1532	0.727	M+FA-H
Loquatifolin A	1.946E-6	1.76E-5	2.0912	0.1445	-2.7907	down	LevelBii	HMDB0030434	821.4139	6.0435	M-H
Phenylacetylglutamine	1.386E-10	2.369E-8	2.0799	0.0583	-4.1002	down	LevelBii	HMDB0006344	323.1235	3.1301	M+Hac-H
Cellopentaose	1.061E-10	2.117E-8	2.0773	7.1212	2.8321	up	LevelBii	HMDB0249794	863.2424	1.1003	M+Cl
Gitonin	2.48	0.00	2.0663	0.20	-2.259	dow	Lev	HMDB0301779	1109	7.5177	M+Hac-H

	5E-5	01345		88	9	n	elBii		.5349		
Clindamycin	6.67E-11	1.57E-8	2.0614	0.135	-2.889	down	LevelBii	HMDB0015321	425.1893	2.915	M+H
Dihydrocapsaicin	0.0002861	0.0008181	2.0549	0.2497	-2.0017	down	LevelBi	HMDB0038457;LMFA08020318	308.2215	6.7388	M+H
Gingerglycolipid B	0.0002543	0.0007383	2.0577	0.1767	-2.5009	down	LevelBii	HMDB0041094	659.3616	6.4618	M-H2O-H
Pyridoxine	8.078E-8	1.559E-6	2.0502	0.1688	-2.5666	down	LevelBi	HMDB0000239;PW_C000161	170.0797	1.4959	M+H
Hypoglycin B	6.405E-7	7.375E-6	2.0473	0.1081	-3.2099	down	LevelBii	HMDB0029428	309.0856	4.7167	M+K
Protoporphyrin	2.549E-6	2.174E-5	2.0433	0.1773	-2.4957	down	LevelBi	HMDB0000241	563.2614	8.5328	M+H
Cys-Trp-Ala	4.165E-10	4.424E-8	2.0405	0.0052	-7.5942	down	LevelBii	HMDB0341149	437.1536	2.9509	M+Hac-H
Flavone Base+3O,C-Hex-Feruloylhex	3.554E-6	2.818E-5	2.0425	0.1893	-2.401	down	LevelBi	-	771.2079	4.1388	M+H
Isomaltotriose	2.295E-7	3.437E-6	2.0342	4.6866	2.2286	up	LevelBii	HMDB0341571	503.1597	1.0818	M-H
Milbemycin Beta1	6.835E-6	4.758E-5	2.0361	0	-14.6744	down	LevelBii	HMDB0254727	608.3514	6.7388	M+ACN+Na
Fluorescein	9.571E-9	3.304E-7	2.0321	39.3631	5.2988	up	LevelBii	HMDB0252365	393.1011	1.9876	M+Hac-H
11Beta-Hydroxytestosterone	4.496E-8	1.02E-6	2.0302	0.0633	-3.9827	down	LevelBii	HMDB0012533	349.1997	5.8494	M+FA-H
Fisetin	3.098E-5	0.0001607	2.0314	0.0617	-4.0184	down	LevelBi	LMPK12111566	285.0393	4.8996	M-H
Physcion 8-Gentiobioside	1.178E-10	2.214E-8	2.0289	4.93	2.3016	up	LevelBii	HMDB0036009	641.2091	0.8735	M+CH3OH+H
Dibekacin	2.258E-7	3.404E-6	2.0282	0.1059	-3.2398	down	LevelBii	HMDB0251157	515.2809	4.7364	M+ACN+Na

2-Pyrrolidinone	0.0001993	0.0005997	2.0192	0.1146	-3.1254	down	LevelBi	HMDB0002039	86.0598	0.6887	M+H
Glucoliquiritin Apioside	4.967E-11	1.321E-8	2.0146	4.0347	2.0125	up	LevelBii	HMDB0041149	711.2181	1.1003	M-H
Hydroxygaleon	1.542E-7	2.548E-6	2.0151	0.14	-2.8361	down	LevelBii	HMDB0031535	343.152	4.0206	M+H
Steviobioside	2.306E-6	2.012E-5	2.0057	0.046	-4.4432	down	LevelBi	HMDB0036707	641.3133	6.0435	M-H
Negletein 6-[Rhamnosyl-(1->2)-Fucoside]	0.000103	0.0003408	2.0067	4.9501	2.3075	up	LevelBii	HMDB0031991	575.1809	2.0157	M-H
Dehydroisoandrosterone 3-Glucuronide	1.874E-9	1.094E-7	1.9989	0.0672	-3.8961	down	LevelBii	HMDB0010327	497.2706	4.8829	M+CH3OH+H
N-Acetylneuraminic Acid	1.45E-5	8.694E-5	1.9897	0.1942	-2.3642	down	LevelBii	HMDB0000230	290.0868	1.6041	M-H2O-H
Dgdg(O-18:5/6:0)	2.365E-5	0.0001292	1.9933	0.0353	-4.8252	down	LevelBi	-	801.4241	7.3685	M+FA-H
Hetisine	8.654E-6	5.789E-5	1.9928	0.2715	-1.881	down	LevelBi	-	330.2035	6.7388	M+H
4'-Hydroxycarvedilol	2.316E-10	3.177E-8	1.9882	0.0173	-5.8549	down	LevelBii	HMDB0013947	403.1696	2.5571	M-H2O-H
Occidentoside	3.547E-10	4.011E-8	1.9888	6.8376	2.7735	up	LevelBii	HMDB0030574	705.1794	1.0886	M+H
N-(1-Deoxy-1-Fructosyl)Isoleucine	2.961E-5	0.0001548	1.9858	0.1707	-2.5505	down	LevelBii	HMDB0039780	292.1391	1.9403	M-H
Mabioside C	8.258E-6	5.573E-5	1.9884	0.0504	-4.3108	down	LevelBii	HMDB0040653	757.4143	3.4491	M+H-2H2O
Core Oligosaccharide	1.024E-8	3.454E-7	1.9808	2.3788	1.2502	up	LevelBii	HMDB0250452	755.2449	1.1003	M-H
Artonin E	2.497E-10	3.32E-8	1.9804	3.229	1.6911	up	LevelBii	HMDB0035186	917.298	1.1097	2M+FA-H
(3Ar,4S,7S,7As)-3A,4,5,6,7,7A-Hexahydro-1	4.12	3.15	1.9731	0.04	-4.404	dow	Lev	-	207.	5.8592	M+H

H-4,7-Methanoinden-6-Yl Propionate	E-6	6E-5		72	8	n	elBi		1372		
Xanthine	5.163E-10	5.011E-8	1.9711	0.2282	-2.1318	down	LevelBi	HMDB0000292;PW_C000198	153.0396	1.8336	M+H
Kanamycin	0.001333	0.003097	1.9646	0.2885	-1.7933	down	LevelBii	HMDB0015303	967.4702	5.7845	2M-H
Esculetin	2.532E-8	6.758E-7	1.9694	0.1075	-3.2182	down	LevelBi	HMDB0030819	177.0185	2.9885	M-H
Oxypurinol	6.212E-8	1.293E-6	1.9681	0.3391	-1.5603	down	LevelBii	HMDB0000786	151.0253	1.8366	M-H
Beta-D-Glucosaminyl-(1->4)-Beta-D-Glucosamine	1.322E-11	6.126E-9	1.9652	0.0196	-5.6734	down	LevelBii	HMDB0249120	305.1332	1.1771	M+H-2H2O
N-Trans-Feruloyltyramine	8.717E-9	3.099E-7	1.9659	0.0014	-9.4542	down	LevelBii	HMDB0029365	312.1222	5.6277	M-H
N-Fructosyl Phenylalanine	4.083E-5	0.000173	1.9589	0.287	-1.801	down	LevelBi	-	328.138	2.1616	M+H
4,4-Dimethyl-2-[3-Carboxylatopropyl]-2-Tridecyloxazolidine 3-Oxide	2.292E-6	2.002E-5	1.9639	0.1973	-2.3418	down	LevelBii	HMDB0246830	366.2991	8.5851	M-H2O-H
Adipic Acid	5.569E-9	2.285E-7	1.9565	9.2125	3.2036	up	LevelBii	HMDB0000448	127.0393	2.0252	M-H2O-H
Trans-3'-Hydroxycotinine O-Beta-D-Glucuronide	1.417E-5	8.536E-5	1.9595	0.1117	-3.1621	down	LevelBii	HMDB0341403	427.1347	4.0277	M+Hac-H
(2E)-3-(1H-Indol-2-Yl)-2-Propenoic Acid	4.131E-10	4.424E-8	1.9533	0.0497	-4.3312	down	LevelBi	-	188.0695	3.2023	M+H
Tragopogonsaponin D	2.984E-6	2.46E-5	1.9527	0.2241	-2.158	down	LevelBii	HMDB0037915	1099.5133	5.6277	M-H2O-H
Ethyl 2-Amino-3-Phenylpropanoate	1.348E-8	4.163E-7	1.9498	0.0227	-5.4622	down	LevelBi	HMDB0252046	194.1167	2.8952	M+H
1-(2,6-Dihydroxy-4-Methoxyphenyl)-3-Phenyl-1-Propanone	4.966E-7	6.159E-6	1.9511	0	-18.9214	down	LevelBii	HMDB0034435	331.1169	4.0648	M+Hac-H
N-(1-Deoxy-1-Fructosyl)Proline	1.842E-9	1.085E-7	1.947	0.0693	-3.8513	down	LevelBii	HMDB0038493	278.1215	0.737	M+H
Inosine	1.20	3.81	1.9497	2.42	1.2774	up	Lev	HMDB0000195;HMDB0247301;	269.	1.9919	M+H

	2E-8	8E-7		41			elBi	PW_C000127	0875		
Diisodityrosine	7.499E-8	1.473E-6	1.9476	0.164	-2.6085	down	LevelBii	HMDB0029798	739.2182	3.9904	M+Na-2H
Carbadox	1.121E-6	1.144E-5	1.9404	0.0591	-4.0799	down	LevelBii	HMDB0031762	307.0707	4.7218	M+FA-H
Apiin	3.702E-7	4.927E-6	1.9407	0.0842	-3.5697	down	LevelBi	HMDB0030843;LMPK12110337	565.1509	3.991	M+H
Capsianoside Ii	0.0009306	0.002258	1.9347	0.2739	-1.8683	down	LevelBii	HMDB0031892	1085.5305	5.4492	M+H
Neogitogenin 3-[Glucosyl-(1->2)-Glucosyl-(1->4)-Galactoside]	0.0032490	0.0091389	1.9365	0.3674	-1.4446	down	LevelBii	HMDB0041134	963.4787	7.1181	M+Cl, M+FA-H
Guanosine Monophosphate	8.548E-8	1.628E-6	1.9332	10.8007	3.4331	up	LevelBii	HMDB0001397	362.0487	1.4547	M-H
Proscillaridin	8.023E-7	8.763E-6	1.9342	0.1115	-3.165	down	LevelBii	HMDB0256851	565.2597	5.8957	M+Cl
Miglitol	6.033E-9	2.388E-7	1.9295	0.0678	-3.8827	down	LevelBii	HMDB0014634	188.0919	2.7083	M-H2O-H
Val-Phe-Phe	1.597E-6	1.513E-5	1.9242	0.0033	-8.2609	down	LevelBi	-	412.2205	3.9416	M+H
Cer(8:0_3O/14:1_(2Oh))	0.0013111	0.0041778	1.9266	0.0449	-4.477	down	LevelBi	-	384.3093	7.1967	M+H-H2O
3-Hydroxyquinidine	1.702E-8	4.994E-7	1.9175	0.2344	-2.093	down	LevelBii	HMDB0245907	395.1972	2.0314	M+CH3OH+Na
(R)-6'-O-(4-Geranyloxy-2-Hydroxycinnamoyl)-Marmin	1.046E-5	6.726E-5	1.9207	3.604	1.8496	up	LevelBii	HMDB0039061	635.2958	2.3204	M+Na-H2O
2-Methyl-4-(Pyridin-2-Yl)But-3-Yn-2-Ol	4.309E-8	9.949E-7	1.913	0.2576	-1.9567	down	LevelBi	-	144.0799	2.8653	M+H-H2O
2-Hydroxyadipic Acid	2.78E-10	3.531E-8	1.911	0.0523	-4.2563	down	LevelBii	HMDB0000321	221.0657	2.0441	M+Hac-H
5,10-Methylenetetrahydromethanopterin	2.425E-9	1.271E-7	1.9082	3.9577	1.9847	up	LevelBii	HMDB0060401	823.2318	1.0911	M+Cl
Val-His	3.00	0.00	1.9064	0.08	-3.601	dow	Lev	HMDB0029129	253.	0.7082	M-H

	9E-5	01566		24	4	n	elBii		1295		
Mifepristone	4.229E-7	5.451E-6	1.9066	0.0389	-4.685	down	LevelBii	HMDB0014972	434.247	6.3883	M+Na-H2O
Adomeglivant	2.859E-5	0.0001502	1.9075	0.2926	-1.7728	down	LevelBii	HMDB0248020	573.2949	2.0512	M+NH4
Methyl 3-Hydroxy-2-[(3-Hydroxybenzoyl)Amino]Propanoate	2.823E-6	2.355E-5	1.9004	3.8973	1.9625	up	LevelBi	-	257.1119	2.0414	M+NH4
Managlinat Dialanetil	3.923E-8	9.312E-7	1.9014	0.1305	-2.9377	down	LevelBii	HMDB0254319	467.1862	2.5469	M+H-H2O
B-D-Glucuronopyranosyl-(1->3)-A-D-Galacturonopyranosyl-(1->2)-L-Rhamnose	3.425E-7	4.635E-6	1.8975	8.0645	3.0116	up	LevelBii	HMDB0039728	497.1103	2.0252	M-H2O-H
3,3-Dimethylacrylic Acid	5.096E-13	7.837E-10	1.8989	0.289	-1.7906	down	LevelBi	HMDB0000509	101.0588	0.7273	M+H
Furylacryloylalanyllysine	0.0003725	0.001032	1.8922	0.1789	-2.4824	down	LevelBii	HMDB0247373	336.1552	2.1297	M-H
Dlcl(14:0/16:4)	3.147E-5	0.0001624	1.8999	0.1183	-3.0801	down	LevelBi	-	885.4197	5.9512	M+FA-H
N-Acetylaspartate	1.498E-6	1.444E-5	1.8928	0.1807	-2.4679	down	LevelBi	HMDB0000812	174.0399	0.7644	M-H
Apo-12'-Zeaxanthinal	2.838E-9	1.393E-7	1.8946	0.1181	-3.0822	down	LevelBii	HMDB0302023	347.2417	4.8442	M-H2O-H
Benazeprilat	8.807E-9	3.107E-7	1.8938	2.4683	1.3035	up	LevelBii	HMDB0060582	431.1391	0.886	M+Cl
Vanillyl Alcohol	0.000236	0.0006934	1.8925	0.3129	-1.6762	down	LevelBi	HMDB0032012	137.0591	6.3883	M+H-H2O
Maltotetraose	5.124E-6	3.773E-5	1.8933	2.4317	1.282	up	LevelBi	HMDB0001296	667.2244	1.0985	M+H
Ile-Ile-Thr	0.0001092	0.0003571	1.8902	0.2204	-2.1817	down	LevelBi	-	346.2315	2.35	M+H
Cilengitide	1.056E-5	6.788E-5	1.8874	0.201	-2.3145	down	LevelBi	HMDB0250242	606.3357	6.398	M+NH4

							i				
Desacetyl-Alacepril	0.0002282	0.0006741	1.891	9.626	3.2669	up	LevelBi	HMDB0251027	385.1206	5.0198	M+Na-2H
Timosaponin B Ii	9.277E-5	0.000312	1.8901	0.32	-1.644	down	LevelBi	-	925.4683	7.6294	M+, M+Na-H2O
Bn-Ncc-2	6.887E-6	4.789E-5	1.8888	0.0171	-5.8688	down	LevelBi	HMDB0038230	837.3169	4.0091	M+FA-H
Cyclohexanesulfamic Acid	2.58E-11	8.823E-9	1.8849	0.2836	-1.8179	down	LevelBi	HMDB0031340	180.068	2.8357	M+H
5-Sulfosalicylic Acid	6.239E-10	5.6E-8	1.8852	7.029	2.8133	up	LevelBi	HMDB0011725	216.9803	2.6326	M-H
Uridine 2'-Phosphate	3.986E-8	9.409E-7	1.883	6.8722	2.7808	up	LevelBi	HMDB0011641	323.027	1.1097	M-H
Xanthosine	1.325E-9	8.728E-8	1.8817	5.2037	2.3795	up	LevelBi	HMDB0000299;PW_C000203	283.0674	2.101	M-H
6-Hydroxy-4,4,7A-Trimethyl-5,6,7,7A-Tetrahydrobenzofuran-2(4H)-One	6.367E-9	2.478E-7	1.8808	0.2078	-2.2669	down	LevelBi	-	197.1165	3.9318	M+H
Flavone Base+3O,2Meo,O-Guaiacylglyceryl-Hex	4.419E-11	1.269E-8	1.8807	2.6571	1.4099	up	LevelBi	-	689.2056	0.7565	M+H
Medicagenate	6.342E-6	4.48E-5	1.8759	0.2884	-1.7941	down	LevelBi	HMDB0304417	499.3018	9.0755	M-H
Xi-7-Hydroxyhexadecanedioic Acid	3.876E-5	0.000173	1.8786	0.1508	-2.7293	down	LevelBi	HMDB0037830	301.2006	5.7845	M-H
N-(2-Hydroxy-3-Methylbutanoyl)Leucine	2.49E-9	1.286E-7	1.8771	0.0742	-3.7525	down	LevelBi	-	230.1385	5.0382	M-H
Alpha-Hydroxymetoprolol	8.69E-12	4.622E-9	1.8755	0.1554	-2.6864	down	LevelBi	HMDB0060994	284.1846	3.123	M+H
gamma-Glu-Cys-Gly	7.161E-8	1.432E-6	1.8745	2.477	1.3086	up	LevelBi	-	308.0903	1.2553	M+H
Capsianoside Iii	0.001866	0.004149	1.8658	0.35	-1.5146	down	LevelBi	HMDB0031891	1145.521	4.657	M+Cl, M+FA-H
1,2-Ethanediol Monoricinoleate	0.0001743	0.0005342	1.8789	0.2641	-1.9211	down	LevelBi	HMDB0252073	365.264	7.7929	M+Na
1,2-Cyclohexanedicarboxylic Acid, 4-Methyl-	1.75	2.78	1.8687	0.07	-3.768	dow	Lev	HMDB0253138	204.	3.5573	M+NH4

	9E-10	1E-8		34	7	n	elBii		1219		
Ent-6R,16Boh,17-Trihydroxy-7-Oxo-6,7-Seco-19,6-Kauranolide 6-O-Glucoside	2.986E-5	0.0001556	1.8615	6.9688	2.8009	up	LevelBii	HMDB0038546	493.2386	2.786	M+H-2H2O
Alpha-Cyperone	6.916E-5	0.000241	1.8697	0.101	-3.3076	down	LevelBi	-	219.174	6.447	M+H
Genistein 4'-O-Glucoside	1.694E-5	9.856E-5	1.8684	0.1271	-2.9758	down	LevelBi	HMDB0252680;LMPK12050168	431.0955	4.139	M-H
Cdp-Dg(Pgf2Alpha/22:5(4Z,7Z,10Z,13Z,16Z))	1.563E-6	1.489E-5	1.8671	0.2874	-1.7991	down	LevelBii	HMDB0292458	1146.516	5.4527	M+Na-2H
Ethyl Oleate	6.102E-5	0.000216	1.8701	0.467	-1.0985	down	LevelBi	HMDB0034451;LMFA07010880	311.293	8.7553	M+H
Beta-D-Xylopyranosyl-(1->5)-Alpha-L-Arabinofuranosyl-(1->5)-L-Arabinose	1.358E-7	2.302E-6	1.8631	2.8405	1.5061	up	LevelBii	HMDB0038860	413.1287	2.0063	M-H
Leu-Pro-Ala	6.54E-6	4.589E-5	1.8608	0.0156	-5.9989	down	LevelBi	-	300.1901	2.1616	M+H
Torvonin A	4.956E-6	3.672E-5	1.8646	0.2245	-2.1554	down	LevelBii	HMDB0030732	759.414	6.4339	M+Cl
Vinyl Ether	4.013E-10	4.333E-8	1.8598	0.0039	-8.0147	down	LevelBii	HMDB0259824	185.0807	5.3422	2M+FA-H
Janthitrem G	4.816E-6	3.589E-5	1.8625	16.6328	4.056	up	LevelBii	HMDB0030531	594.36	3.2123	M+H-2H2O
Araloside A	0.0008331	0.002055	1.8577	0.3041	-1.7172	down	LevelBii	HMDB0034535	961.4617	6.9054	M+Cl
Adenine	6.638E-9	2.549E-7	1.859	0.3123	-1.6791	down	LevelBi	HMDB0000034;PW_C000024	136.0618	1.1086	M+H
Ubiquinone-1	4.354E-6	3.3E-5	1.8572	0.0715	-3.8051	down	LevelBii	HMDB0002012	314.1369	4.4039	M+ACN+Na
15-Epi Prostaglandin A1	4.043E-6	3.111E-5	1.86	0.2053	-2.2842	down	LevelBi	-	337.2354	5.8592	M+H
Pgp(Pge2/I-14:0)	1.527E-6	1.468E-5	1.859	0.002	-8.9623	down	LevelBii	HMDB0275018	905.3961	6.1478	M+Cl
Pgp(Pgf2Alpha/22:6(4Z,7Z,10Z,13Z,16Z,19Z))	8.491E-7	9.148E-6	1.8583	0.0494	-4.3389	down	LevelBii	HMDB0274193	977.4479	5.322	M+Na-H2O

Fahfa(16:0/6:0)	2.803E-5	0.0001478	1.8543	0.0786	-3.6697	down	LevelBi	-	369.2989	9.4389	M-H
N-Linoleoyl Threonine	1.037E-9	7.406E-8	1.8544	10.4172	3.3809	up	LevelBii	HMDB0241985	440.2997	6.4062	M+Hac-H
Fa(18:3)	0.0003527	0.0009829	1.859	0.2782	-1.8458	down	LevelBi	-	555.4375	8.4549	2M-H
N-[4-(Aminosulfonyl)Phenyl]Propanamide	5.401E-10	5.106E-8	1.8494	0.1885	-2.4075	down	LevelBi	-	229.063	2.9249	M+H
Oxyglutinosone	6.861E-10	5.762E-8	1.8499	0.1552	-2.6876	down	LevelBii	HMDB0302978	281.138	3.2337	M+FA-H
7-[(3-Methoxy-4-Propan-2-Yloxyphenyl)Methyl]-7-Azaspiro[3.5]Nonan-3-Ol	8.224E-5	0.0002813	1.854	0.1463	-2.7731	down	LevelBi	-	320.2202	6.7681	M+H
Capsaicin	8.51E-5	0.0002897	1.8502	0.4627	-1.1119	down	LevelBi	HMDB0002227;HMDB0302729;LMFA08020085	306.2059	6.3883	M+H
[8]-Shogaol	0.0001639	0.0005068	1.8532	0.1505	-2.7324	down	LevelBi	HMDB0031463	322.2359	7.0989	M+NH4
3-Phenylpropyl Acetate	7.263E-8	1.449E-6	1.8458	0.0018	-9.0833	down	LevelBii	HMDB0033386	223.096	5.6553	M+FA-H
Carmine Red	4.594E-5	0.000173	1.8462	0.1458	-2.7782	down	LevelBii	HMDB0030658	513.0674	4.2781	M+Na-2H
Lactobionic Acid	3.278E-8	8.061E-7	1.8428	6.2156	2.6359	up	LevelBii	HMDB0253945	379.0852	1.9687	M+Na-2H
Ala-Arg	1.819E-7	2.894E-6	1.8426	0.3318	-1.5918	down	LevelBii	HMDB0028681	268.1392	1.5719	M+Na
Ser-Ser	6.005E-10	5.508E-8	1.8418	0.001	-10.0257	down	LevelBii	HMDB0029048	383.143	2.2829	2M-H
Thr-Ala-Phe	6.159E-7	7.173E-6	1.839	0.0134	-6.2186	down	LevelBi	-	338.1684	2.5772	M+H
Pinoquercetin	3.082E-5	0.0001601	1.8433	0.031	-5.0125	down	LevelBii	HMDB0256564	337.0309	5.2503	M+Na-2H

Kievitone Hydrate	5.312E-5	0.0001923	1.8416	0.0923	-3.4371	down	LevelBii	HMDB0038111	373.127	4.2596	M-H
3-Hydroxynevirapine Glucuronide	1.178E-8	3.766E-7	1.8355	0.1558	-2.6818	down	LevelBii	HMDB0060743	479.1177	4.3429	M+Na-2H
14-Hdohe	7.757E-6	5.287E-5	1.8336	0.0655	-3.9322	down	LevelBii	HMDB0060044	391.2059	2.4066	M+Cl
Mozenavir	3.965E-7	5.177E-6	1.8355	0.1442	-2.7942	down	LevelBii	HMDB0254911	571.2459	1.846	M+Cl
Decanoyl-L-Carnitine	0.0006556	0.00167	1.8414	4.6293	2.2108	up	LevelBi	HMDB0000651	316.2475	5.361	M+H
Isoeruboside B	0.0004311	0.00117	1.8294	0.4709	-1.0865	down	LevelBii	HMDB0029769	1125.5302	7.044	M+FA-H
Cyclosquamosin D	3.387E-7	4.606E-6	1.8318	0.0888	-3.4926	down	LevelBii	HMDB0303369	835.3926	5.5541	M-H
Hydroxy-Lacosamide	6.232E-8	1.293E-6	1.8282	0.0036	-8.1137	down	LevelBii	HMDB0061146	281.1129	1.8366	M-H
24,24-Difluoro-1Alpha,25-Dihydroxyvitamin D3	6.039E-5	0.0002141	1.8219	0.3684	-1.4406	down	LevelBii	HMDB0242645	473.2853	9.0478	M+Na-2H
2-Hydroxy-4-(4-Methoxyphenyl)-6-Phenylnicotinonitrile	2.234E-5	0.0001235	1.823	0.3097	-1.6912	down	LevelBi	-	320.1424	2.6865	M+NH4
Tamsulosin	9.89E-9	3.366E-7	1.8236	0.2706	-1.8856	down	LevelBi	HMDB0014844;HMDB0259951	409.1811	3.133	M+H
Stearidonic Acid Ethyl Ester	0.0006308	0.001615	1.8253	0.2289	-2.1269	down	LevelBi	-	305.2458	7.6196	M+H
N-Fructosyl Isoleucine	1.887E-5	0.0001076	1.8184	0.4363	-1.1966	down	LevelBi	-	294.1541	1.9424	M+H
N-Lactoyl-Tryptophan	6.047E-10	5.508E-8	1.8192	0.173	-2.5309	down	LevelBii	HMDB0062178	297.0842	2.979	M+Na-2H
Tropic Acid	1.185E-7	2.08E-6	1.818	0.169	-2.565	down	LevelBi	HMDB0062590	167.0686	3.7548	M+H
Leu-Leu-Phe	1.06	1.09	1.8131	0.07	-3.716	dow	Lev	-	392.	4.0598	M+H

	4E-6	5E-5		61	5	n	elBi		2519		
Thromboxane B2	2.18E-10	3.1E-8	1.8145	0.3059	-1.7088	down	LevelBi	HMDB0003252;PW_C001820	393.2273	2.2904	M+Na
Agnuside	2.827E-10	3.531E-8	1.8142	0.1114	-3.1664	down	LevelBii	HMDB0036561	431.1355	4.5992	M+H-2H2O
Npc	8.553E-7	9.193E-6	1.8158	0.0031	-8.3441	down	LevelBii	HMDB0060499	1081.4294	5.2134	2M+FA-H
2-Cyclopropyl-4-Quinolinecarboxylic Acid	9.029E-6	5.992E-5	1.8137	0.2499	-2.0007	down	LevelBi	-	214.0853	2.9844	M+H
Nona-5,7-Dienoic Acid	6.016E-6	4.298E-5	1.8088	0.149	-2.7467	down	LevelBii	HMDB0341026	137.0953	4.7951	M+H-H2O
Ala-Arg-Tyr	3.837E-7	5.05E-6	1.8094	0.2206	-2.1806	down	LevelBi	-	409.2163	3.9615	M+H
8-Aminooctanoic Acid	5.472E-9	2.265E-7	1.8087	0.2393	-2.0632	down	LevelBii	HMDB0247418	160.1324	2.6965	M+H
N-(3,5-Dimethylphenyl)-2-(5-Pyrazin-2-Yl-1,3,4-Oxadiazol-2-Yl)Acetamide	0.002351	0.005077	1.8164	0.3054	-1.7111	down	LevelBi	-	310.1281	2.1616	M+H
Methyl (9Z,14Z)-12,13,16-Trihydroxyoctadeca-9,14-Dienoate	3.607E-8	8.662E-7	1.8083	300.7685	8.2325	up	LevelBi	-	384.2719	5.5369	M+ACN+H
Eriojaposide B	1.488E-5	8.885E-5	1.8131	0.215	-2.2174	down	LevelBii	HMDB0038029	1077.5079	7.3405	2M+FA-H
28-Glucosyloleanolic Acid 3-[Rhamnosyl-(1->2)-Galactosyl-(1->3)-Glucuronide]	2.546E-8	6.76E-7	1.809	0.1892	-2.4021	down	LevelBii	HMDB0040859	1151.5092	5.4436	M+Cl
Paxilline	1.79E-6	1.646E-5	1.8049	0.2144	-2.2216	down	LevelBi	HMDB0030323	458.2331	1.6199	M+Na
7(14)-Bisabolene-2,3,10,11-Tetrol	1.707E-6	1.591E-5	1.8065	0.1212	-3.0443	down	LevelBii	HMDB0035918	317.1952	4.8996	M+FA-H
Benzo[A]Pyrene-9,10-Oxide	5.64E-8	1.202E-6	1.8041	2.5043	1.3244	up	LevelBii	HMDB0060090	313.089	2.0345	M+FA-H
Oxoglutaric Acid	4.392E-10	4.475E-8	1.8045	0.1454	-2.7815	down	LevelBii	HMDB0000208	205.0345	2.1593	M+Hac-H
N-3-Hydroxyoctanoyl-L-Homoserine Lactone	2.311E-9	1.243E-7	1.8043	0.0109	-6.5151	down	LevelBii	HMDB0341343	264.1231	5.1765	M+Na-2H

Spinacetin 3-Gentiobioside	6.763E-10	5.747E-8	1.8041	0.411	-1.2829	down	LevelBii	HMDB0037469	729.1887	3.7495	M+Hac-H
Apiosylglucosyl 4-Hydroxybenzoate	1.754E-6	1.624E-5	1.803	0.091	-3.4586	down	LevelBii	HMDB0035318	431.12	3.8785	M-H
Ala-Pro-Pro	5.65E-9	2.305E-7	1.8018	0.4393	-1.1867	down	LevelBi	-	284.1588	1.9821	M+H
Cassiaside B2	6.533E-10	5.641E-8	1.8017	2.517	1.3317	up	LevelBii	HMDB0032833	965.2839	1.9969	M+FA-H
Formononetin 7-(6''-Methylmalonylglucoside)	5.891E-7	6.957E-6	1.8012	0.2623	-1.9305	down	LevelBii	HMDB0029494	1105.2822	3.7404	2M+FA-H
1-Cyclohexyl-3-(1-Propylsulfonyl-3,4-Dihydro-2H-Quinolin-7-Yl)Urea	5.036E-7	6.235E-6	1.8	6.963	2.7997	up	LevelBi	-	380.2025	5.1562	M+H
Hetacillin	2.665E-7	3.824E-6	1.7961	0.1432	-2.804	down	LevelBii	HMDB0014877	388.135	3.8134	M-H
Delta-Hydroxylysylnorleucine	1.065E-6	1.095E-5	1.7939	0.324	-1.6258	down	LevelBii	HMDB0253190	274.1762	1.9919	M+H-H2O
Meta-O-Dealkylated Flecainide Lactam	1.294E-9	8.606E-8	1.795	0.1209	-3.0477	down	LevelBii	HMDB0060832	391.11	3.1301	M+FA-H
(S)-3-Hydroxy-N-Methylcoclaurine	1.9E-6	1.73E-5	1.7963	0.088	-3.5058	down	LevelBii	HMDB0006921	316.1536	4.0598	M+H
Monotropein	6.536E-8	1.341E-6	1.7924	2.3529	1.2344	up	LevelBii	HMDB0035608	435.111	2.0252	M+FA-H
19-Noretiocholanolone 3-O-Glucuronide	0.0001294	0.0004134	1.7926	0.1147	-3.1236	down	LevelBii	HMDB0341226	453.2456	6.0806	M-H
Ala-Gly-Leu-Val-Ser	8.774E-6	5.852E-5	1.7942	4.8693	2.2837	up	LevelBi	-	444.2446	3.273	M-H
Leucomycin	1.047E-9	7.44E-8	1.7912	2.5952	1.3758	up	LevelBii	HMDB0254035	724.3903	3.38	M+Na
Pro-Val-Val	6.011E-7	7.034E-6	1.7875	0.369	-1.4382	down	LevelBi	-	314.2059	2.3602	M+H
Elocalcitol	1.544E-6	1.476E-5	1.7833	0.4921	-1.023	down	LevelBii	HMDB0251753	443.3276	9.1971	M+H

Fahfa(20:4/6:0)	0.0008299	0.002048	1.7933	0.1659	-2.5914	down	LevelBi	-	417.2982	9.121	M-H
1,1'-(Phenylazanediyl)Dipropan-2-Ol	3.817E-8	9.106E-7	1.7843	0.2335	-2.0988	down	LevelBi	-	227.1744	2.3602	M+NH4
Capecitabine	2.478E-9	1.285E-7	1.7834	49.1383	5.6188	up	LevelBii	HMDB0015233	418.1634	4.1947	M+Hac-H
1-Methylene-5Alpha-Androstan-3Alpha-Ol-17-One	7.141E-5	0.000248	1.7884	0.4441	-1.1711	down	LevelBi	-	303.2307	7.0116	M+H
Halometasone	1.894E-6	1.727E-5	1.7833	0.0509	-4.2972	down	LevelBii	HMDB0253041	479.1176	4.8627	M+Cl
Abscisic Acid	3.091E-9	1.489E-7	1.7804	0.1208	-3.0489	down	LevelBi	HMDB0035140;HMDB0036093	263.1273	4.6849	M-H
1-Methylene-5Alpha-Androstan-3Alpha-Ol-17-One Glucuronide	2.662E-7	3.823E-6	1.7801	0.2228	-2.1663	down	LevelBii	HMDB0341213	479.2605	5.2342	M+H
Lyciumoside Vii	1.392E-6	1.356E-5	1.7764	0.3901	-1.358	down	LevelBii	HMDB0033501	983.4666	4.8996	M+FA-H
N-Docosahexaenoyl Valine	2.22E-5	0.0001228	1.7776	0.3225	-1.6328	down	LevelBii	HMDB0242025	460.3389	5.2049	M+CH3OH+H
7-Hydroxybutylidenephthalide 7-(6-Malonylglucoside)	5.718E-10	5.297E-8	1.7751	0.0585	-4.0958	down	LevelBii	HMDB0034753	497.13	4.7428	M+FA-H
Retinol	3.562E-7	4.785E-6	1.7755	0.0859	-3.5412	down	LevelBi	LMPR01090001	269.225	4.6482	M+H-H2O
Gambogic Acid	0.001234	0.002895	1.7726	0.4079	-1.2939	down	LevelBii	HMDB0034095	687.3194	6.0435	M+Hac-H
Roxindole	2.342E-9	1.243E-7	1.7736	0.0256	-5.2867	down	LevelBii	HMDB0257319	405.2205	2.7743	M+Hac-H
15(S)-Hpete	4.628E-8	1.04E-6	1.7729	0.0215	-5.5425	down	LevelBii	HMDB0004244	371.2025	4.9828	M+Cl
Fructosylvaline	0.002229	0.004845	1.7683	0.1642	-2.6065	down	LevelBii	HMDB0252498	278.1238	1.156	M-H
N-Palmitoyl Gaba	1.512E-5	8.981E-5	1.7742	0.2917	-1.7775	down	LevelBii	HMDB0062338	340.2829	8.5103	M-H

Tyr-His-Asp-Asp	6.193E-8	1.291E-6	1.7703	0.1255	-2.994	down	LevelBi	-	547.1772	2.4066	M-H
Phe-Pro-Thr	0.0003954	0.001087	1.762	0.147	-2.7663	down	LevelBi	-	364.1857	2.537	M+H
Orientin 7,3'-Dimethyl Ether	1.36E-7	2.303E-6	1.761	2.9756	1.5732	up	LevelBii	HMDB0037450	475.1288	2.0063	M-H
2-[4-(4-Tert-Butylphenyl)Sulfonylpiperazin-1-Yl]-1-Pyrrolidin-1-Ylpropan-1-One	1.097E-5	6.993E-5	1.7593	6.7756	2.7604	up	LevelBi	-	430.2152	5.8787	M+Na
(3-Ethyl-3-Hydroxy-7-Azaspiro[3.5]Nonan-7-Yl)-Thiophen-2-Ylmethanone	6.602E-8	1.352E-6	1.7602	0.3373	-1.568	down	LevelBi	-	280.1382	1.1576	M+H
Methyl Cinnamate	1.346E-5	8.225E-5	1.7636	0.1221	-3.0336	down	LevelBi	HMDB0303905;HMDB0033833	163.0744	4.0206	M+H
Strobilurin	2.135E-7	3.278E-6	1.7603	0.0031	-8.3154	down	LevelBii	HMDB0258515	441.1953	3.9344	M-H
Phytosphingosine-1-P	0.0003495	0.0009753	1.7547	0.4136	-1.2736	down	LevelBii	HMDB0012280	415.2962	8.9216	M+NH4
1-Beta-Hydroxymedroxyprogesterone	6.19E-8	1.291E-6	1.7552	0.2942	-1.7653	down	LevelBii	HMDB0061065	424.2453	2.8159	M+ACN+Na
N-Isobutyloctadeca-Trans-2-Trans-4-Dienamide	1.25E-8	3.921E-7	1.7568	0.2551	-1.9708	down	LevelBii	HMDB0302880	380.3154	8.8985	M+FA-H
Valtrate	1.129E-8	3.678E-7	1.7541	0.0791	-3.661	down	LevelBii	HMDB0034493	233.0416	3.4613	M+Na-2H
Pro-Ile	3.23E-10	3.743E-8	1.7542	0.3036	-1.7196	down	LevelBi	HMDB0304810	211.143	2.1912	M+H-H2O
Carpinontriol B	1.491E-7	2.473E-6	1.7547	0.2836	-1.8183	down	LevelBi	-	345.1304	3.6363	M+H
20-Hydroxy-6Z,15Z-Eicosadienoic Acid	0.002196	0.004782	1.7571	0.3138	-1.672	down	LevelBii	HMDB0245564	325.2719	8.4642	M+H
23-O-Beta-D-Glucopyranosyl-25-Methyldolichosterone	0.0001569	0.0004879	1.7508	43.5212	5.4436	up	LevelBii	HMDB0301798	677.3672	8.0379	M+K
Fahfa(18:1/6:0)	0.0003691	0.001024	1.7486	0.2501	-1.9994	down	LevelBi	-	395.3141	9.4389	M-H
2-[2-(4-Aminophenyl)Ethylamino]Ethanol	4.915E-5	0.000181	1.7475	0.3315	-1.5929	down	LevelBi	-	213.1588	2.1512	M+CH3OH+H

Val-Phe-Val-Tyr	3.012E-7	4.182E-6	1.7504	0.3293	-1.6025	down	LevelBii	HMDB0304812	571.2819	2.0535	M+FA-H
Queuine	9.384E-5	0.0003147	1.745	0.1869	-2.4195	down	LevelBii	HMDB0001495	278.1223	1.02	M+H
5-(3-Pyridyl)-2-Hydroxytetrahydrofuran	2.617E-8	6.863E-7	1.7437	0.1558	-2.6826	down	LevelBii	HMDB0062407	210.0761	3.0167	M+FA-H
Acetyldiphenylalanyl-Leucyl-Aspartyl-Isoleucyl-Isoleucyl-Tryptophan	1.624E-5	9.512E-5	1.7451	0.2227	-2.1671	down	LevelBii	HMDB0256173	968.4728	5.9973	M+FA-H
Irbesartan	8.335E-8	1.596E-6	1.742	0.269	-1.8942	down	LevelBi	HMDB0015163;PW_C009460	427.2218	8.6225	M-H
Fahfa(18:2/6:0)	1.621E-5	9.504E-5	1.7427	0.3101	-1.6892	down	LevelBi	-	393.2989	9.2145	M-H
3,4',5-Trihydroxy-3',7-Dimethoxyflavanone	1.612E-8	4.797E-7	1.7382	0.3752	-1.4143	down	LevelBii	HMDB0037503	353.0663	2.5287	M+Na-2H
Adenosyl Cobyrinic Acid A,C Diamide	2.46E-6	2.109E-5	1.74	0.1747	-2.5173	down	LevelBii	HMDB0001083	1167.4386	7.1364	M-H2O-H
Rebaudioside A	0.0002143	0.0006389	1.7414	0.0535	-4.2236	down	LevelBii	HMDB0034950	967.4383	5.9668	M+H
Coprine	9.704E-9	3.334E-7	1.7352	0.0407	-4.6186	down	LevelBii	HMDB0034266	201.0868	0.727	M-H
Ile-Leu-Gln	0.0001092	0.0003572	1.7349	0.2507	-1.9959	down	LevelBi	-	373.2422	2.1616	M+H
Neuromedin B (4-10)	3.021E-8	7.584E-7	1.7345	0.2662	-1.9096	down	LevelBii	HMDB0013017	846.3751	2.1593	M-H
Atropine	6.885E-6	4.789E-5	1.7339	0.3579	-1.4822	down	LevelBi	HMDB0000779	322.1992	3.7153	M+CH3OH+H
(2S)-2-[[(2R)-2-Amino-3-Methylbutanoyl]Amino]-4-Methylsulfanylbutanoic Acid	3.15E-5	0.0001624	1.7306	0.4136	-1.2736	down	LevelBi	-	249.1257	2.1512	M+H
Halosulfuron-Methyl	4.038E-11	1.208E-8	1.7331	0.1927	-2.3754	down	LevelBii	HMDB0034859	493.0592	2.5098	M+Hac-H
Cyclovirobuxine D	0.000643	0.001641	1.7363	0.1208	-3.0492	down	LevelBi	HMDB0250688	407.3388	7.0892	M+Na-H2O

Tenuazonic Acid	0.0001535	0.0004795	1.7267	0.1731	-2.5303	down	LevelBi	HMDB0036074	198.1115	4.9511	M+H
Tri-N-Acetylchitotriose	1.169E-7	2.063E-6	1.7322	0.0023	-8.7582	down	LevelBii	HMDB0006698	648.2239	2.416	M+Na-2H
Ile-Pro-Pro	2.612E-5	0.0001395	1.7252	0.4135	-1.274	down	LevelBii	HMDB0253021	324.1914	2.1976	M-H
(2'E,4'Z,8E)-Colneleic Acid	0.0001333	0.0004239	1.7323	0.1335	-2.9049	down	LevelBii	HMDB0030995	329.1868	7.415	M+Cl
1-Linoleoyl Glycerol	0.0146	0.02535	1.7362	2.4569	1.2968	up	LevelBi	-	355.2829	8.3582	M+H
Isomaltulose	2.118E-9	1.184E-7	1.7244	1.6665	0.7368	up	LevelBii	HMDB0341167	377.0842	0.7177	M+Cl
Thr-Hyp	7.056E-7	7.972E-6	1.7239	0.2859	-1.8065	down	LevelBii	HMDB0029062	231.0978	1.9499	M-H
Mg(5-Iso Pgf2Vi/0:0/0:0)	3.931E-8	9.316E-7	1.7254	0.1295	-2.9489	down	LevelBii	HMDB0260485	421.2186	5.4251	M+Na-2H
Val-Ile-Phe	4.269E-7	5.489E-6	1.7202	0.2459	-2.0237	down	LevelBi	-	378.2366	3.7941	M+H
Phenylalaninamide	0.0003001	0.0008528	1.72	0.2681	-1.8991	down	LevelBi	-	197.1281	2.0614	M+CH3OH+H
Palmitoyl Glucuronide	0.008535	0.01584	1.7261	2.3353	1.2236	up	LevelBii	HMDB0010331	399.2736	8.3544	M-H2O-H
Sipoglitazar	5.282E-7	6.434E-6	1.7214	0.0009	-10.0604	down	LevelBii	HMDB0258310	444.1378	4.4817	M-H2O-H
Naringin 6"-Rhamnoside	4.401E-11	1.269E-8	1.7205	2.4971	1.3203	up	LevelBii	HMDB0038048	765.1998	1.0886	M+K
Paeonol	2.752E-6	2.306E-5	1.7166	0.2192	-2.1894	down	LevelBii	HMDB0256081	225.0755	3.4613	M+Hac-H
Pro-Pro-Phe	4.356E-8	1.002E-6	1.7174	0.4343	-1.2031	down	LevelBii	HMDB0304811	358.1777	3.0733	M-H
Thr-Arg-Lys-Glu	0.00	0.00	1.7211	0.02	-5.391	dow	Lev	-	531.	5.9789	M-H

	0109	0357		38	5	n	elBi		2957		
3-Ureidopropionic Acid	1.935E-12	1.737E-9	1.7177	0.163	-2.6168	down	LevelBi	HMDB0000026	115.0495	1.1285	M+H-H2O
(2S,3R,4R,5S,6S)-2-(Hydroxymethyl)-6-Phenylmethoxyoxane-3,4,5-Triol	3.856E-9	1.775E-7	1.7139	0.1465	-2.771	down	LevelBii	HMDB0243491	269.1015	3.1396	M-H
8-Methyl-2-(Trifluoromethyl)Quinolin-4-Ol	2.915E-6	2.415E-5	1.7147	0.3271	-1.6122	down	LevelBi	-	228.0614	3.8723	M+H
Asp-His	2.482E-5	0.0001344	1.7112	0.2934	-1.769	down	LevelBi	HMDB0028755	325.111	4.3449	M+CH3OH+Na
Apc	4.008E-6	3.091E-5	1.7114	0.1856	-2.4295	down	LevelBii	HMDB0060661	677.2796	4.2873	M+Hac-H
Val-Ile-Arg	0.00022	0.000653	1.7044	0.1799	-2.475	down	LevelBi	-	387.2688	1.9026	M+H
Cis-3-Methyl Thiofentanyl	2.571E-5	0.0001381	1.7075	0.1463	-2.7725	down	LevelBi	-	357.2009	5.7522	M+H
Arg-Met-His	7.788E-10	6.179E-8	1.7062	0.2457	-2.025	down	LevelBi	-	460.2478	1.1086	M+NH4
16-Acetylpriverogenin A	2.538E-5	0.0001369	1.7016	0.3382	-1.564	down	LevelBii	HMDB0034529	514.3633	9.1283	M+
Tyr-Gly-Gly-Trp-Leu	1.765E-8	5.131E-7	1.7013	0.0147	-6.0852	down	LevelBii	HMDB0259342	615.2601	3.48	M+Na-2H
Yessotoxin	1.166E-8	3.754E-7	1.6994	0.3387	-1.5621	down	LevelBii	HMDB0033320	1187.4818	5.4527	M+FA-H
Val-Phe-Arg	6.684E-6	4.678E-5	1.6954	0.2284	-2.1302	down	LevelBi	-	421.2523	2.3204	M+H
Estradiol Valerate	8.889E-9	3.123E-7	1.6979	0.2339	-2.096	down	LevelBii	HMDB0251966	391.2062	2.2829	M+Cl
Dodec-2-Enedioic Acid	1.018E-5	6.592E-5	1.6965	0.1946	-2.3612	down	LevelBii	HMDB0340663	246.1693	5.5564	M+NH4
Ile-Glu-Thr-Asp-Fluoromethyl Ketone	3.504E-8	8.481E-7	1.6979	0.1588	-2.6546	down	LevelBii	HMDB0253396	473.208	2.8956	M-H2O-H
Gly-Lys-Phe	0.00	0.00	1.6892	0.06	-4.049	dow	Lev	-	351.	1.9026	M+H

	02256	06671		04		n	elBi		2026		
Pe(Pgf1Alpha/24:1(15Z))	4.082E-5	0.000173	1.6959	0.2496	-2.0021	down	LevelBii	HMDB0284643	882.6224	8.9077	M-H2O-H
Merodesmosine	8.98E-5	0.0003036	1.6914	0.4219	-1.2449	down	LevelBii	HMDB0030407	403.2527	2.8059	M+H
Pectic Acid	0.00041132	0.001128	1.6904	0.1463	-2.773	down	LevelBii	HMDB0003363	175.0234	1.8648	M-H2O-H
Pyroglutamyllysine	1.149E-7	2.044E-6	1.6938	0.1394	-2.8432	down	LevelBii	HMDB0341383	302.1348	2.5098	M+FA-H
Guanosine	4.81E-11	1.321E-8	1.6941	2.3916	1.258	up	LevelBi	HMDB0000133;PW_C000087	284.0977	1.9721	M+H
Retaspimycin	0.0001636	0.0005062	1.6926	0.1354	-2.8847	down	LevelBii	HMDB0257169	570.3168	3.6363	M+H-H2O
Arg-Pro-Pro-Gly-Phe-Ser-Pro	3.39E-5	0.0001727	1.6936	0.0706	-3.8248	down	LevelBii	HMDB0248578	761.3786	3.3603	M+Na-H2O
3-Butyn-1-Al	0.007307	0.01384	1.7012	0.2009	-2.3152	down	LevelBii	HMDB0006853	69.0334	0.6887	M+H
Pip(Pgf2Alpha/16:0)	1.25E-5	7.74E-5	1.6937	0.1446	-2.7899	down	LevelBii	HMDB0278446	1023.4594	5.5172	M+Cl
Tomatine	2.339E-5	0.0001279	1.6936	0.3608	-1.4708	down	LevelBii	HMDB0034103	1092.5546	5.0105	M+Hac-H
Dlcl(16:4/22:7)	9.282E-5	0.0003121	1.6893	0.2496	-2.0021	down	LevelBi	-	983.4332	4.8351	M+FA-H
Lys-Ile-Asn	0.00029958	0.00084222	1.6934	4.3724	2.1284	up	LevelBi	-	374.2368	1.9026	M+H
1-[[3-(3-Fluorophenyl)-1,2,4-Oxadiazol-5-Yl] Methyl]-4-Phenylpyrazine-2,3-Dione	1.136E-8	3.691E-7	1.6872	1.7068	0.7713	up	LevelBi	-	365.1044	0.708	M+H
Alpha-Zearalenol	3.242E-6	2.623E-5	1.6883	0.2625	-1.9295	down	LevelBii	HMDB0041824	362.1941	3.7845	M+ACN+H
Mlcl(14:0/14:1/18:3)	0.000444	0.001201	1.6931	0.3144	-1.6692	down	LevelBi	-	1123.636	7.5551	M+FA-H

	1								5		
Fruleuile	2.038E-8	5.735E-7	1.6853	0.3867	-1.3708	down	LevelBi	-	407.2371	2.7762	M+H
2',4',6'-Trihydroxydihydrochalcone 2'-Glucoside	8.676E-7	9.305E-6	1.6866	0.1073	-3.22	down	LevelBii	HMDB0037483	441.1133	4.2596	M+Na-2H
Val-Thr	3.965E-5	0.000173	1.6816	0.3012	-1.731	down	LevelBii	HMDB0029137	219.133	1.1086	M+H
2Beta-Hydroxytestosterone	3.821E-7	5.039E-6	1.6847	0.0757	-3.7239	down	LevelBii	HMDB0012654	349.1992	5.5908	M+FA-H
Streptozocin	8.191E-8	1.577E-6	1.683	0.1356	-2.8827	down	LevelBii	HMDB0014572	264.0855	3.675	M-H
Dihydrogenistein	1.152E-5	7.281E-5	1.6865	0.1465	-2.7711	down	LevelBii	HMDB0005897	271.0597	5.0474	M-H
Desmethyldihydrocapsaicin	7.862E-5	0.0002702	1.6875	0.0852	-3.5522	down	LevelBi	-	292.19	6.3508	M-H
Lys-Asp-Tyr	1.166E-5	7.333E-5	1.684	0.0391	-4.6766	down	LevelBii	HMDB0304806	459.1635	6.1478	M+Cl
Hesperidin	4.872E-10	4.893E-8	1.6835	7.9152	2.9846	up	LevelBii	HMDB0003265	591.1746	1.1003	M-H2O-H
Lpc(O-20:2)	2.528E-5	0.0001365	1.6853	0.089	-3.4907	down	LevelBi	-	578.3781	8.5195	M+FA-H
Gibberellin A28	6.136E-9	2.408E-7	1.6824	0.034	-4.8763	down	LevelBii	HMDB0303573	375.1415	3.48	M-H2O-H
N-Isobutyl-2-(2-Methyl-1H-Indol-1-Yl)Acetamide	2.488E-6	2.131E-5	1.681	0.1262	-2.9866	down	LevelBi	-	308.174	4.1978	M+ACN+Na
N-(1-Deoxy-1-Fructosyl)Leucine	8.155E-5	0.0002791	1.6838	3.0187	1.5939	up	LevelBii	HMDB0037840	292.1396	1.1283	M-H
Prolinamide,D-Tyrosyl-L-Arginylglycyl-4-Nitro-L-Phenylalanyl-	1.002E-6	1.044E-5	1.6804	0.3979	-1.3293	down	LevelBii	HMDB0247954	727.3134	5.3238	M+FA-H
2,3-Butanediol Glucoside	3.519E-7	4.741E-6	1.6796	3.6824	1.8807	up	LevelBii	HMDB0040822	297.1179	2.1393	M+FA-H

4-Deoxytetronic Acid	8.35E-10	6.453E-8	1.6775	0.4536	-1.1406	down	LevelBi	HMDB0000549	87.0436	0.6887	M+H
6-Carboxy-5,6,7,8-Tetrahydropterin	1.067E-8	3.563E-7	1.6786	0.4612	-1.1164	down	LevelBii	HMDB0060410	246.0396	2.9046	M+Cl
Ile-Lys-Tyr	7.087E-9	2.671E-7	1.6761	0.5343	-0.9043	down	LevelBi	-	423.2575	2.3204	M+H
1-Deoxy-1-Morpholino-D-Fructose	4.27E-8	9.894E-7	1.6743	0.0001	-12.8314	down	LevelBii	HMDB0243858	270.0939	3.4894	M+Na-2H
Spinorphin	0.000524	0.001383	1.68	0.1303	-2.9398	down	LevelBii	HMDB0059790	875.4637	6.7023	M-H
Androsterone Glucuronide	0.0002391	0.0007006	1.6774	0.3936	-1.3454	down	LevelBii	HMDB0002829	525.2677	6.6005	M+Hac-H
Galactitol	5.245E-8	1.141E-6	1.6728	0.0035	-8.1666	down	LevelBii	HMDB0000107	181.0708	0.671	M-H
(4-Formyl-9-Methoxy-5-Methylnaphtho[2,3-B]Furan-3-Yl)Methyl Acetate	3.344E-6	2.683E-5	1.6715	0.2812	-1.8304	down	LevelBi	-	345.1303	4.2569	M+CH3OH+H
Azepan-1-Yl-(6-Thiophen-2-Ylpyridin-3-Yl) Methanone	3.752E-6	2.94E-5	1.6651	0.2411	-2.0523	down	LevelBi	-	287.1236	4.6876	M+H
9-Hydroxy-3,4-Dihydroacridin-1(2H)-One	0.002388	0.005144	1.6607	0.1863	-2.4244	down	LevelBi	-	214.0848	3.1428	M+H
1,2-Diphenylhydrazine	3.545E-5	0.000173	1.6638	0.3513	-1.5094	down	LevelBi	-	217.1321	2.9844	M+CH3OH+H
Epothilone C	6.231E-6	4.41E-5	1.6625	0.1176	-3.0881	down	LevelBii	HMDB0251875	536.2702	2.6042	M+Hac-H
Kaempferol-7-O-Glucoside	2.985E-5	0.0001556	1.6639	0.3683	-1.4411	down	LevelBi	-	447.0909	4.1669	M-H
7Beta-Hydroxy-7-Desacetoxykhivorinic Acid, Methyl Ester	0.0001095	0.000358	1.6587	0.0977	-3.3562	down	LevelBi	-	571.2377	4.8627	M+Cl
Ethyl Linoleate	0.0006251	0.001603	1.6636	0.5449	-0.8758	down	LevelBi	HMDB0252048	309.2774	8.4642	M+H
Ouabain	0.0001242	0.0003999	1.6635	0.1754	-2.511	down	LevelBii	HMDB0015224	549.2719	3.3902	M+H-2H2O
Ethyl Acrylate	7.48E-6	5.139E-5	1.6603	0.2887	-1.7926	down	LevelBi	HMDB0033978	101.0593	2.2706	M+H

Methyl (2S)-2-Amino-6-[(Tert-Butoxycarbonyl)Amino]Hexanoate	2.638E-7	3.801E-6	1.6595	0.2648	-1.9172	down	LevelBi	-	243.1699	2.6467	M+H-H2O
Salmeterol	1.553E-8	4.685E-7	1.6582	0.2231	-2.1642	down	LevelBi	HMDB0015073	414.2692	2.1297	M-H
N2-(3-Hydroxysuccinoyl)Arginine	6.029E-6	4.303E-5	1.6567	0.2656	-1.9125	down	LevelBii	HMDB0032765	323.156	3.9219	M+CH3OH+H
Cdp-Dg(Lte4/I-14:0)	1.152E-5	7.281E-5	1.6584	0.3208	-1.6405	down	LevelBii	HMDB0293501	1167.4993	5.6368	M-H2O-H, M+Hac-H
Dansylglutamyl-Glycyl-Arginine Chloromethyl Ketone	7.055E-9	2.666E-7	1.6527	1.9044	0.9294	up	LevelBii	HMDB0250847	670.2049	0.727	M+FA-H
Pyroglutamylthreonine	2.934E-6	2.424E-5	1.6513	0.2379	-2.0718	down	LevelBii	HMDB0341387	211.0713	2.1297	M-H2O-H
1,2,3,4-Tetrahydro-6-Propanoylpyridine	2.134E-8	5.894E-7	1.6491	0.1477	-2.7594	down	LevelBii	HMDB0034884	198.1124	4.38	M+Hac-H
Phe-Tyr	2.332E-5	0.0001277	1.6449	0.3939	-1.344	down	LevelBi	HMDB0256384	329.1486	2.5772	M+H
Val-Val-Ile	0.0007299	0.001831	1.6433	0.3505	-1.5126	down	LevelBi	-	330.2374	3.133	M+H
Kudzusaponin Sa2	2.585E-5	0.0001385	1.6479	0.3473	-1.5258	down	LevelBii	HMDB0041241	979.472	5.2227	M+Cl
Thymine	6.795E-9	2.602E-7	1.6436	0.2616	-1.9347	down	LevelBi	HMDB0000262;HMDB0010337;HMDB0000262;PW_C000179	127.0491	1.9821	M+H
Ethyl Arachidonate	1.043E-5	6.722E-5	1.6413	0.4151	-1.2685	down	LevelBi	HMDB0062725	333.2781	9.207	M+H
Lpg(20:5)	1.345E-6	1.318E-5	1.6409	0.2092	-2.2573	down	LevelBi	LMGP04050030	575.2677	3.48	M+FA-H
Leu-Ala	0.0001082	0.0003549	1.6375	0.3635	-1.4601	down	LevelBi	HMDB0028922	201.1236	1.9592	M-H
Asp-Gly-Leu	8.24E-5	0.0002818	1.6408	0.3611	-1.4694	down	LevelBi	-	304.1478	2.5069	M+H
Ala-Val	0.0002137	0.0006375	1.6358	0.3935	-1.3455	down	LevelBi	HMDB0028700	189.1227	1.2356	M+H

Diisobutyl Cyclohexane-1,2-Dicarboxylate	3.974E-6	3.07E-5	1.6367	0.2035	-2.2971	down	LevelBi	-	348.2121	2.7064	M+ACN+Na
Ile-Glu-Phe	5.208E-8	1.14E-6	1.6367	0.37	-1.4345	down	LevelBi	-	408.2102	3.4686	M+H
Trp-Asp-Asp	2.302E-9	1.243E-7	1.638	0.3689	-1.4386	down	LevelBi	-	435.1488	2.2607	M+H
N-Acetylhistamine	1.347E-5	8.225E-5	1.6327	0.3024	-1.7254	down	LevelBii	HMDB0013253	154.0966	1.0886	M+H
Leucic Acid	3.998E-8	9.412E-7	1.6342	0.0422	-4.5671	down	LevelBii	HMDB0000624	113.0599	5.3422	M-H2O-H
2-Deoxy-2,3-Dehydro-N-Acetyl-Neuraminic Acid	1.244E-6	1.241E-5	1.6328	0.4365	-1.1959	down	LevelBi	HMDB0245680	314.0825	0.7467	M+Na
7,8-Epoxydihydrogrindelic Acid	1.142E-6	1.158E-5	1.6349	0.0322	-4.9588	down	LevelBi	-	337.236	5.1952	M+H
Posatirelin	0.0002914	0.0008308	1.628	0.0808	-3.6297	down	LevelBii	HMDB0256710	385.2421	2.3402	M+CH3OH+H
Val-Ile-Leu	4.906E-5	0.0001809	1.6288	0.3255	-1.6194	down	LevelBi	-	344.253	3.4	M+H
N-Acetyl-L-Methionine	0.0001403	0.0004434	1.6283	0.3659	-1.4506	down	LevelBi	HMDB0011745	192.0677	2.5772	M+H
Bezitramide	7.97E-5	0.0002735	1.63	0.1446	-2.7898	down	LevelBii	HMDB0249171	534.2895	3.2418	M+ACN+H
Pro-Met	2.077E-7	3.21E-6	1.6304	0.4419	-1.1781	down	LevelBi	HMDB0029023	247.11	2.0512	M+H
Etodolac Acyl Glucuronide	7.909E-7	8.677E-6	1.6307	0.3228	-1.6312	down	LevelBii	HMDB0060916	484.1544	2.463	M+Na-2H
N-Eicosapentaenoyl Aspartic Acid	0.002682	0.005706	1.6362	0.2661	-1.9098	down	LevelBii	HMDB0242067	438.2292	5.4712	M+Na-2H
4-(2,2,6,6-Tetramethyl-1-Oxylpiperidin-4-Ylamino)-4'-Demethylepipodophyllotoxin	4.24E-7	5.46E-6	1.6281	0.5269	-0.9243	down	LevelBii	HMDB0252920	537.2621	4.7364	M+H-H2O
N2-Gamma-Glutamylglutamine	1.687E-9	1.031E-7	1.625	0.2049	-2.2868	down	LevelBii	HMDB0011738	274.1029	0.727	M-H
Pravastatin	1.634E-5	9.562E-5	1.6262	0.2314	-2.1115	down	LevelBi	HMDB0005022	483.2561	5.9789	M+Hac-H

							i				
6-(1-Naphthyl)-5-Nitro-2-Piperidinone	3.173E-6	2.582E-5	1.6201	0.3583	-1.481	down	LevelBi	-	325.1183	3.4686	M+CH3OH+Na
Mgdg(O-22:5/2:0)	4.391E-5	0.000173	1.6261	0.4275	-1.2262	down	LevelBi	-	595.3806	7.0504	M+H
2'-Guanylic Acid	2.682E-6	2.259E-5	1.6227	0.2558	-1.9669	down	LevelBi	HMDB0240586	364.0632	2.0116	M+H
Cefadroxil	5.769E-9	2.327E-7	1.6212	3.0601	1.6136	up	LevelBii	HMDB0015271	427.1058	0.776	M+ACN+Na
Aconitic Acid	6.383E-8	1.317E-6	1.6202	0.1742	-2.5215	down	LevelBi	HMDB0247961	173.0079	1.9403	M-H
Leukotriene E4	0.001048	0.002507	1.6222	3.0617	1.6143	up	LevelBii	HMDB0002200	474.2058	5.8865	M+Cl
N-(5-Aminopentyl)Acetamide	5.544E-7	6.657E-6	1.619	0.4073	-1.2958	down	LevelBi	HMDB0002284	145.1329	1.1771	M+H
Dg(5-Iso Pgf2Vi/18:0/0:0)	4.457E-7	5.67E-6	1.6189	0.3234	-1.6284	down	LevelBii	HMDB0295904	701.4745	8.88	M+Cl
Nifedipine	1.651E-6	1.547E-5	1.6197	0.3783	-1.4024	down	LevelBi	HMDB0015247;PW_C009534	347.1219	2.905	M+H
16Alpha-Fluoroestradiol	5.318E-6	3.894E-5	1.6185	0.2474	-2.0149	down	LevelBii	HMDB0244757	325.1388	2.1105	M+Cl
Deoxypyridinoline	1.291E-7	2.225E-6	1.6173	0.4175	-1.2601	down	LevelBii	HMDB0000569	413.2015	2.0216	M+H
2-[4-(Dimethylamino)Phenyl]-1,2,3,4-Tetrahydroquinazolin-4-One	4.258E-6	3.24E-5	1.6171	0.2675	-1.9025	down	LevelBi	-	268.144	5.1367	M+H
Leu-Arg	0.0003047	0.000863	1.6105	0.37	-1.4343	down	LevelBi	HMDB0028923	288.2019	1.2454	M+H
(S)-2-((2-Hydroxy-2-Phenylethyl)Amino)-2-Methylpropan-1-Ol	1.161E-6	1.175E-5	1.6145	0.0776	-3.6871	down	LevelBi	-	242.1747	3.9416	M+CH3OH+H
Atorvastatin	2.133E-6	1.888E-5	1.614	0.122	-3.0353	down	LevelBii	HMDB0005006	539.232	4.1298	M-H2O-H
Magnolol	0.001057	0.002527	1.6206	0.2158	-2.2123	down	LevelBi	HMDB0254305	265.1217	7.0719	M-H
Matairesinol	0.0008493	0.00209	1.6148	0.3032	-1.7216	down	LevelBii	HMDB0035698	357.1324	4.2873	M-H
Delta-Valerolactone	1.33	1.31	1.6099	0.44	-1.157	dow	Lev	HMDB0250981	199.	4.7981	2M-H

	9E-6	4E-5		83	6	n	elBii		0967		
Kalkitoxin	0.001081	0.002575	1.6176	0.0596	-4.0676	down	LevelBi	-	367.2795	7.8804	M+H
2-(2-Hydroxyethylamino)-1-Phenylethanol	3.066E-6	2.513E-5	1.607	0.2739	-1.8681	down	LevelBi	-	199.1433	2.1212	M+NH4
Fahfa(20:3/22:3)	0.002429	0.005219	1.6054	0.3608	-1.4708	down	LevelBi	-	637.5167	8.9731	M-H
N-(1-Deoxy-1-Fructosyl)Phenylalanine	1.499E-5	8.916E-5	1.6047	0.4413	-1.1803	down	LevelBii	HMDB0037846	326.1233	2.1593	M-H
Ilekudinchoside D	0.0007706	0.001918	1.6154	4.0528	2.0189	up	LevelBi	-	801.4466	4.2174	M+CH3OH+Na
Leu-Val-His	4.648E-6	3.487E-5	1.6056	0.4582	-1.126	down	LevelBi	-	368.2266	1.8827	M+H
Avenacoside A	9.984E-5	0.0003318	1.6117	0.374	-1.4189	down	LevelBii	HMDB0033815	1107.5183	7.2847	M+FA-H
N-Stearoyl Cysteine	2.038E-5	0.0001146	1.6061	0.672	-0.5735	down	LevelBii	HMDB0241940	370.276	8.843	M+H-H2O
Kaempferol 3-O-Alpha-L-Rhamnofuranoside	0.0001408	0.0004448	1.6118	0.2081	-2.2649	down	LevelBii	HMDB0040542	431.0953	4.5003	M-H
De-O-Methylsimmondsin	7.316E-6	5.041E-5	1.6072	0.3713	-1.4294	down	LevelBii	HMDB0041208	360.1281	1.912	M-H
Paliperidone	1.187E-7	2.081E-6	1.6051	0.2194	-2.1881	down	LevelBii	HMDB0015396	447.1824	2.0631	M+Na-2H
Pip(Pgf2Alpha/22:4(10Z,13Z,16Z,19Z))	9.634E-5	0.0003218	1.5991	0.146	-2.7764	down	LevelBii	HMDB0280418	1085.4952	4.1484	M+Na-2H
Salidroside	1.399E-5	8.459E-5	1.6024	0.1059	-3.2386	down	LevelBii	HMDB0257463	359.1342	3.7218	M+Hac-H
Chenodeoxycholylaspartic Acid	7.951E-5	0.0002729	1.5973	0.0958	-3.3831	down	LevelBii	HMDB0242402	552.3135	2.3969	M+FA-H
Garcimangosone C	1.965E-6	1.771E-5	1.5968	0.1035	-3.272	down	LevelBii	HMDB0036984	471.1686	2.5194	M+Hac-H
Leu-Ile-Thr	0.00	0.00	1.5954	0.42	-1.225	dow	Lev	-	346.	2.4679	M+H

	02246	06643		77	5	n	elBi		2318		
5-Methoxy-6-(2-Propenyl)-1,3-Benzodioxole	1.064E-8	3.563E-7	1.5966	0.1436	-2.7999	down	LevelBii	HMDB0303894	227.0482	2.9322	M+Cl
3-O-Methylkaempferol	0.0003117	0.0008806	1.6002	0.0884	-3.4991	down	LevelBii	HMDB0302564	299.0545	5.1673	M-H
Multinoside A	1.706E-6	1.591E-5	1.5958	0.2307	-2.1156	down	LevelBii	HMDB0037935	609.1427	3.9998	M-H
Fa(20:3)	0.0007005	0.001768	1.5898	0.4459	-1.1653	down	LevelBi	-	611.5024	8.88	2M-H
Dopa 3'-Glucoside	4.077E-8	9.566E-7	1.5932	0.3954	-1.3386	down	LevelBii	HMDB0029452	394.0921	2.5665	M+Cl
Trp-Ser	3.318E-7	4.53E-6	1.5922	0.1489	-2.7475	down	LevelBii	HMDB0029092	336.1183	2.6326	M+FA-H
11'-Carboxy-Gamma-Chromanol	2.39E-7	3.524E-6	1.5919	0.1881	-2.4105	down	LevelBii	HMDB0012517	403.2816	9.2981	M-H
Diprotin A	1.749E-6	1.623E-5	1.5877	0.1884	-2.4081	down	LevelBi	HMDB0251473	340.2227	3.3296	M-H
6-Chloro-2,3,4,9-Tetrahydro-1H-Carbazole-1-Carboxamide	1.07E-8	3.564E-7	1.5884	0.573	-0.8033	down	LevelBii	HMDB0246699	541.143	3.7495	2M+FA-H
Kinetensin 4-7	0.001502	0.003446	1.592	4.1472	2.0522	up	LevelBii	HMDB0012986	552.2739	6.5729	M-H2O-H
5-Pentyltetrahydro-2-Oxo-3-Furancarboxylic Acid	4.174E-6	3.187E-5	1.5859	0.4377	-1.1921	down	LevelBii	HMDB0030989	221.0784	4.7981	M+Na-2H
Spirolide E	1.083E-7	1.963E-6	1.5887	0.2869	-1.8015	down	LevelBii	HMDB0030494	760.4606	8.5851	M+Cl
N-Eicosapentaenoyl Tryptophan	0.002131	0.004654	1.5865	0.4369	-1.1948	down	LevelBii	HMDB0242081	530.3381	8.9906	M+ACN+H
Difructose Anhydride Iii	2.083E-7	3.213E-6	1.5858	0.3606	-1.4716	down	LevelBii	HMDB0251271	345.0781	2.2638	M+Na-2H
Ethylestrenol	0.00	0.00	1.5873	0.43	-1.198	dow	Lev	HMDB0252076	271.	6.4371	M+H-H2O

	0107	03513		58	2	n	elBii		2408		
Ala-Ile-Leu	3.793E-5	0.000173	1.5749	0.3404	-1.5548	down	LevelBi	-	316.222	3.4588	M+H
N-Fructosyl Tyrosine	1.388E-7	2.341E-6	1.5752	0.4722	-1.0824	down	LevelBi	-	344.1321	1.8729	M+H
Withangulatin A	1.628E-6	1.535E-5	1.5752	2.1099	1.0772	up	LevelBii	HMDB0037383	527.2627	2.8357	M+H
Serofendic Acid	6.058E-6	4.315E-5	1.5752	0.2521	-1.9878	down	LevelBii	HMDB0258253	417.1866	5.8587	M+Cl
2,4,6-Phenanthrenetriol 2-O-B-D-Glucoside	5.891E-8	1.248E-6	1.5728	2.097	1.0683	up	LevelBii	HMDB0036335	387.1121	0.7365	M-H
4-O-Beta-D-Glucosyl-4-Coumaric Acid	5.951E-8	1.253E-6	1.5747	0.0174	-5.8429	down	LevelBi	HMDB0039509	344.1329	2.7562	M+NH4
Fahfa(22:4/22:3)	0.0008862	0.002166	1.5692	0.4445	-1.1698	down	LevelBi	-	663.5327	8.9824	M-H
Cenisertib	0.0003988	0.001093	1.5782	8.5597	3.0976	up	LevelBii	HMDB0249799	452.253	2.8357	M+H
Leu-Phe	7.33E-7	8.185E-6	1.5693	0.3814	-1.3905	down	LevelBi	HMDB0302841	261.1585	4.8536	M+H-H2O
4-Methyl-2-Phenylpyridine	1.153E-7	2.047E-6	1.5701	0.5559	-0.8472	down	LevelBi	-	170.096	3.0142	M+H
(7E,10E,13E,16E)-3-Hydroxydocosa-7,10,13,16-Tetraenoylcarnitine	0.0001059	0.0003481	1.573	0.3814	-1.3905	down	LevelBii	HMDB0241607	546.3722	6.729	M+CH3OH+Na
Val-Tyr-Gly	2.09E-5	0.000117	1.5656	0.5186	-0.9472	down	LevelBi	-	338.1703	2.1311	M+H
N,N-Diallyl-N'-Phenylthiourea	0.008784	0.01623	1.5754	3.7774	1.9174	up	LevelBi	-	274.1368	3.2322	M+ACN+H
Uridine, 2'-Deoxy-2'-Fluoro-2'-Methyl-, (2'R)-	2.402E-6	2.073E-5	1.5676	0.448	-1.1583	down	LevelBii	HMDB0247529	305.0799	2.3304	M+FA-H
Cdp-Dg(Lte4/I-18:0)	0.002519	0.005393	1.5612	0.5194	-0.945	down	LevelBii	HMDB0293917	1145.5212	5.1303	M-H2O-H, M+Na-2H
Trp-Trp	9.406E-6	6.18E-5	1.5635	0.3301	-1.5992	down	LevelBi	HMDB0029094	391.1739	3.804	M+H
Liquiritin	0.000367	0.00102	1.5617	1.908	0.9321	up	LevelBi	HMDB0029520	383.1139	0.6692	M+H-2H2O

	2						i				
Quinovose	3.116E-8	7.743E-7	1.5637	1.7369	0.7965	up	LevelBii	HMDB0000849	145.05	2.0252	M-H2O-H
2,6,6-Trimethyl-3-Oxocyclohexene-1-Carboxylic Acid	6.409E-7	7.375E-6	1.5637	0.2994	-1.7399	down	LevelBi	-	183.1003	4.7951	M+H
Met-Leu-Phe	1.69E-6	1.578E-5	1.5609	0.2957	-1.7577	down	LevelBii	HMDB0254492	410.2085	3.7746	M+H
(2-((3,5-Di-T-Butyl-4-Hydroxyphenyl)Thio)-1-Methylpropoxy)Acetic Acid	0.0003926	0.00108	1.5669	0.3071	-1.7033	down	LevelBii	HMDB0242455	413.2015	2.082	M+FA-H
Convalloside	0.001451	0.003344	1.5603	0.3522	-1.5056	down	LevelBii	HMDB0034195	711.3171	6.2492	M-H
Wikstromol	1.481E-6	1.432E-5	1.5626	0.165	-2.5993	down	LevelBii	HMDB0030598	355.116	4.2596	M-H2O-H
Dihydroxyphenylacetic Acid Sulfate	0.00038882	0.001069	1.5621	2.4442	1.2893	up	LevelBii	HMDB0304904	246.9906	2.1688	M-H
Pro-Leu-Pro	2.617E-7	3.785E-6	1.5572	0.6423	-0.6388	down	LevelBi	-	326.2059	2.1814	M+H
Gingerglycolipid A	1.095E-7	1.983E-6	1.5589	0.4039	-1.3079	down	LevelBii	HMDB0041093	675.3567	5.4988	M-H
Phe-Met	0.001174	0.002772	1.5499	0.3067	-1.7049	down	LevelBi	HMDB0029001	297.1254	2.6965	M+H
Pi(6 Keto-Pgf1Alpha/18:3(6Z,9Z,12Z))	0.006516	0.01249	1.5646	0.3288	-1.6046	down	LevelBii	HMDB0276784	981.466	6.6929	M+Cl
Borrelidin	4.472E-5	0.000173	1.5503	0.2091	-2.2579	down	LevelBii	HMDB0341293	472.3101	3.735	M+H-H2O
Ibritumomab Tiuxetan	5.756E-6	4.144E-5	1.5529	0.2023	-2.3056	down	LevelBii	HMDB0253331	570.2407	4.6482	M+H
8-(3-Oxo-2-(Pent-2-Enyl) Cyclopentenyl)Octanoic Acid	5.782E-6	4.158E-5	1.5554	0.4561	-1.1327	down	LevelBi	-	293.2096	5.7323	M+H
2-Naphthyl Laurate	2.594E-7	3.76E-6	1.553	0.707	-0.5003	down	LevelBi	-	344.261	8.6115	M+NH4
Gentian Violet Cation	0.00044044	0.001193	1.5446	0.0132	-6.2468	down	LevelBi	-	373.2528	2.0512	M+H

Pgp(Txb2/I-18:0)	0.0001024	0.0003389	1.5532	0.3093	-1.693	down	LevelBii	HMDB0275447	979.4694	5.6646	M+Cl
Cinnamyl Anthranilate	1.102E-5	7.015E-5	1.5522	0.1956	-2.3543	down	LevelBii	HMDB0041319	312.1218	4.4169	M+Hac-H
Pe(O-12:0/20:4)	5.245E-7	6.404E-6	1.5509	0.5147	-0.9581	down	LevelBi	-	668.4662	8.6598	M-H
2-(4-(2-Carboxyethyl)Phenethylamino)-5'-N-Ethylcarboxamidoadenosine	1.705E-5	9.893E-5	1.5455	0.0709	-3.819	down	LevelBii	HMDB0249866	480.2007	2.2638	M-H2O-H
Ala-Met	1.127E-8	3.678E-7	1.549	0.4308	-1.2148	down	LevelBi	HMDB0028693	221.0938	2.0314	M+H
4-(3-Aminophenyl)-2-Methyl-3-Butyn-2-Ol	1.334E-8	4.127E-7	1.5479	0.5804	-0.7848	down	LevelBi	-	158.0959	3.0042	M+H-H2O
1,2-Dioctanoylglycerol	0.0001824	0.0005559	1.5496	0.2183	-2.1955	down	LevelBii	HMDB0244086	403.2683	6.7394	M+Hac-H
Vellein	0.0001041	0.0003438	1.5452	0.2	-2.3219	down	LevelBii	HMDB0303360	413.1191	3.6936	M+Na-2H
Arg-Pro-Pro	4.983E-7	6.174E-6	1.5463	0.3406	-1.5539	down	LevelBi	-	373.1943	5.7323	M+Na-H2O
Phe-Leu-Ile	0.0009882	0.00238	1.5428	0.2491	-2.0054	down	LevelBi	-	392.2519	4.1879	M+H
Val-Met	0.0003141	0.0008866	1.5373	0.4427	-1.1755	down	LevelBii	HMDB0029133	247.1113	2.1593	M-H
Cdp-Dg(6 Keto-Pgf1Alpha/18:2(9Z,11Z))	0.0003574	0.0009942	1.5367	0.356	-1.49	down	LevelBii	HMDB0291312	1074.5159	3.0238	M+H-H2O
Asn-Ile	5.838E-9	2.348E-7	1.539	0.3565	-1.4881	down	LevelBii	HMDB0028734	246.1444	1.9424	M+H
16-Hydroxyhexadecanoylcarnitine	0.002701	0.005743	1.5418	2.3138	1.2103	up	LevelBii	HMDB0241458	398.3247	6.9441	M+H-H2O
Ala-Ile-Phe	0.000348	0.0009716	1.533	0.3346	-1.5796	down	LevelBi	-	350.206	3.5078	M+H
Milbemycin Beta3	7.102E-7	8.018E-6	1.5398	0.1179	-3.085	down	LevelBii	HMDB0254729	515.2805	2.3208	M+Na-2H

Calenduloside H Methyl Ester	0.0006969	0.00176	1.545	0.2085	-2.2621	down	LevelBii	HMDB0041021	1005.4864	7.2568	M+Cl
Pyropheophorbide A	5.393E-5	0.0001946	1.5427	0.3921	-1.3508	down	LevelBii	HMDB0256993	1113.5288	5.8032	2M+FA-H
Phe-Val-Phe	0.0005795	0.001502	1.535	0.0678	-3.8823	down	LevelBi	-	412.2207	4.0303	M+H
Scillipheosidin 3-[Glucosyl-(1->2)-Rhamnoside]	1.938E-6	1.756E-5	1.5376	0.2876	-1.7978	down	LevelBii	HMDB0036946	743.3083	4.3522	M+Cl
Delimotecan	0.00149	0.003422	1.5396	3.474	1.7966	up	LevelBii	HMDB0250970	655.2244	2.3969	M+Cl
Tobramycin	1.297E-5	7.989E-5	1.5364	0.251	-1.9945	down	LevelBii	HMDB0014822	448.2445	3.5833	M-H2O-H
4-Hydroxyphenyl-2-Propionic Acid	1.177E-7	2.071E-6	1.5357	0.0503	-4.3145	down	LevelBii	HMDB0041683	165.0544	5.0198	M-H
Phosphorylcholine	5.754E-9	2.327E-7	1.533	0.117	-3.0955	down	LevelBii	HMDB0001565	165.0544	4.4909	M-H2O-H
Val-Pro-Pro	1.127E-8	3.678E-7	1.5318	0.6307	-0.665	down	LevelBi	HMDB0253034	312.1903	2.1112	M+H
Trp-Asp	8.543E-5	0.0002907	1.5317	0.246	-2.0233	down	LevelBii	HMDB0029079	318.1081	2.1879	M-H
Histamine	2.635E-9	1.332E-7	1.5325	0.5555	-0.8482	down	LevelBi	HMDB0000870;PW_C000693	112.0865	0.6298	M+H
Annonacin	0.0004949	0.001319	1.5378	0.2265	-2.1425	down	LevelBii	HMDB0033327	617.4354	9.3543	M+Na-2H
19-Oxoandrost-4-Ene-3,17-Dione	0.004009	0.008127	1.5311	0.2581	-1.9542	down	LevelBii	HMDB0006768	645.3448	6.5175	2M+FA-H
Val-Pro-Gly-Val-Gly	1.371E-6	1.338E-5	1.5278	0.4083	-1.2923	down	LevelBii	HMDB0259877	472.239	2.3114	M+FA-H
Tyr-Pro-Leu	8.7E-5	0.0002953	1.5229	0.49	-1.0291	down	LevelBi	-	392.2158	2.9645	M+H
N-Phenethylacetamide	6.36	0.00	1.5286	0.35	-1.494	dow	Lev	HMDB0255222	164.	4.1783	M+H

	9E-5	02244		5	1	n	elBi		1061		
Ile-Val-Asp	8.496E-6	5.699E-5	1.5277	0.3543	-1.4971	down	LevelBi	-	346.1965	5.029	M+H
Tangeraxanthin	0.001458	0.003358	1.5249	7.7333	2.9511	up	LevelBii	HMDB0039015	505.3056	8.3635	M+Na-2H
Deacetylchloronectrin	1.374E-7	2.325E-6	1.5244	0.5647	-0.8243	down	LevelBi	-	423.1967	2.0512	M+H
Pro-Ser	9.21E-8	1.731E-6	1.5245	0.3758	-1.4119	down	LevelBi	HMDB0029026	203.1014	0.7273	M+H
Benzodioxole Fentanyl	2.813E-10	3.531E-8	1.5248	0.0304	-5.0382	down	LevelBi	-	429.2157	3.1032	M+H
Tryptophan N-Glucoside	1.129E-6	1.148E-5	1.5244	0.4995	-1.0013	down	LevelBii	HMDB0341443	367.1483	2.3402	M+H
Misoprostol Acid	0.001257	0.002942	1.5289	0.3139	-1.6718	down	LevelBii	HMDB0254756	389.2282	7.6204	M+Na-2H
N-[(5-Hydroxy-2-Pyridinyl)Methyl]Adenosine	0.0011143	0.0037233	1.5221	0.3845	-1.3788	down	LevelBii	HMDB0040912	397.1227	4.2569	M+Na
N-Biotinyl-3,6-Dioxaoctane-1,8-Diamine	6.435E-5	0.00022265	1.5158	0.3751	-1.4147	down	LevelBi	-	429.2153	3.804	M+CH3OH+Na
Leu-Asp-Phe	0.00337	0.006991	1.5138	0.3632	-1.4613	down	LevelBi	-	394.1962	3.4196	M+H
Mangostanol	1.161E-5	7.318E-5	1.5181	0.3118	-1.6811	down	LevelBii	HMDB0029868	461.1379	2.576	M+Cl
(2Z,4'Z)-2-(5-Methylthio-4-Penten-2-Ynylidene)-1,6-Dioxaspiro[4.4]Non-3-Ene	6.457E-7	7.407E-6	1.5178	0.3039	-1.7186	down	LevelBii	HMDB0032660	234.0717	3.0238	M+
Agavasaponin C	0.0038007	0.001051	1.5222	0.4703	-1.0884	down	LevelBii	HMDB0033353	917.4671	6.9051	M+H
Adipate Semialdehyde	3.03E-6	2.489E-5	1.5155	0.349	-1.5186	down	LevelBii	HMDB0012882	151.0389	2.9979	M+Na-2H
Val-Ala	1.102E-5	7.015E-5	1.5143	0.3056	-1.7105	down	LevelBii	HMDB0029120	187.1076	0.7365	M-H
Cyclocalamin	3.51	4.73	1.5177	0.60	-0.717	dow	Lev	HMDB0035863	537.	2.1688	M+Cl

	4E-7	8E-6		82	3	n	elBi i		1916		
N-Arachidonoyl Alanine	8.447E-6	5.669E-5	1.5145	0.5949	-0.7493	down	LevelBii	HMDB0241989	358.276	8.2812	M+H-H2O
Glycerophosphocholine	3.985E-7	5.198E-6	1.5141	1.6959	0.7621	up	LevelBi	HMDB0000086;PW_C000059	258.1092	0.6595	M+H
Thr-Ile-Ile	6.346E-6	4.48E-5	1.5101	0.3228	-1.6314	down	LevelBi	-	346.2322	3.1825	M+H
2-(1,2,3,4-Tetrahydroxybutyl)-6-(2,3,4-Trihydroxybutyl)Pyrazine	6.047E-9	2.388E-7	1.5119	0.3675	-1.4441	down	LevelBii	HMDB0034894	349.1248	3.5365	M+FA-H
Fahfa(18:3/3:0)	0.001843	0.00411	1.509	0.4434	-1.1734	down	LevelBi	-	349.2363	8.5663	M-H
N-(3,4-Difluorophenyl)-3-Oxobutanamide	7.019E-7	7.943E-6	1.5085	0.4009	-1.3187	down	LevelBi	-	231.0929	1.2356	M+NH4
(E)-Ethenoxyimino-Oxido-Pyrrolidin-1-Ylazanium	5.538E-5	0.0001991	1.5103	0.4809	-1.0561	down	LevelBi	-	175.1179	0.6595	M+NH4
Thr-Gln-Lys	0.0009459	0.002289	1.5052	0.4195	-1.2531	down	LevelBi	-	376.2208	2.7262	M+H
Thr-Val	5.745E-7	6.814E-6	1.5076	0.4123	-1.2782	down	LevelBii	HMDB0029074	217.1187	1.1097	M-H
N-Arachidonoyl Dopamine	2.329E-5	0.0001276	1.503	0.6951	-0.5246	down	LevelBi	HMDB0243624;LMFA08020084	457.3435	9.2648	M+NH4
1-Nitro-5-Glutathionyl-6-Hydroxy-5,6-Dihydronaphthalene	1.318E-5	8.09E-5	1.5007	2.0132	1.0095	up	LevelBii	HMDB0060326	531.0914	1.6973	M+Cl
Gly-Ser-Lys	9.271E-6	6.11E-5	1.5018	0.3831	-1.3841	down	LevelBi	-	255.1426	1.8827	M+H-2H2O
Val-Pro	2.731E-9	1.362E-7	1.5026	0.6767	-0.5635	down	LevelBi	HMDB0029135	215.1386	2.1012	M+H
Lippioside Ii	1.389E-7	2.341E-6	1.5035	0.4205	-1.2498	down	LevelBii	HMDB0034268	589.1278	2.5476	M+Cl
6-Hydroxypseudooxynicotine	1.328E-7	2.278E-6	1.5005	0.2767	-1.8536	down	LevelBii	HMDB0240264	253.1187	2.2353	M+Hac-H
Pe-Cer(12:2_2O/12:0)	0.004593	0.009162	1.4896	0.151	-2.7274	down	LevelBi	-	1035.6996	9.5133	M+Cl, 2M-H

1'-O-Acetylpaxilline	5.912E-7	6.976E-6	1.4992	0.6076	-0.7189	down	LevelBii	HMDB0037530	478.2622	2.1616	M+H
Isocucurbitacin B	0.0030188	0.0085633	1.5049	2.2079	1.1427	up	LevelBi	-	581.3057	4.1189	M+Na
Mg(Pgf2Alpha/0:0/0:0)	0.0018896	0.0057552	1.4964	0.4415	-1.1797	down	LevelBii	HMDB0260478	433.2546	3.0833	M+Na-H2O
(2-Anilino-4-Methyl-1,3-Thiazol-5-Yl)(1-Benzothiophen-2-Yl)Methanone	1.52E-7	2.515E-6	1.4941	0.2367	-2.079	down	LevelBi	-	389.0175	0.5895	M+K
Ile-Val-Leu	0.0014774	0.0046266	1.4902	0.5415	-0.8849	down	LevelBi	-	344.2533	3.5475	M+H
Ethyl 4-Isothiocyanatopiperidine-1-Carboxylate	2.543E-6	2.172E-5	1.493	0.1699	-2.5572	down	LevelBi	-	237.0683	0.5895	M+Na
N-Cyclopropyl-4-[[1-(3,4-Dimethylbenzoyl)Piperidin-4-Yl]Methoxy]Benzamide	5.719E-7	6.799E-6	1.4952	0.5218	-0.9384	down	LevelBi	-	407.2367	2.4872	M+H
N(Epsilon)-(Carboxymethyl)Hydroxylysine	9.324E-7	9.845E-6	1.4919	0.146	-2.7758	down	LevelBii	HMDB0250375	201.0877	2.0063	M-H2O-H
Valorphin	2.548E-6	2.174E-5	1.4954	3.7067	1.8901	up	LevelBii	HMDB0059789	927.4112	2.7461	M+Cl
3,4-Dihydro-2H-1-Benzopyran-2-One	7.901E-7	8.674E-6	1.4949	0.145	-2.7854	down	LevelBii	HMDB0036626	193.0494	3.9066	M+FA-H
O-Oxalylhomoserine	4.524E-8	1.025E-6	1.4916	0.3097	-1.6909	down	LevelBii	HMDB0302143	250.0563	1.9309	M+Hac-H
Quercetin 3-O-Glucoside	0.0012114	0.0039244	1.4935	0.1116	-3.1637	down	LevelBi	HMDB0037362	463.0876	3.8321	M-H
Val-Ile-Thr	0.0014033	0.0044334	1.4861	0.5166	-0.9528	down	LevelBi	-	332.2154	2.1616	M+H
7-Methylinosine	0.0039666	0.001089	1.4896	0.3981	-1.3288	down	LevelBii	HMDB0003950	342.1176	1.8648	M+Hac-H
Phenmedipham	1.15E-7	2.044E-6	1.49	0.3315	-1.5929	down	LevelBii	HMDB0256405	345.1068	2.9602	M+FA-H
N-Linoleoyl Glutamine	0.00352	0.00726	1.4854	0.3774	-1.4057	down	LevelBi	HMDB0241975	447.2588	2.2904	M+K

							i				
Leu-Ser-Pro	0.0001975	0.000595	1.4916	0.5003	-0.9991	down	LevelBi	-	316.1864	6.349	M+H
3-Chloro-N-(4-Imidazo[1,2-A]Pyrimidin-2-Ylphenyl)Benzamide	1.491E-7	2.473E-6	1.4868	0.2255	-2.1487	down	LevelBi	-	349.0883	2.7562	M+H
L-Proline	2.544E-5	0.0001371	1.4848	0.6193	-0.6912	down	LevelBi	HMDB0000162;PW_C000106	116.0699	2.1012	M+H
Dehydroxynocardamine	1.327E-5	8.132E-5	1.4794	0.5283	-0.9204	down	LevelBi	-	585.357	2.1616	M+H
Paromomycin I	0.004199	0.008471	1.4706	0.1404	-2.8328	down	LevelBii	HMDB0242512	633.3348	4.5797	M+NH4
5-Methyl-N-(2-Oxo-3-Azepanyl)-2-(Trifluoromethyl)-3-Furansulfonamide	1.701E-6	1.587E-5	1.4773	0.2525	-1.9857	down	LevelBi	-	305.0558	0.5797	M+H-2H2O
(E)-Zeatin Glucoside	1.297E-5	7.989E-5	1.4767	0.2592	-1.9481	down	LevelBii	HMDB0303782	440.1777	2.9885	M+Hac-H
Oxytocin 1-8	1.343E-6	1.317E-5	1.4743	0.4971	-1.0085	down	LevelBii	HMDB0013033	935.3714	4.9551	M-H
Melleolide B	4.2E-6	3.205E-5	1.4719	0.3735	-1.421	down	LevelBii	HMDB0038787	413.2012	2.6136	M-H2O-H
Sordarin	4.529E-6	3.411E-5	1.4714	0.3149	-1.6669	down	LevelBii	HMDB0242737	457.2606	3.656	M+H-2H2O
(11E,13Z)-10-Hydroxyoctadeca-11,13-Dienoylcarnitine	0.0002471	0.0007208	1.4749	2.2518	1.1711	up	LevelBii	HMDB0241544	484.325	6.5452	M+FA-H
Hesperetin 7-Neohesperidoside	4.008E-10	4.333E-8	1.4713	1.7409	0.7998	up	LevelBii	HMDB0030748	652.2235	0.854	M+ACN+H
Vinblastine	0.0002417	0.0007073	1.4747	0.3485	-1.5209	down	LevelBii	HMDB0014710	845.3841	5.0198	M+Cl
Trp-Phe	8.71E-5	0.0002956	1.4646	0.5326	-0.9089	down	LevelBi	HMDB0029090	352.1642	3.735	M+H
6-(2-Hydroxyethoxy)-6-Oxohexanoic Acid	6.098E-8	1.278E-6	1.4674	0.4551	-1.1358	down	LevelBii	HMDB0061681	254.0984	3.38	M+ACN+Na
Tyr-Hyp	3.80	0.00	1.4687	0.22	-2.129	dow	Lev	HMDB0029106	339.	2.2258	M+FA-H

	4E-5	0173		85	6	n	elBii		1181		
(R)-Dihydroactinidiolide	4.06E-6	3.12E-5	1.465	0.5234	-0.9339	down	LevelBi	-	181.1216	5.3904	M+H
5-Methoxysalicylic Acid	1.303E-5	8.016E-5	1.466	0.3656	-1.4518	down	LevelBi	HMDB0001868	167.0337	3.1018	M-H
Theanine	5.771E-6	4.152E-5	1.4627	0.5054	-0.9846	down	LevelBii	HMDB0034365	173.0925	1.3148	M-H
Glutaric Acid	3.229E-6	2.615E-5	1.4602	0.321	-1.6395	down	LevelBi	HMDB0000661	131.0336	2.2353	M-H
N-Cyclopropyl-2-Quinolinecarboxamide	3.155E-7	4.356E-6	1.4631	0.4491	-1.1549	down	LevelBi	-	245.1278	3.0833	M+CH3OH+H
Lpe(24:4)	0.001995	0.004394	1.4592	0.2019	-2.308	down	LevelBi	-	556.3374	8.5289	M-H
Fleroxacin	1.489E-8	4.53E-7	1.4606	0.2352	-2.0882	down	LevelBii	HMDB0252299	368.1207	2.7176	M-H
1,4,10,13-Tetraoxa-7,16-Diazacyclooctadecane	2.241E-6	1.964E-5	1.4569	0.6075	-0.7191	down	LevelBii	HMDB0244223	263.1955	1.7269	M+H
Tyr-Val-Phe	2.676E-6	2.257E-5	1.4576	0.2385	-2.0682	down	LevelBi	-	428.215	3.4783	M+H
Protoveratrine	3.298E-8	8.083E-7	1.4588	3.7988	1.9256	up	LevelBii	HMDB0256876	838.4291	3.703	M+FA-H
4-Hydroxyretinoic Acid Glucuronide	2.408E-5	0.0001311	1.4548	0.4904	-1.0279	down	LevelBii	HMDB0061688	493.2417	2.5069	M+H
Ser-Val-Trp	1.051E-5	6.754E-5	1.4593	0.3928	-1.348	down	LevelBi	-	391.1953	3.1528	M+H
L-Aspartic Acid	0.0005915	0.001529	1.4594	0.3852	-1.3765	down	LevelBi	HMDB0000191;PW_C000123	134.0436	2.2806	M+H
Ala-Pro	1.413E-6	1.373E-5	1.4525	0.5797	-0.7865	down	LevelBi	HMDB0028695	187.1069	1.2943	M+H
Asp-Pro-Arg	0.002093	0.004583	1.451	0.4169	-1.2622	down	LevelBi	-	419.2265	3.1428	M+CH3OH+H
5-(Hydroxyphenyl)-Gamma-Valerolactone-O-Sulphate	6.22E-6	4.407E-5	1.4523	0.4395	-1.186	down	LevelBii	HMDB0059993	327.05	0.5895	M+CH3OH+Na
Gamma-L-Glutamyl-L-Pipecolic Acid	4.263E-5	0.000173	1.4499	0.1955	-2.3549	down	LevelBii	HMDB0038614	317.1337	3.1018	M+Hac-H

Desmethyldeschlorobenzoyl Indomethacin	3.024E-6	2.486E-5	1.452	0.1141	-3.1317	down	LevelBi	-	190.0499	3.2431	M-H
Glycerophosphoinositol	0.006697	0.01281	1.4591	0.1092	-3.195	down	LevelBii	HMDB0011649	333.0592	4.0091	M-H
N-(3-Methylbut-2-En-1-Yl)-9H-Purin-6-Amine	3.875E-6	3.013E-5	1.4483	0.4908	-1.0269	down	LevelBii	HMDB0245646	204.1226	2.2904	M+H
Citrulline	3.006E-9	1.464E-7	1.449	0.5356	-0.9008	down	LevelBi	HMDB0000904;PW_C000723	140.081	1.9523	M+H-2H2O
Phe-Ile-Ser	0.0004763	0.001276	1.4422	0.358	-1.4818	down	LevelBi	-	366.2013	2.4775	M+H
Soybean Saponin Bg	0.001651	0.003733	1.4467	0.4247	-1.2355	down	LevelBii	HMDB0302658	945.4665	7.3871	M+Cl
Antibiotic Sb 202742	1.492E-5	8.898E-5	1.4438	0.4409	-1.1815	down	LevelBii	HMDB0041206	405.2195	3.8134	M+Cl
Deoxyloganic Acid	4.532E-7	5.744E-6	1.4453	0.1934	-2.3705	down	LevelBii	HMDB0037028	359.133	3.9531	M-H
Ile-Val-Val	0.0034462	0.0096669	1.4422	0.3812	-1.3915	down	LevelBii	HMDB0304801	328.2226	3.1301	M-H
2-Hydroxypropyl Stearate	0.0342	0.05265	1.4563	0.1479	-2.757	down	LevelBi	HMDB0029764	343.3186	8.9216	M+H
Silodosin	8.122E-5	0.0027822	1.4417	0.2447	-2.031	down	LevelBii	HMDB0258291	516.2137	3.7495	M+Na-2H
Phenytoin Quinone	9.546E-7	1.004E-5	1.4413	0.414	-1.2722	down	LevelBii	HMDB0060868	327.0649	0.5667	M+FA-H
Destruxin-E Diol	0.0004254	0.001157	1.4393	1.9759	0.9825	up	LevelBi	-	650.3109	3.1032	M+K
Isopentyl Isopentanoate	1.898E-8	5.431E-7	1.4361	0.1521	-2.7166	down	LevelBii	HMDB0030029	231.1588	4.5557	M+Hac-H
Val-Pro-Ala	0.001145	0.00271	1.4387	0.0753	-3.7305	down	LevelBi	-	286.1769	6.2803	M+H
Trp-Ile-Asp	1.208E-5	7.532E-5	1.434	0.089	-3.4898	down	LevelBi	-	433.2071	3.1428	M+H
Pg(O-12:0/22:4)	0.00	0.00	1.4287	0.37	-1.433	dow	Lev	-	727.	8.9731	M-H

	4937	9762		02	7	n	elBi		4903		
Ganoderiol F	0.0001577	0.0004903	1.4237	0.6767	-0.5634	down	LevelBii	HMDB0038707	489.3136	9.2332	M+Cl
5-Methoxydimethyltryptamine	0.000223	0.0006601	1.4249	0.4983	-1.0049	down	LevelBii	HMDB0002004	263.139	2.463	M+FA-H
Quaternary Ammonium Chloride Combination	3.713E-7	4.937E-6	1.4283	0.4664	-1.1002	down	LevelBii	HMDB0303284	229.0987	3.1301	M+FA-H
2-Phenylethyl Acetate	0.0002197	0.0006523	1.4208	0.2505	-1.9971	down	LevelBii	HMDB0033945	209.0808	3.6564	M+FA-H
3-Dehydro-6-Deoxoteasterone	9.657E-5	0.0003223	1.4202	0.5125	-0.9644	down	LevelBii	HMDB0304120	467.3285	9.2145	M+Cl
Delprostenato	0.0001148	0.0003735	1.4178	0.2043	-2.2913	down	LevelBii	HMDB0250975	435.1536	2.491	M-H
Gln-Val-Ile	0.00147	0.003381	1.422	2.0382	1.0273	up	LevelBi	-	359.2274	2.6665	M+H
5,6-Dihydrothymine	1.76E-7	2.818E-6	1.4203	0.3355	-1.5758	down	LevelBii	HMDB0000079	187.0718	1.9403	M+Hac-H
Pgp(Lte4/I-16:0)	0.0002185	0.0006491	1.4245	0.4803	-1.058	down	LevelBii	HMDB0275235	984.4618	5.7845	M-H
Avenic Acid A	9.302E-5	0.0003127	1.4192	0.2648	-1.9173	down	LevelBii	HMDB0030416	357.1049	2.4066	M+Cl
Flavin Adenine Dinucleotide (Fad)	0.0002076	0.0006217	1.4168	2.0605	1.043	up	LevelBi	-	784.1472	2.5287	M-H
(1-(3-Oxo-2-Aza-Spiro(4.5)Dec-2-Ylmethyl)-Cyclohexyl)-Acetic Acid	0.0001068	0.0003506	1.4183	0.4091	-1.2894	down	LevelBi	-	308.2195	4.6876	M+H
N-Methyl(4-Pyrimidinyl)Methanamine	2.632E-8	6.869E-7	1.4137	1.5602	0.6417	up	LevelBi	-	124.0863	0.679	M+H
Lomefloxacin	5.012E-9	2.155E-7	1.414	2.682	1.4233	up	LevelBi	HMDB0015113	369.1758	1.0985	M+NH4
Phenylbutyric Acid	1.634E-6	1.537E-5	1.4142	0.4775	-1.0665	down	LevelBi	-	165.09	4.7951	M+H
Hophe-Lys-Oh	0.000202	0.000607	1.4076	0.1464	-2.7718	down	LevelBi	-	414.1628	3.4236	M-H

	2	7									
Chitotriose	0.0001485	0.0004659	1.4084	0.3647	-1.4552	down	LevelBii	HMDB0006578	560.2301	2.3398	M+Hac-H
Phe-Val-Gly	0.0004013	0.001099	1.4077	0.4356	-1.199	down	LevelBi	-	322.1734	2.2309	M+H
3-O-Demethylfortimicin A	3.368E-5	0.0001717	1.4161	1.9652	0.9747	up	LevelBii	HMDB0245956	446.2592	3.2813	M+CH3OH+Na
Cdp-Dg(Pge2/A-21:0)	0.004738	0.009414	1.4163	2.507	1.326	up	LevelBii	HMDB0293076	1100.5692	3.2619	M-H2O-H
Acetyl-L-Carnitine	7.382E-9	2.746E-7	1.4101	1.3844	0.4692	up	LevelBi	HMDB0000201;LMFA07070050	204.1226	0.7273	M+H
Trans-O-Coumaric Acid 2-Glucoside	0.0002586	0.0007491	1.4051	0.3369	-1.5697	down	LevelBi	HMDB0033581	325.0917	2.7555	M-H
N-Stearoyl Valine	1.673E-5	9.764E-5	1.4096	0.2045	-2.29	down	LevelBii	HMDB0241952	382.3303	9.2051	M-H
Chamaechromone	1.102E-10	2.168E-8	1.4077	1.5385	0.6215	up	LevelBii	HMDB0249879	543.1281	0.7565	M+H
Pyrodone	5.524E-7	6.645E-6	1.4067	0.3976	-1.3307	down	LevelBii	HMDB0256988	320.1844	4.7335	M+FA-H
Morphiceptin	5.297E-5	0.0001919	1.4019	0.3641	-1.4576	down	LevelBii	HMDB0005777	486.254	3.1925	M+H-2H2O
7-Hydroxyoct-3-Enoylglycine	2.416E-5	0.0001315	1.4025	0.6289	-0.6691	down	LevelBii	HMDB0094775	233.1486	1.8827	M+NH4
Roridin A	0.0003881	0.001069	1.4004	0.5243	-0.9314	down	LevelBi	-	533.2705	4.1586	M+H
Asparagylthreonyllysine	7.527E-5	0.00026	1.403	0.549	-0.8651	down	LevelBi	-	362.2045	3.123	M+H
Octanamide	0.0004349	0.00118	1.399	0.0009	-10.1269	down	LevelBii	HMDB0255903	185.1636	2.2904	M+ACN+H
Acetophenone	5.067E-8	1.116E-6	1.4032	0.561	-0.834	down	LevelBi	HMDB0033910	121.064	3.745	M+H
Val-Gln	0.00	0.00	1.3992	0.51	-0.967	dow	Lev	HMDB0029125	246.	1.1086	M+H

	01863	05661		12	9	n	elBi		1442		
Gabexate	0.0001016	0.0003368	1.3966	0.1109	-3.1726	down	LevelBii	HMDB0252569	380.18	2.1688	M+Hac-H
P-Salicylic Acid	2.389E-6	2.068E-5	1.3982	0.455	-1.1361	down	LevelBi	HMDB0000500	137.0235	2.9979	M-H
2-{[2-(1H-Indol-3-Yl)Ethyl]Ethanimidoyl}Phenol	3.894E-10	4.287E-8	1.3953	11.2266	3.4889	up	LevelBi	-	333.1545	2.4099	M+CH3OH+Na
Capsoside A	3.189E-6	2.589E-5	1.3924	0.4748	-1.0746	down	LevelBii	HMDB0037613	659.3593	2.2111	M+H-2H2O
2-Methylglutamic Acid	1.058E-7	1.927E-6	1.3947	0.5524	-0.8561	down	LevelBi	-	144.0652	2.5772	M+H-H2O
Neoeriocitrin	1.022E-6	1.057E-5	1.3952	0.3043	-1.7162	down	LevelBii	HMDB0255521	577.1529	3.5082	M-H2O-H
Verbascose	5.098E-9	2.162E-7	1.3932	1.6683	0.7384	up	LevelBii	HMDB0259784	873.2715	1.1189	M+FA-H
Pro-Pro	7.447E-6	5.122E-5	1.3936	0.5753	-0.7975	down	LevelBi	HMDB0011180	213.1222	0.737	M+H
Ethyl Beta-D-Fructofuranoside	0.007489	0.01414	1.3876	0.3126	-1.6775	down	LevelBii	HMDB0303035	229.0684	2.0063	M+Na-2H
Dg(Lte4/I-12:0/0:0)	0.000101	0.0003347	1.3923	0.5839	-0.7763	down	LevelBii	HMDB0298379	695.4447	5.8592	M+
Cyclo(Prolylvalyl)	3.663E-6	2.887E-5	1.3908	0.4633	-1.1101	down	LevelBi	-	197.1275	2.7362	M+H
10-Hydroxy-3-Methoxy-1,3,5,7-Cadinatetraen-9-One	0.0002463	0.0007189	1.3931	2.3234	1.2163	up	LevelBii	HMDB0036456	565.2841	8.6598	2M+FA-H
23-Hydroxyphysalolactone	0.0006723	0.001706	1.3914	3.7944	1.9239	up	LevelBii	HMDB0031388	613.2376	3.2242	M+Hac-H
[4-(4-Nitrophenyl)Piperazin-1-Yl](4-Propoxyphenyl)Methanone	2.162E-5	0.0001204	1.3834	0.1943	-2.3636	down	LevelBi	-	370.1748	3.2222	M+H
Linalool Oxide D 3-[Apiosyl-(1->6)-Glucoside]	0.0002014	0.0006057	1.3837	0.0218	-5.5179	down	LevelBii	HMDB0031367	485.1997	3.1396	M+Na-2H
Leu-Leu-Lys	0.01	0.03	1.3933	0.21	-2.218	dow	Lev	-	373.	2.4197	M+H

	919	21		49	3	n	elBi		2793		
Ac-Thr-D-Trp(Cho)-Phe-Nmebzl	1.289E-5	7.949E-5	1.3861	1.7738	0.8269	up	LevelBii	HMDB0257639	670.2936	2.1492	M+FA-H
Rilmakalim	4.467E-9	1.965E-7	1.3848	1.6959	0.7621	up	LevelBii	HMDB0257230	803.2611	1.0985	2M+H
Pc(Pgj2/P-18:0)	0.0002419	0.0007077	1.3803	0.3554	-1.4927	down	LevelBii	HMDB0289562	882.5816	9.2145	M+Hac-H
Pollenin A	0.001014	0.002435	1.3851	0.174	-2.523	down	LevelBii	HMDB0303704	301.0336	4.8166	M-H
Pc-M6	0.0001874	0.0005689	1.3818	2.6542	1.4083	up	LevelBii	HMDB0038568	402.247	5.0474	M-H2O-H
Trp-Ala-Asn	8.389E-6	5.637E-5	1.3767	0.4101	-1.286	down	LevelBi	-	390.1752	2.3204	M+H
Ile-Val-Gly	9.278E-6	6.112E-5	1.3799	0.6465	-0.6292	down	LevelBi	-	288.1912	2.1512	M+H
2-Amino-9-[(2R,4S,5R)-5-(Hydroperoxymethyl)-4-Hydroxyoxolan-2-Yl]-1H-Purin-6-One	2.777E-7	3.952E-6	1.3796	1.7209	0.7831	up	LevelBii	HMDB0257980	282.0831	1.9781	M-H
Pirbuterol	2.073E-6	1.847E-5	1.3807	0.293	-1.7708	down	LevelBii	HMDB0015407	275.1183	2.3019	M+Cl
Indolelactic Acid	6.012E-7	7.034E-6	1.3788	0.6345	-0.6564	down	LevelBi	HMDB0000671	204.066	3.8785	M-H
5-Hydroxyoctanoylcarnitine	3.589E-8	8.634E-7	1.3787	1.5746	0.6549	up	LevelBii	HMDB0241694	304.2114	3.656	M+H
Leu-Ile-Ala	0.001608	0.003654	1.3697	0.4843	-1.0461	down	LevelBi	-	316.2215	2.5469	M+H
Heptanohydrazide	0.001267	0.002961	1.375	0.1397	-2.8393	down	LevelBi	-	199.143	3.133	M+CH3OH+Na
Cyclolinopeptide B	0.01581	0.02715	1.3848	2.3272	1.2186	up	LevelBii	HMDB0302161	1078.587	3.2619	M+Na-2H
Ile-Ala	9.998E-5	0.0003321	1.3723	0.618	-0.6944	down	LevelBi	HMDB0028900	203.1381	1.9623	M+H
Ethyl L-Tryptophanate	5.436E-7	6.577E-6	1.3742	0.3776	-1.4051	down	LevelBi	HMDB0252047	233.1269	3.3505	M+H
Cetilistat	0.00	0.00	1.3774	0.57	-0.808	dow	Lev	HMDB0249835	382.	8.4642	M-H2O-H

	158	3599		1	4	n	elBii		2762		
6-Methyl-1-(3-Sulfanylpropanoyl)-2-Piperidinecarboxylic Acid	0.0006141	0.001579	1.3693	0.5511	-0.8596	down	LevelBi	-	295.11	3.991	M+ACN+Na
Methyl Octadecadienoate	0.01274	0.02247	1.375	0.5986	-0.7404	down	LevelBi	-	327.2882	8.4447	M+CH3OH+H
Trimetrexate	5.381E-7	6.521E-6	1.3702	0.6275	-0.6722	down	LevelBii	HMDB0015288	390.1516	3.7948	M+Na-2H
Deoxycholic Acid Glycine Conjugate	0.0012766	0.0040884	1.3718	1.9352	0.9525	up	LevelBii	HMDB0000631	484.2867	6.5729	M+Cl
Ketoleucine	0.0006301	0.001614	1.3731	0.3738	-1.4198	down	LevelBi	HMDB0000695;PW_C000547	130.0643	2.9943	M+
Hyp-Pro	0.0010288	0.0034002	1.3668	0.2885	-1.7932	down	LevelBii	HMDB0028871	227.1018	2.6609	M-H
6-Hydroxymelatonin	7.447E-5	0.0002577	1.3615	0.4128	-1.2763	down	LevelBii	HMDB0004081	497.2443	3.1925	2M+H
Phe-Ile-Asp	4.827E-5	0.0001785	1.3665	0.505	-0.9858	down	LevelBi	-	394.1952	2.6169	M+H
(2S,3S)-Nemonapride	1.761E-5	0.0001016	1.366	0.3325	-1.5884	down	LevelBii	HMDB0255510	388.182	2.9943	M+H
Anserine	2.611E-10	3.409E-8	1.3663	1.3854	0.4703	up	LevelBi	HMDB0000194;PW_C000126	241.1288	0.679	M+H
Leu-Ile-Gly	3.242E-5	0.0001665	1.3632	0.5403	-0.8883	down	LevelBi	-	302.2059	2.3602	M+H
N-Cycloheptyl-1-[2-(4-Fluorophenyl)Pyrimidin-4-Yl]Piperidine-3-Carboxamide	6.819E-6	4.749E-5	1.3628	0.5026	-0.9924	down	LevelBi	-	397.2429	2.5469	M+H
Tigecycline	1.587E-5	9.335E-5	1.3606	0.1217	-3.0386	down	LevelBii	HMDB0014700	584.2702	2.7839	M-H
Thr-Ile	1.748E-5	0.0001008	1.3572	0.5952	-0.7485	down	LevelBii	HMDB0029064	231.1339	1.846	M-H
1,2-Cyclohexanedione	0.000163	0.000506	1.3556	0.3252	-1.6206	down	LevelBi	HMDB0031344	93.0339	4.574	M-H2O-H

	6	2					i				
Ala-Tyr-Leu	0.0006388	0.001632	1.3536	0.3292	-1.6028	down	LevelBi	-	366.2019	2.6567	M+H
Rhamnazin 3-Rutinoside	7.031E-8	1.414E-6	1.3572	1.8325	0.8738	up	LevelBii	HMDB0037467	637.1818	2.0631	M-H
Cosmosiin	0.0004146	0.001131	1.3611	0.4592	-1.123	down	LevelBi	HMDB0037340	433.109	4.1388	M+H
11-Hydroxyprogesterone 11-Glucuronide	0.0002737	0.0007865	1.3523	0.0646	-3.9515	down	LevelBii	HMDB0010364	487.2388	3.4048	M-H2O-H
Dopamine	1.905E-9	1.107E-7	1.3519	4.3879	2.1335	up	LevelBii	HMDB0000073	351.156	3.3671	2M+FA-H
N-Arachidonoyl Tryptophan	0.003996	0.008105	1.3434	0.5772	-0.7928	down	LevelBii	HMDB0242005	979.6367	9.2332	2M-H
Citbismine A	1.007E-7	1.864E-6	1.3498	1.6534	0.7255	up	LevelBii	HMDB0041086	661.1783	0.9324	M+Na-2H
N-Cyclooctyl-1-(4-Fluorobenzoyl)Piperidine-4-Carboxamide	2.339E-5	0.0001279	1.3499	0.5629	-0.8291	down	LevelBi	-	361.2309	5.5077	M+H
Val-Leu-Ser	5.926E-7	6.987E-6	1.3466	0.6238	-0.6809	down	LevelBi	-	318.1993	2.1311	M+H
Phe-Gln	1.179E-5	7.388E-5	1.3451	0.6192	-0.6915	down	LevelBi	HMDB0028993	294.1447	2.1112	M+H
1-Hydroxyvitamin D5	2.595E-5	0.0001389	1.3492	1.717	0.7798	up	LevelBii	HMDB0243905	451.3584	7.0019	M+Na
1-(2-Amino-3-Phenylpropanoyl)Proline	5.82E-5	0.0002075	1.343	0.7153	-0.4834	down	LevelBi	-	263.1386	2.4582	M+H
(E,E)-Futoamide	1.588E-8	4.762E-7	1.3452	0.5866	-0.7697	down	LevelBii	HMDB0032654	322.1397	2.2353	M+Na-2H
Phe-Val-Trp	0.002752	0.005838	1.3361	0.0913	-3.4531	down	LevelBi	-	451.2305	4.0401	M+H
Val-Pro-Gly	9.874E-7	1.033E-5	1.3447	0.6108	-0.7111	down	LevelBi	-	272.1617	2.1411	M+H
Nordihydrocapsaicin	0.001924	0.004259	1.3499	0.5533	-0.8539	down	LevelBi	HMDB0036328;LMFA08020319	294.2054	6.349	M+H

L-Lysine	2.58E-8	6.811E-7	1.3424	0.5819	-0.7811	down	LevelBi	HMDB0000182;PW_C000118	147.112	0.5797	M+H
Pa(Pgf2Alpha/12:0)	5.563E-5	0.001998	1.3461	1.8929	0.9206	up	LevelBii	HMDB0262777	711.3836	2.3777	M+Na-2H
Ile-Trp	3.953E-5	0.000173	1.3383	0.6316	-0.6628	down	LevelBi	HMDB0028918	318.1799	3.3405	M+H
1-Hexadecyl-1H-Indole-2,3-Dione	2.604E-7	3.77E-6	1.3395	0.759	-0.3978	down	LevelBi	-	372.2919	9.2841	M+H
Styrene	2.494E-7	3.655E-6	1.3403	0.7201	-0.4738	down	LevelBi	HMDB0034240	105.0693	2.1616	M+H
Isocolumbin	0.0005367	0.001409	1.3414	0.426	-1.231	down	LevelBii	HMDB0036837	339.1213	4.1855	M-H2O-H
N-(2,4-Dinitrophenyl)-5-Methoxytryptamine	1.102E-7	1.99E-6	1.3349	1.4545	0.5406	up	LevelBii	HMDB0254997	391.084	1.688	M+Cl
Val-Asp	0.001617	0.00367	1.3326	0.527	-0.9242	down	LevelBi	HMDB0029123	233.1126	1.0985	M+H
Pip(Pgf2Alpha/16:2(9Z,12Z))	0.001781	0.005446	1.3415	0.5608	-0.8344	down	LevelBii	HMDB0278654	1029.4502	6.896	M+FA-H
Asp-Phe	7.083E-6	4.902E-5	1.3374	1.9984	0.9989	up	LevelBi	HMDB0000706	281.1124	2.4294	M+H
Ziritaxestat	0.0005111	0.001354	1.3345	0.4943	-1.0166	down	LevelBii	HMDB0252755	630.2794	1.8534	M+ACN+H
Phe-Val-Asp	3.708E-6	2.914E-5	1.333	0.4097	-1.2872	down	LevelBi	-	380.1821	2.3004	M+H
Ala-Val-Phe	3.986E-6	3.078E-5	1.3308	0.5449	-0.876	down	LevelBi	-	336.1889	3.0635	M+H
3-Phenyllactic Acid	7.411E-8	1.464E-6	1.3326	0.7564	-0.4027	down	LevelBi	HMDB0000779	165.0553	3.7495	M-H
2-{[1-(2-Amino-Acetyl)-Pyrrolidine-2-Carbonyl]-Amino}-Propionic Acid	1.806E-5	0.0001035	1.3315	0.5552	-0.8489	down	LevelBi	-	244.1278	1.2943	M+H
20-Hydroxy-Leukotriene E4	3.541E-6	2.812E-5	1.3311	0.4458	-1.1655	down	LevelBii	HMDB0012639	454.227	3.8598	M-H
3-(3-Hydroxydecanoyloxy)Decanoic Acid	0.0001899	0.0005757	1.3328	0.6011	-0.7343	down	LevelBi	-	381.2588	6.729	M+Na
Cys-Phe	0.00132	0.003071	1.3234	0.4405	-1.1828	down	LevelBi	HMDB0028782	269.0939	2.4775	M+H

Vulgaxanthin I	3.408E-6	2.727E-5	1.329	2.271	1.1833	up	LevelBii	HMDB0029840	723.2118	2.3969	2M+FA-H
(1R*,2R*,4R*,8S*)-P-Menthane-1,2,8,9-Tetrol 9-Glucoside	5.782E-5	0.0002064	1.3262	0.3556	-1.4918	down	LevelBii	HMDB0039976	425.2018	2.3874	M+Hac-H
Ala-Tyr-Lys	1.291E-8	4.014E-7	1.3289	0.7156	-0.4828	down	LevelBi	-	381.2105	2.0614	M+H
Pyroglutamic Acid	0.0001814	0.0005534	1.3254	1.5606	0.6421	up	LevelBi	HMDB0000267;PW_C000182	130.0491	0.6692	M+H
Tyr-Asn-Glu	4.669E-7	5.887E-6	1.3288	0.6064	-0.7216	down	LevelBi	-	425.1641	2.0116	M+H
2',3',4'-Trihydroxyacetophenone	5.05E-6	3.731E-5	1.3314	0.5332	-0.9074	down	LevelBi	-	167.034	2.4348	M-H
Formyl-5-Hydroxykynurenamine	0.002291	0.004965	1.3277	3.6442	1.8656	up	LevelBii	HMDB0012948	267.0971	1.5948	M+Hac-H
(2R)-2-[[(2S)-2-Amino-3-Methylbutanoyl]Amino]-3-(4-Hydroxyphenyl)Propanoic Acid	0.0001594	0.000494	1.3226	0.6109	-0.711	down	LevelBi	-	281.1477	2.1616	M+H
(R)-2-Nitro-P-Mentha-1,5-Diene	1.538E-5	9.092E-5	1.3228	0.6166	-0.6976	down	LevelBii	HMDB0036090	199.1432	2.0017	M+NH4
Tryptoline	0.01163	0.02075	1.3283	0.4608	-1.1177	down	LevelBi	HMDB0012488	173.1065	2.8555	M+H
Ile-His	0.006444	0.01237	1.3281	0.4432	-1.174	down	LevelBi	HMDB0028909	269.1594	1.0591	M+H
Pyrano(2,3-C)Pyrazol-6(2H)-One, 3,4-Dimethyl-2-(2-Thienylmethyl)-	0.0003515	0.0009803	1.3248	0.5203	-0.9426	down	LevelBii	HMDB0253035	319.0753	1.156	M+Hac-H
(22E, 24X)-Ergosta-4,6,8,22-Tetraen-3-One	0.000128	0.0004094	1.3153	0.5584	-0.8405	down	LevelBii	HMDB0030898	413.2822	8.917	M+Na-2H
Pgp(Txb2/A-13:0)	0.0003135	0.0008853	1.3193	0.3783	-1.4024	down	LevelBii	HMDB0274305	855.4096	3.0922	M-H2O-H
Erythroascorbic Acid 1'-A-D-Glucoside	3.387E-5	0.0001725	1.3199	0.5649	-0.8239	down	LevelBii	HMDB0033627	353.0706	0.7365	M+FA-H
Methyl 3-(Benzylamino)-2-Methylpropanoate	2.097E-6	1.865E-5	1.3163	0.4729	-1.0803	down	LevelBi	-	262.1424	5.5272	M+CH3OH+Na
Trp-Ala	0.000974	0.002351	1.3112	0.4372	-1.1936	down	LevelBi	HMDB0029076	276.1328	2.3004	M+H

	8										
12-Epinapelline	5.864E-5	0.0002088	1.3141	0.4701	-1.0891	down	LevelBi	-	360.2545	8.1733	M+H
Achyranthoside D	9.041E-5	0.0003054	1.32	0.5808	-0.7838	down	LevelBii	HMDB0031023	1177.5225	7.5177	M+Hac-H
Fahfa(20:3/22:5)	7.339E-5	0.0002543	1.3146	0.6341	-0.6571	down	LevelBi	-	633.4836	8.88	M-H
18R-Hepe	3.569E-6	2.825E-5	1.313	0.5241	-0.932	down	LevelBii	HMDB0012611	319.2248	5.0586	M+H
Tyr-Ala	0.005698	0.01111	1.3049	0.3507	-1.5118	down	LevelBi	HMDB0029098	253.1169	1.9323	M+H
Farnesylcysteine	4.437E-8	1.008E-6	1.3134	0.5165	-0.9531	down	LevelBii	HMDB0011627	326.213	6.7878	M+H
Dialdehyde	5.124E-8	1.125E-6	1.3138	4.7748	2.2554	up	LevelBii	HMDB0251125	403.1463	4.4538	M+Cl
Traumatic Acid	4.439E-6	3.354E-5	1.3127	0.5398	-0.8894	down	LevelBii	HMDB0000933	227.1275	5.0198	M-H
Pa(5-Iso Pgf2Vi/18:3(6Z,9Z,12Z))	0.001945	0.004296	1.3173	0.4508	-1.1495	down	LevelBii	HMDB0264142	785.4291	7.8848	M+FA-H
2-Amino-4-Oxopentanoic Acid	1.064E-7	1.937E-6	1.3112	2.5141	1.33	up	LevelBii	HMDB0244991	152.034	2.6799	M+Na-2H
4-Hydroxycinnamyl Aldehyde	1.171E-7	2.064E-6	1.3102	0.7286	-0.4568	down	LevelBi	HMDB0040986	147.0446	3.7495	M-H
Lpc(O-14:0)	0.0004143	0.00113	1.3083	0.6254	-0.6771	down	LevelBi	LMGP01060007	498.3165	7.7115	M+FA-H
Phe-Phe	0.0003964	0.001089	1.3012	0.6368	-0.6511	down	LevelBi	HMDB0013302	313.1536	3.41	M+H
8-Hydroxy-Deoxyguanosine	5.069E-5	0.0001855	1.308	1.7751	0.8279	up	LevelBii	HMDB0003333	316.1276	2.7064	M+CH3OH+H
Trp-Met	0.001174	0.002772	1.2997	0.4967	-1.0096	down	LevelBi	HMDB0029089	336.1362	2.9844	M+H
Leu-Lys	0.00	0.00	1.3007	0.54	-0.873	dow	Lev	HMDB0028934	260.	1.0396	M+H

	1117	2651		6		n	elBi		1955		
Pipecolic Acid	3.878E-7	5.095E-6	1.3053	0.6109	-0.711	down	LevelBi	HMDB0000070;HMDB0000716;HMDB0000070	130.0854	0.5697	M+H
Casomorphin	5.563E-9	2.285E-7	1.3053	0.3019	-1.7277	down	LevelBii	HMDB0059787	828.4309	3.9615	M+H-2H2O
Temocaprilat	4.472E-7	5.684E-6	1.3021	1.7985	0.8468	up	LevelBii	HMDB0061721	429.0965	3.0733	M-H2O-H
Annomuricatin B	0.02469	0.03982	1.2859	0.1045	-3.2588	down	LevelBii	HMDB0303182	722.3621	4.0206	M+H-H2O
Ala-Val-Pro	5.437E-6	3.962E-5	1.3031	0.6339	-0.6576	down	LevelBi	-	286.1745	2.1616	M+H
(2R)-1-(2-Hydroxyethyl)-2,5,5,8A-Tetramethyldecahydronaphthalen-2-Ol	0.000005008	0.001332	1.3056	0.6562	-0.6078	down	LevelBi	-	277.2154	6.641	M+Na
Phosphoribosyl Formamidocarboxamide	5.818E-7	6.888E-6	1.3007	1.6577	0.7292	up	LevelBii	HMDB0001439	365.0474	1.9969	M-H
His-gamma-Glu	7.227E-7	8.109E-6	1.298	0.4659	-1.1019	down	LevelBii	HMDB0028899	264.1078	0.727	M-H2O-H
Ile-Ile-Asp	8.795E-6	5.864E-5	1.2959	0.6474	-0.6272	down	LevelBi	-	360.2105	2.2507	M+H
Byssochlamic Acid	0.001651	0.003733	1.2982	0.3623	-1.4646	down	LevelBii	HMDB0034262	353.1007	4.3708	M+Na-2H
Gly-Leu-Phe	5.406E-5	0.000195	1.2952	0.5881	-0.7659	down	LevelBi	-	336.1898	3.5573	M+H
Verdamicin	0.009738	0.01776	1.2987	0.3448	-1.5361	down	LevelBii	HMDB0259789	444.2786	2.6069	M+H-H2O
Trans-Zeatin	9.362E-5	0.0003143	1.2921	0.5948	-0.7496	down	LevelBi	HMDB0012204	274.1278	1.6006	M+CH3OH+Na
N-Arachidonoyl Gaba	3.33E-7	4.542E-6	1.294	0.7699	-0.3772	down	LevelBii	HMDB0062330	372.293	9.1381	M+H-H2O
Tyr-Gln	1.891E-7	2.982E-6	1.2932	0.6833	-0.5494	down	LevelBi	HMDB0029103	327.1655	1.9323	M+NH4
Rimcazole	0.0004641	0.001248	1.2953	2.04	1.0285	up	LevelBii	HMDB0257233	342.192	3.3483	M+Na-2H

(11E,15Z)-9,10,13-Trihydroxy-11,15-Octadecadienoic Acid	2.579E-5	0.0001384	1.2931	0.7129	-0.4882	down	LevelBi	-	327.2161	5.7291	M-H
Prostaglandin A2	0.003626	0.007444	1.2916	0.4534	-1.1412	down	LevelBi	HMDB0002752;PW_C001699	335.2202	5.3319	M+H
Tyr-Ala-Asp	1.403E-5	8.475E-5	1.286	0.4119	-1.2796	down	LevelBi	-	368.1439	2.0017	M+H
Vaccenyl Carnitine	0.006993	0.01331	1.2914	1.474	0.5597	up	LevelBii	HMDB0006351	470.3466	7.1364	M+FA-H, 2M+FA-H, M+Cl
Tetradecanoylcarnitine	0.005441	0.01066	1.2861	1.4193	0.5052	up	LevelBi	HMDB0005066;LMFA07070107	372.3096	6.5641	M+H
Glucosyl (E)-2,6-Dimethyl-2,5-Heptadienoate	5.733E-6	4.131E-5	1.2844	0.5115	-0.9673	down	LevelBii	HMDB0035136	361.1479	3.8507	M+FA-H
Rutin	2.38E-6	2.063E-5	1.2869	0.4844	-1.0457	down	LevelBii	HMDB0003249	631.1236	3.4236	M+Na-2H
Val-Ala-Leu	0.0003013	0.0008557	1.2811	0.4257	-1.232	down	LevelBi	-	302.2055	2.8754	M+H
(3S)-3-(Benzyloxy)-L-Aspartic Acid	5.405E-5	0.000195	1.2862	0.4349	-1.2012	down	LevelBii	HMDB0250058	238.0742	1.156	M-H
O,O-Didodecyl Phosphorodithioate	0.002155	0.0047	1.2834	0.5275	-0.9227	down	LevelBi	-	505.2725	2.8653	M+K
Leu-Pro	4.084E-8	9.567E-7	1.2816	0.7735	-0.3705	down	LevelBi	HMDB0011175	229.1543	2.1912	M+H
Leu-Pro-Val-Leu-Glu	0.001855	0.00413	1.2741	0.2267	-2.1409	down	LevelBi	-	568.3365	9.5504	M-H
Leu-Ile-Glu	0.0005032	0.001338	1.275	0.5354	-0.9012	down	LevelBi	-	374.2265	2.3901	M+H
Betavulgaroside Ix	8.451E-5	0.000288	1.2839	0.6061	-0.7225	down	LevelBii	HMDB0034869	1147.512	7.5737	M+Hac-H
4-(4-Methylcyclohexyl)-4-Oxobutanoic Acid	0.000413	0.001128	1.2762	0.2561	-1.9651	down	LevelBii	HMDB0253742	199.1325	5.7423	M+H
Tyrphostin B42	0.004659	0.00927	1.2802	0.2127	-2.2331	down	LevelBi	HMDB0244549	312.1325	4.8049	M+NH4
N(4)-Octadecyl-1-Arabinofuranosylcytosine	9.019E-5	0.0003048	1.2778	0.6602	-0.5991	down	LevelBii	HMDB0248147	494.3578	8.8892	M-H

Cdp-Dg(6 Keto-Pgf1Alpha/18:0)	0.002965	0.006242	1.2812	0.6283	-0.6704	down	LevelBii	HMDB0291000	1096.5449	6.8269	M+H
Docosapentaenoic Acid (22N-3)	0.0002145	0.0006392	1.2782	0.5342	-0.9047	down	LevelBii	HMDB0006528	363.2872	7.9394	M+CH3OH+H
Stevioside	2.019E-5	0.0001136	1.2765	0.5804	-0.785	down	LevelBii	HMDB0034945	849.3723	5.7568	M+FA-H
Zearalanone	0.0001397	0.000442	1.277	0.519	-0.9462	down	LevelBii	HMDB0259993	341.1366	4.0277	M+Na-2H
Lentialexin	0.0004237	0.001153	1.2746	0.371	-1.4305	down	LevelBii	HMDB0038997	121.0638	5.0781	M+H
5-Hydroxylysinonorleucine	3.58E-7	4.8E-6	1.2716	0.7055	-0.5033	down	LevelBii	HMDB0246805	346.1937	2.0911	M+CH3OH+Na
N-[[3-Hydroxy-2-(2-Pentenyl)Cyclopentyl]Acetyl]Isoleucine	0.0003955	0.001087	1.2729	0.2407	-2.0548	down	LevelBii	HMDB0041248	360.1927	2.979	M+Cl
Angiotensin Iv	4.224E-5	0.000173	1.2767	0.5274	-0.9231	down	LevelBii	HMDB0001038	819.3978	5.5264	M+FA-H
Lpe(19:0)	0.009932	0.01808	1.2779	2.45	1.2928	up	LevelBi	LMGP02050028	494.3244	8.9077	M-H
Avocadene 4-Acetate	0.005967	0.01157	1.2755	1.6891	0.7562	up	LevelBii	HMDB0031045	327.2514	7.1456	M-H
Ser-Val	3.252E-5	0.000167	1.2681	0.4665	-1.0999	down	LevelBii	HMDB0029052	203.1023	0.727	M-H
3,4-Diethoxybenzaldehyde	2.144E-5	0.0001195	1.2722	0.5354	-0.9012	down	LevelBi	-	217.0842	5.0781	M+Na
8-Chloroinosine	6.994E-5	0.0002435	1.2689	1.607	0.6843	up	LevelBii	HMDB0247428	347.0375	2.0157	M+FA-H
Biotin	1.842E-5	0.0001054	1.2686	1.8734	0.9057	up	LevelBii	HMDB0000030	533.1713	1.9969	2M+FA-H
Nigellicine	0.0001239	0.000399	1.2664	0.4968	-1.0093	down	LevelBii	HMDB0030269	247.1095	2.8159	M+H

3-(4-Methylphenyl)-2-Propenenitrile	5.517E-7	6.641E-6	1.2669	0.7975	-0.3264	down	LevelBi	-	144.0808	2.4099	M+H
Kaempferol 3-(3",6"-Diacetyl-2",4"-Di-P-Coumaroylrhamnoside)	0.0002937	0.0008367	1.2712	0.5255	-0.9284	down	LevelBii	HMDB0029503	805.1708	4.139	M-H2O-H, M+Cl
N6-Methyl-2'-Deoxyadenosine	0.0005169	0.001367	1.2615	0.0002	-12.0477	down	LevelBii	HMDB0255300	529.2276	3.1583	2M-H
Corymboside	1.512E-5	8.981E-5	1.2647	0.6424	-0.6385	down	LevelBi	HMDB0030803;LMPK12110260	565.1507	3.1925	M+H
Estradiol Enanthate	0.0002727	0.0007845	1.2642	0.6281	-0.671	down	LevelBii	HMDB0251964	419.2386	8.8332	M+Cl
6-O-(7-Methyloctanoyl)-Alpha-D-Glucopyranosyl Alpha-D-Glucopyranoside	0.02761	0.04388	1.2463	0.0915	-3.4494	down	LevelBi	-	483.2471	4.1978	M+H
10-Formyldihydrofolate	2.398E-8	6.462E-7	1.2618	0.5816	-0.7818	down	LevelBii	HMDB0006485	470.1423	2.5853	M-H
Phytosphingosine	0.01255	0.0222	1.2524	1.7631	0.8181	up	LevelBi	HMDB0004610;HMDB0004628;HMDB0004610;LMSP01030001	318.2988	6.7485	M+H
15-Stachen-19-Ol	0.00354	0.007295	1.265	0.6472	-0.6277	down	LevelBi	-	289.2511	7.5701	M+H
Ser-Leu-Lys	2.699E-7	3.857E-6	1.2566	0.5928	-0.7543	down	LevelBi	-	347.2272	1.2454	M+H
N5-Acetyl-N2-Gamma-L-Glutamyl-L-Ornithine	5.054E-5	0.0001851	1.2572	0.5011	-0.9968	down	LevelBii	HMDB0039423	324.1161	1.3241	M+Na-2H
Concanamycin	0.001047	0.002504	1.2584	2.7087	1.4376	up	LevelBii	HMDB0250429	751.4694	3.2148	M+Hac-H
Pisumionoside	0.0006547	0.001668	1.255	0.1814	-2.4631	down	LevelBii	HMDB0039947	449.2035	3.4518	M+FA-H
Serotonin	0.0004836	0.001292	1.2534	0.6566	-0.6069	down	LevelBi	HMDB0000259;PW_C000178	177.1013	2.1311	M+H
Prostaglandin A1	5.274E-5	0.0001912	1.26	0.4298	-1.2184	down	LevelBii	HMDB0002656	381.2259	5.5725	M+FA-H
Anandamide	0.003692	0.007567	1.2609	0.4551	-1.1358	down	LevelBi	HMDB0004080	348.2867	8.2027	M+H
Tuberoside F	0.005347	0.01049	1.2634	0.397	-1.3327	down	LevelBii	HMDB0302986	1077.5425	6.5081	M-H

Betaine	0.0002456	0.0007173	1.2505	0.5458	-0.8736	down	LevelBii	HMDB0000043	237.1831	7.4132	2M+H
2-Aminooctadec-4-Yne-1,3-Diol	2.291E-6	2.002E-5	1.2544	0.6774	-0.562	down	LevelBi	-	298.2728	6.447	M+H
Kaempferol 3-[Apiosyl-(1->2)-Galactoside]	0.01127	0.0202	1.2508	0.3039	-1.7184	down	LevelBii	HMDB0037430	579.1333	3.6657	M-H
N-Acetyl-L-Tyrosine	0.00072588	0.001822	1.251	0.556	-0.8469	down	LevelBii	HMDB0000866	188.0687	2.8754	M+H-2H2O
2-Hydroxy-4-(Methylthio)Butanoic Acid	4.122E-7	5.348E-6	1.2533	0.7081	-0.4979	down	LevelBi	LMFA01050480	149.0273	2.5194	M-H
Val-Phe-Asp	2.401E-5	0.0001309	1.2495	0.5897	-0.7618	down	LevelBi	-	380.1804	2.37	M+H
Lys-Val	9.065E-5	0.0003061	1.251	0.6347	-0.6558	down	LevelBi	HMDB0028964	246.1799	0.7178	M+H
Methyl 3B,24-Dihydroxy-11,13(18)-Oleanadien-30-Oate	0.01121	0.02011	1.2604	1.8974	0.924	up	LevelBii	HMDB0035257	502.3856	7.2361	M+NH4
13,14-Dihydro Pgf-1A	0.003328	0.006912	1.2568	0.5263	-0.926	down	LevelBii	HMDB0005076	357.2632	6.6837	M-H
Ile-Asp-Asp	3.65E-6	2.882E-5	1.2489	0.6642	-0.5902	down	LevelBi	-	362.1558	2.0216	M+H
Moexipril	1.624E-5	9.512E-5	1.2518	0.4822	-1.0522	down	LevelBii	HMDB0014829	995.4645	4.4631	2M-H
Abeado	5.138E-6	3.782E-5	1.2488	0.1621	-2.6248	down	LevelBii	HMDB0247746	370.1592	2.6894	M+Na-2H
Paramethasone Acetate	7.64E-6	5.225E-5	1.2459	0.3821	-1.3879	down	LevelBii	HMDB0256122	433.2046	3.0639	M-H
N-Myristoyl Methionine	0.00073388	0.001839	1.2413	0.5835	-0.7771	down	LevelBii	HMDB0242056	360.2579	8.3772	M+H
3-Hydroxy-5-(Hydroxymethyl)-2-Methylisonicotinaldehyde Oxime	2.526E-5	0.0001364	1.2467	1.4873	0.5727	up	LevelBi	-	183.0765	1.9721	M+H
Pro-Ile-Leu	0.002696	0.005733	1.2392	0.62	-0.6897	down	LevelBi	-	342.237	3.3308	M+H

1-Monolinolenin	0.007016	0.01335	1.2451	1.5808	0.6607	up	LevelBi	-	353.2671	8.028	M+H
Pip(5-Iso Pgf2Vi/20:4(5Z,8Z,11Z,14Z))	0.003473	0.00718	1.2502	0.5246	-0.9308	down	LevelBii	HMDB0280017	989.4475	5.9605	M-H2O-H
1-(5Z,8Z,11Z,14Z,17Z-Eicosapentaenoyl)-Glycero-3-Phosphate	0.007144	0.01357	1.2395	0.4349	-1.2014	down	LevelBii	HMDB0062308	491.1938	1.9687	M+Cl
Thr-Phe	0.001815	0.004056	1.2372	0.5152	-0.9569	down	LevelBii	HMDB0029068	265.1183	2.1297	M-H
Urolithin C 3-Glucuronide	2.717E-5	0.0001439	1.2422	0.3464	-1.5296	down	LevelBii	HMDB0142405	441.0449	0.5569	M+Na-2H
Ala-Phe	1.652E-6	1.547E-5	1.2416	0.6661	-0.5863	down	LevelBi	HMDB0028694	237.1217	2.1616	M+H
2''-O-Acetylisoorientin	4.251E-8	9.894E-7	1.2428	0.5808	-0.7839	down	LevelBii	HMDB0038816	525.0822	2.5287	M+Cl
Fasoracetam	0.0007515	0.001878	1.236	0.4656	-1.1027	down	LevelBii	HMDB0252174	241.1185	3.1018	M+FA-H
Ascofuranone	1.788E-6	1.646E-5	1.2417	0.6006	-0.7356	down	LevelBii	HMDB0248651	385.1586	4.0796	M+H-2H2O
(6E,9E)-Hexadeca-6,9-Dienedioylcarnitine	0.0001674	0.0005162	1.2477	1.5545	0.6364	up	LevelBii	HMDB0241489	430.2556	3.2715	M+Na-H2O
Amylopectin	2.811E-8	7.209E-7	1.2416	1.4359	0.5219	up	LevelBii	HMDB0003255	827.2663	1.1097	M-H
17-Hydroxypregnenolone Sulfate	3.264E-5	0.0001674	1.2361	0.3191	-1.6477	down	LevelBii	HMDB0000416	457.1861	2.5949	M+FA-H
Glu-Ile	0.0005379	0.001411	1.242	1.7749	0.8277	up	LevelBi	HMDB0028822	283.1253	2.2211	M+Na
Dihydroouabain	8.124E-6	5.493E-5	1.2361	2.2292	1.1566	up	LevelBii	HMDB0251322	641.3158	2.6369	M+CH3OH+Na
Tunicamycin A	0.0005249	0.001384	1.2434	0.5735	-0.8022	down	LevelBii	HMDB0259327	851.3886	5.5541	M+Na-2H
11-Cis-Retinol	0.01	0.02	1.2428	0.51	-0.959	dow	Lev	HMDB0006216;LMPR01090005	269.	9.4716	M+H-H2O

	625	781		41	9	n	elBi	;PW_C002665	2254		
Cucurbitacin S	0.0003519	0.0009812	1.2371	0.5678	-0.8165	down	LevelBii	HMDB0250583	537.2566	5.9078	M+K
Asp-Trp	0.001544	0.003529	1.2317	1.878	0.9092	up	LevelBi	HMDB0028764	320.1235	2.7762	M+H
1-Isothiocyanato-8-(Methylthio)Octane	7.219E-6	4.987E-5	1.235	0.544	-0.8782	down	LevelBii	HMDB0038447	216.0865	2.642	M-H
Palmitoyl-L-Carnitine	0.02729	0.04342	1.2387	2.1701	1.1178	up	LevelBi	-	398.3249	7.0533	M-H
Bisphenol S	0.004322	0.008688	1.2375	9.6406	3.2691	up	LevelBi	HMDB0240712	251.0361	4.2271	M+H
4-(4-Nitrobenzyl)Pyridine	3.834E-6	2.99E-5	1.2346	0.4194	-1.2537	down	LevelBii	HMDB0246294	259.0742	2.4535	M+FA-H
3-Hydroxydodecanedioic Acid	2.71E-6	2.273E-5	1.2317	0.2984	-1.7445	down	LevelBii	HMDB0000413	267.1206	4.528	M+Na-2H
7-Hydroxy-5-Methoxy-6,8-Dimethylflavanone	0.0002734	0.0007861	1.2273	0.4665	-1.1	down	LevelBii	HMDB0030695	333.0899	4.7243	M+Cl
Deacetoxy(7)-7-Oxokhivorinic Acid	1.682E-5	9.801E-5	1.2292	0.0113	-6.4688	down	LevelBii	HMDB0341151	541.2104	2.2735	M+Na-2H
Dethiobiotin	4.534E-5	0.000173	1.2321	0.6231	-0.6824	down	LevelBii	HMDB0003581	213.1235	1.912	M-H
Diethylphosphate	8.098E-7	8.807E-6	1.2273	0.5871	-0.7683	down	LevelBii	HMDB0012209	199.0363	1.1097	M+FA-H
3-(1H-Indol-3-Yl)-2-(Trimethylazaniumyl)Propanoate	0.0002413	0.0007064	1.2287	0.3399	-1.5567	down	LevelBi	-	247.1424	2.4872	M+H
Leu-Trp	4.911E-7	6.101E-6	1.2242	0.7185	-0.4769	down	LevelBi	HMDB0028940	318.1802	3.1925	M+H
Lisinopril, Epsilon-Biotinamidocaproyl-	5.078E-7	6.265E-6	1.2258	0.6111	-0.7106	down	LevelBii	HMDB0244524	777.4202	5.5174	M+CH3OH+H
Indole-3-Lactic Acid	9.61E-7	1.01E-5	1.2252	0.6755	-0.5659	down	LevelBi	HMDB0000671	206.0811	3.8823	M+H
Pe(O-20:5/18:0)	0.005242	0.0103	1.2277	1.9943	0.9959	up	LevelBi	-	810.5637	8.6411	M+Hac-H
Ile-Lys	0.00	0.00	1.2211	0.54	-0.869	dow	Lev	HMDB0028912	258.	1.0538	M-H

	0549	1437		73	5	n	elBii		1814		
5,6-Octadienoylglycine	2.269E-5	0.000125	1.2218	0.4653	-1.1037	down	LevelBii	HMDB0094798	215.1376	2.6665	M+NH4
3-Hydroxybutyrylcarnitine	7.164E-7	8.057E-6	1.2241	1.3638	0.4476	up	LevelBi	HMDB0013127;LMFA07070037	248.1485	1.5244	M+H
Hydroxyphenyllactic Acid	2.581E-7	3.758E-6	1.2219	0.7365	-0.4413	down	LevelBi	HMDB0000755	181.0503	2.5476	M-H
Mesterolone	0.0008784	0.0002149	1.2251	0.5093	-0.9734	down	LevelBi	HMDB0006036;LMST02020107	269.225	5.361	M+H-2H2O
Octadec-9-Enedioic Acid	0.0008756	0.0002144	1.2253	0.6938	-0.5274	down	LevelBi	-	313.2361	6.641	M+H
Crenolanib	2.632E-5	0.0001403	1.2173	0.5912	-0.7584	down	LevelBii	HMDB0250534	442.2288	2.3777	M-H
Histidylleucine	7.433E-8	1.464E-6	1.2186	0.6288	-0.6694	down	LevelBii	HMDB0028889	327.1653	2.0725	M+Hac-H
Ps(Monome(11,3)/Monome(13,5))	0.02933	0.04624	1.2212	2.1904	1.1312	up	LevelBii	HMDB0061604	894.5472	7.978	M-H
Hexanoyl-L-Carnitine	2.173E-6	1.914E-5	1.2207	1.3659	0.4499	up	LevelBi	-	260.1853	3.2517	M+H
Apigenin 6,8-Di-Glucopyranoside	8.342E-6	5.611E-5	1.2188	0.6394	-0.6451	down	LevelBi	-	595.1604	3.0238	M+H
Ile-Ser	0.0012944	0.0041344	1.2136	0.68	-0.5564	down	LevelBi	HMDB0028916	219.1328	1.5339	M+H
2-Benzylsulfanyl-N-[[1-(Hydroxymethyl)Cyclopropyl]Methyl]Acetamide	6.502E-8	1.336E-6	1.2163	0.6233	-0.682	down	LevelBi	-	266.1212	0.7273	M+H
1-Methyl-L-Histidine	2.114E-6	1.875E-5	1.2145	1.3772	0.4617	up	LevelBi	HMDB0000001	170.0915	0.679	M+H
N-[2-(1,3-Benzodioxol-5-Yloxy)Ethyl]Cyclopentanecarboxamide	5.729E-7	6.805E-6	1.2167	0.6261	-0.6755	down	LevelBi	-	278.1376	2.3004	M+H
Gly-Pro-Phe	1.601E-6	1.515E-5	1.2145	0.6642	-0.5903	down	LevelBi	-	320.1586	2.7562	M+H
1,2,10-Trihydroxydihydro-Trans-Linalyl Oxide 7-O-Beta-D-Glucopyranoside	6.148E-6	4.367E-5	1.2137	0.6962	-0.5224	down	LevelBii	HMDB0033237	415.218	2.1512	M+CH3OH+H
Ile-Ile-Glu	0.000169	0.000522	1.2151	0.5215	-0.9393	down	LevelBi	-	374.2252	2.517	M+H

	7	3									
13-Hydroxy-5'-O-Methylmelledonal	0.005188	0.01021	1.2069	0.1424	-2.8121	down	LevelBii	HMDB0035864	443.1675	2.2353	M-H2O-H
4-Hydroxycoumarin	2.612E-6	2.217E-5	1.2157	0.0933	-3.4227	down	LevelBi	-	161.0235	3.6936	M-H
4,17-Dimethyltrilostane	0.0018311	0.0055799	1.2124	0.4974	-1.0074	down	LevelBii	HMDB0242688	378.2021	3.4613	M+Na-2H
Gliadins	9.534E-5	0.00031889	1.2101	0.3642	-1.4574	down	LevelBii	HMDB0252744	652.2694	2.6515	M+Na-2H
Plazomicin	0.007467	0.01411	1.2081	0.4491	-1.1548	down	LevelBii	HMDB0256636	615.3316	3.4686	M+Na
Ser-Ile-Leu	6.114E-5	0.00021644	1.2116	0.6193	-0.6913	down	LevelBi	-	332.2164	2.7762	M+H
1-Hexanol Arabinosylglucoside	0.004593	0.009162	1.2127	0.5959	-0.7469	down	LevelBii	HMDB0031689	414.2333	2.5069	M+NH4
Nebularine	3.741E-7	4.96E-6	1.2106	1.3068	0.3861	up	LevelBii	HMDB0029956	251.0765	1.688	M-H
Glu-Phe	0.005023	0.009914	1.2196	1.8506	0.888	up	LevelBi	HMDB0029156	295.1274	2.4	M+H
Elvitegravir	5.084E-6	3.75E-5	1.2101	1.4993	0.5843	up	LevelBii	HMDB0251760	492.1189	2.0063	M+FA-H
1-(4-Methoxyphenyl)Cyclopropanecarboxylic Acid	5.868E-5	0.00020888	1.2115	0.6438	-0.6352	down	LevelBi	-	175.0745	4.0206	M+H-H2O
3-O-Beta-D-Galactopyranosyl-L-Arabinose	0.03253	0.05048	1.2218	0.1817	-2.4606	down	LevelBii	HMDB0038854	347.0747	4.6386	M+Cl
2-Feruloyl-1-Sinapoylgentiobiose	1.469E-7	2.452E-6	1.2082	1.3545	0.4377	up	LevelBii	HMDB0030334	705.2048	0.9324	M-H2O-H
Ser-Tyr	0.02707	0.0431	1.2153	2.3536	1.2349	up	LevelBi	HMDB0258243	269.1109	2.0414	M+H
Cdp-Dg(Pgj2/I-22:0)	0.02572	0.04126	1.2161	0.5508	-0.8604	down	LevelBii	HMDB0294330	1133.6129	6.2901	M+NH4
(9Z)-5,8,11-Trihydroxyoctadec-9-Enoic Acid	0.00	0.00	1.2107	0.60	-0.714	dow	Lev	-	331.	5.6542	M+H

	06734	1708		93	8	n	elBi		246		
Asparaginylglycyl-L-Arginine	2.488E-6	2.131E-5	1.2042	0.6875	-0.5405	down	LevelBii	HMDB0253872	326.157	3.5833	M-H2O-H
Isonuatigenin 3-[Rhamnosyl-(1->2)-Glucoside]	0.01141	0.0204	1.1931	0.3501	-1.5144	down	LevelBii	HMDB0030132	721.4154	5.0586	M+H-H2O
Asp-Tyr	6.132E-7	7.148E-6	1.2064	2	1	up	LevelBi	HMDB0028765	297.1072	2.1311	M+H
Dimethisterone	5.15E-5	0.0018 78	1.2033	0.7716	-0.3742	down	LevelBii	HMDB0251378	358.2762	8.3772	M+NH4
1-O-Hexadecyl-2-O-Ethyl-Sn-Glycero-3-Phosphocholine	0.0005744	0.001491	1.204	0.8032	-0.3161	down	LevelBi	-	510.3887	8.8826	M+H
Tryptamine	8.269E-6	5.578E-5	1.2021	0.7708	-0.3756	down	LevelBi	HMDB0000303;PW_C000207	161.1067	2.4197	M+H
Butaclamol	0.000992	0.002387	1.1984	0.2946	-1.7631	down	LevelBii	HMDB0242247	396.2102	3.1301	M+Cl
Val-Val-Phe	0.001724	0.00388	1.1956	0.6436	-0.6357	down	LevelBi	-	364.2213	3.321	M+H
St(24:1_O4_T/19:2)	0.004811	0.009541	1.1975	2.7433	1.4559	up	LevelBi	-	834.5484	9.0846	M+Hac-H
Oleyl Alcohol	0.0008532	0.002098	1.201	0.4203	-1.2506	down	LevelBii	HMDB0029632	286.3099	7.0892	M+NH4
Lpc(O-15:0)	0.0001342	0.0004263	1.2006	0.7454	-0.4239	down	LevelBi	LMGP01060009	512.3329	8.0341	M+FA-H
Avenestergenin B2	3.713E-5	0.000173	1.2001	0.47	-1.0892	down	LevelBii	HMDB0035266	627.3422	9.2703	M+Cl
Libenzapril	6.897E-5	0.0002405	1.1957	0.5523	-0.8564	down	LevelBii	HMDB0254077	384.1518	3.3388	M+Na-2H
Glyceryl Monolinoleate	0.01208	0.02147	1.2014	1.4655	0.5514	up	LevelBi	HMDB0242115	372.3092	8.4253	M+NH4
Quercetin	0.01134	0.02029	1.2026	0.4019	-1.3153	down	LevelBi	HMDB0005794;LMPK12110004	303.0483	4.1683	M+H
Pyridoxal	6.889E-7	7.84E-6	1.1987	1.4684	0.5542	up	LevelBi	HMDB0001545;PW_C001188	150.0544	2.0414	M+H-H2O
N6-Galacturonyl-L-Lysine	0.00	0.00	1.1952	0.29	-1.770	dow	Lev	HMDB0033106	343.	1.3148	M+Na-2H

	02563	07435		31	7	n	elBii		1105		
Patuletin 7-Glucoside	0.0003263	0.0009173	1.2005	0.5379	-0.8946	down	LevelBii	HMDB0303788	515.0775	4.1669	M+Na-2H
Gly-Thr-Ile	3.809E-6	2.979E-5	1.1922	0.6009	-0.7349	down	LevelBi	-	272.1613	1.9424	M+H-H2O
N-Formyl-L-Methionine	5.094E-5	0.0001863	1.1917	0.6535	-0.6138	down	LevelBii	HMDB0001015	176.0376	2.4535	M-H
Val-Asn-Pro	8.549E-8	1.628E-6	1.1926	0.7328	-0.4485	down	LevelBi	-	329.1807	2.0712	M+H
1-Methoxy-2-Propanol	1.122E-5	7.123E-5	1.1943	0.7018	-0.5108	down	LevelBi	HMDB0243915	55.0542	0.737	M+H-2H2O
Tyr-Phe	0.002492	0.005342	1.1874	0.6376	-0.6492	down	LevelBi	HMDB0029112	329.1482	2.8653	M+H
Amastatin	0.001703	0.003839	1.1948	0.3026	-1.7245	down	LevelBii	HMDB0248277	455.255	9.2981	M-H2O-H
(2E,4E)-Octadeca-2,4-Dienoylcarnitine	0.001056	0.002525	1.1979	1.8391	0.879	up	LevelBii	HMDB0241541	468.33	7.0347	M+FA-H
Cdp-Dg(Pgf1Alpha/20:1(11Z))	0.0006945	0.001756	1.1959	0.6451	-0.6325	down	LevelBii	HMDB0291796	1090.5679	5.0586	M+H-H2O
Cyclo(Pro-Phe-Trp-Lys-Thr-Phe)	0.02802	0.04445	1.1807	0.4685	-1.0938	down	LevelBii	HMDB0250003	787.3943	2.1593	M-H2O-H
16,17-Dihydro-16Alpha,17-Dihydroxygibberellin A4 17-Glucoside	0.004351	0.008738	1.199	3.3483	1.7434	up	LevelBii	HMDB0040647	527.2094	1.9876	M-H
Asp-Ala-Lys	2.197E-5	0.0001219	1.1891	0.6603	-0.5988	down	LevelBi	-	333.1751	2.2607	M+H
Oleoyl-L-Carnitine	0.01146	0.02049	1.1934	1.3035	0.3824	up	LevelBi	-	426.3565	7.1381	M+H
3-Carboxy-4-Methyl-5-Pentyl-2-Furanpropanoic Acid	0.007382	0.01397	1.1951	2.2319	1.1583	up	LevelBii	HMDB0061643	289.1044	5.9051	M+Na-2H
Mimosine	0.001507	0.003455	1.1924	1.8497	0.8873	up	LevelBii	HMDB0015188	243.0609	1.8648	M+FA-H
Acetyl-Dl-Valine	4.533E-6	3.412E-5	1.1877	0.62	-0.6896	down	LevelBi	-	158.0812	2.6136	M-H

(9Z,11E)-Octadeca-9,11-Dienoylcarnitine	0.0004692	0.001258	1.1909	1.4061	0.4917	up	LevelBii	HMDB0241539	468.3306	6.85	M+FA-H
Jasmonic Acid	7.05E-6	4.882E-5	1.1873	0.6144	-0.7028	down	LevelBi	HMDB0032797;LMFA02020001	211.1316	5.0194	M+H
Ile-Thr-Ile	7.602E-6	5.204E-5	1.1827	0.6249	-0.6784	down	LevelBi	-	346.2319	3.4196	M+H
6-Oxopiperidine-2-Carboxylic Acid	1.83E-6	1.675E-5	1.1848	0.6455	-0.6314	down	LevelBii	HMDB0061705	142.0499	2.576	M-H
Cyasterone	0.001997	0.004398	1.1767	0.5979	-0.742	down	LevelBi	HMDB0250618;LMST01040153	521.3108	2.7064	M+H
Coagulin R 3-Glucoside	1.811E-5	0.0001038	1.1792	0.4715	-1.0847	down	LevelBii	HMDB0033207	691.3292	2.1105	M+Hac-H
N-(1-Deoxy-1-Fructosyl)Histidine	0.004768	0.009468	1.1918	2.2091	1.1434	up	LevelBii	HMDB0037839	359.1556	4.4527	M+ACN+H
Glu-Tyr	3.366E-5	0.0001717	1.1806	1.6259	0.7012	up	LevelBii	HMDB0028831	309.1104	2.1202	M-H
Anapear	0.0008552	0.002101	1.1818	0.5575	-0.843	down	LevelBii	HMDB0040285	155.1059	6.0938	M+H
Glycochenodeoxycholic Acid 3-Glucuronide	6.796E-8	1.382E-6	1.1789	1.8884	0.9172	up	LevelBii	HMDB0002579	624.3341	2.6988	M-H
Dehydroascorbic Acid	0.0002323	0.0006839	1.1754	0.6212	-0.6868	down	LevelBii	HMDB0001264	173.0075	0.7458	M-H
Cholylisoleucine	0.0003712	0.001029	1.1818	0.6083	-0.7171	down	LevelBii	HMDB0242373	544.3567	6.4175	M+Na
Phe-Trp	0.0006567	0.001672	1.1716	0.5951	-0.7489	down	LevelBi	HMDB0029006	352.1638	3.5177	M+H
Dehydroeburicoic Acid	0.001684	0.003801	1.1727	0.6804	-0.5555	down	LevelBii	HMDB0250950	503.3284	9.392	M+Cl
Maltopentaose	2.043E-8	5.735E-7	1.1763	1.3262	0.4073	up	LevelBi	HMDB0012254	829.2758	1.1086	M+H
Leu-Met	6.666E-5	0.0002333	1.1725	0.7087	-0.4967	down	LevelBi	HMDB0028935	263.1412	2.2806	M+H

Isorhamnetin	0.00351	0.007243	1.1777	0.4697	-1.0901	down	LevelBi	HMDB0002655;LMPK12110002	315.0495	5.1949	M-H
(2S)-3-Hydroxy-1-[(2S)-Pyrrolidine-2-Carbonyl]Pyrrolidine-2-Carboxylic Acid	5.253E-6	3.859E-5	1.1729	0.7093	-0.4956	down	LevelBii	HMDB0257791	273.1084	2.2735	M+FA-H
Levonorgestrel	0.007865	0.01475	1.1757	0.6627	-0.5935	down	LevelBii	HMDB0014511	330.2444	8.4545	M+NH4
Physangulide	0.0009958	0.002396	1.1753	2.097	1.0684	up	LevelBii	HMDB0037381	543.2534	6.4896	M+Na-2H
12-O-Retinoylphorbol-13-Acetate	0.000351	0.0009791	1.175	0.6545	-0.6115	down	LevelBii	HMDB0249729	743.4157	5.7913	M+CH3OH+Na
Trp-Val-Asn	0.01309	0.02301	1.1655	0.5523	-0.8565	down	LevelBi	-	418.2073	2.3104	M+H
Gly-Pro-Glu	9.726E-7	1.019E-5	1.1708	0.624	-0.6804	down	LevelBii	HMDB0252832	360.1383	2.0252	M+Hac-H
Alpha-Calacorene	0.002929	0.006175	1.1759	0.5566	-0.8452	down	LevelBi	HMDB0036453	201.1629	6.447	M+H
Trp-Gln	5.043E-5	0.0001849	1.1703	0.3937	-1.3447	down	LevelBii	HMDB0029081	331.1394	2.416	M-H
Daidzein 7-O-Beta-D-Glucoside 4''-O-Methylate	1.072E-8	3.564E-7	1.1702	1.4424	0.5284	up	LevelBi	-	453.1195	2.0116	M+Na
2-(5,8-Tetradecadienyl)Cyclobutanone	0.002355	0.005082	1.172	0.7365	-0.4412	down	LevelBii	HMDB0037519	263.2354	8.7842	M+H
His-Asp-Leu	0.0003406	0.0009541	1.165	0.5133	-0.9621	down	LevelBi	-	384.1859	1.0788	M+H
Car(5:0)	8.621E-5	0.0002929	1.1721	1.3737	0.4581	up	LevelBi	-	246.1696	2.4775	M+
Tetrahydroharman-3-Carboxylic Acid	9.624E-7	1.011E-5	1.1678	0.7517	-0.4118	down	LevelBi	-	231.1123	2.9844	M+H
N-Cyclohexyl-9H-Purin-6-Amine	1.981E-7	3.097E-6	1.1676	1.4166	0.5024	up	LevelBi	-	218.1379	1.9523	M+H
Phe-Val-Leu	0.0005975	0.001544	1.1609	0.5426	-0.882	down	LevelBi	-	378.2366	3.9713	M+H
Leu-Tyr-Glu	0.009847	0.01795	1.1671	0.2549	-1.9718	down	LevelBi	-	424.206	2.2507	M+H

Gln-Phe	0.001823	0.004072	1.1578	0.6053	-0.7242	down	LevelBii	HMDB0028804	292.1297	2.1105	M-H
(3B,20R,22R)-3,20,27-Trihydroxy-1-Oxowitha-5,24-Dienolide 3-Glucoside	0.001008	0.002422	1.1597	0.4008	-1.319	down	LevelBii	HMDB0033573	676.3647	4.5896	M+ACN+H
1-[(3-Phenyl-3-Cyclohexenyl)Methyl]-4-Phenyl-1,2,3,6-Tetrahydropyridine	0.001863	0.004143	1.1532	0.2538	-1.978	down	LevelBii	HMDB0260239	374.2096	3.6657	M+FA-H
25-Cinnamoyl-Vulgaroside	0.02087	0.03449	1.1672	2.9708	1.5708	up	LevelBii	HMDB0041367	625.3415	8.2258	M+Hac-H
Pro-Glu	6.289E-7	7.278E-6	1.1563	0.6627	-0.5935	down	LevelBi	HMDB0029016	245.112	1.02	M+H
12-Oxo Phytodienoic Acid	0.0001746	0.000535	1.16	0.6827	-0.5507	down	LevelBi	-	293.2096	5.5174	M+H
Fahfa(18:2/3:0)	0.0007802	0.00194	1.158	0.753	-0.4093	down	LevelBi	-	351.2523	8.8332	M-H
Quadranoside Iii	0.01991	0.03309	1.1411	0.1765	-2.5025	down	LevelBi	-	705.3549	3.5968	M+K
Val-Phe-Ser	2.743E-5	0.000145	1.1532	0.614	-0.7037	down	LevelBi	-	393.211	3.6265	M+ACN+H
Alpha-Curcumene	0.004378	0.008782	1.1596	0.5222	-0.9374	down	LevelBi	HMDB0061838;HMDB0059878; LMPR0103060015	203.1781	6.447	M+H
4,6-O-Ethylidene-D-Glucose	5.629E-7	6.714E-6	1.152	1.2858	0.3627	up	LevelBii	HMDB0246615	251.0762	0.7644	M+FA-H
Val-Gly	8.298E-5	0.0002833	1.1499	0.6378	-0.6487	down	LevelBi	HMDB0259740	197.0886	1.2846	M+Na
Troxerutin	1.331E-7	2.281E-6	1.1527	1.4317	0.5178	up	LevelBii	HMDB0006083	801.2523	1.0911	M+Hac-H
Ile-Gln-Ile	0.009568	0.01748	1.1436	0.5613	-0.8332	down	LevelBi	-	373.2426	3.3505	M+H
Gln-Arg-Thr	0.00079	0.001963	1.1515	0.6692	-0.5795	down	LevelBi	-	445.2503	6.035	M+ACN+H
Caffeic Acid	8.926E-8	1.689E-6	1.1484	0.5825	-0.7797	down	LevelBii	HMDB0001964	179.0336	3.2431	M-H
1,4-Dihydro-3(2H)-Isoquinolinone	9.519E-5	0.000318	1.147	1.8557	0.892	up	LevelBi	-	130.0642	2.4	M+H-H2O

		5									
Fumonisin A2	0.003406	0.007056	1.1437	0.4577	-1.1276	down	LevelBii	HMDB0034699	712.3915	3.6363	M+H-2H2O
1-Aminocyclopropanecarboxylic Acid	2.228E-6	1.955E-5	1.1418	0.6913	-0.5327	down	LevelBi	HMDB0036458	102.0541	1.148	M+H
6-Hydroxy-2-Aminopurine	0.00010566	0.00034774	1.1397	1.3589	0.4425	up	LevelBi	HMDB0000132	152.0558	1.9821	M+H
Prenyl Glucoside	3.475E-6	2.771E-5	1.1375	1.3509	0.4339	up	LevelBii	HMDB0031876	293.1228	3.0733	M+FA-H
Ile-Pro	1.657E-7	2.695E-6	1.1389	0.7734	-0.3707	down	LevelBii	HMDB0011174	227.139	2.207	M-H
Xylose	3.256E-7	4.469E-6	1.139	0.6101	-0.7128	down	LevelBii	HMDB0000098	149.0444	0.7365	M-H
Lnape(2:0/N-20:0)	0.00067774	0.001715	1.1387	0.7702	-0.3767	down	LevelBi	-	550.3488	8.0059	M-H
Trans-Delta-Viniferin 3"-Glucoside	4.78E-5	0.00017773	1.1395	0.3753	-1.4138	down	LevelBii	HMDB0036366	597.1789	3.7123	M-H2O-H
Kinetensin 1-3	0.00029949	0.00084	1.1404	0.5282	-0.9208	down	LevelBii	HMDB0012983	379.2081	6.0528	M+Na-2H
Pe(O-8:0/10:0)	0.01551	0.02671	1.1481	1.4294	0.5154	up	LevelBi	-	961.6266	8.669	2M-H
N-Acetyl-L-Phenylalanine	1.044E-5	6.723E-5	1.1312	0.7216	-0.4706	down	LevelBii	HMDB0000512	206.0807	3.7311	M-H
Mavatrep	0.00065166	0.001661	1.1309	0.4883	-1.0342	down	LevelBii	HMDB0254350	443.1384	2.6988	M+Na-2H
Sacubitrilat	8.303E-6	5.593E-5	1.1308	1.5495	0.6318	up	LevelBii	HMDB0257440	404.1514	1.688	M+Na-2H
Solasodine Base + 2H + H2O, O-Hex-Hex-Hex-Pen	0.001192	0.002808	1.136	0.7257	-0.4626	down	LevelBi	-	1034.5437	5.029	M+H-H2O
Verbasoside	5.693E-5	0.00020389	1.1303	0.62	-0.6896	down	LevelBii	HMDB0039233	463.1855	3.6068	M+H

Pilocarpine	2.372E-7	3.505E-6	1.1305	0.6663	-0.5858	down	LevelBi	HMDB0015217	226.1547	1.9124	M+NH4
Sweroside	0.001293	0.003015	1.1325	0.1508	-2.7295	down	LevelBii	HMDB0258643	417.1418	2.6326	M+Hac-H
(E,E)-1,7-Diphenyl-4,6-Heptadien-3-Ol	0.0001199	0.0003884	1.1297	0.5475	-0.8691	down	LevelBii	HMDB0040893	299.1215	1.24	M+Cl
N-Docosahexaenoyl Phenylalanine	0.01089	0.01959	1.1238	0.4731	-1.0797	down	LevelBii	HMDB0062333	440.2949	2.557	M+H-2H2O
Val-Val	2.612E-5	0.0001395	1.1269	0.7647	-0.387	down	LevelBi	HMDB0029140	217.1538	2.1012	M+H
Scabioside C	0.01642	0.02808	1.1153	0.4154	-1.2675	down	LevelBi	-	789.443	4.1388	M+Na
Physagulin G	0.0002585	0.0007491	1.125	1.5412	0.6241	up	LevelBii	HMDB0039694	703.3015	2.1593	M-H2O-H
Tricoumaroyl Spermidine	0.001988	0.00438	1.1267	0.5554	-0.8484	down	LevelBii	HMDB0304518	628.2671	1.8555	M+FA-H
Platelet-Activating Factor	0.02186	0.0359	1.1306	0.4618	-1.1146	down	LevelBii	HMDB0062195	568.36	7.044	M+FA-H
Pe(O-8:0/8:0)	0.0001438	0.0004532	1.1251	1.4823	0.5678	up	LevelBi	-	905.5692	8.1614	2M-H
Cyclo(Trp-Asp-Pro-Ile-Leu)	0.01271	0.02241	1.137	1.847	0.8852	up	LevelBii	HMDB0250629	642.3557	4.3943	M+NH4
Dlcl(18:2/22:7)	0.003685	0.007555	1.1179	0.4815	-1.0545	down	LevelBi	-	991.4699	3.1865	M+Na-2H
S-(2-Methylpropionyl)-Dihydrolipoamide-E	1.289E-6	1.278E-5	1.1216	0.6193	-0.6912	down	LevelBii	HMDB0006868	276.1074	0.7365	M-H
N-Arachidonoyl Proline	0.02844	0.04508	1.1272	0.1462	-2.7742	down	LevelBii	HMDB0242002	436.2654	7.3219	M+Cl
Ceanothine B	0.02283	0.03722	1.1247	2.3598	1.2386	up	LevelBii	HMDB0029339	568.2915	4.2569	M+ACN+Na
Celosianin	0.002185	0.00476	1.126	0.5053	-0.9849	down	LevelBi	HMDB0302989	905.1695	4.1484	M+Cl

							i				
L-Alanine	8.911E-7	9.493E-6	1.1204	1.2596	0.333	up	LevelBi	HMDB0000161;PW_C000105	90.0543	0.7178	M+H
Isolinariinn D	1.866E-9	1.094E-7	1.1202	1.2737	0.349	up	LevelBi	-	707.2172	0.708	M+H
Porphobilinogen	2.116E-6	1.875E-5	1.1197	0.7236	-0.4667	down	LevelBii	HMDB0000245	225.0871	2.1688	M-H
5-[2-[(E)-4-Carboxybut-1-Enyl]-6-Hydroxy-4-Methoxybenzoyl]Oxyhexanoic Acid	2.316E-9	1.243E-7	1.1195	0.6254	-0.6771	down	LevelBi	-	381.1549	4.5992	M+H
Fahfa(18:1/3:0)	0.001075	0.002563	1.121	0.7758	-0.3662	down	LevelBi	-	353.2678	9.1118	M-H
Azacitidine	0.0005305	0.001397	1.1212	1.5646	0.6458	up	LevelBii	HMDB0015063	225.0619	1.3521	M-H2O-H
Leu-Phe-Asp	0.003476	0.007184	1.1131	0.4431	-1.1744	down	LevelBi	-	394.1968	2.905	M+H
Sulfolithocholylglycine	0.01472	0.02553	1.1128	0.2506	-1.9968	down	LevelBii	HMDB0002639	512.2671	2.2926	M-H
3-(4-Hydroxy-3-Methoxyphenyl)Propionic Acid	2.669E-6	2.252E-5	1.1165	0.7116	-0.4909	down	LevelBi	-	195.0652	3.5365	M-H
2-Hydroxyacetophenone	1.789E-6	1.646E-5	1.117	0.7078	-0.4987	down	LevelBii	HMDB0245150	135.0443	2.5476	M-H
Oxiracetam	0.0001146	0.0003731	1.1113	2.047	1.0335	up	LevelBii	HMDB0255986	123.0538	1.3528	M+H-2H2O
Kaempferol	0.01714	0.02913	1.1198	0.5708	-0.809	down	LevelBi	HMDB0005801;HMDB0032008;LMPK12110003	287.053	4.9024	M+H
P-Tolyl Sulfate	0.0002375	0.0006966	1.113	1.8547	0.8912	up	LevelBi	-	187.006	3.6936	M-H
Deoxynivalenol 3-Glucoside	0.0007662	0.001909	1.1179	1.7183	0.781	up	LevelBii	HMDB0039852	476.2132	3.0437	M+NH4
Gentamicin C	0.03314	0.05129	1.1117	0.0752	-3.7331	down	LevelBii	HMDB0252688	470.2597	2.5665	M+Na-2H
Cholylasparagine	0.000469	0.001258	1.1048	0.7369	-0.4404	down	LevelBii	HMDB0242378	557.3004	9.2238	M+Cl
Butyrylcarnitine	2.338E-5	0.000127	1.1123	1.2416	0.3122	up	LevelBi	HMDB0002013;LMFA07070054	232.1542	2.1616	M+H

		9									
Arg-Pro	0.03871	0.05857	1.0979	0.2888	-1.7917	down	LevelBi	HMDB0028717	272.1705	1.1086	M+H
Tryptophol	0.005582	0.01091	1.1129	1.8216	0.8652	up	LevelBii	HMDB0003447	142.0654	2.3969	M-H2O-H
9-Octadecynoic Acid	0.01214	0.02156	1.1151	0.5529	-0.8548	down	LevelBi	LMFA01030455	281.2466	7.1087	M+H
Tyr-Gln-Ile	8.454E-5	0.000288	1.1052	0.6818	-0.5527	down	LevelBi	-	423.2209	3.0339	M+H
Ergosine	0.01674	0.02854	1.0992	0.2371	-2.0767	down	LevelBii	HMDB0251913	582.2518	3.4425	M+Cl
Ile-Asn-Ile	6.005E-6	4.292E-5	1.1099	1.2829	0.3595	up	LevelBi	-	359.2275	3.3012	M+H
Inuline	0.001302	0.003034	1.1004	0.4523	-1.1446	down	LevelBii	HMDB0248466	585.3231	4.3337	M-H
Lpe(17:1)	1.535E-5	9.08E-5	1.1079	1.4086	0.4942	up	LevelBi	LMGP02050008	464.2761	7.9126	M-H
Lobucavir	0.003778	0.007712	1.1011	0.2919	-1.7764	down	LevelBii	HMDB0254142	310.1182	4.7981	M+FA-H
Ile-Glu-Asp	4.059E-7	5.284E-6	1.1053	0.7664	-0.3838	down	LevelBi	-	376.1735	2.0812	M+H
Uridine	0.0001577	0.0004903	1.1053	1.7646	0.8193	up	LevelBi	HMDB0000296;PW_C000202	243.0603	1.1746	M-H
12-Oxo-20-Trihydroxy-Leukotriene B4	4.953E-6	3.672E-5	1.1027	0.5273	-0.9232	down	LevelBii	HMDB0012553	403.1702	2.3495	M+Na-2H
Ser-Leu-Asp	1.396E-7	2.347E-6	1.1027	0.751	-0.4131	down	LevelBi	-	334.1608	2.0712	M+H
Riboflavin	0.0001296	0.0004137	1.1012	0.7176	-0.4788	down	LevelBi	HMDB0000244;HMDB0247501;PW_C000165	377.1441	2.9447	M+H
Ethyl D-Allothreoninate	2.171E-6	1.914E-5	1.1013	0.6462	-0.6299	down	LevelBi	-	130.0849	1.0591	M+H-H2O
5,6,7,8-Tetrahydroquinolin-8-Ol	0.0002063	0.0006183	1.1036	0.7639	-0.3886	down	LevelBi	-	132.0797	2.4099	M+H-H2O
(3S,5R,6R,7E)-3,5,6-Trihydroxy-7-Megastigmen-9-One	4.394E-5	0.000173	1.0999	0.5794	-0.7875	down	LevelBii	HMDB0038736	225.1476	3.133	M+H-H2O

Carnitine	2.218E-9	1.225E-7	1.0997	1.2126	0.2781	up	LevelBi	-	162.1121	0.6983	M+H
Propyleneglycol Diacetate	5.073E-7	6.264E-6	1.0982	0.6186	-0.6929	down	LevelBii	HMDB0041605	181.0483	3.0543	M+Na-2H
Thr-Met-Phe	6.376E-5	0.0002246	1.0992	0.5484	-0.8668	down	LevelBi	-	398.1716	3.2023	M+H
2-Amino-5-[(1-Carboxy-2-Phenylethyl)Amino]-5-Oxopentanoic Acid	0.003651	0.007489	1.1031	0.6491	-0.6235	down	LevelBi	-	295.1272	2.766	M+H
Hophe-Trp-Oh	0.0003136	0.0008856	1.0954	2.0921	1.0649	up	LevelBi	-	472.1565	2.1976	M-H
Quinoline-4,8-Diol	0.0007676	0.001912	1.1009	1.8779	0.9091	up	LevelBii	HMDB0060289	160.0396	3.4048	M-H
Asn-Trp	0.003005	0.006317	1.0922	0.6109	-0.7109	down	LevelBi	HMDB0028742	319.1395	2.6069	M+H
Thr-Val-Phe	0.0005632	0.001466	1.0926	0.7242	-0.4655	down	LevelBi	-	366.2006	2.9844	M+H
Narirutin	2.652E-7	3.813E-6	1.0986	1.3703	0.4545	up	LevelBi	HMDB0033740;LMPK12140236	545.1645	0.6983	M+H-2H2O
(-)-Zoapatanol	0.0419	0.06268	1.1035	0.272	-1.8781	down	LevelBii	HMDB0242237	337.2359	7.4709	M-H
Pg(Pgf1Alpha/16:0)	0.02719	0.0433	1.1053	1.4282	0.5142	up	LevelBii	HMDB0268652	840.5676	7.7639	M+NH4
(13Z,16Z,19Z)-Docosa-13,16,19-Trienoylcarnitine	0.03806	0.05773	1.0971	1.8215	0.8651	up	LevelBii	HMDB0241605	522.377	7.3685	M+FA-H
2,3-Dihydroxycarbamazepine	1.911E-5	0.0001087	1.0945	0.5484	-0.8666	down	LevelBii	HMDB0060673	327.0997	3.4048	M+Hac-H
Cinnamyl Phenylacetate	0.0001729	0.0005304	1.0934	0.4777	-1.0659	down	LevelBii	HMDB0037707	273.0884	1.9403	M+Na-2H
Ile-Glu-Leu	0.01106	0.01986	1.0909	0.5274	-0.923	down	LevelBi	-	374.2261	3.5573	M+H
Gluconic Acid	6.415E-6	4.52E-5	1.0882	0.6794	-0.5578	down	LevelBi	HMDB0000625	195.0501	0.671	M-H
Penta-1,3-Diene	3.408E-7	4.621E-6	1.0859	0.7853	-0.3487	down	LevelBi	HMDB0256243	69.0701	1.6103	M+H

							i				
Plantainoside C	5.668E-7	6.749E-6	1.0858	0.7277	-0.4586	down	LevelBi	-	639.2265	3.6068	M+H
Fahfa(20:4/19:0)	0.004059	0.008219	1.0898	1.2582	0.3314	up	LevelBi	-	659.523	8.6961	M+Hac-H
Gln-Asp-Leu	0.0008723	0.002138	1.0815	0.5856	-0.7721	down	LevelBi	-	429.1966	1.9424	M+CH3OH+Na
Cannabigerolate	6.853E-5	0.0002392	1.087	0.7316	-0.451	down	LevelBii	HMDB0304284	395.2028	5.5172	M+Cl
Ricinoleic Acid	0.01572	0.02703	1.0818	0.6445	-0.6337	down	LevelBii	HMDB0034297	319.2264	7.978	M+Na-2H
1H-Indole-4-Carboxaldehyde	0.004889	0.009678	1.0877	1.4101	0.4958	up	LevelBi	-	146.0596	2.3901	M+H
Val-Trp	4.143E-5	0.000173	1.0801	0.791	-0.3382	down	LevelBi	HMDB0029138	304.1648	2.8754	M+H
Smgdg(O-13:1/7:0)	0.008001	0.01498	1.0877	0.6256	-0.6768	down	LevelBi	-	671.3259	6.9054	M+FA-H
Succinylcholine	0.004888	0.009677	1.0752	0.4085	-1.2916	down	LevelBi	HMDB0014347	273.2161	4.0993	M+H-H2O
Ser-Val-Phe	9.635E-5	0.0003218	1.0761	0.7144	-0.4852	down	LevelBi	-	352.1848	3.0437	M+H
Gingerenone A	0.002058	0.004517	1.0837	0.5227	-0.936	down	LevelBii	HMDB0035403	391.1317	2.7839	M+Cl
Phe-Glu	0.02432	0.03932	1.0895	1.5838	0.6634	up	LevelBi	HMDB0028994	295.1273	2.1512	M+H
Chlorphenesin	0.003591	0.007382	1.0783	0.3339	-1.5826	down	LevelBii	HMDB0014994	247.0395	0.6896	M+FA-H
Sphingosine	3.58E-5	0.000173	1.076	0.8077	-0.3082	down	LevelBi	HMDB0000252;LMSP01010001;PW_C000172	300.2877	6.8173	M+H
Capsianoside Vi	0.01231	0.02184	1.0824	0.6175	-0.6955	down	LevelBii	HMDB0039165	923.4767	6.2803	M+H
Guanidylic Acid (Guanosine Monophosphate)	0.0005603	0.001461	1.0742	0.7091	-0.4959	down	LevelBi	-	362.0492	2.0063	M-H
Blasticidin S	0.004628	0.009218	1.0759	0.6526	-0.6157	down	LevelBii	HMDB0030452	405.1999	1.9623	M+H-H2O

Trp-Leu	9.849E-5	0.0003276	1.0724	0.7762	-0.3656	down	LevelBi	HMDB0029087	318.1803	3.5671	M+H
Erythro-L-Galacto-Nonulose	1.946E-6	1.76E-5	1.0728	1.2937	0.3715	up	LevelBii	HMDB0029955	275.0723	0.776	M+Na-H2O
Floionolic Acid	0.01566	0.02693	1.0804	0.5828	-0.779	down	LevelBii	HMDB0034295	353.2311	6.9054	M+Na-2H
Paroxetine	4.913E-5	0.000181	1.0722	0.5351	-0.902	down	LevelBii	HMDB0014853	388.1594	1.8836	M+Hac-H
Cyclosquamosin F	0.004357	0.008747	1.0767	0.6297	-0.6672	down	LevelBii	HMDB0303371	819.3969	6.3694	M+FA-H
Ile-Met	0.0001334	0.0004239	1.0672	0.7038	-0.5067	down	LevelBii	HMDB0028913	261.1268	2.2829	M-H
Ethyl 1-(Cyanomethyl)-4-Piperidinecarboxylate	6.23E-9	2.438E-7	1.0715	1.2074	0.2719	up	LevelBi	-	229.154	0.7565	M+CH3OH+H
1-O-Hexadecyl-Sn-Glycero-3-Phosphocholine	0.0001053	0.0003469	1.0707	0.8675	-0.2051	down	LevelBii	HMDB0243890	482.3586	8.3392	M+H
Ile-Ser-Leu	0.0001089	0.0003568	1.0671	0.6324	-0.6612	down	LevelBi	-	332.2164	3.3703	M+H
Tridec-10-Enedioylcarnitine	0.00204	0.004482	1.0645	1.3385	0.4206	up	LevelBii	HMDB0241320	406.2189	5.8865	M+Na-2H
Lysope(16:1(9Z)/0:0)	0.004805	0.00953	1.0719	0.6536	-0.6136	down	LevelBi	HMDB0011504	452.2761	7.9885	M+H
6-Hydroxy-5-Methoxyindole Glucuronide	0.0002534	0.000736	1.0655	0.3276	-1.61	down	LevelBii	HMDB0010362	360.072	3.4613	M+Na-2H
Ps(6 Keto-Pgf1Alpha/16:1(9Z))	0.0005524	0.001444	1.0652	1.8602	0.8955	up	LevelBii	HMDB0281243	882.456	4.1204	M+Cl
N-Valylphenylalanine	0.0004318	0.001172	1.0635	0.8087	-0.3063	down	LevelBi	HMDB0255244	265.1546	2.6767	M+H
Ribostamycin	0.001234	0.002895	1.0705	0.6554	-0.6096	down	LevelBii	HMDB0257216	953.4543	5.6277	2M+FA-H
Mosinone A	0.03	0.05	1.0748	0.27	-1.869	dow	Lev	HMDB0031228	665.	9.634	M+FA-H

	314	129		37	2	n	elBii		4608		
3-O-Methylniveusin A	1.527E-5	9.048E-5	1.0648	0.748	-0.4189	down	LevelBii	HMDB0039084	409.1821	1.9424	M+H
Val-Glu	3.261E-5	0.0001673	1.0609	0.778	-0.3622	down	LevelBi	HMDB0029126	247.1287	1.2748	M+H
Gamabufotalin	0.0004631	0.0001246	1.0589	0.6988	-0.5171	down	LevelBii	HMDB0252604	407.2227	2.3104	M+Na-H2O
Dodecanamide,N-[3-(Dimethyloxidoamino)Propyl]	0.02045	0.03389	1.0546	0.5925	-0.7551	down	LevelBi	-	301.2839	5.9375	M+H
Harman	2.455E-7	3.612E-6	1.062	0.7414	-0.4317	down	LevelBi	HMDB0035196	183.0905	3.0437	M+H
Lps(22:6)	0.0001415	0.0004467	1.062	1.4508	0.5368	up	LevelBi	LMGP03050013	568.2636	8.3544	M-H
6"-O-Malonyldaidzin	1.359E-5	8.262E-5	1.0586	1.4596	0.5455	up	LevelBii	HMDB0041263	537.0771	1.2026	M+Cl
Met-Val-Phe	0.0002583	0.0007487	1.0552	0.6341	-0.6572	down	LevelBi	-	396.1931	3.5276	M+H
4-O-Methyl-Myo-Inositol	4.277E-5	0.000173	1.0567	1.4576	0.5436	up	LevelBii	HMDB0029915	215.0527	1.9024	M+Na-2H
Canrenoic Acid	0.0001936	0.0005851	1.0567	0.698	-0.5187	down	LevelBii	HMDB0249591	393.1831	1.9876	M+Cl
Laurocapram	0.0001054	0.000347	1.0532	0.8119	-0.3006	down	LevelBii	HMDB0253992	282.2785	6.8173	M+H
N(4)-Oleylcytosine Arabinoside	0.04353	0.06477	1.0533	0.4831	-1.0496	down	LevelBii	HMDB0255284	528.3245	9.001	M+Cl
Arg-Ile	1.499E-5	8.916E-5	1.0548	2.0153	1.011	up	LevelBii	HMDB0028712	286.1876	1.8648	M-H
N-L-Gamma-Glutamyl-L-Leucine	7.582E-6	5.192E-5	1.0515	0.7405	-0.4334	down	LevelBi	HMDB0011171	261.1461	2.5469	M+H
Val-Leu	9.657E-5	0.0003223	1.0499	0.7974	-0.3267	down	LevelBi	HMDB0029131	231.1695	2.2806	M+H

Gibberellin A39	0.0008402	0.002071	1.0504	0.6278	-0.6716	down	LevelBii	HMDB0035045	429.1339	2.7839	M+Cl
Quetiapine	0.00103	0.00247	1.0466	0.4624	-1.1128	down	LevelBii	HMDB0005021	404.1421	2.0157	M+Na-2H
Dextran-70	3.367E-6	2.7E-5	1.0466	0.7796	-0.3592	down	LevelBii	HMDB0256672	397.2089	2.0614	M+H
12,15-Epoxy-13,14-Dimethyloctadeca-12,14,16-Trienoic Acid	0.01629	0.02788	1.0599	0.4375	-1.1925	down	LevelBii	HMDB0112071	355.2076	6.8774	M+Cl
Ganosporeric Acid A	0.005912	0.01148	1.0493	2.2343	1.1598	up	LevelBii	HMDB0033022	527.2611	3.2715	M+H
Citreoviridin D	1.136E-5	7.196E-5	1.0451	0.6147	-0.702	down	LevelBii	HMDB0030522	417.2309	2.5469	M+H
Ps(6 Keto-Pgf1Alpha/22:4(7Z,10Z,13Z,16Z))	0.004237	0.008541	1.0502	0.5996	-0.7378	down	LevelBii	HMDB0283217	946.4974	5.0105	M+Na-2H
9S-Hydroxy-10E,12Z,15Z-Octadecatrienoic Acid	0.002015	0.004429	1.0492	0.7605	-0.3949	down	LevelBi	LMFA02000024	295.2256	6.641	M+H
2-(1-Piperidinyl)Ethanol	3.441E-6	2.75E-5	1.0416	0.6959	-0.5231	down	LevelBi	-	112.1114	0.4715	M+H-H2O
Quinic Acid	2.609E-5	0.0001394	1.0403	0.5877	-0.7669	down	LevelBii	HMDB0003072	213.0368	2.9415	M+Na-2H
gamma-Glu-Val	0.0001391	0.0004404	1.0386	0.741	-0.4324	down	LevelBii	HMDB0011172	245.1136	1.2774	M-H
Sumithrin	0.0223	0.03649	1.0387	0.4075	-1.295	down	LevelBi	HMDB0242366	368.221	2.3004	M+NH4
20,26-Dihydroxyecdysone	0.04874	0.07149	1.028	0.2916	-1.7781	down	LevelBii	HMDB0245570	555.3161	3.2619	M+Hac-H
Pyroglutamylleucine	0.04137	0.062	1.0481	1.8969	0.9236	up	LevelBii	HMDB0341382	241.1185	2.2165	M-H
Asp-Ile	1.19E-5	7.439E-5	1.0377	0.7053	-0.5038	down	LevelBi	HMDB0028756	247.1313	4.6876	M+H
Trp-Lys	0.004216	0.0085	1.0339	0.6412	-0.6412	down	LevelBii	HMDB0029088	391.197	3.4141	M+Hac-H

Ala-Ile-Met	1.902E-5	0.0001083	1.0349	0.708	-0.4981	down	LevelBi	-	334.1773	2.6267	M+H
2-[Methyl(3-Phenylpropanoyl)Amino]Benzoic Acid	9.159E-5	0.0003088	1.0362	0.6433	-0.6364	down	LevelBi	HMDB0032845	342.1322	4.1484	M+Hac-H
7-Methylguanine	0.0002369	0.0006954	1.0321	0.5867	-0.7693	down	LevelBi	HMDB0000897	375.1285	3.9531	2M+FA-H
Trp-Val-Phe	0.005826	0.01133	1.0271	0.5168	-0.9522	down	LevelBi	-	451.2308	4.2174	M+H
18-Carboxy-Dinor-Lte4	0.02947	0.04644	1.0404	0.6055	-0.7238	down	LevelBi	HMDB0012607	883.3675	6.0742	2M+H
Guanadrel	0.0001937	0.0005851	1.0284	0.7278	-0.4584	down	LevelBi	HMDB0014371	258.1448	1.8555	M+FA-H
Phe-Thr	0.00004271	0.000116	1.026	0.745	-0.4246	down	LevelBi	HMDB0029005	267.1332	2.1311	M+H
2-(4-Chlorophenyl)-1-[3-(3-Cyclopropyl-1,2,4-Triazol-4-Yl)Azetidin-1-Yl]Ethanone	5.6E-6	4.062E-5	1.0288	1.274	0.3494	up	LevelBi	-	317.1194	3.0735	M+H
Methyl (2R)-Piperidine-2-Carboxylate	7.83E-7	8.624E-6	1.0284	0.7001	-0.5144	down	LevelBi	-	144.1011	0.9516	M+H
Peridinin	0.01269	0.02239	1.0326	1.6368	0.7109	up	LevelBi	HMDB0256341	635.3364	2.517	M+Na-H2O
Ethiofencarb	9.751E-5	0.000325	1.0289	1.5228	0.6067	up	LevelBi	HMDB0031782	260.0525	3.1865	M+Cl
Lnape(13:1/N-7:0)	0.02886	0.04564	1.0189	0.3641	-1.4575	down	LevelBi	-	580.3235	6.4803	M+Hac-H
12-Hydroxy-12-Octadecanoylcarnitine	0.01314	0.0231	1.0285	1.4045	0.49	up	LevelBi	HMDB0013154	488.3563	7.1456	M+FA-H
Cer(9:0_3O/18:2_(2Oh))	0.04383	0.06515	1.025	1.3752	0.4596	up	LevelBi	-	470.3809	7.2361	M+H
Cdp-Dg(5-Iso Pgf2Vi/22:3(10Z,13Z,16Z))	0.03259	0.05055	1.0316	0.6971	-0.5205	down	LevelBi	HMDB0292368	1116.5494	6.5251	M+H
beta-Asp-Leu	0.01549	0.02669	1.0334	1.5433	0.626	up	LevelBi	HMDB0011166	245.1136	2.2638	M-H
Methyl 5-Hydroxyoxindole-3-Acetate	0.00	0.00	1.025	0.44	-1.155	dow	Lev	HMDB0038990	242.	4.3059	M+Na-2H

	04542	1224		9	3	n	elBii		0423		
Desacetoxyvindoline	0.002707	0.005753	1.0188	0.6022	-0.7318	down	LevelBii	HMDB0304331	433.1936	2.623	M+Cl
Fahfa(18:2/22:4)	0.007405	0.01401	1.021	0.651	-0.6194	down	LevelBi	-	631.4681	8.8239	M+Na-2H
Mg(18:1(9Z)/0:0/0:0)	0.03109	0.04853	1.0261	1.2663	0.3406	up	LevelBi	HMDB0011567	357.2987	8.7745	M+H
2-Methylthio-N6-(Delta2-Isopentenyl)Adenosine	0.00187	0.004157	1.0111	0.6297	-0.6672	down	LevelBii	HMDB0301782	362.1313	3.273	M-H2O-H
Thr-Pro	2.15E-5	0.0001198	1.0133	0.3196	-1.6456	down	LevelBii	HMDB0029069	215.1028	1.846	M-H
(2S)-2-[[(2R)-2-Amino-3-Carboxypropanoyl]Amino]-4-Methylpentanoic Acid	0.0003673	0.00102	1.0179	1.2838	0.3604	up	LevelBi	-	247.1287	2.2706	M+H
Methylpyrazine	1.975E-5	0.0001115	1.0148	0.7784	-0.3615	down	LevelBii	HMDB0033112	95.06	0.6298	M+H
Val-Pro-Leu	9.425E-5	0.0003158	1.0113	0.7727	-0.372	down	LevelBi	-	328.2205	2.7362	M+H
Camphorsulfonic Acid	0.0003237	0.0009106	1.0113	1.4109	0.4966	up	LevelBii	HMDB0243496	267.0448	3.5457	M+Cl
N-Cyclopentyl-2-(Diethylamino)-2-(6-Nitro-1,3-Benzodioxol-5-Yl)Acetamide	0.001113	0.002643	1.0077	0.6943	-0.5265	down	LevelBi	-	364.1874	2.786	M+H
1-Dodecyl-1H-Indole-2,3-Dione	0.0004109	0.001124	1.012	0.7573	-0.4011	down	LevelBi	-	316.23	7.969	M+H
N-Acetyl-1-Aspartylglutamic Acid	3.204E-5	0.0001648	1.0092	0.7642	-0.388	down	LevelBi	HMDB0255414	305.0964	1.8827	M+H
[6]-Gingerdiol 3,5-Diacetate	0.002003	0.004408	1.0151	0.767	-0.3827	down	LevelBi	HMDB0040568	379.2086	6.6467	M-H
Pg(Pge1/I-16:0)	0.0307	0.04805	1.0139	1.6143	0.6909	up	LevelBii	HMDB0271557	838.5513	7.6294	M+NH4
Coumarin	0.03006	0.0472	1.0174	1.6498	0.7223	up	LevelBi	HMDB0001218	147.0427	1.8436	M+H
Ethyl Palmitoleate	0.001632	0.003697	1.008	0.7842	-0.3508	down	LevelBi	-	283.2614	9.0795	M+H

Val-Val-Asp	1.996E-6	1.795E-5	1.0045	0.8158	-0.2936	down	LevelBi	-	332.1806	2.0116	M+H
Thr-Lys	2.669E-5	0.0001418	1.0045	0.7828	-0.3533	down	LevelBii	HMDB0029066	270.1432	1.9821	M+Na
Estragonoside	0.00565	0.01102	1.0121	0.5716	-0.807	down	LevelBii	HMDB0033650	499.0821	4.1298	M+Na-2H
Ethyl 1-Boc-3-Allylpiperidine-3-Carboxylate	0.0025777	0.0007475	1.006	1.3952	0.4805	up	LevelBi	-	330.2279	4.0993	M+CH3OH+H
3-Anisaldehyde	0.01008	0.01833	1.0104	0.7618	-0.3925	down	LevelBi	HMDB0031459	137.059	6.7388	M+H
Thr-Pro-Pro	2.269E-7	3.409E-6	1.002	0.811	-0.3022	down	LevelBi	-	314.172	2.0712	M+H
Phe-Hyp	5.13E-5	0.0001873	1.0008	0.7643	-0.3877	down	LevelBii	HMDB0011176	261.1224	3.2023	M+H-H2O
Ala-Trp	0.02889	0.04567	0.994	0.6147	-0.7021	down	LevelBi	HMDB0013209	276.1332	2.6467	M+H
4-(Dimethylamino)Azobenzene N-Oxide	0.002564	0.00548	0.997	0.7212	-0.4714	down	LevelBi	-	281.0942	3.2322	M+K
Pindolol	0.0016066	0.0049755	1.0001	0.7113	-0.4915	down	LevelBii	HMDB0015095	293.1497	2.4722	M+FA-H
Arbutin	6.064E-6	4.317E-5	1.0017	0.6696	-0.5786	down	LevelBii	HMDB0029943	293.0631	2.5476	M+Na-2H
